# A Redox‐active Cyclometalated Platinum Ring Enables Synthetic Post‐processing of a [2]Rotaxane

**DOI:** 10.1002/anie.202415381

**Published:** 2024-10-31

**Authors:** Raksha Kandel, Miguel A. Soto, Daniel Medina, Brian O. Patrick, Francesco Lelj, Mark J. MacLachlan

**Affiliations:** ^1^ Department of Chemistry University of British Columbia 2036 Main Mall Vancouver BC V6T 1Z1 Canada; ^2^ La.M.I. and LaSCAMM INSTM Sezione Basilicata, Dipartimento di Scienze Università della Basilicata Via dell'Ateneo Lucano 10 85100 Potenza Italy; ^3^ Stewart Blusson Quantum Matter Institute University of British Columbia 2355 East Mall Vancouver BC V6T 1Z4 Canada; ^4^ WPI Nano Life Science Institute Kanazawa University Kanazawa 920-1192 Japan

**Keywords:** rotaxanes, platinum, redox, metal-metal bond, supramolecular chemistry

## Abstract

Post‐synthetic modification of mechanically interlocked molecules (MIMs) is an attractive avenue to add complexity to already intricate systems. This remains an important, challenging topic that is under‐developed. In this paper, we report the synthesis and characterization of a [2]rotaxane molecule featuring a ring appended to an emissive cyclometalated Pt^II^ unit. Modulation of the oxidation state at the metal center can transform the interlocked molecule into a new Pt^IV^ [2]rotaxane or a Pt^III^ [3]rotaxane held together by an intermetallic Pt−Pt bond – a first of its kind. These molecules display distinct structural and photophysical properties, as well as shuttling dynamics. This approach for post‐synthetic modification could be used to construct more complex MIMs and inorganic supramolecular assemblies with redox properties.

## Graphical abstract

A new platinum‐containing rotaxane was developed and underwent post‐synthetic modification via modulation of the redox properties of the metal center. Oxidation using mild reagents led to rapid conversion to a Pt^IV^ complex and a Pt^III^ dimer with different photophysical and shuttling dynamics. Notably, this is the first known example where a Pt−Pt bond has been used as a bridge to access higher‐order rotaxanes.

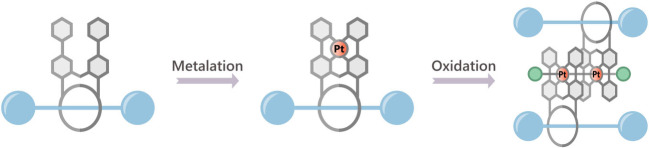

The intriguing topologies afforded by mechanical bonds have long fascinated chemists due to their structural complexity and potential applications. Significant progress has been made in synthesizing mechanically interlocked molecules (MIMs) such as catenanes,^[1]^ rotaxanes,^[2]^ knots,^[3]^ and Borromean rings.^[4]^ Among them, rotaxanes are one of the most studied MIMs partly due to the ever‐expanding repertoire of synthetic procedures[^5^, ^6^] that have led to their incorporation into different functional assemblies,[^7^, ^8^, ^9^, ^10^, ^11^] and versatile applications.[^12^, ^13^, ^14^, ^15^, ^16^, ^17^] While purely organic rotaxanes are more common, appropriate design and placement of donor atoms on the axle, macrocycle, or inside the cavity between the two have enabled the introduction of metal ions within rotaxanes,^[18]^ opening applications in catalysis,[^19^, ^20^, ^21^] sensing,^[22]^ and self‐assembly.^[23]^

Despite the diversity of accessable rotaxanes, examples of post‐synthetically processable rotaxanes remain rare.[^24^, ^25^] In general, rotaxanes can be synthetically modified on their axles, macrocycles, or, in some cases, both components after assembly. For instance, post‐functionalization of the axle has led to the formation of insulated molecular wires^[26]^ and metal‐organic rotaxane frameworks.[^18^, ^27^] Complexation of a metal ion with the axle and the macrocycle has been used to generate metal complexes with coordination geometries inaccessible in the equivalent non‐interlocked species.[^23^, ^28^] Likewise, post‐functionalization of macrocycles (the most commonly observed modification) has previously been used to switch between [2]rotaxanes and [3]rotaxanes,[^29^, ^30^] introduce intercomponent motion,^[31]^ append chelating species for bacterial binding,^[32]^ sense metal ions,^[33]^ and synthesize rotaxane‐crosslinked polymers.[^34^, ^35^]

Our group has recently focused on the investigation of redox‐active cyclometalated Pt^II^ complexes with compact backbones^[36]^ as well as more elaborate structures including crown ethers and pseudocrown ethers.[^37^, ^38^, ^39^] These species can undergo fast and reversible oxidation under mild conditions^[40]^ to generate Pt^III^ and Pt^IV^ species, which may be useful to develop new responsive systems and supramolecular assemblies.[^41^, ^42^] Many of these Pt‐containing rings have small cavities that can only interact with inorganic ions, thus preventing the assembly of threaded complexes and MIMs. Here, we report a ring‐expanded host (**1⋅H_2_**, Figure 1a) fused to a tetradentate phenylpyridine ligand that can form an emissive cyclometalated Pt complex and accommodate a large organic cation in its cavity, allowing the formation of an interlocked structure. This MIM, a [2]rotaxane, is redox‐active at its ring and, therefore, can be post‐synthetically transformed into a Pt^IV^ structure ([2]rotaxane) and a Pt^III^ dimer ([3]rotaxane), causing changes in the shuttling dynamics of the MIM and its emissive properties.

**Figure 1:**
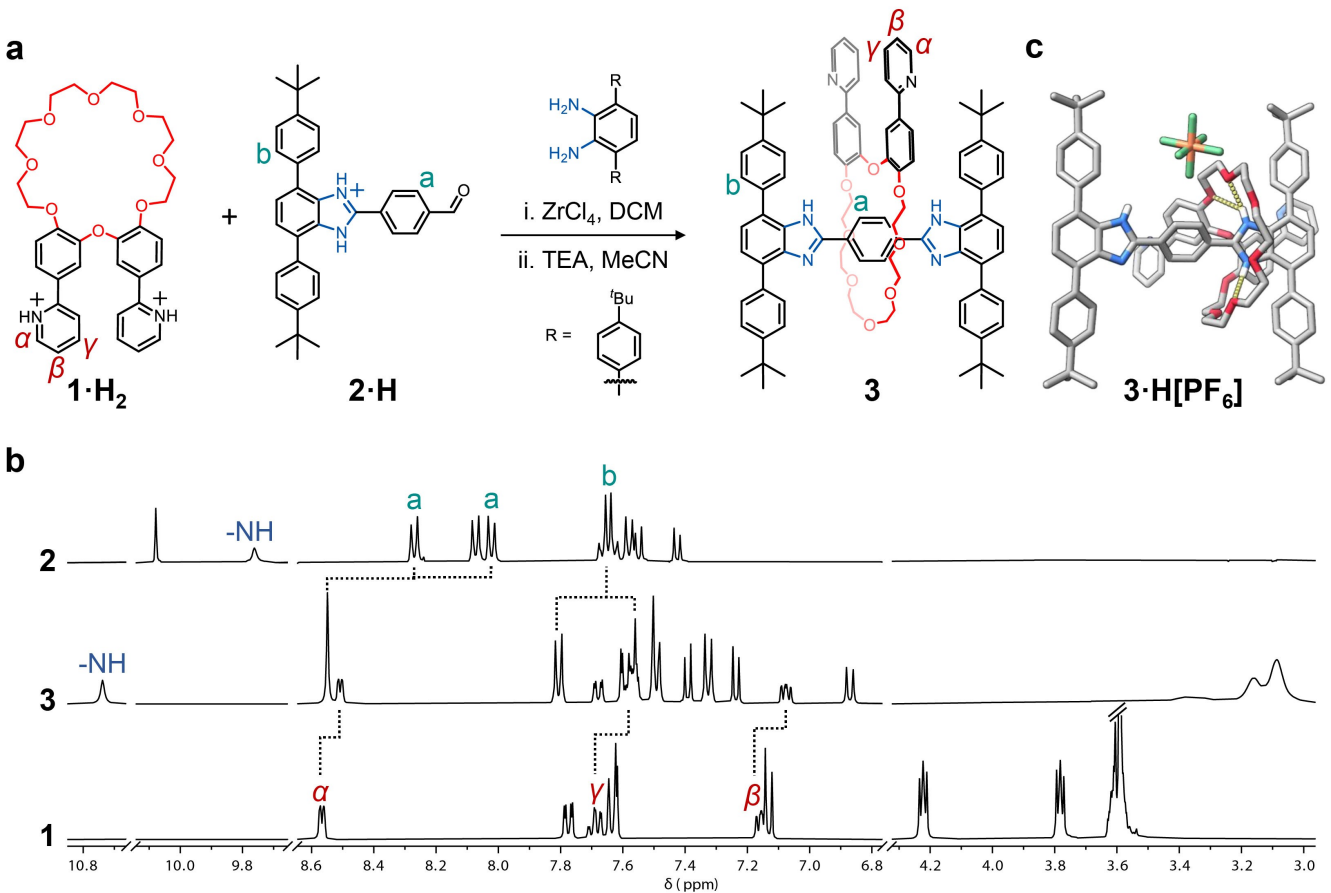
a. Synthesis of rotaxane **3** via threading‐followed‐by‐stoppering. Counterions (BF_4_^−^) for **1⋅H_2_** and **2⋅H** have been omitted for clarity; TEA=triethylamine; DCM=dichloromethane. b. ^1^H NMR spectra (400 MHz, CD_2_Cl_2_) of the neutral T‐shaped axle precursor **2** (top), the organic [2]rotaxane **3** (middle), and the macrocycle **1** (bottom). c. Solid‐state molecular structure of compound **3** as determined by SCXRD. Solvent molecules and most of the hydrogen atoms have been omitted for clarity (C=grey, O=red, N=blue, P=orange, F=green).

We first synthesized ligand **1** (Scheme S1) by adapting a published procedure.^[37]^ Starting from diphenyl ether, we obtained compound **1** in five steps with an overall yield of 19 % (Scheme S1, Figures S1–S12). For the guest, we chose a T‐shaped benzimidazolium (**2**), which has been extensively studied by Loeb's group (Scheme S2, Figures S13–S25).[^43^, ^44^, ^45^, ^46^] This organic cation shows excellent binding affinity to [24]‐membered crown ethers and contains a useful aldehyde handle to form rotaxanes via threading‐followed‐by‐stoppering.^[45]^

With the guest and host synthesized, we first tested their self‐assembly. Notably, mixing **1** with **2⋅H** in solution (CD_2_Cl_2_) did not lead to pseudorotaxane formation (Figure S26). This can be explained by the deactivation of **2⋅H** via proton transfer from **2⋅H** to **1** (imidazolium to pyridine). Protonating **1**, to generate **1⋅H_2_** (Scheme S1, Figures S27–S30), prevents proton transfer and allows strong intercomponent charge‐assisted hydrogen bonding to produce [2]pseudorotaxane **2** ⊂ **1⋅H_2_** (Figure S31). This intermediate was capped by a bulky diamine in a stoppering reaction (condensation followed by ZrCl_4_‐catalyzed oxidation), and then neutralized by triethylamine to produce [2]rotaxane **3** in 79 % yield (Figures 1a, S32–S45). The product was observed by high‐resolution mass spectrometry (HRMS) at *m/z*=1442.7706 Da (calc. [**3** + H]^+^ =1442.7709, see Figure S46).

Rotaxane **3** was characterized in solution by ^1^H nuclear magnetic resonance (NMR) spectroscopy (Figure 1b). The successful capture of the axle in the macrocyclic cavity was evidenced by 1) the splitting pattern of the glycolic protons in **1** (δ=3.1–4.0 ppm); 2) the disappearance of the aldehyde proton in **2** (δ=10.7 ppm), indicative of the successful condensation with the diamine stopper; and 3) the downfield shift of the NH proton (Δδ=0.98 ppm).^[45]^

The solid‐state structure of the rotaxane, isolated as **3**⋅H[PF_6_], was determined by single‐crystal X‐ray diffraction (SCXRD), confirming the mechanical interlocking between the macrocycle and the axle (Figure 1c). Similar to the structures reported by Loeb,^[45]^ NH⋅⋅⋅O hydrogen bonding interactions (2.05–2.70 Å, Figure 1c) and [N^+^⋅⋅⋅O] ion dipole interactions (2.85–2.99 Å, Figure S47a) are present in the solid state between the benzimidazolium and the crown ether. There are also weak CH⋅⋅⋅O (2.52–2.71 Å) interactions (Figure S47b) between the crown ether and the phenyl bridge on the axle. These short contacts lock the macrocycle predominantly on the protonated site of the axle. Interestingly, the two phenylpyridine moieties on the macrocycle are almost perpendicular to each other (ca. 109°) due to repulsion between neighboring hydrogen atoms (Figure S47d). This allows [N−H⋅⋅⋅N] hydrogen bonding interactions (2.12 Å) between neighboring rotaxanes, which propagates within the lattice (Figure S47c).

With the purely organic rotaxane characterized, we then performed a cyclometalation reaction using PtCl_2_ as the metal source^[47]^ to obtain Pt^II^ rotaxane **3‐Pt^II^** (Figure 2a, S48–S56) in 73 % yield. As expected, the proton signal ortho to the oxo‐bridge is no longer observed, indicating successful deprotonation followed by metalation. Resonances for the aromatic protons in the pyridyl ring of the ligand (e.g., proton *α*) are all shifted downfield owing to the presence of the electrophilic platinum center (Δδ_α_=0.43 ppm, Figure 2b). HRMS showed [**3‐Pt^II^** + H]^+^ at *m/z*=1635.7180 Da, which matches well with the calculated value (*m/z*=1635.7201 Da, Figure S57).

**Figure 2:**
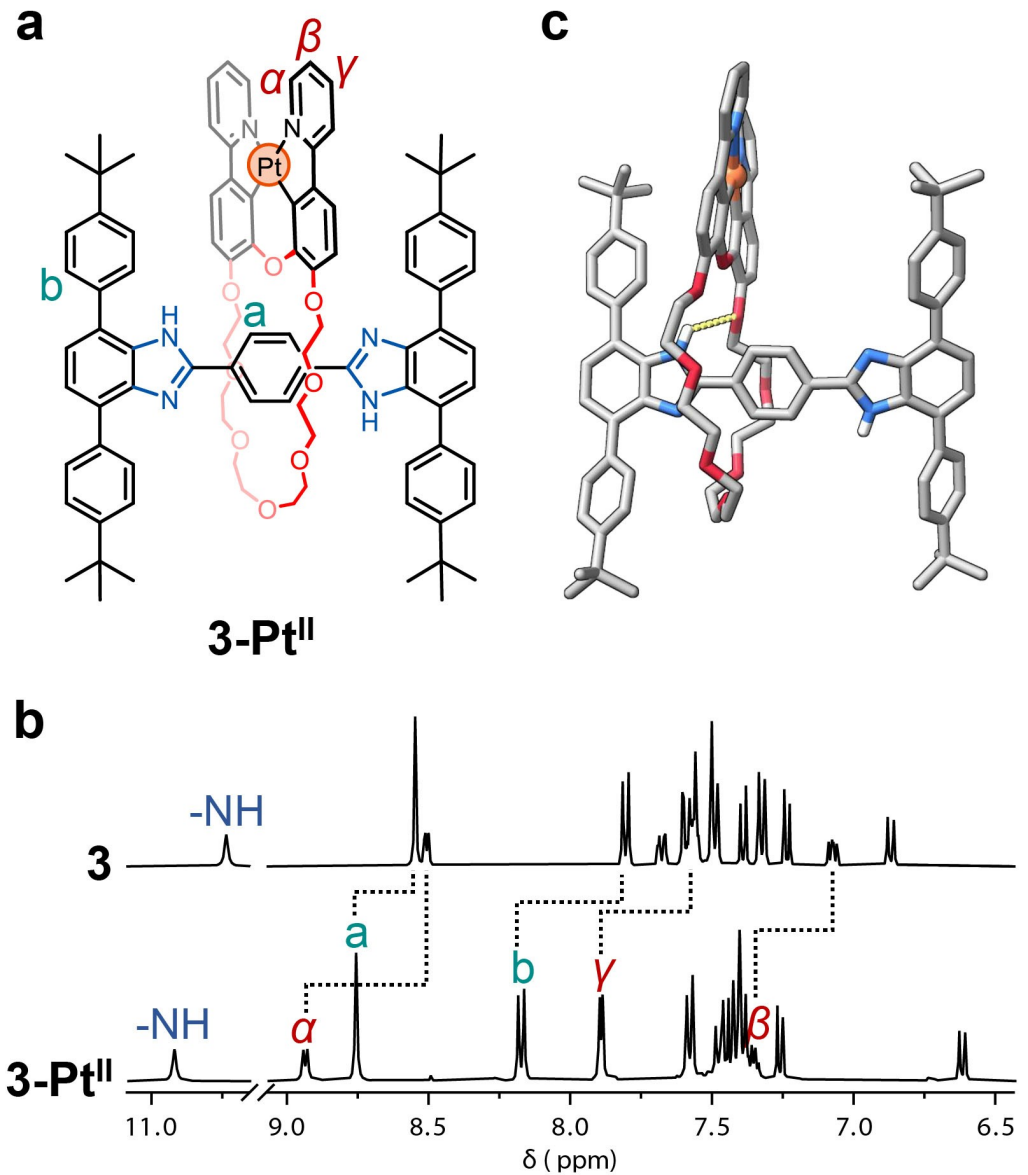
a. Chemical structure of **3‐Pt^II^**. b. ^1^H NMR spectra (400 MHz, CD_2_Cl_2_) of rotaxanes **3** (top) and **3‐Pt^II^** (bottom). c. Solid‐state molecular structure of **3‐Pt^II^** as determined by SCXRD. All solvent molecules and hydrogen atoms (except for the two on the benzimidazole) have been omitted for clarity (C=grey, O=red, N=blue, Pt=orange).

Analysis by SCXRD further confirmed the formation of **3‐Pt^II^** (Figure 2c). Upon cyclometallation, the two phenylpyridine moieties became almost planar with a small deviation of ca. 9° between the two planes (Figure S58b). The [N−H⋅⋅⋅O] hydrogen bonding (2.46 Å, Figure 2c) interactions as well as C−H⋅⋅⋅O (2.38–2.70 Å), and C−H⋅⋅⋅N (2.50–2.72 Å) interactions were observed between the crown ether and the axle (Figure S58a,b). The pyridyl nitrogen atom is no longer available for hydrogen bonding due to metalation; instead, π⋅⋅⋅π interactions (3.36 Å) are observed between one of the pyridyl rings of the neighboring rotaxanes (Figure S58c). The Pt⋅⋅⋅Pt distance between macrocycles of adjacent rotaxanes is too large (ca. 6.84 Å) for there to be any Pt⋅⋅⋅Pt interactions. The bulky stoppers likely prevent the phenylpyridine moieties from coming close together in the solid state, thus impeding the formation of metallophilic interactions. This is in contrast to the typical Pt^II^⋅⋅⋅Pt^II^ contacts (*d*(Pt^II^⋅⋅⋅Pt^II^) <3.5 Å) observed for a similar Pt‐containing macrocycle.^[37]^

Our group has previously shown that controlled oxidation of cyclometalated Pt^II^ structures can selectively yield Pt^III^ and Pt^IV^ species with structural and photophysical features distinct from their Pt^II^ counterpart.^[36]^ After isolating **3‐Pt^II^**, we wondered if this cyclometalated structure would remain redox‐active once it forms part of a MIM, especially because of the sterically demanding axle. We were also curious if these transformations on the rotaxane would change some properties of the MIM itself, such as its photophysical properties, stability, and shuttling dynamics.

To start, a solution of rotaxane **3‐Pt^II^** in CD_2_Cl_2_ (2.2×10^−3^ M) was treated with 1.1 equiv. of PhICl_2_ and monitored by ^1^H NMR spectroscopy. We expected PhICl_2_ to add two chloro ligands on the axial positions of the platinum center.[^36^, ^40^] Immediately after adding the oxidant, the bright yellow solution turned pale yellow, and some of the signals belonging to **3‐Pt^II^** in the ^1^H NMR spectrum disappeared to give rise to new resonances (Figure S59). These changes were an early indication of the formation of **3‐Pt^IV^** (Figure 3a) via oxidation. After isolation (78 % yield) (Figures S60–S62), the ^1^H NMR spectrum of **3‐Pt^IV^** (Figure 3b) showed similar features to those of **3‐Pt^II^**, except all the pyridyl resonances (e.g., *α*, *β*, and *γ*) in the macrocycle shifted downfield compared to the Pt^II^ rotaxane. This is indicative of the presence of a more electrophilic metal center following oxidation. The signals corresponding to the aromatic protons in the axle mostly remained unchanged compared to those in **3‐Pt^II^**. HRMS indicated the formation of **3‐Pt^IV^** at *m/z*=1705.6547 Da (calc. [**3‐Pt^IV^** + H]^+^ = 1705.6578 Da, see Figure S63 for details). The solid‐state structure of **3‐Pt^IV^** further confirmed the successful synthesis of **3‐Pt^IV^** (Figure 3c). Interestingly, the planes containing the crown ether and the cyclometalated portions in **3‐Pt^II^** (Figure 2c) are almost parallel with a dihedral angle (θ) of ca. 8°, whereas in **3‐Pt^IV^** (Figure 3c), θ is 41°. We believe that this distorted conformation helps to avoid steric clash between the chloro ligands and the tert‐butyl phenyl groups in **3‐Pt^IV^**.

**Figure 3:**
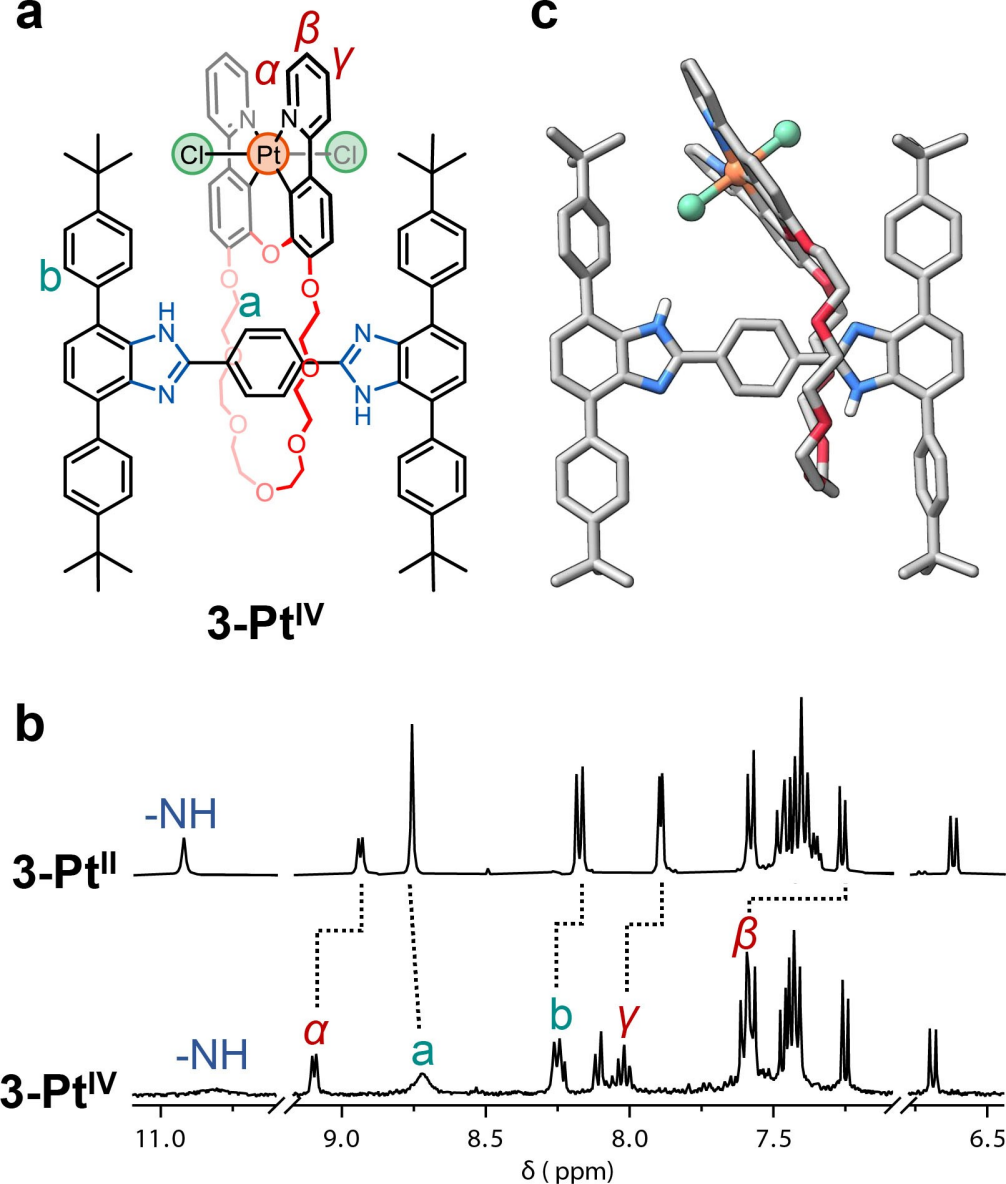
a. Chemical structure of **3‐Pt^IV^**. b. ^1^H NMR spectra (400 MHz, CD_2_Cl_2_) of **3‐Pt^II^** (top) and **3‐Pt^IV^** (bottom). c. Solid‐state molecular structure of **3‐Pt^IV^** as determined by SCXRD. All hydrogen atoms (except for the two on the benzimidazoles) and solvent molecules have been omitted for clarity (C=grey, O=red, N=blue, Pt=orange, Cl=green).

On the other hand, *N*‐chlorosuccinimide (NCS) was used to oxidize rotaxane **3‐Pt^II^** into a dimeric Pt^III^ species, **3‐Pt^III^** (Figure 4a). Previously, our group showed that a cyclometalated Pt^II^ crown ether dimerizes upon treatment with NCS via a single Pt^III^‐Pt^III^ bond.^[39]^ We anticipated that the oxidation of **3‐Pt^II^** by this method would generate an intermetallic‐bonded MIM, a [3]rotaxane. We first tested the reaction in an NMR tube. A solution of **3‐Pt^II^** (6.4×10^−3^ M) in CD_2_Cl_2_ was treated with 1.1 equiv. of NCS at room temperature. The color of the solution immediately changed from yellow to orange and the ^1^H NMR spectrum of the system showed the disappearance of **3‐Pt^II^** signals along with the appearance of new resonances belonging to a Pt^III^ species (Figure 4b).

**Figure 4:**
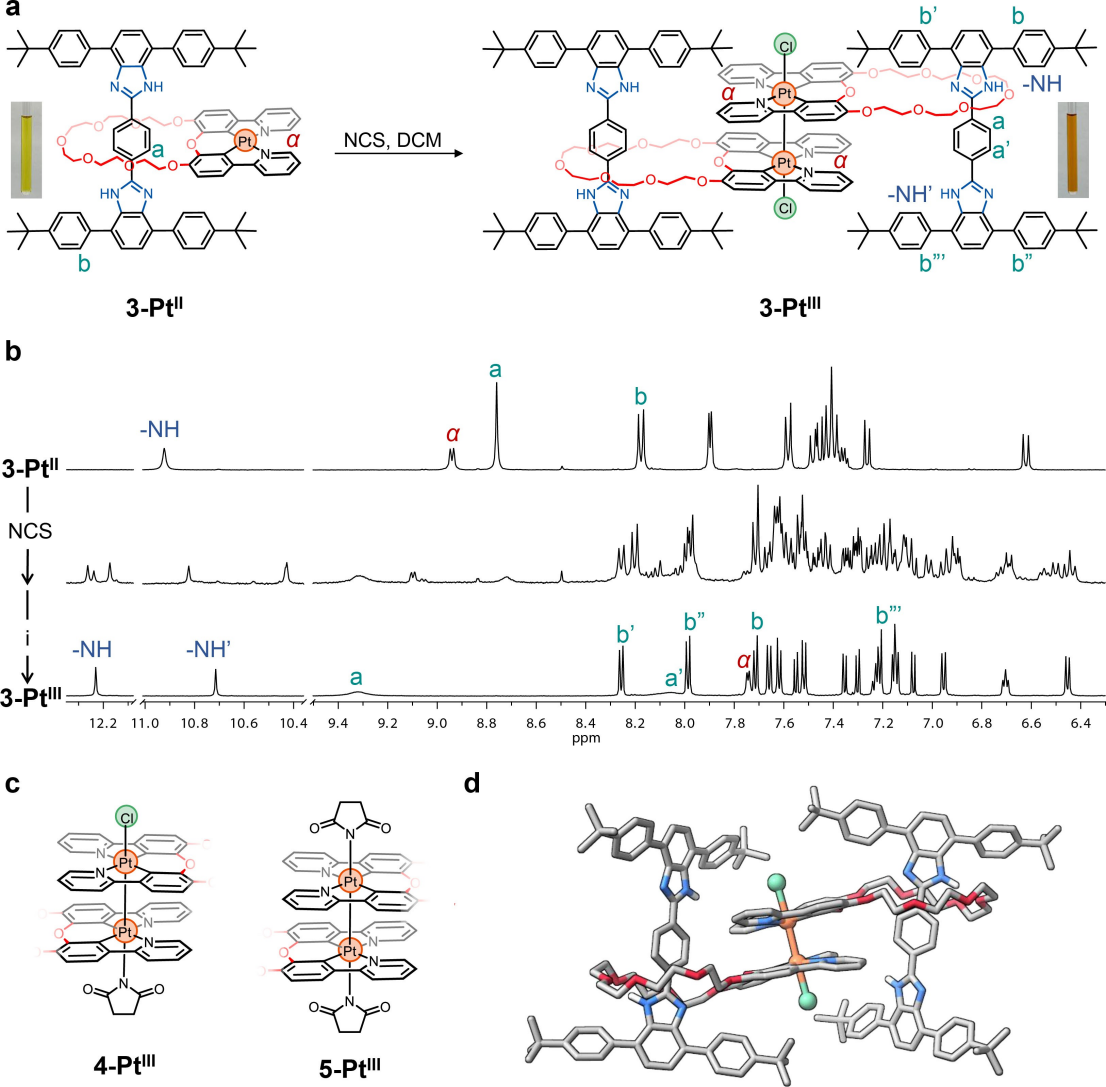
a. Synthesis of [3]rotaxane **3‐Pt^III^** via oxidation of the platinum center in [2]rotaxane **3‐Pt^II^**. Images of the purified **3‐Pt^II^** and **3‐Pt^III^** in CD_2_Cl_2_ are shown next to their respective chemical structures. b. (from top to bottom) ^1^H NMR spectra (600 MHz, CD_2_Cl_2_) of **3‐Pt^II^**, the crude product after adding NCS, and finally **3‐Pt^III^** (i=brine wash followed by size‐exclusion chromatography). c. Oxo‐bridged bis(phenylpyridine) portions of possible [3]rotaxanes formed after adding NCS and before brine wash. d. Computed structure of **3‐Pt^II^**. All hydrogen atoms except for the two on the benzimidazole have been omitted for clarity (C=grey, O=red, N=blue, Pt=orange, Cl=green).

We attributed the complexity of the resulting ^1^H NMR spectrum (Figure 4b, middle) to the formation of chloro and *N*‐bound succinimido ligands coordinated to the Pt center in axial positions as shown in Figure 4c; related species were previously observed in a Pt‐containing cyclometalated complex.^[39]^ When the orange solution (reaction crude product) was washed with saturated brine, the NMR spectrum of the resulting product showed only one set of signals associated with **3‐Pt^III^**, with minor impurities present (Figure S64) – the labile succinimido ligands were replaced with chloro ligands. Compound **3‐Pt^III^** was purified by size exclusion chromatography and was isolated in 63 % yield (Figures S65–S75).

The ^1^H NMR spectrum of **3‐Pt^III^** is shown in Figure 4b (bottom). In contrast to Pt^II^ and Pt^IV^ species, the axle of **3‐Pt^III^** has a lower symmetry (i.e. the two ends are inequivalent), thus giving rise to a greater number of signals. We observed two sets of resonances for the NH protons (Figure 4b) and the phenyl protons (i.e., protons a and a’, Figures 4a,b), and four sets of signals for all the other protons on the axle (e.g., b, b’, b”, b”’; Figures 4a,b). The identical chemical environment around both macrocycles leads to only one set of signals for the ligand protons on the complexes. As we were unable to grow a single crystal of this molecule suitable for SCXRD, we modeled the complex using the Gaussian suite of programs G016.revC01^[48]^ and the hybrid PBE1PBE^[49]^ functional (Figure 4d). The computed structure clearly shows the reduced symmetry of the [3]rotaxane. Furthermore, the intermetallic Pt−Pt bond (2.781 Å, Figure S80) in the calculated structure is comparable with that obtained for another Pt−Pt bonded structure with similar ligands (2.717 Å, Figure S79).^[39]^

In addition to the post‐synthetic processability of **3‐Pt^II^** in solution, we were also interested in studying the effects that oxidation would have on the dynamics of the MIMs, namely on the shuttling of the ring along the axle. The rates of exchange for all three rotaxanes (**3‐Pt^II^**, **3‐Pt^III^**, and **3‐Pt^IV^**) were determined using the coalescence temperature method.^[50]^ At room temperature, **3‐Pt^II^** has the highest rate of exchange, with the macrocycle shuttling 3.1×10^5^ times per second (Table S5) between the two benzimidazole stations. For neutral rotaxanes with the same axle but different [24]‐membered macrocycles, Zhu et al. estimated a minimum shuttling rate of 10^7^ s^−1^.^[45]^ We believe the relative bulkiness of our Pt^II^ macrocycle, compared to the ones previously reported, poses a greater barrier for translation, thus leading to a lower shuttling rate.

Rotaxanes **3‐Pt^IV^** and **3‐Pt^III^** have even lower shuttling rates of 5.7×10^4^ s^−1^ and 2.0×10^3^ s^−1^, respectively (Table S5). The lower shuttling rate for **3‐Pt^IV^** compared to **3‐Pt^II^** rotaxane is likely due to the axial chloro ligands in the octahedral **3‐Pt^IV^** species, which slow down the translation of the macrocycle through sterically unfavorable interactions with the bulky stoppers. The steric congestion for **3‐Pt^III^**, on the other hand, is much greater since the Pt−Pt bond between the two macrocycles also brings two mechanically interlocked axles into close proximity.

These differences in shuttling behavior are further evidenced by the solvent‐excluded volumes of the cyclometalated portions of the molecules; based on our estimations, the values go from 308 Å^3^ to 362 Å^3^ and 749 Å^3^ for the Pt^II^, Pt^III^ and Pt^IV^ species, respectively (Figure S84). The effect of the shuttling rate is most prominently observed in the phenyl protons on the axle (i.e., proton a). Fast exchange of the macrocycle between the two benzimidazole sites on the axle in **3‐Pt^II^** produces a sharp signal for the proton, which becomes progressively broader when going from **3‐Pt^IV^** to **3‐Pt^III^** due to decreasing shuttling rate (Figure 5a).

**Figure 5:**
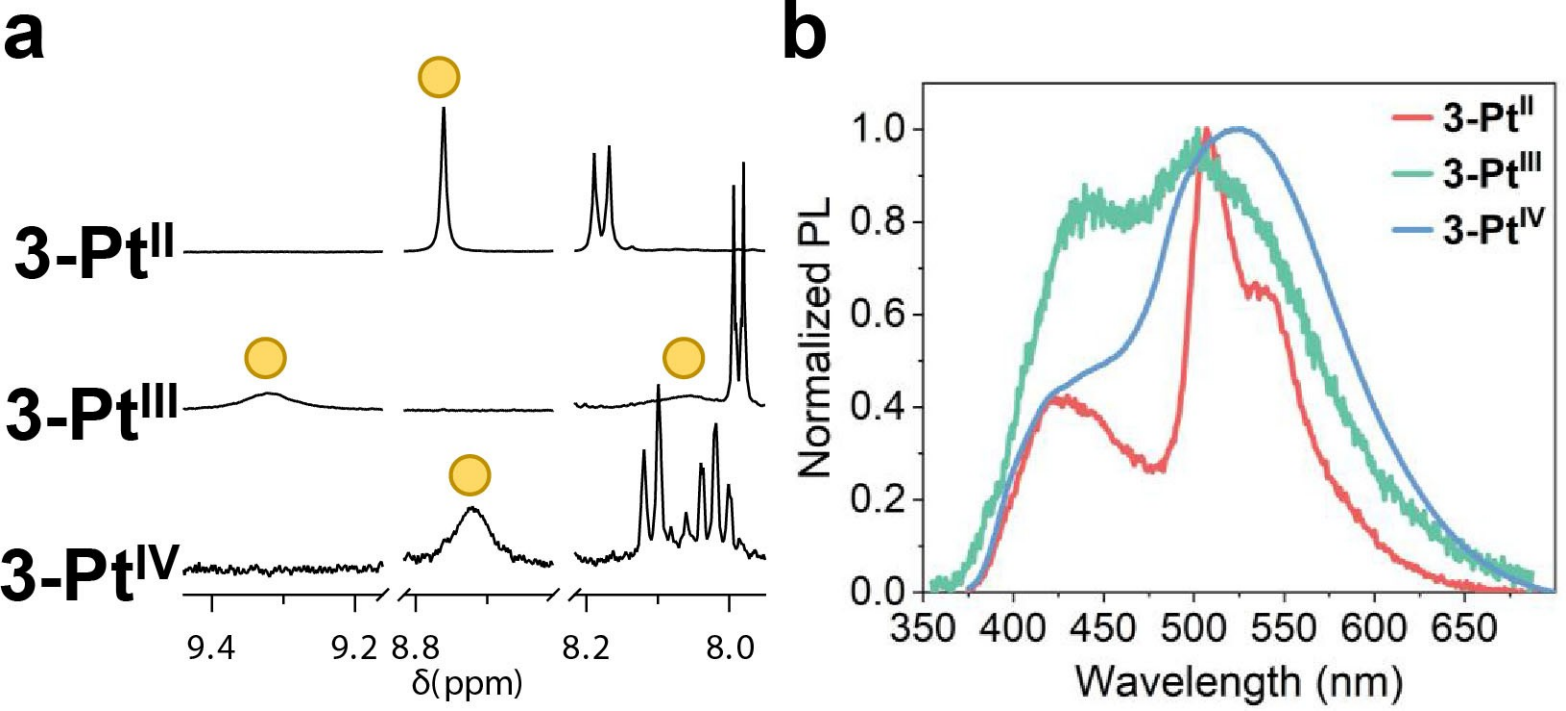
a. Stacked partial ^1^H NMR spectra (400 MHz, CD_2_Cl_2,_ 298 K) of **3‐Pt^II^**, **3‐Pt^III^**, and **3‐Pt^IV^** (from top to bottom) with phenyl protons a indicated by the yellow circles. b. Emission spectra for rotaxanes **3‐Pt^II^**, **3‐Pt^III^**, and **3‐Pt^IV^** (DCM).

Finally, we investigated the effects of the oxidation states on the photophysical properties of the MIMs. **3‐Pt^III^** and **3‐Pt^IV^** show distinct transitions for intra‐ligand (IL) and metal‐to‐ligand charge transfer (MLCT) bands in the recorded UV‐vis spectra compared to **3‐Pt^II^** (Figure S85a). Perhaps the most distinguishing feature between the three species is the unstructured band for **3‐Pt^III^** at 457 nm, which we ascribe to a metal‐to‐metal‐to‐ligand charge transfer (MMLCT) transition. The appearance of this band is consistent with the color change (yellow to orange) upon oxidation and the Pt−Pt bond formation. The band at *ca*. 428 nm for **3‐Pt^II^** is likely due to very weak Pt⋅⋅⋅Pt and π–π interactions favored by the square planar geometry of the macrocycle. On the other hand, **3‐Pt^IV^** contains neither of the bands due to its octahedral structure, which prevents both intermetallic bonding and Pt−Pt interactions (Figures S85a, S86).

Likewise, different emission bands are observed for **3‐Pt^II^** (λ_max_≈426, 507, 540 nm), **3‐Pt^III^** (λ_max_≈442, 503 nm), and **3‐Pt^IV^** (λ_max_≈441, 525 nm) (Figures 5b, S87). Their distinct emission colors are also depicted in the CIE plot (Figure S85b) for a better visual reference. It is worth noting that while free macrocycles containing the Pt^III^ and Pt^IV^ centers are non‐emissive, rotaxanes **3‐Pt^III^** and **3‐Pt^IV^** emit light upon photoexcitation. We believe this is due to the changes in electronic properties when the macrocycles are mechanically bonded with the axle. This offers a new avenue for exploration to harness properties otherwise inaccessible in free components.

In summary, by appending a cyclometalating ligand to a 24‐crown‐8 ether, we constructed a [2]rotaxane, **3**, that can bind to platinum to form a rotaxane containing a cyclometalated Pt^II^ unit appended to its ring (**3‐Pt^II^**). In addition to its distinct luminescence properties, the redox‐active platinum center afforded two new species, a [3]rotaxane (**3‐Pt^III^**) and a [2]rotaxane (**3‐Pt^IV^**) using mild oxidants. These rotaxanes display different shuttling dynamics and emission properties compared to **3‐Pt^II^**. Notably, to the best of our knowledge, **3‐Pt^III^** is the first reported rotaxane to be held in place via a Pt−Pt bond. This novel concept of modulating a metal's properties for post‐processing of a mechanically interlocked molecule could be used to add complexity and new functionality to an already dynamic system, enabling the synthesis of inorganic supramolecular assemblies with interesting properties.

## Supporting Information

The authors have cited additional references within the Supporting Information.[^35^, ^44^, ^45^, ^46^, ^47^, ^48^, ^49^, ^51^, ^52^, ^53^, ^54^, ^55^, ^56^, ^57^, ^58^, ^59^, ^60^, ^61^]

## Conflict of Interests

The authors declare no conflict of interest.

## Supporting information

As a service to our authors and readers, this journal provides supporting information supplied by the authors. Such materials are peer reviewed and may be re‐organized for online delivery, but are not copy‐edited or typeset. Technical support issues arising from supporting information (other than missing files) should be addressed to the authors.

Supporting Information

## Data Availability

The data that support the findings of this study are available in the supplementary material of this article.

## References

[1] G. Gil-Ramírez , D. A. Leigh , A. J. Stephens , Angew. Chem. Int. Ed. 2015, 54, 6110–6150. 10.1002/anie.201411619 PMC4515087 25951013

[2] H. Tian , Q.-C. Wang , Chem. Soc. Rev. 2006, 35, 361–374. 16565753 10.1039/b512178g

[3] S. D. P. Fielden , D. A. Leigh , S. L. Woltering , Angew. Chem. Int. Ed. 2017, 56, 11166–11194. 10.1002/anie.201702531 PMC5582600 28477423

[4] S. J. Cantrill , K. S. Chichak , A. J. Peters , J. F. Stoddart , Acc. Chem. Res. 2005, 38, 1–9. 15654731 10.1021/ar040226x

[5] Carson J. Bruns , J. Fraser Stoddart , The Nature of the Mechanical Bond, John Wiley & Sons, Ltd, 2016.

[6] M. Xue , Y. Yang , X. Chi , X. Yan , F. Huang , Chem. Rev. 2015, 115, 7398–7501. 25734835 10.1021/cr5005869

[7] H. V. Schröder , C. A. Schalley , Chem. Sci. 2019, 10, 9626–9639. 32110308 10.1039/c9sc04118d PMC7020790

[8] S. Mena-Hernando , E. M. Pérez , Chem. Soc. Rev. 2019, 48, 5016–5032. 31418435 10.1039/c8cs00888d

[9] H.-Y. Zhou , Q.-S. Zong , Y. Han , C.-F. Chen , Chem. Commun. 2020, 56, 9916–9936. 10.1039/d0cc03057k 32638726

[10] M. C. Jiménez , C. Dietrich-Buchecker , J.-P. Sauvage , Angew. Chem. Int. Ed. 2000, 39, 3284–3287. 10.1002/1521-3773(20000915)39:18<3284::aid-anie3284>3.0.co;2-7 11028078

[11] P. N. Taylor , M. J. O'Connell , L. A. McNeill , M. J. Hall , R. T. Aplin , H. L. Anderson , Angew. Chem. Int. Ed. 2000, 39, 3456–3460. 10.1002/1521-3773(20001002)39:19<3456::aid-anie3456>3.0.co;2-0 11091388

[12] P. Rajamalli , F. Rizzi , W. Li , M. A. Jinks , A. K. Gupta , B. A. Laidlaw , I. D. W. Samuel , T. J. Penfold , S. M. Goldup , E. Zysman-Colman , Angew. Chem. Int. Ed. 2021, 60, 12066–12073. 10.1002/anie.202101870 PMC8251797 33666324

[13] Y. Sagara , M. Karman , E. Verde-Sesto , K. Matsuo , Y. Kim , N. Tamaoki , C. Weder , J. Am. Chem. Soc. 2018, 140, 1584–1587. 29355316 10.1021/jacs.7b12405 PMC5806082

[14] Y. Sagara , M. Karman , A. Seki , M. Pannipara , N. Tamaoki , C. Weder , ACS Cent. Sci. 2019, 5, 874–881. 31139723 10.1021/acscentsci.9b00173 PMC6535770

[15] W. Yang , Y. Li , H. Liu , L. Chi , Y. Li , Small 2012, 8, 504–516. 22267051 10.1002/smll.201101738

[16] K. Yang , S. Chao , F. Zhang , Y. Pei , Z. Pei , Chem. Commun. 2019, 55, 13198–13210. 10.1039/c9cc07373f 31631211

[17] M. J. Langton , P. D. Beer , Acc. Chem. Res. 2014, 47, 1935–1949. 24708030 10.1021/ar500012a

[18] S. J. Loeb , Chem. Soc. Rev. 2007, 36, 226–235. 17264925 10.1039/b605172n

[19] P. Thordarson , E. J. A. Bijsterveld , A. E. Rowan , R. J. M. Nolte , Nature 2003, 424, 915–918. 12931181 10.1038/nature01925

[20] M. Galli , J. E. M. Lewis , S. M. Goldup , Angew. Chem. Int. Ed. 2015, 54, 13545–13549. 10.1002/anie.201505464 PMC4678423 26387887

[21] Y. Cakmak , S. Erbas-Cakmak , D. A. Leigh , J. Am. Chem. Soc. 2016, 138, 1749–1751. 26835978 10.1021/jacs.6b00303 PMC4805306

[22] M. Denis , J. Pancholi , K. Jobe , M. Watkinson , S. M. Goldup , Angew. Chem. Int. Ed. 2018, 57, 5310–5314. 10.1002/anie.201712931 PMC5947674 29537728

[23] J. E. M. Lewis , M. Galli , S. M. Goldup , Chem. Commun. 2016, 53, 298–312. 10.1039/c6cc07377h 27819362

[24] P. Waelès , M. Gauthier , F. Coutrot , Angew. Chem. Int. Ed. 2021, 60, 16778–16799. 10.1002/anie.202007496 32894812

[25] S. S. Razi , M. Marin-Luna , M. Alajarin , A. Martinez-Cuezva , J. Berna , Commun. Chem. 2024, 7, 170. 39098851 10.1038/s42004-024-01258-4 PMC11298525

[26] J. Terao , S. Tsuda , Y. Tanaka , K. Okoshi , T. Fujihara , Y. Tsuji , N. Kambe , J. Am. Chem. Soc. 2009, 131, 16004–16005. 19831359 10.1021/ja9074437

[27] V. N. Vukotic , S. J. Loeb , Chem. Soc. Rev. 2012, 41, 5896–5906. 22717946 10.1039/c2cs35141b

[28] M. Cirulli , A. Kaur , J. E. M. Lewis , Z. Zhang , J. A. Kitchen , S. M. Goldup , M. M. Roessler , J. Am. Chem. Soc. 2019, 141, 879–889. 30562470 10.1021/jacs.8b09715

[29] M. Franz , J. A. Januszewski , F. Hampel , R. R. Tykwinski , Eur. J. Org. Chem. 2019, 2019, 3503–3512.

[30] P. J. Altmann , A. Pöthig , Angew. Chem. Int. Ed. 2017, 56, 15733–15736. 10.1002/anie.201709921 29044899

[31] S. Santra , S. Bej , M. Nandi , P. Mondal , P. Ghosh , Dalton Trans. 2017, 46, 13300–13313. 28771266 10.1039/c7dt01364g

[32] T. Mohy El Dine , R. Jimmidi , A. Diaconu , M. Fransolet , C. Michiels , J. De Winter , E. Gillon , A. Imberty , T. Coenye , S. P. Vincent , J. Med. Chem. 2021, 64, 14728–14744. 34542288 10.1021/acs.jmedchem.1c01241

[33] S. Bej , M. Nandi , P. Ghosh , Dalton Trans. 2021, 50, 294–303. 33300925 10.1039/d0dt03645e

[34] J. Sawada , D. Aoki , S. Uchida , H. Otsuka , T. Takata , ACS Macro Lett. 2015, 4, 598–601. 35596280 10.1021/acsmacrolett.5b00242

[35] J. Sawada , D. Aoki , H. Otsuka , T. Takata , Angew. Chem. Int. Ed. 2019, 58, 2765–2768. 10.1002/anie.201813439 30600883

[36] M. A. Soto , V. Carta , R. J. Andrews , M. T. Chaudhry , M. J. MacLachlan , Angew. Chem. Int. Ed. 2020, 59, 10348–10352. 10.1002/anie.202002646 32222012

[37] M. A. Soto , V. Carta , M. T. Cano , R. J. Andrews , B. O. Patrick , M. J. MacLachlan , Inorg. Chem. 2022, 61, 2999–3006. 34797043 10.1021/acs.inorgchem.1c03178

[38] M. A. Soto , V. Carta , I. Suzana , B. O. Patrick , F. Lelj , M. J. MacLachlan , Angew. Chem. Int. Ed. 2023, 62, e202216029. 10.1002/anie.202216029 36426408

[39] M. A. Soto , M. T. Chaudhry , G. K. Matharu , F. Lelj , M. J. MacLachlan , Angew. Chem. Int. Ed. 2023, 62, e202305525. 10.1002/anie.202305525 37208297

[40] S. R. Whitfield , M. S. Sanford , Organometallics 2008, 27, 1683–1689.

[41] M. A. Soto , M. J. MacLachlan , Chem. Sci. 2024, 15, 431–441. 38179527 10.1039/d3sc05524h PMC10763547

[42] M. A. Soto , R. Kandel , M. J. MacLachlan , Eur. J. Inorg. Chem. 2021, 2021, 894–906.

[43] G. Gholami , K. Zhu , G. Baggi , E. Schott , X. Zarate , S. J. Loeb , Chem. Sci. 2017, 8, 7718–7723. 29568435 10.1039/c7sc03736h PMC5851341

[44] N. Noujeim , K. Zhu , V. N. Vukotic , S. J. Loeb , Org. Lett. 2012, 14, 2484–2487. 22551383 10.1021/ol300761q

[45] K. Zhu , V. N. Vukotic , N. Noujeim , S. J. Loeb , Chem. Sci. 2012, 3, 3265–3271.

[46] K. Zhu , V. N. Vukotic , S. J. Loeb , Angew. Chem. Int. Ed. 2012, 51, 2168–2172. 10.1002/anie.201108488 22259027

[47] I. Allison , H. Lim , A. Shukla , V. Ahmad , M. Hasan , K. Deshmukh , R. Wawrzinek , S. K. M. McGregor , J. K. Clegg , V. V. Divya , C. Govind , C. H. Suresh , V. Karunakaran , N. U. K. N. A. Ajayaghosh , E. B. Namdas , S.-C. Lo , ACS Appl. Electron. Mater. 2019, 1, 1304–1313.

[48] Gaussian 16, Revision C.01, M. J. Frisch, G. W. Trucks, H. B. Schlegel, G. E. Scuseria, M. A. Robb, J. R. Cheeseman, G. Scalmani, V. Barone, G. A. Petersson, H. Nakatsuji, X. Li, M. Caricato, A. V. Marenich, J. Bloino, B. G. Janesko, R. Gomperts, B. Mennucci, H. P. Hratchian, J. V. Ortiz, A. F. Izmaylov, J. L. Sonnenberg, D. Williams-Young, F. Ding, F. Lipparini, F. Egidi, J. Goings, B. Peng, A. Petrone, T. Henderson, D. Ranasinghe, V. G. Zakrzewski, J. Gao, N. Rega, G. Zheng, W. Liang, M. Hada, M. Ehara, K. Toyota, R. Fukuda, J. Hasegawa, M. Ishida, T. Nakajima, Y. Honda, O. Kitao, H. Nakai, T. Vreven, K. Throssell, J. A. Montgomery Jr., J. E. Peralta, F. Ogliaro, M. J. Bearpark, J. J. Heyd, E. N. Brothers, K. N. Kudin, V. N. Staroverov, T. A. Keith, R. Kobayashi, J. Normand, K. Raghavachari, A. P. Rendell, J. C. Burant, S. S. Iyengar, J. Tomasi, M. Cossi, J. M. Millam, M. Klene, C. Adamo, R. Cammi, J. W. Ochterski, R. L. Martin, K. Morokuma, O. Farkas, J. B. Foresman, D. J. Fox, Gaussian, Inc., Wallingford CT, **2016**.

[49] C. Adamo , V. Barone , J. Chem. Phys. 1999, 110, 6158–6170.

[50] A. D. Bain , G. J. Duns , in Anal. Spectrosc. Libr. (Eds.: Gy. Batta , K. E. Kövér , Cs. Szántay ), Elsevier, 1997, pp. 227–263.

[51] L. Ji , Z. Yang , Y. Zhao , M. Sun , L. Cao , X.-J. Yang , Y.-Y. Wang , B. Wu , Chem. Commun. 2016, 52, 7310–7313. 10.1039/c6cc03144g 27181693

[52] N. Huang , P. Wang , M. A. Addicoat , T. Heine , D. Jiang , Angew. Chem. Int. Ed. 2017, 56, 4982–4986. 10.1002/anie.201611542 28370738

[53] Y. Zhao , D. G. Truhlar , J. Phys. Chem. A 2005, 109, 5656–5667. 16833898 10.1021/jp050536c

[54] J.-D. Chai , M. Head-Gordon , Phys. Chem. Chem. Phys. 2008, 10, 6615–6620. 18989472 10.1039/b810189b

[55] W. J. Hehre , R. Ditchfield , J. A. Pople , J. Chem. Phys. 1972, 56, 2257–2261.

[56] M. M. Francl , W. J. Pietro , W. J. Hehre , J. S. Binkley , M. S. Gordon , D. J. DeFrees , J. A. Pople , J. Chem. Phys. 1982, 77, 3654–3665.

[57] D. Figgen , K. A. Peterson , M. Dolg , H. Stoll , J. Chem. Phys. 2009, 130, 164108. 19405562 10.1063/1.3119665

[58] G. Scalmani , M. J. Frisch , J. Chem. Phys. 2010, 132, 114110. 20331284 10.1063/1.3359469

[59] G. M. Sheldrick , Acta Crystallogr. Sect. Found. Adv. 2015, 71, 3–8. 10.1107/S2053273314026370 PMC4283466 25537383

[60] O. V. Dolomanov , L. J. Bourhis , R. J. Gildea , J. A. K. Howard , H. Puschmann , J. Appl. Crystallogr. 2009, 42, 339–341. 10.1107/S0021889811041161 PMC3236671 22199401

[61] G. M. Sheldrick , Acta Crystallogr. Sect. C 2015, 71, 3–8. 10.1107/S2053273314026370 PMC4283466 25537383

