# Supplementary material for: A Redox‐active Cyclometalated Platinum Ring Enables Synthetic Post‐processing of a [2]Rotaxane
Source: Angew Chem Int Ed Engl. 2024 Oct 31;64(3):e202415381. doi: 10.1002/anie.202415381 (PMC11735883; doi:10.1002/anie.202415381)
Supplement: Supplementary file 1 — Supporting Information [file ANIE-64-e202415381-s001.pdf]

Angewandte Chemie
Eine Zeitschrift der Gesellschaft Deutscher Chemiker

Supporting Information

A Redox-active Cyclometalated Platinum Ring Enables Synthetic Post-processing of a [2]Rotaxane

*R. Kandel, M. A. Soto, D. Medina, B. O. Patrick, F. Lelj, M. J. MacLachlan**

Supplementary Information

A Redox-active Cyclometalated Platinum Ring Enables Synthetic Post-processing of a [2]Rotaxane

Raksha Kandel,[a] Miguel A. Soto,[a] Daniel Medina,[a] Brian O. Patrick,[a] Francesco Lelj,[b] and Mark J. MacLachlan*[a,c,d]

[a] Department of Chemistry, University of British Columbia, 2036 Main Mall, Vancouver, BC, V6T 1Z1, Canada

[b] La.M.I. and LaSCAMM INSTM Sezione Basilicata, Dipartimento di Scienze, Università della Basilicata, Via dell'Ateneo Lucano 10, 85100 Potenza, Italy

[c] Stewart Blusson Quantum Matter Institute, University of British Columbia, 2355 East Mall, Vancouver, BC, V6T 1Z4, Canada

[d] WPI Nano Life Science Institute, Kanazawa University, Kanazawa, 920-1192, Japan

mmaclach@chem.ubc.ca

Table of contents

Methods 3
Computational methods 3
Synthesis 4
Compound D 4
Compound E 8
Compound 1 10
Compound G 13
Compound H 17
Compound 2 18
Compound 2·H[BF_4] 20
Compound 1·$H_2[BF_4]_2$ 24
Compound 3·$H_3[BF_4]_3$ 28
Compound 3 32
Compound 3-Pt^{II} 38
Compound 3-Pt^{IV} 45
Compound 3-Pt^{III} 49

Computational data 57
Comparison between computed structures of the rotaxanes 57
3-Pt^{II} 57
3-Pt^{IV} 60
Dimeric $Pt^{III}Cl_2$ and 3-Pt^{III} 63
Variable-temperature (VT) ^{1}H NMR characterization 73
Determination of the shuttling rates 76
Solvent-excluded surface calculations 77
UV-Vis and emission spectra 78
Crystallographic data 80
3[PF_6] 80
3-Pt^{II} 81
3-Pt^{IV} 83
References 86

Methods

All commercially available reagents were purchased from Sigma Aldrich and Oakwood Chemical and used as received unless otherwise stated. All deuterated solvents were purchased from Sigma Aldrich except for deuterated chloroform, which was purchased from Cambridge Isotope Laboratories. Dry acetonitrile was prepared by sparging it under nitrogen (N_2) gas and storing over 4 Å molecular sieves overnight. Flash column chromatography was performed using SiliCycle silica gel (230-400 mesh) as the stationary phase. Unless otherwise noted, all reactions were carried out under air. ^{1}H and ^{13}C{^{1}H} Nuclear Magnetic Resonance (NMR) spectra were recorded on Bruker AV III HD 400 MHz, Bruker Avance 400 MHz, and Bruker Neo 600 MHz spectrometers. Chemical shifts (δ) are reported in parts per million (ppm) and referenced to the residual solvent signal; the coupling constants (*J*) are reported in Hertz (Hz) and the multiplicities are denoted using the following abbreviations: *s* = singlet, *d* = doublet, *t* = triplet, *dd* = doublet of doublets, and *m* = multiplet. Ultraviolet-visible (UV-vis) spectroscopy measurements were conducted on a UV-vis-NIR Cary 5000 spectrophotometer using a 1 cm pathlength quartz cuvette. Photoluminescence spectra were collected on a Photon Technology International (PTI) QuantaMaster 50 fluorimeter fitted with a 75 W Xe arc lamp as the light source. Single crystal X-ray diffraction (SCXRD) data were obtained on a Bruker APEX II using a monochromatic Mo-Kα (λ = 0.71073 Å) or Cu-Kα (λ = 1.5406 Å). Low-resolution mass spectra (LRMS) and high-resolution mass spectra (HRMS) were collected on a Waters ZQ spectrometer equipped with ESCI (Electrospray chemical ionization) ion source and an ESI-TOF (Electrospray ionization time-of-flight) Waters Micromass LCT spectrometer, respectively. The mass spectra for **3-Pt^{III}** was obtained using a matrix assisted laser desorption/ionization (MALDI) equipped with a Bruker Biflex IV time-of-flight (TOF) mass spectrometer; trans-2-[3-(4- tert-butylphenyl)-2-methyl-2-propenylidene]malononitrile (dctb) was used as the matrix compound.

Compounds **A,**[1] **B,**[1] **C,**[2] and **F**[3] were synthesized using previously reported procedures.

Computational methods

The Gaussian suite of programs G016.revC01[4] was used for all geometry calculations. In addition, geometries were computed with the hybrid xc functional PBE1PBE[5] and in some cases with the range separated dispersion corrected PW6B95D3,[6] ωB97xD.[7] The basis set for all atoms was the 6-31g[8] and 6-31g(d).[9] In case of Pt, the fully relativistic energy consistent pseudopotential was used with cc-pXVZPP (x=D, T) in correspondence with the similar multiple zeta basis on non-Pt atoms).[10] The level of theory used is indicated as: (non-Pt atom basis set; Pt basis set, Pt pseudopotential / xc-functional / solvent). All integrals have been computed using the "Ultrafine" integration grid. Solvent environment was considered by the self-consistent reaction field (SCRF) approach for continuum solvent model simulation included in the Gaussian suite of programs.[11]

Synthesis

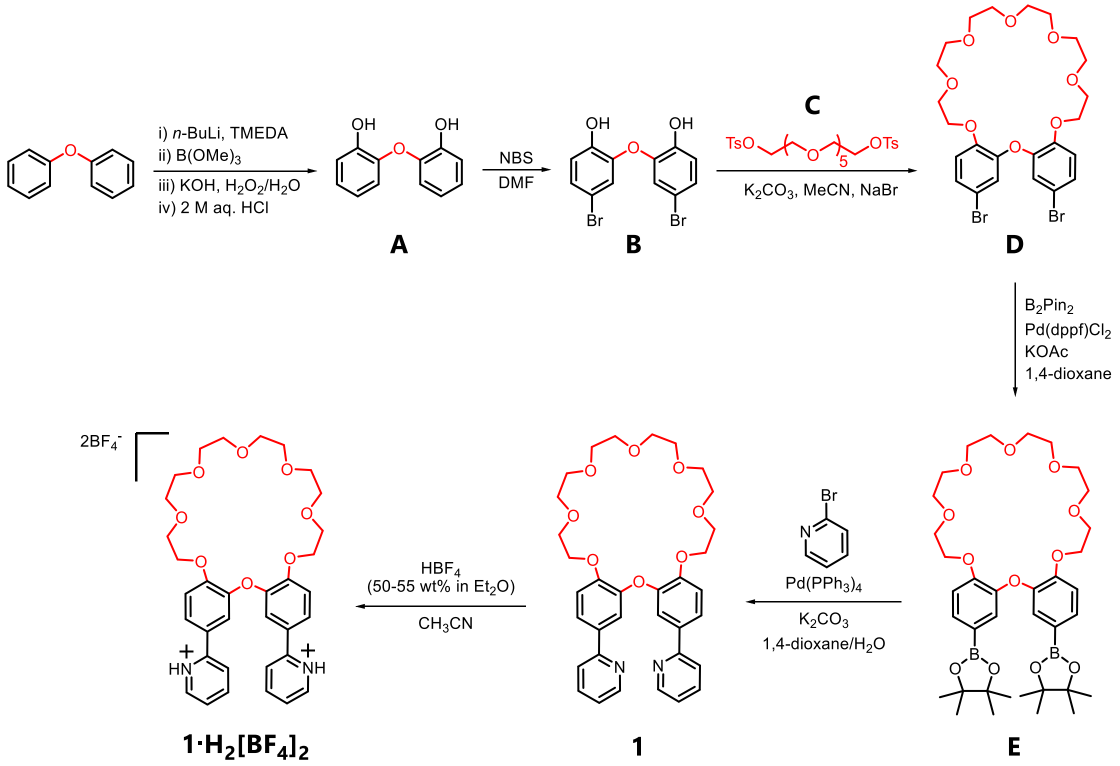

Scheme S1: Synthesis of the macrocyclic ligand **1·H**$_2$[BF$_4$]$_2$.

Compound D

A reaction flask containing K_2CO_3 (3.30 g, 24 mmol) and NaBr (56 mg, 0.55 mmol) was evacuated and backfilled with N_2 three times, followed by the addition of dry CH_3CN (340 mL). A solution of **B** (1.96 g, 5.5 mmol) and **C** (3.87 g, 6.5 mmol) prepared in dry CH_3CN (340 mL) was then added dropwise to the reaction mixture *via* an addition funnel. Once the addition was complete, the reaction was heated at 82 °C for 48 h under the protection of N_2. After letting the system cool down to room temperature, the solvents were removed under vacuum and the white residue was partitioned between water (180 mL) and $CHCl_3$ (120 mL). The organic layer was extracted with $CHCl_3$ (2 × 90 mL), then washed with water (110 mL) and brine (110 mL), dried over anhydrous $MgSO_4$, and concentrated under vacuum. The residual yellow oil was purified by column chromatography on silica gel using dichloromethane (DCM):acetone as the mobile phase (4:1, v/v, R_f = 0.30) to give compound **D** as a clear oil, which solidified into white crystals over time (1.76 g, 2.9 mmol, 53%). **^{1}H NMR (400 MHz, CDCl$_3$)**: δ 7.17 (*dd*, *J* = 8.6 Hz, 1.6 Hz, 2H), 6.96 (*d*, *J* = 1.6 Hz, 2H), 6.90 (*d*, *J* = 8.6, 2H), 4.14 (*t*, *J* = 4.5 Hz 4H), 3.77 (*t*, *J* = 4.5 Hz, 4H), 3.62 (*m*, *J* = 3.8 Hz, 16 H). **^{13}C{^{1}H} NMR (101 MHz, CDCl$_3$)**: δ 149.0, 147.1, 127.2, 122.4, 117.0, 113.3, 71.1, 70.96, 70.94, 70.8, 70.0, 69.8. **ESI-HRMS:** [**D** + K$^+$] *m/z* = 644.9922 (experimental), *m/z* = 644.9919 (calculated), relative error = -0.5 ppm.

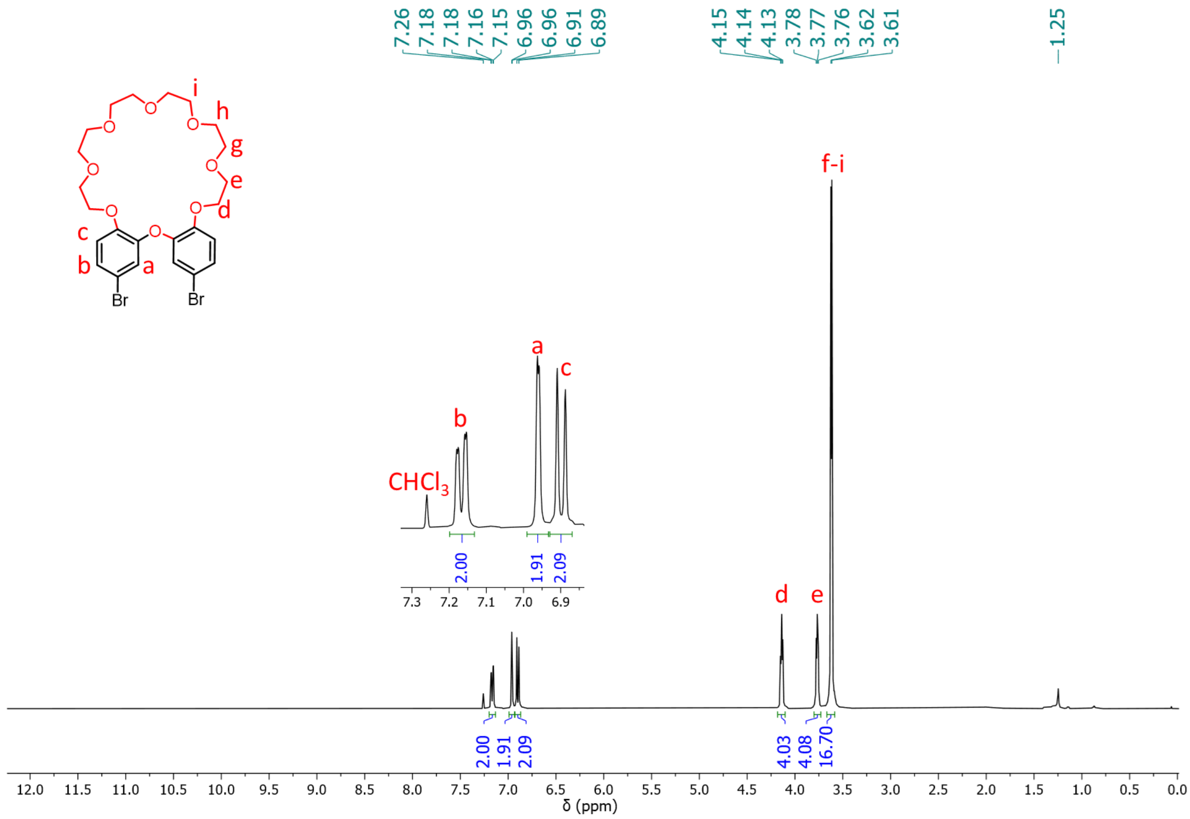

Figure S1. ^{1}H NMR spectrum (400 MHz, $CDCl_3$) of compound **D**.

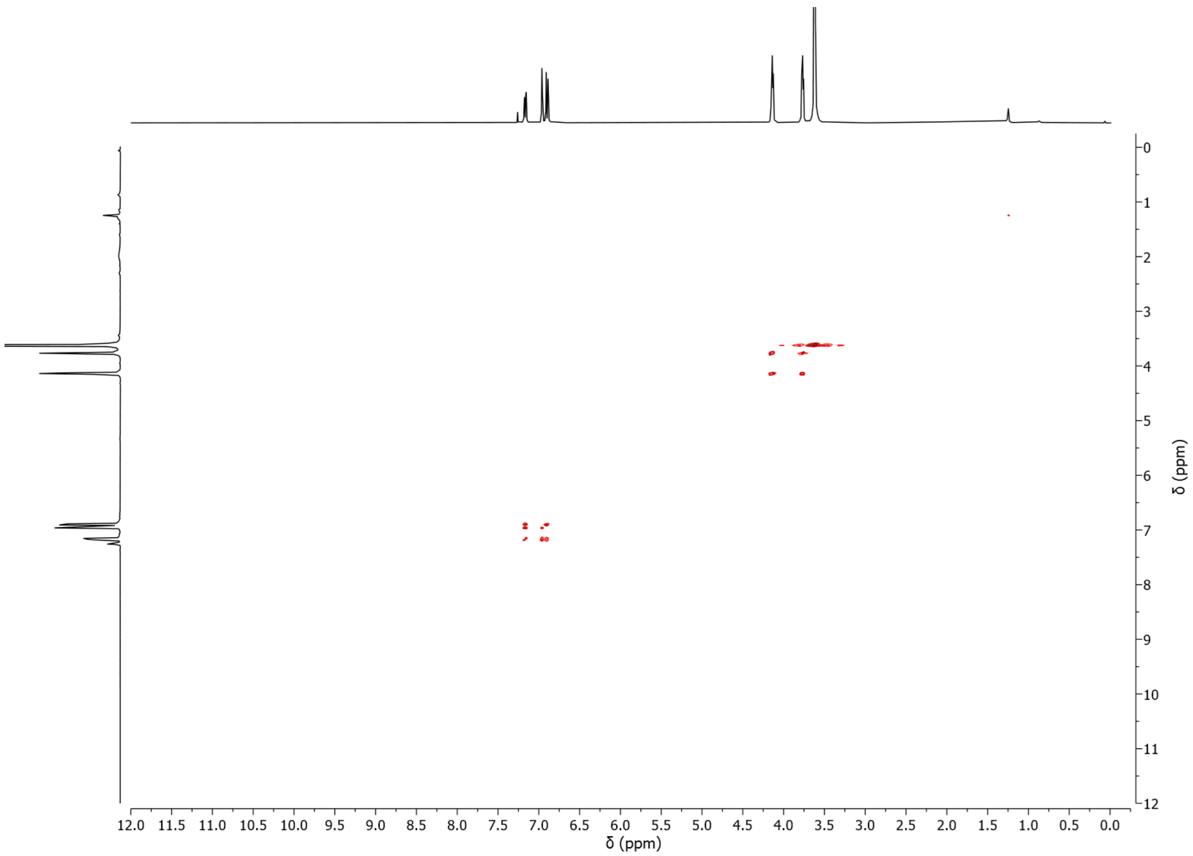

Figure S2. ^{1}H-^{1}H COSY NMR spectrum (400 MHz, $CDCl_3$) of compound **D**.

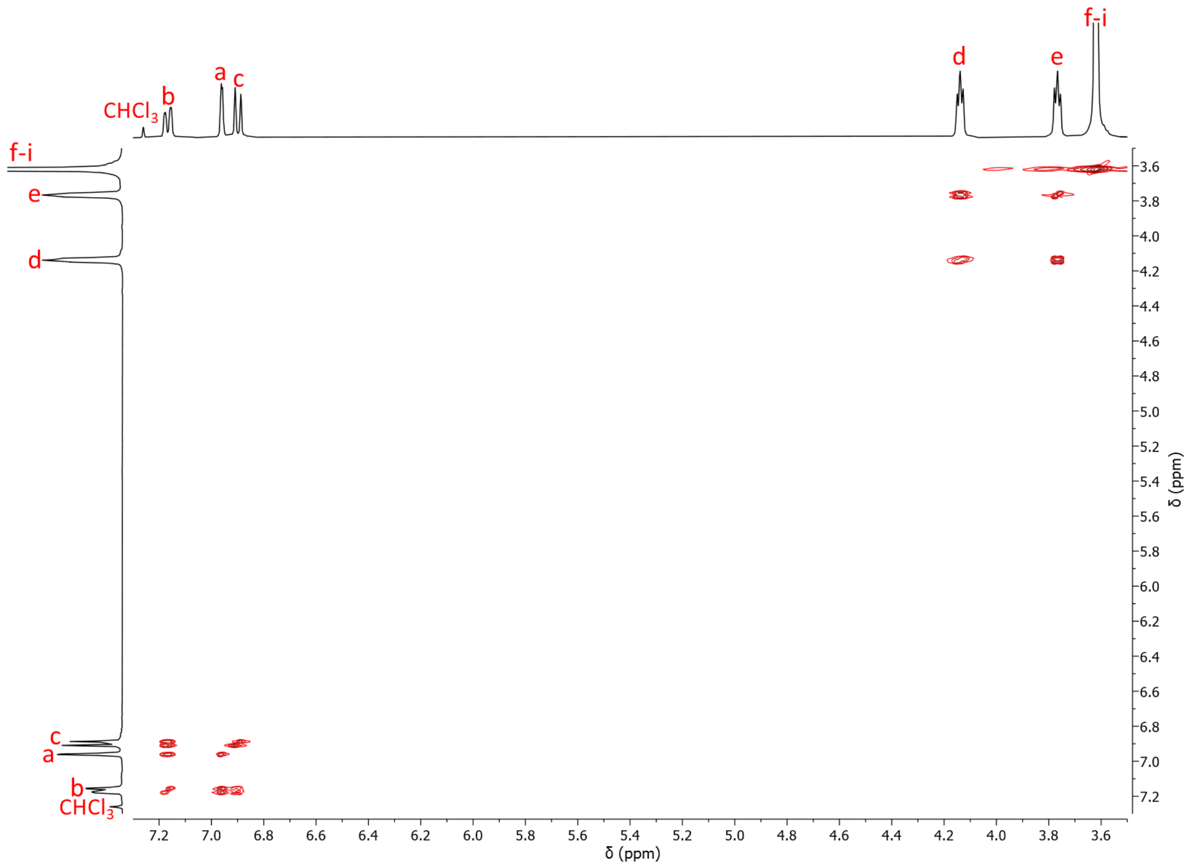

Figure S3. Partial ^{1}H-^{1}H COSY NMR spectrum (400 MHz, $CDCl_3$) of compound **D**.

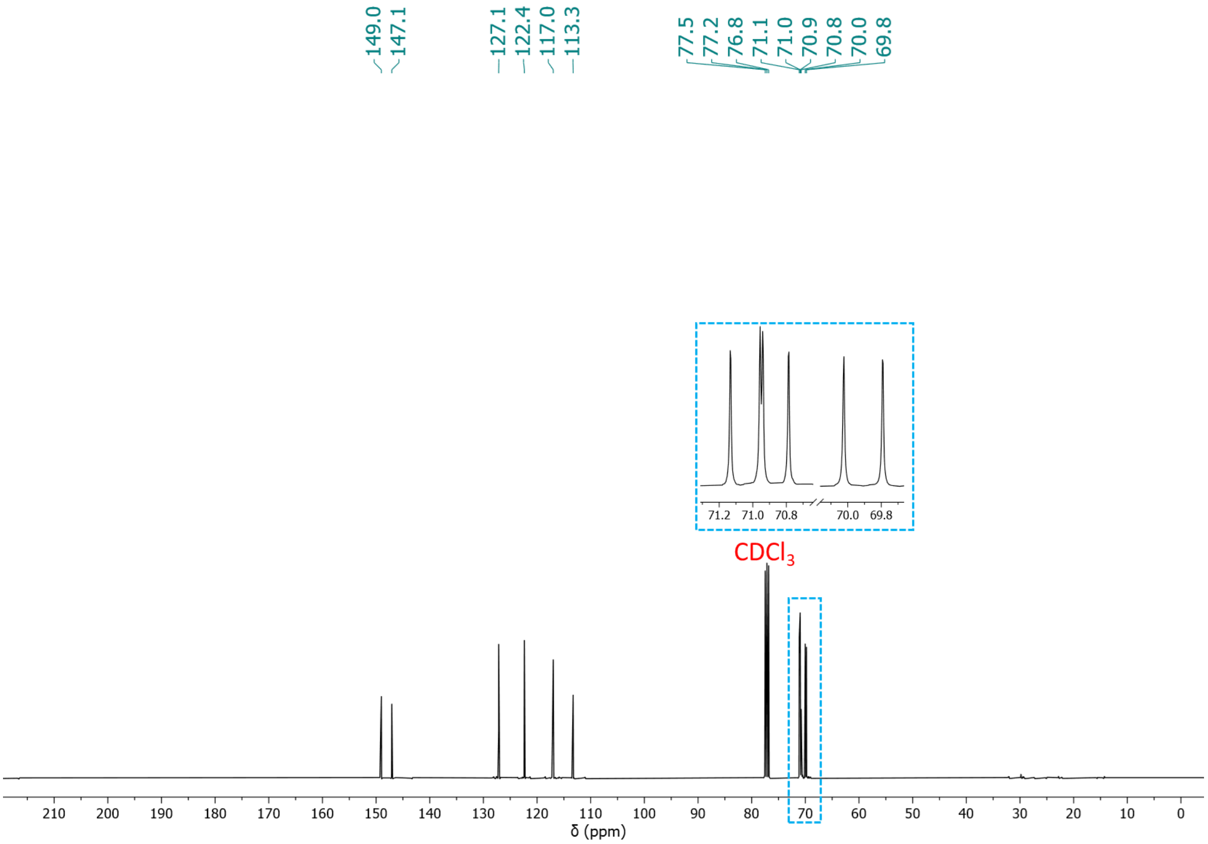

Figure S4. $^{13}C\{^{1}H\}$ NMR spectrum (101 MHz, $CDCl_3$) of compound **D**.

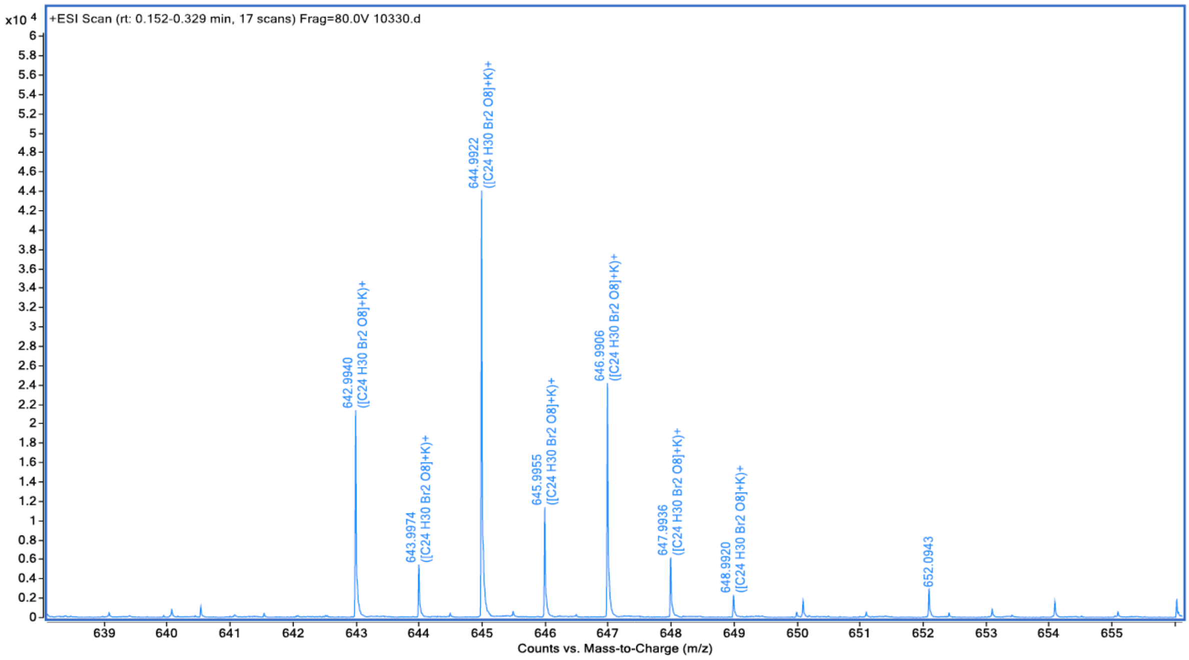

Figure S5. ESI-HRMS of compound **D**.

Compound E

The synthesis for compound **E** was adapted from a previously reported procedure.[12] A flask containing compound **D** (803 mg, 1.3 mmol), KOAc (390 mg, 4.0 mmol), bis(pinacolato)diboron (B_2Pin_2) (807 mg, 3.2 mmol), and Pd(dppf)Cl_2 (108 mg, 10 mol%) was evacuated and backfilled with N_2 three times, followed by the addition of deaired 1,4-dioxane (8 mL). This mixture was stirred at 80 °C under N_2 for 24 h. After cooling the system to room temperature, the solvent was removed under vacuum to obtain a reddish-brown oil, which was suspended in water (80 mL) and extracted with benzene (3 × 50 mL). The combined organic layers were dried over anhydrous $MgSO_4$ and concentrated by rotary evaporation to yield a reddish-brown oil, identified as compound **F**. This was used without further purification. **^{1}H NMR (400 MHz, $CDCl_3$)**: δ 7.50 (*dd*, *J* = 8.0 Hz, 1.4 Hz, 2H), 7.27 (*d*, *J* = 1.4 Hz, 2H), 6.98 (*d*, *J* = 8.0 Hz, 2H), 4.20 – 4.17 (*m*, 4H), 3.78 – 3.76 (*m*, 4H), 3.63 – 3.62 (*m*, 16H), 1.29 (*s*, 24H). **ESI-HRMS:** [**E** + K^+] *m/z* = 739.3457 (experimental), *m/z* = 739.3433 (calculated), relative error = -3.2 ppm.

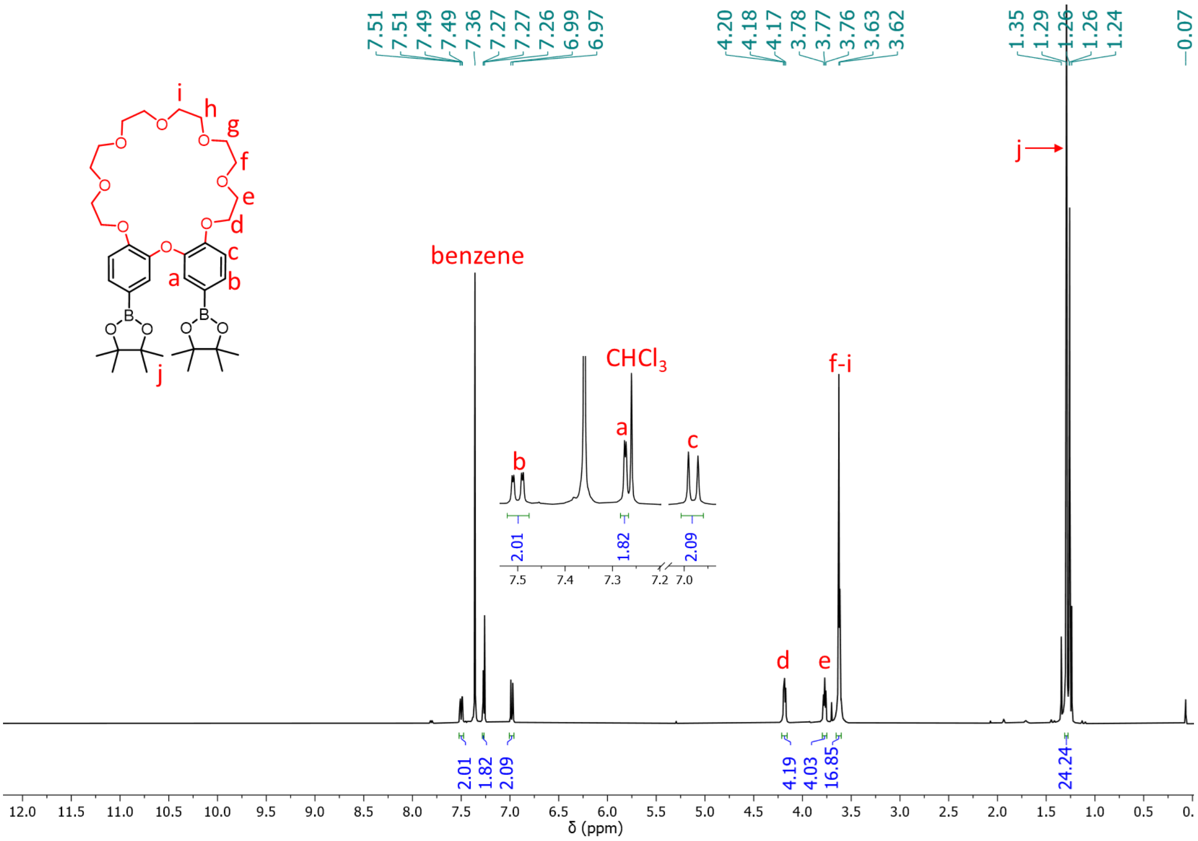

Figure S6. ^1H NMR spectrum (400 MHz, $CDCl_3$) of crude compound **E**.

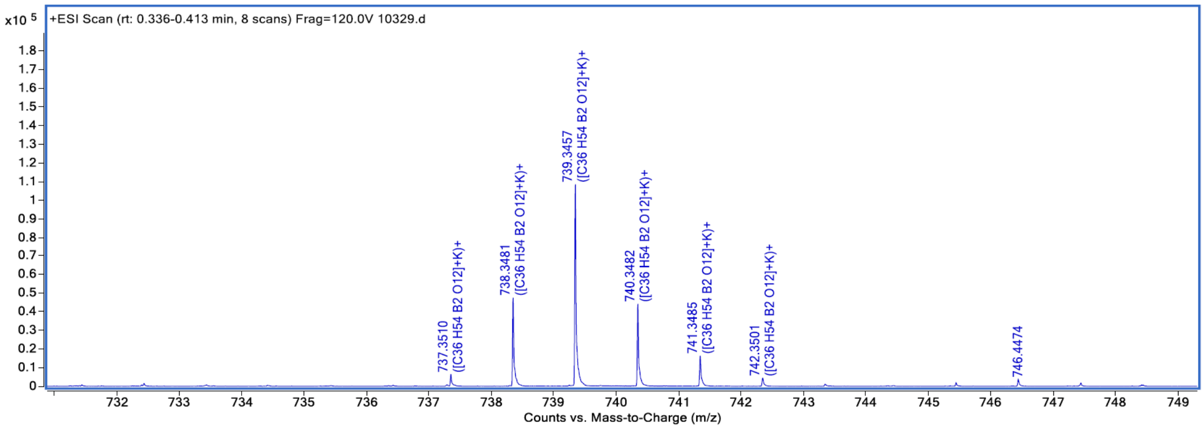

Figure S7. ESI-HRMS of compound **E**.

Compound 1

Compound **E** (930 mg, 1.3 mmol), K_2CO_3 (1.8 g, 13 mmol), and $Pd(PPh_3)_4$ (150 mg, 0.13 mmol) were combined in a flask. This system was evacuated and backfilled with N_2 three times. Next, degassed 2-bromopyridine (1.0 mL, 10 mmol) was added to the reaction flask, followed by the addition of a N_2-sparged mixture of 1,4-dioxane (9 mL) and water (3 mL). The resulting suspension was then stirred at 80 °C for 24 h to afford a dark brown mixture. The solvents were removed under vacuum and the resulting oil was partitioned in water (120 mL) and DCM (120 mL). After further extractions with DCM (2 × 80 mL), the combined organic fractions were washed with brine (100 mL), dried over anhydrous $MgSO_4$, and concentrated in *vacuo* to obtain a dark brown oil. Column purification in silica gel using DCM:acetone (3:2, v/v, R_f = 0.18) as the mobile phase gave the product (**1**) as a white solid (410 mg, 0.7 mmol, 52 %). **^{1}H NMR (400 MHz, $CDCl_3$)**: δ 8.60 – 8.58 (*m*, 2H), 7.77 (*dd*, *J* = 8.5, 2.2 Hz, 2H), 7.68 – 7.64 (*td*, *J* = 7.8, 1.8 Hz, 2H), 7.57 – 7.55 (*m*, 4H), 7.16 – 7.11 (*m*, 4H), 4.26 – 4.23 (*m*, 4H), 3.82 – 3.80 (*m*, 4H), 3.68 – 3.62 (*m*, 16H). **^{13}C{^{1}H} NMR (101 MHz, $CDCl_3$)**: δ 156.6, 150.7, 149.5, 146.9, 136.9, 133.1, 122.7, 121.8, 120.2, 117.8, 115.5, 71.2, 71.0, 70.983, 70.976, 69.88, 69.87. **ESI-HRMS:** [**1** + K^+] *m/z* = 641.2261 (experimental), *m/z* = 641.2260 (calculated), relative error = -0.2 ppm.

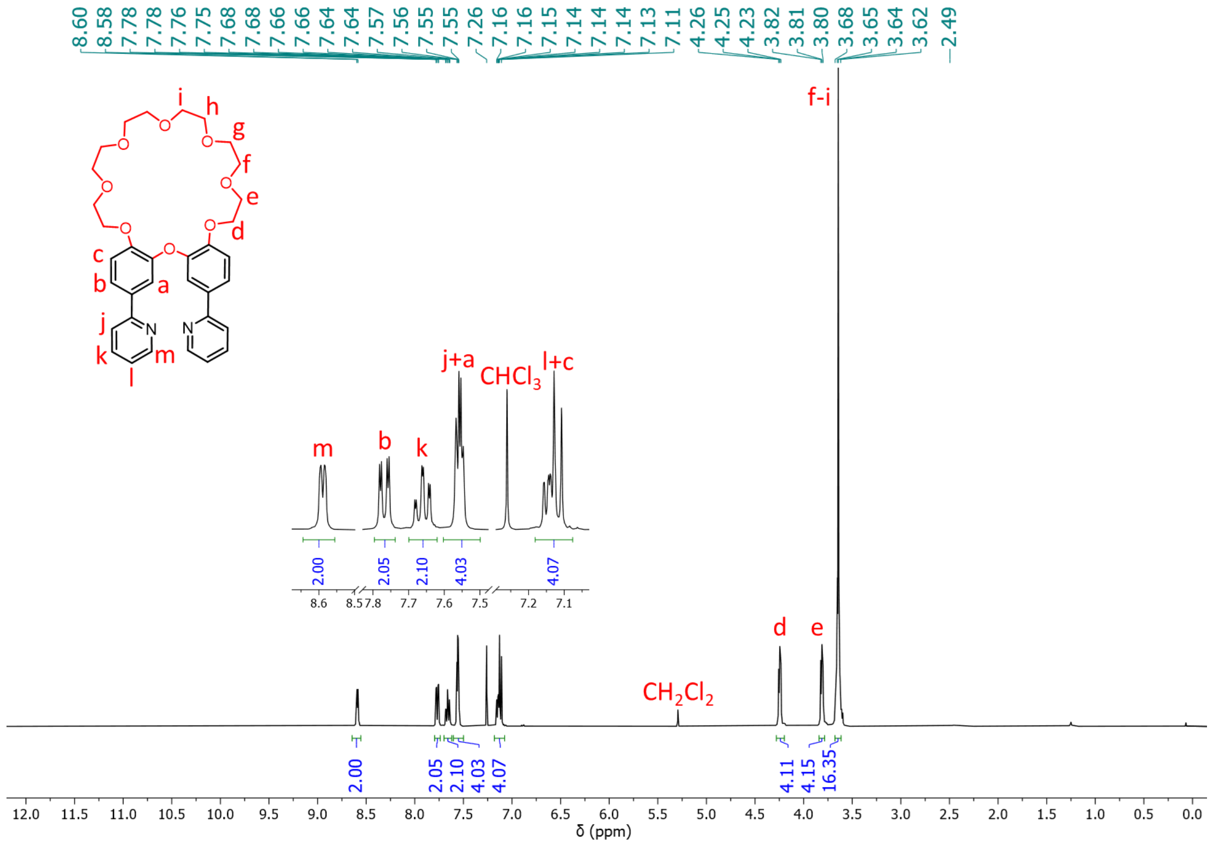

Figure S8. ^{1}H NMR spectrum (400 MHz, $CDCl_3$) of compound **1**.

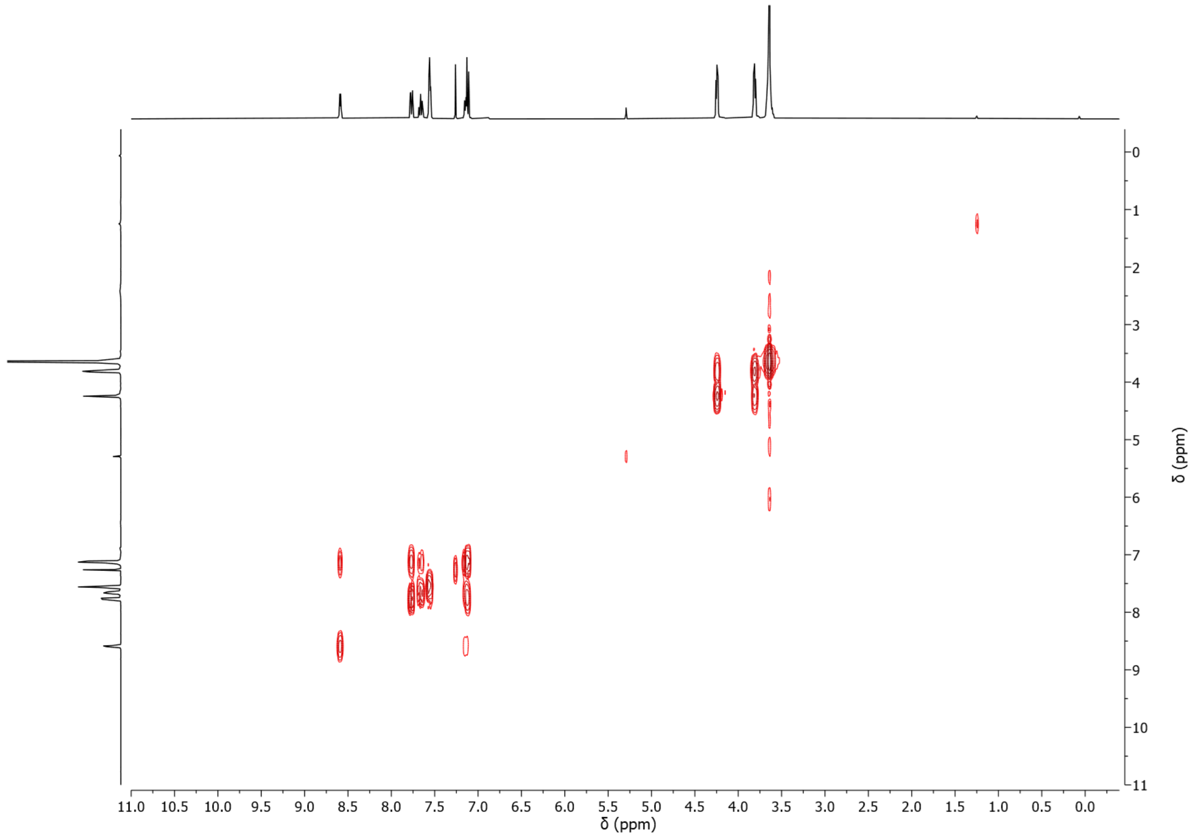

Figure S9. ^{1}H-^{1}H COSY NMR spectrum (400 MHz, $CDCl_3$) of compound **1**.

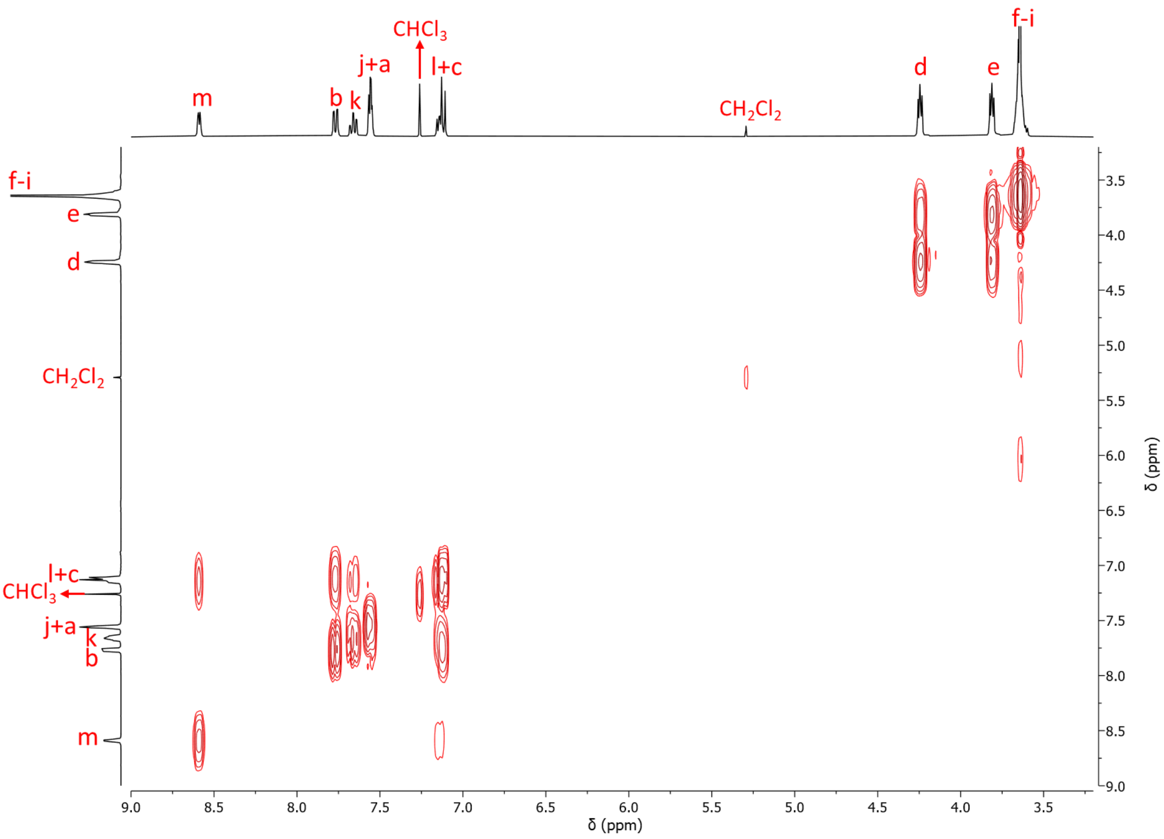

Figure S10. Partial ^{1}H-^{1}H COSY NMR spectrum (400 MHz, $CDCl_3$) of compound **1**.

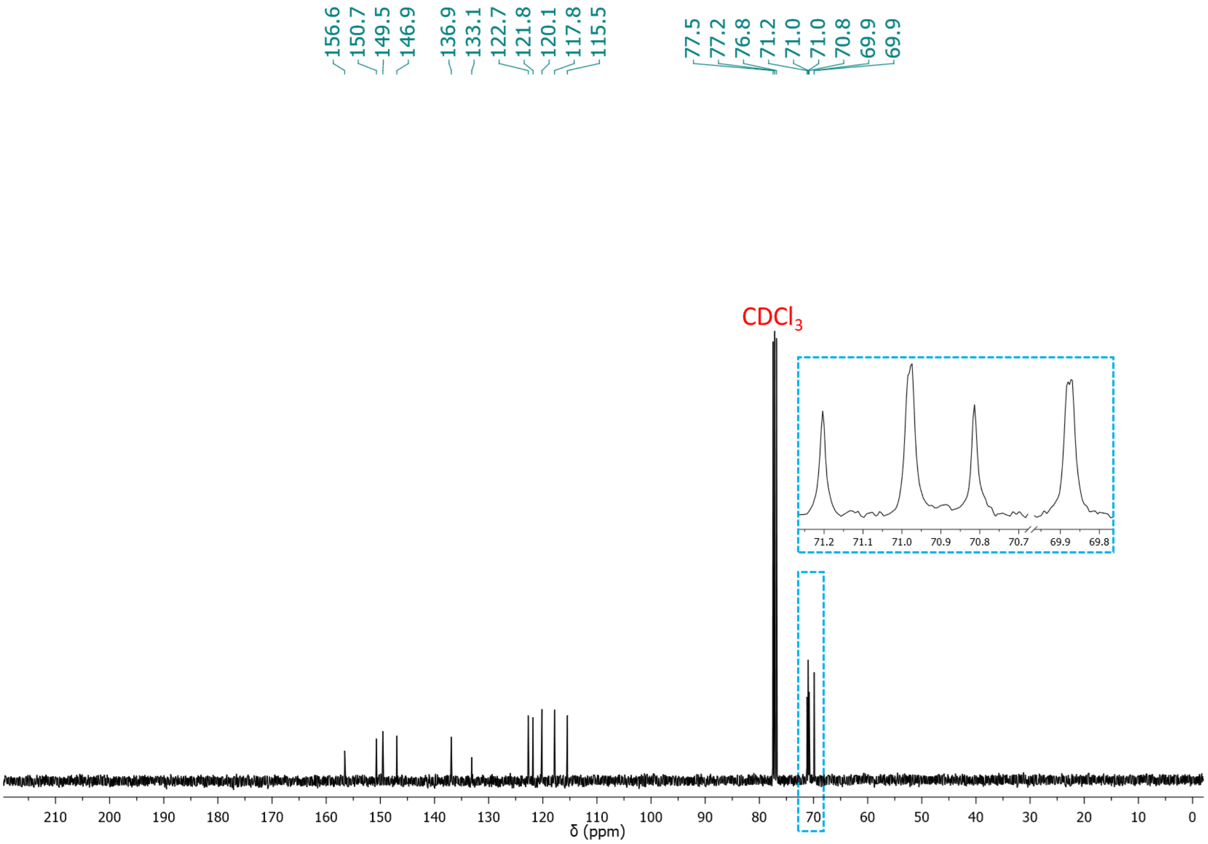

Figure S11. ^{13}C{^{1}H} NMR spectrum (101 MHz, $CDCl_3$) of compound **1**.

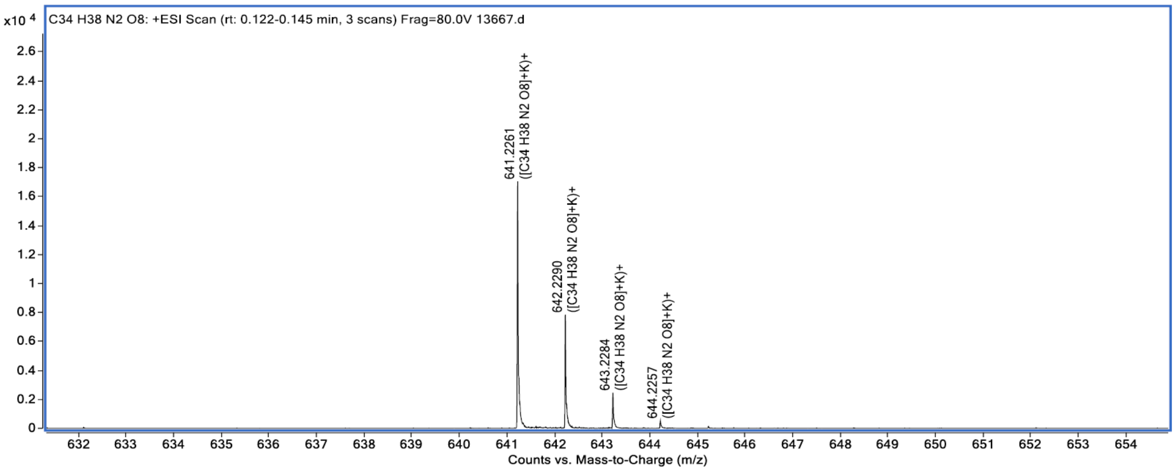

Figure S12. ESI-HRMS of compound **1**.

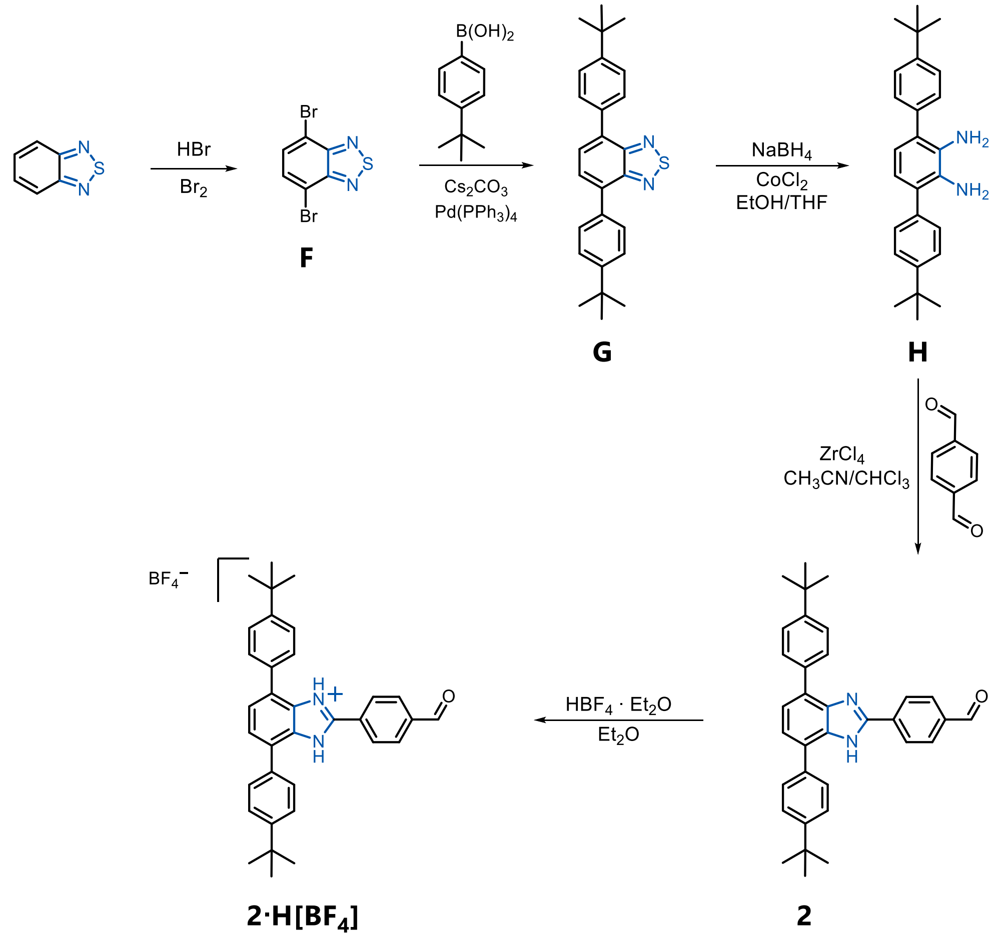

Scheme S2: Synthesis of the T-shaped axle precursor **2·H[BF$_4$]**.

Compound G

Compound **G** was synthesized *via* a modified literature procedure.[13] Compounds 4,7-dibromobenzo[c]-1,2,5-thiadiazole (1.0 g, 3.4 mmol), 4-tert-butylphenylboronic acid (1.8 g, 10.2

mmol), $CsCO_3$ (3.1 g, 9.5 mmol), and $Pd(PPh_3)_4$ (200 mg, 0.17 mmol, 5 mol%) were combined in a Schlenk flask. This vessel was evacuated and backfilled with N_2 three times, followed by the addition of a degassed mixture of dimethylformamide (30 mL) and toluene (30 mL). The resulting mixture was stirred at 85 °C under N_2 for 48 h. Once it cooled to room temperature, the suspension was filtered through celite, and the brownish light green filtrate was dried under vacuum. Finally, the solid residue was purified using column chromatography on silica gel, with DCM:hexanes (3:7, v/v, R_f = 0.41) as the eluent to afford neon green solids (**G**) (1.2 g, 3.1 mmol, 91%). **^{1}H NMR (400 MHz, $CDCl_3$)**: δ 7.91 (*d*, 4H, *J* = 8.4 Hz), 7.78 (*s*, 2H), 7.59 (*d*, 4H, *J* = 8.4 Hz), 1.41 (*s*, 18H). **$^{13}C\{^1H\}$ NMR (101 MHz, $CDCl_3$)**: δ 154.3, 151.5, 134.7, 133.1, 129.0, 128.0, 125.8, 34.8, 32.0.

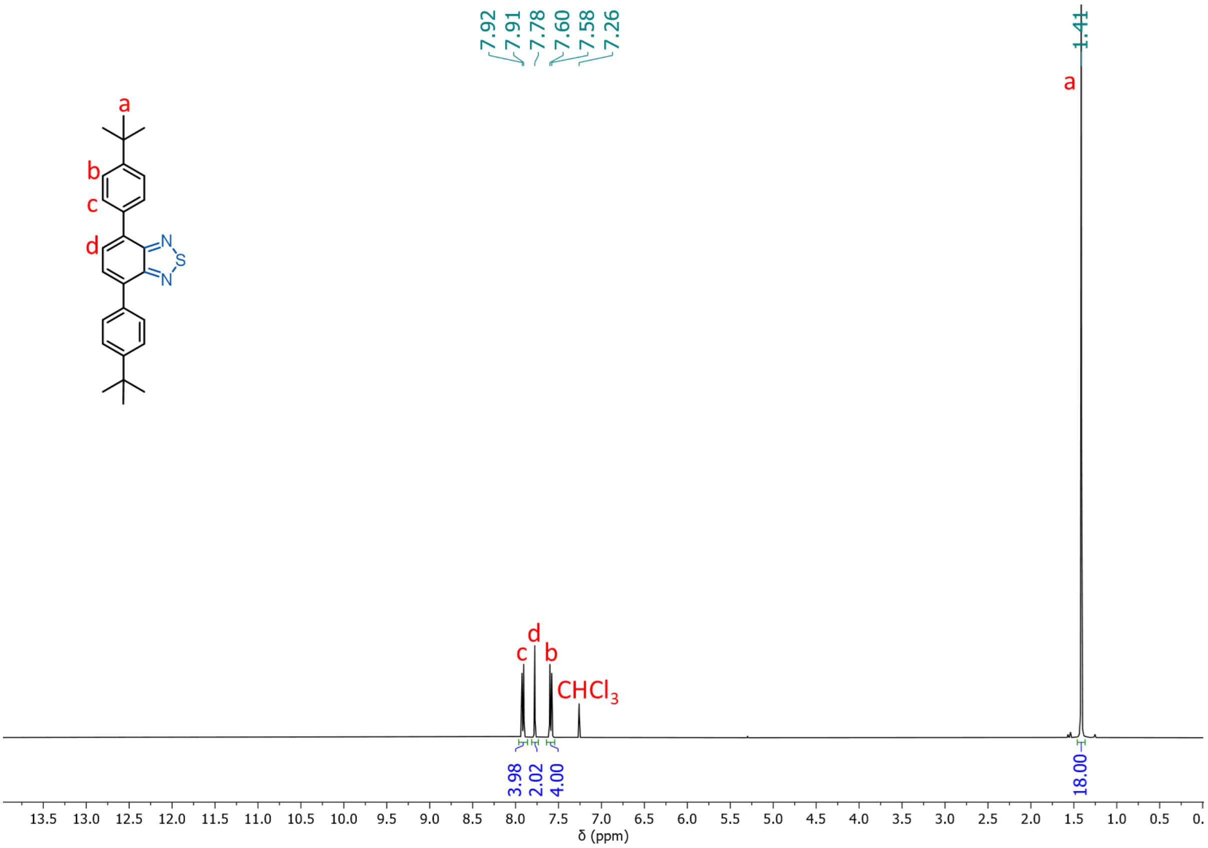

Figure S13. ^{1}H NMR spectrum (400 MHz, $CDCl_3$) of compound **G**.

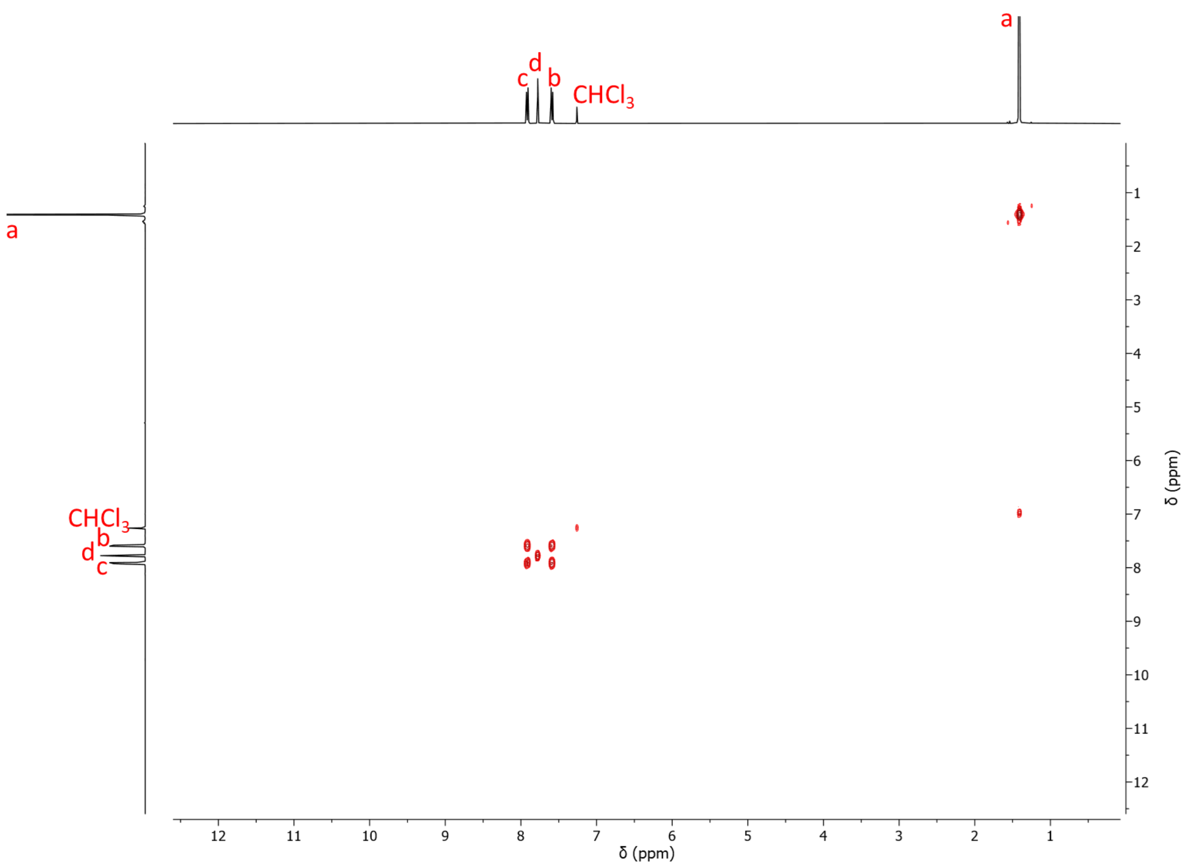

Figure S14. ^{1}H-^{1}H COSY NMR spectrum (400 MHz, $CDCl_3$) of compound **G**.

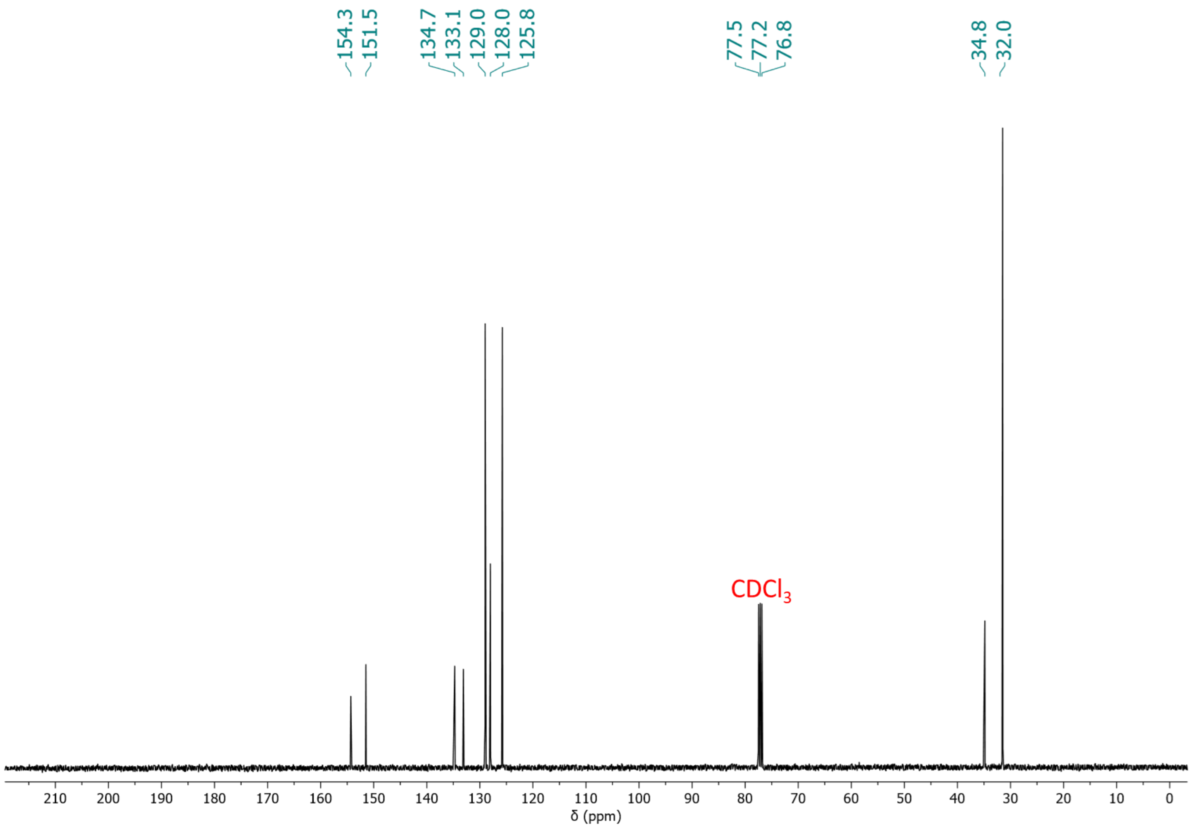

Figure S15. $^{13}C\{^{1}H\}$ NMR spectrum (101 MHz, $CDCl_3$) of compound **G**.

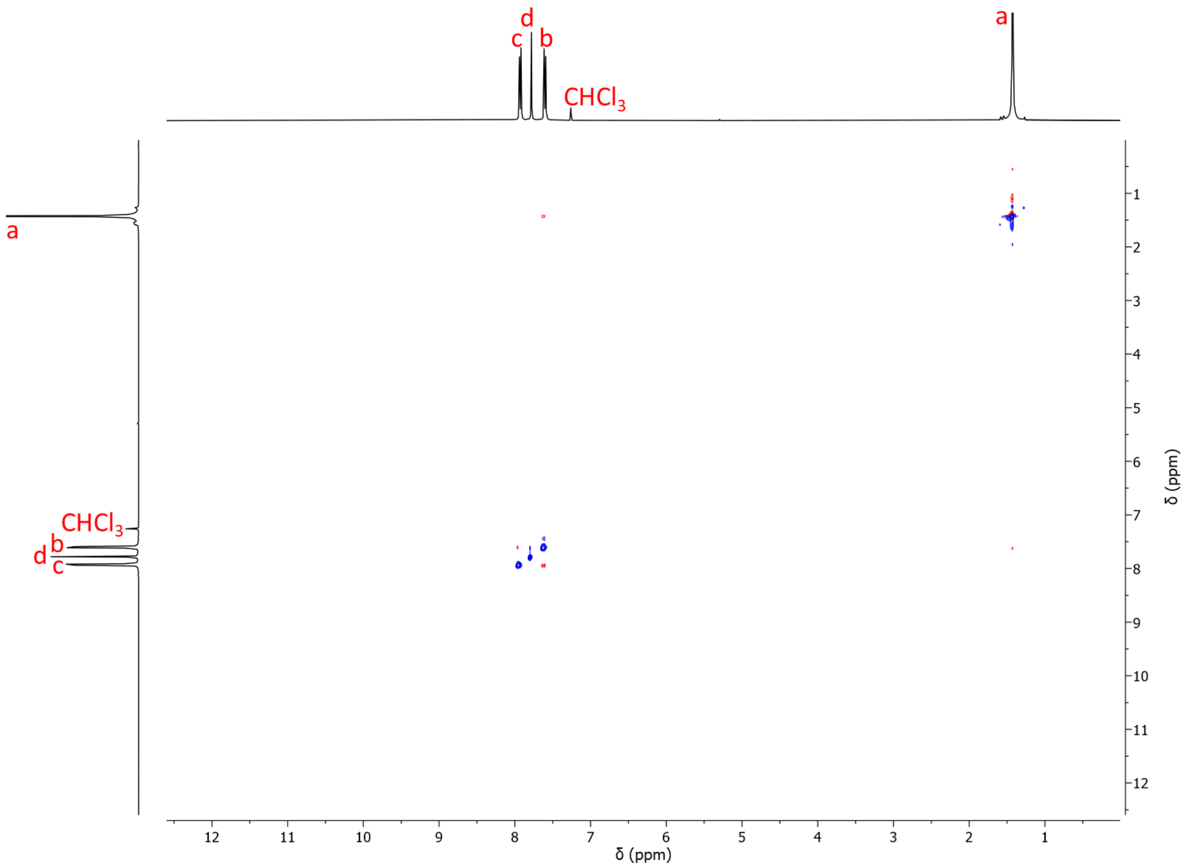

Figure S16. ^{1}H-^{1}H NOESY spectrum (400 MHz, CD_2Cl_2) of compound **G.**

Compound H

Compound **H** was synthesized with slight modifications of a previously reported procedure.[14] A flask containing compound **G** (260 mg, 0.65 mmol) was evacuated and backfilled with N_2 three times. The solid was then dissolved in a degassed mixture of EtOH (98 mL) and tetrahydrofuran (33 mL), followed by the addition, in portions, of $NaBH_4$ (295 mg, 7.8 mmol) and anhydrous $CoCl_2$ (34 mg, 0.65 mmol). The reaction was heated to reflux for 4 h under N_2. A clear solution with black precipitate was observed; this was filtered through celite, and the clear filtrate was then dried by rotary evaporation to afford a white solid. This was partitioned in brine (65 mL) and diethyl ether (65 mL). After further extractions with diethyl ether (2 × 60 mL), the organic layers were combined and dried over anhydrous $MgSO_4$ and concentrated in *vacuo* to afford **H** as a fluffy white solid (228 mg, 0.61 mmol, 95%). The solid was stored under N_2 and at -12°C until its next use. The ^{1}H NMR spectrum matches well with the previously reported. **^{1}H NMR (400 MHz, DMSO-d$_6$)**: δ 7.49 (*d*, 4H, *J* = 8.3 Hz), 7.43 (*d*, 4H, *J* = 8.3 Hz), 6.79 (*s*, 2H), 3.63 (*s*, 4H), 1.38 (*s*, 18H).

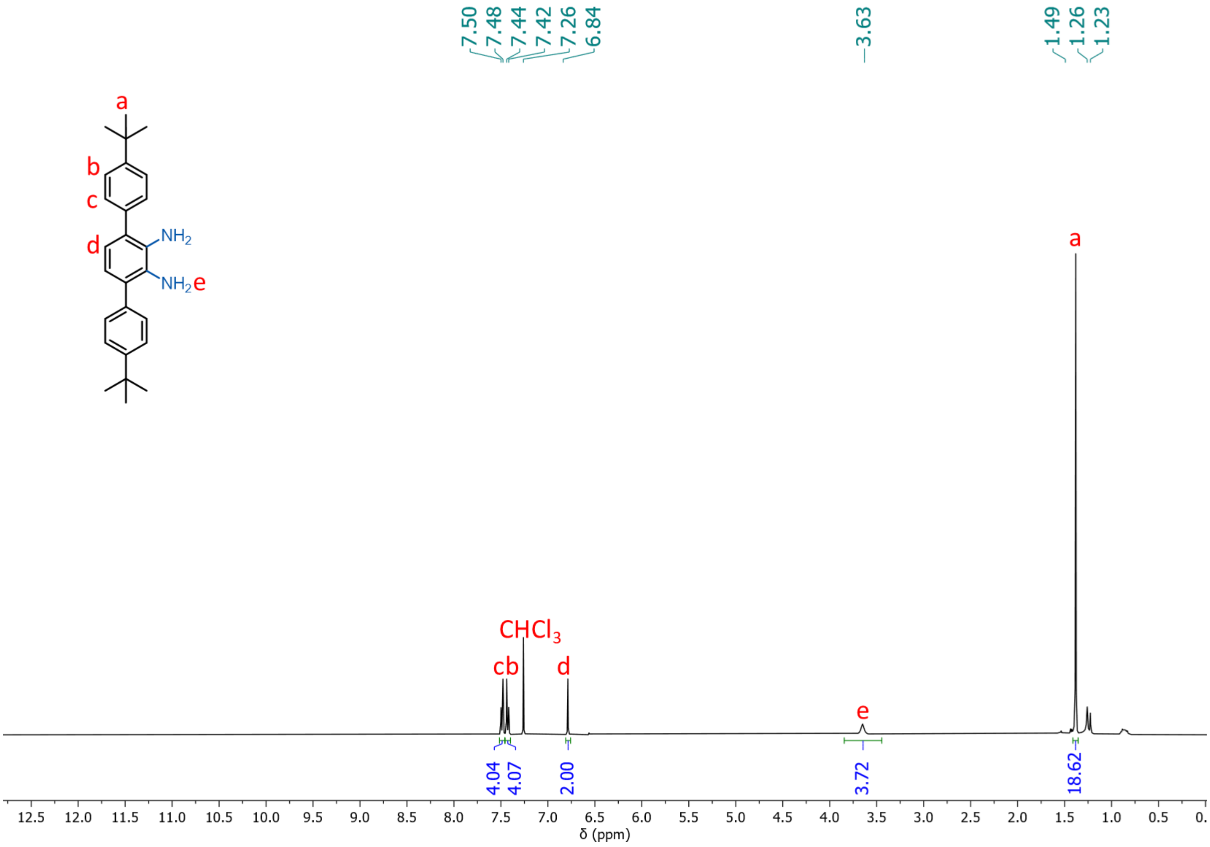

Figure S17. ^{1}H NMR spectrum (400 MHz, $CDCl_3$) of compound **H**.

Compound 2

Compound **2** was synthesized based on a previously reported procedure with some modifications.[14] Compound **H** (179 mg, 0.47 mmol), $ZrCl_4$ (11 mg, 0.047 mmol), and terephthaldehyde (253 mg, 1.90 mmol) were combined in a flask (covered with aluminum foil) and dissolved in acetonitrile (90 mL) and chloroform (18 mL). The reaction was stirred in the dark at room temperature for 1.5 h. Removal of solvents under vacuum produced light yellow solids, which were purified by column chromatography on silica gel using a mixture of ethyl acetate:hexanes as the eluent (1:3, v/v, R_f = 0.41). Compound **2** was isolated as a yellow solid (173 mg, 0.36 mmol, 81%). Spectral data matched previous reports.[14] **^{1}H NMR (400 MHz, $CDCl_3$)**: δ 10.05 (*s*, 1H), 9.70 (*s*, 1H), 8.25 (*d*, *J* = 8.3 Hz, 2H), 8.11 (*d*, *J* = 8.4 Hz, 2H), 7.98 (*d*, *J* = 8.3 Hz, 2H), 7.65 – 7.55 (*m*, 7H), 7.41 (*d*, *J* = 7.7 Hz, 1H), 1.42 (*s*, 9H), 1.41 (*s*, 9H). **^{13}C{^{1}H} NMR (101 MHz, $CDCl_3$)**: δ 191.7, 151.1, 150.5, 149.9, 142.4, 137.0, 135.6, 135.34, 135.26, 133.4, 131.9, 130.3, 129.0, 127.7, 127.3, 126.6, 125.6, 124.7, 124.0, 122.6, 34.8, 31.5.

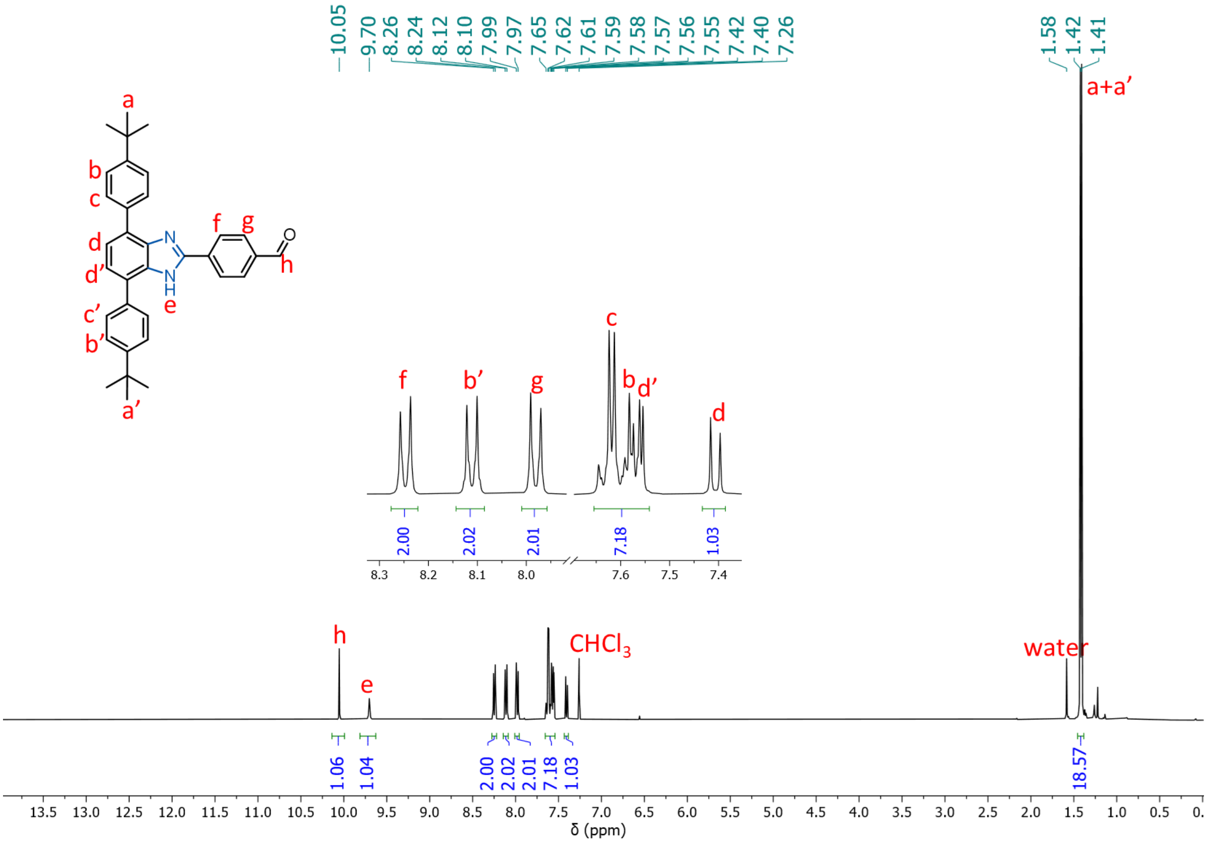

Figure S18. ^{1}H NMR spectrum (400 MHz, $CDCl_3$) of compound **2**.

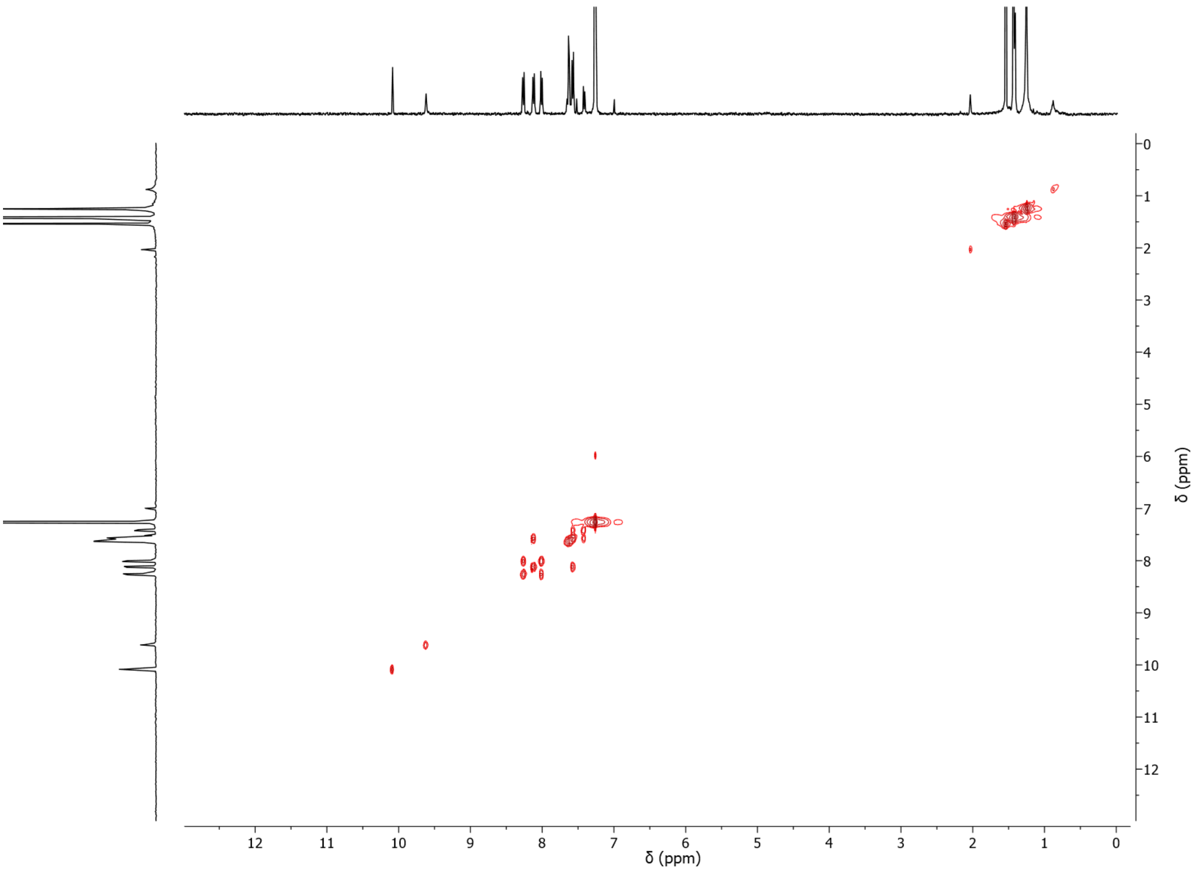

Figure S19. ^{1}H-^{1}H COSY NMR spectrum (400 MHz, $CDCl_3$) of compound **2**.

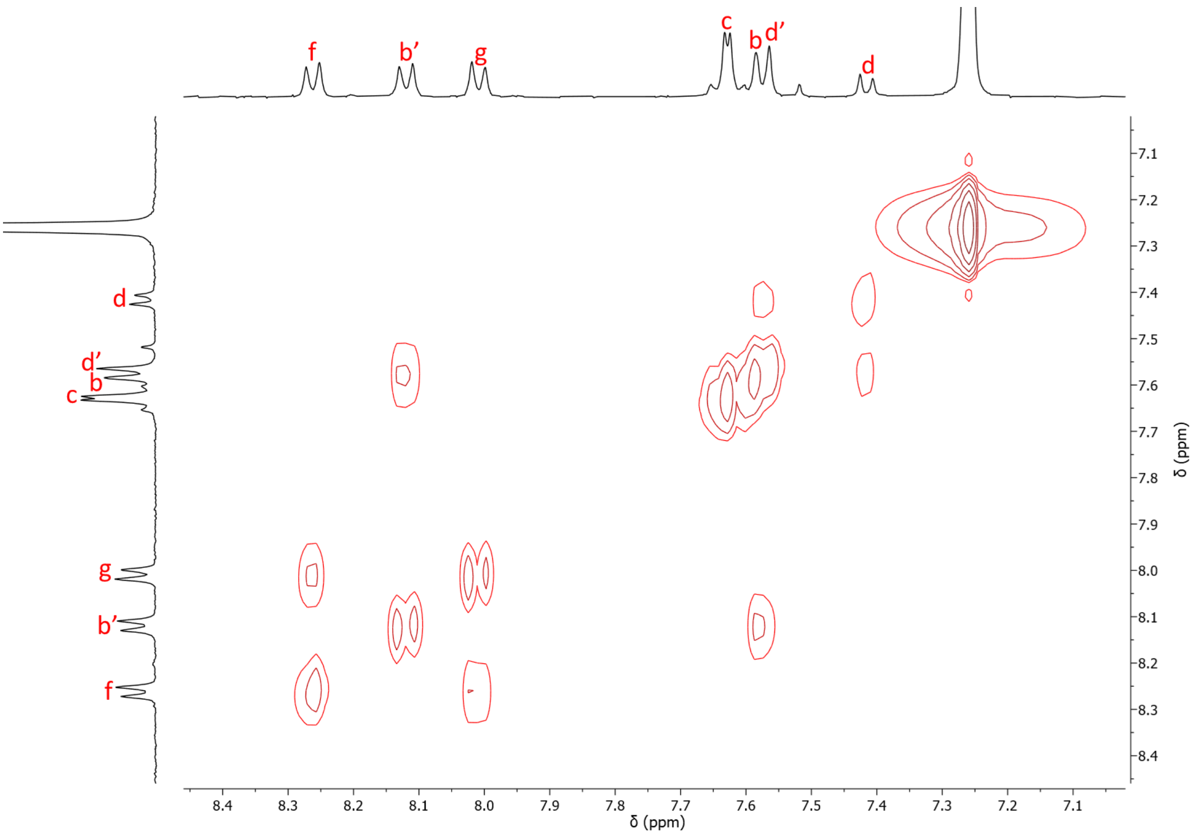

Figure S20. Partial ^{1}H-^{1}H COSY NMR spectrum (400 MHz, $CDCl_3$) of compound **2**.

Compound 2·H[BF4]

Compound **2·H[BF4]** was synthesized based on a previously reported procedure.[14] Compound **2** (120 mg, 0.25 mmol) was dissolved in diethyl ether (48 mL) in a polypropylene beaker, followed by the dropwise addition of HBF_4 (50-55 wt.% in Et_2O, 300 µL, 1.16 mmol) using a plastic syringe. The reaction mixture was covered to prevent evaporation and stirred for *ca.* 15 minutes after the addition was complete. Gravity filtration yielded the product (**2·H[BF4]**) as a light-yellow residue, which was washed with diethyl ether (3 × 5 mL) and air-dried (137 mg, 0.24 mmol, 97%). **^{1}H NMR (400 MHz, $CDCl_3$)**: δ 9.97 (*s*, 1H), 8.24 (*d*, 2H, *J* = 5.6 Hz), 8.02 (*d*, 2H, *J* = 5.6 Hz), 7.72 (*s*, 2H), 7.70 – 7.68 (*m*, 5H), 7.64 – 7.62 (*m*, 5H), 1.42 (*s*, 18H). **^{13}C{^{1}H} NMR (101 MHz, $CDCl_3$)**: δ 191.2, 153.0, 150.1, 140.5, 132.4, 130.8, 130.2, 128.5, 128.4, 127.9, 127.2, 35.2, 32.0. **ESI-HRMS:** [**2** + H$^+$] *m/z* = 487.2745 (experimental), *m/z* = 487.2744 (calculated), relative error = 0.2 ppm.

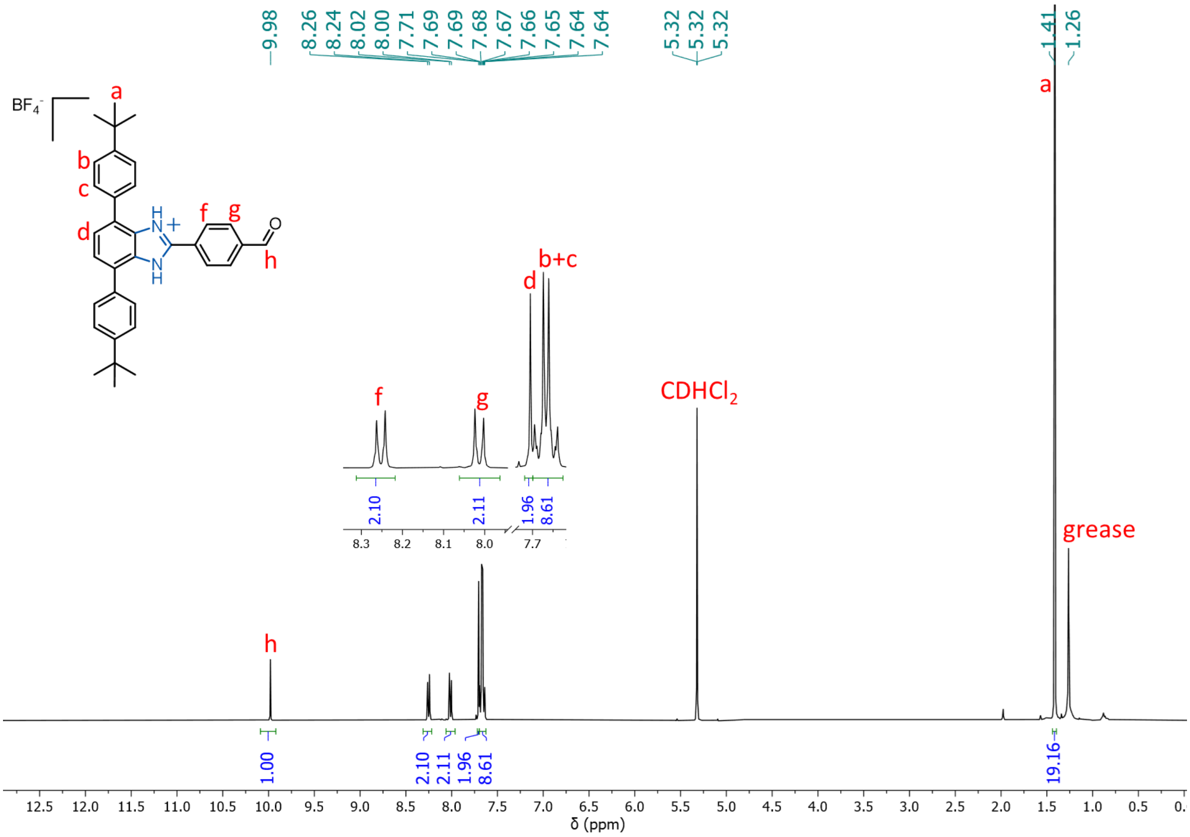

Figure S21. ^{1}H NMR spectrum (400 MHz, CD_2Cl_2) of compound **2·H[BF4]**.

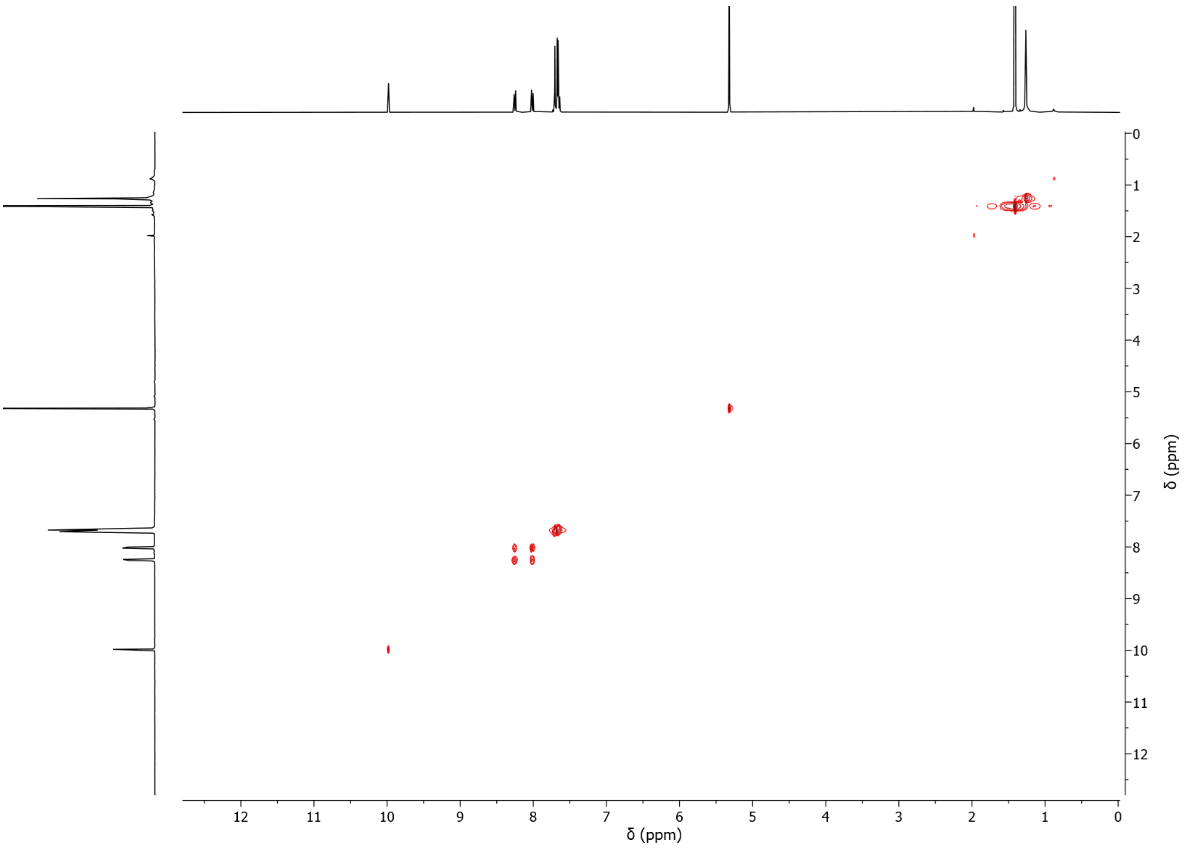

Figure S22. ^{1}H-^{1}H COSY NMR spectrum (400 MHz, CD_2Cl_2) of compound **2·H[BF$_4$]**.

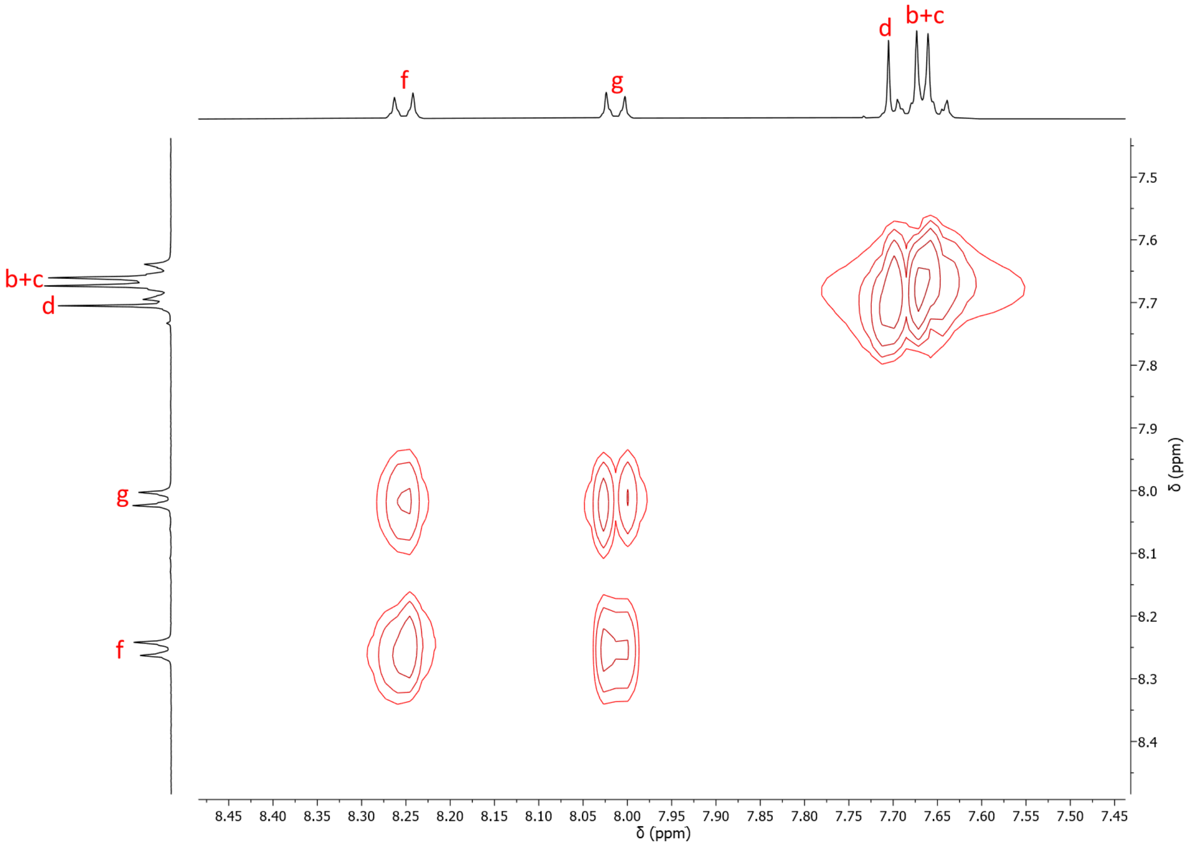

Figure S23. Partial ^{1}H-^{1}H COSY NMR spectrum (400 MHz, CD_2Cl_2) of compound **2·H[BF$_4$]**.

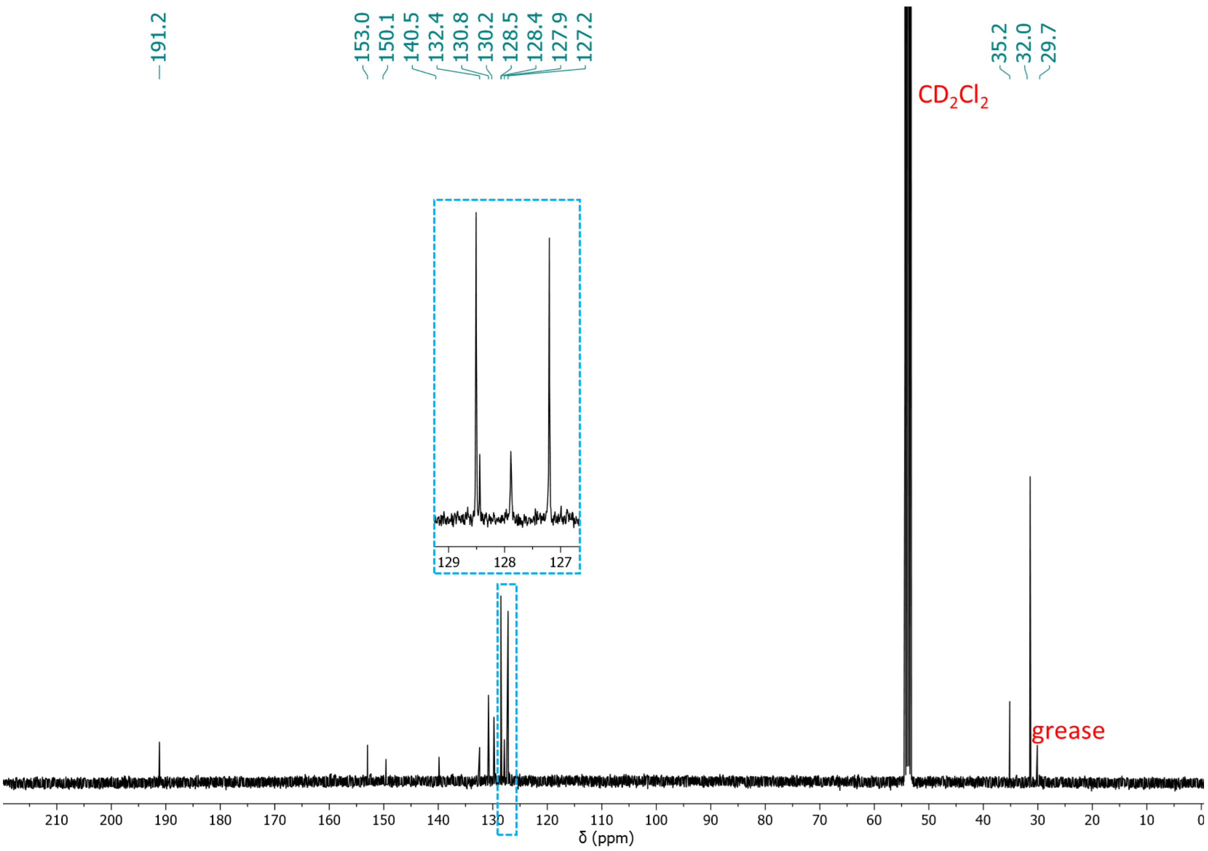

Figure S24. $^{13}C\{^{1}H\}$ NMR spectrum (101 MHz, CD_2Cl_2) of compound **2·H[BF$_4$]**.

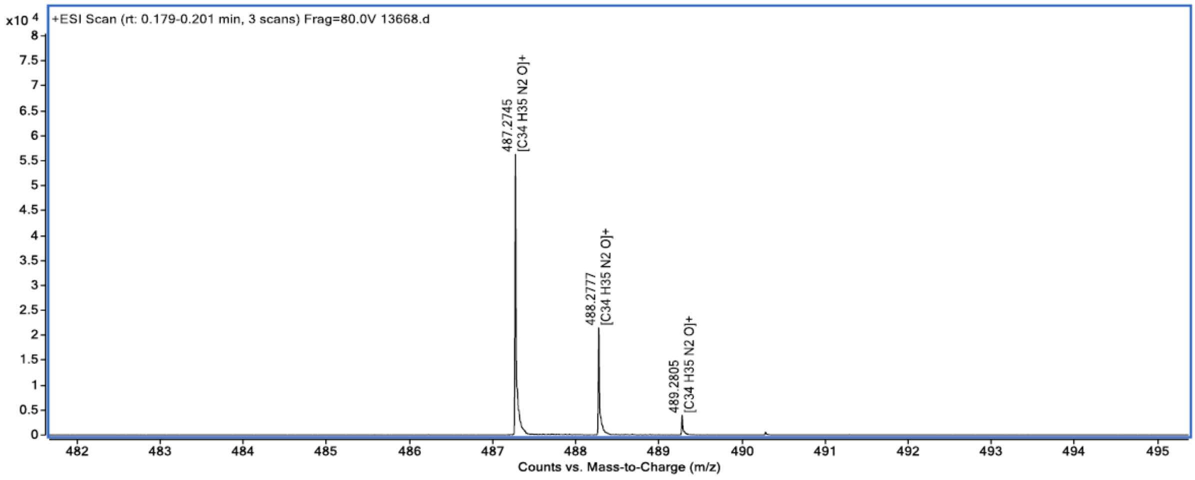

Figure S25. ESI–HRMS of compound **2·H[BF$_4$]**.

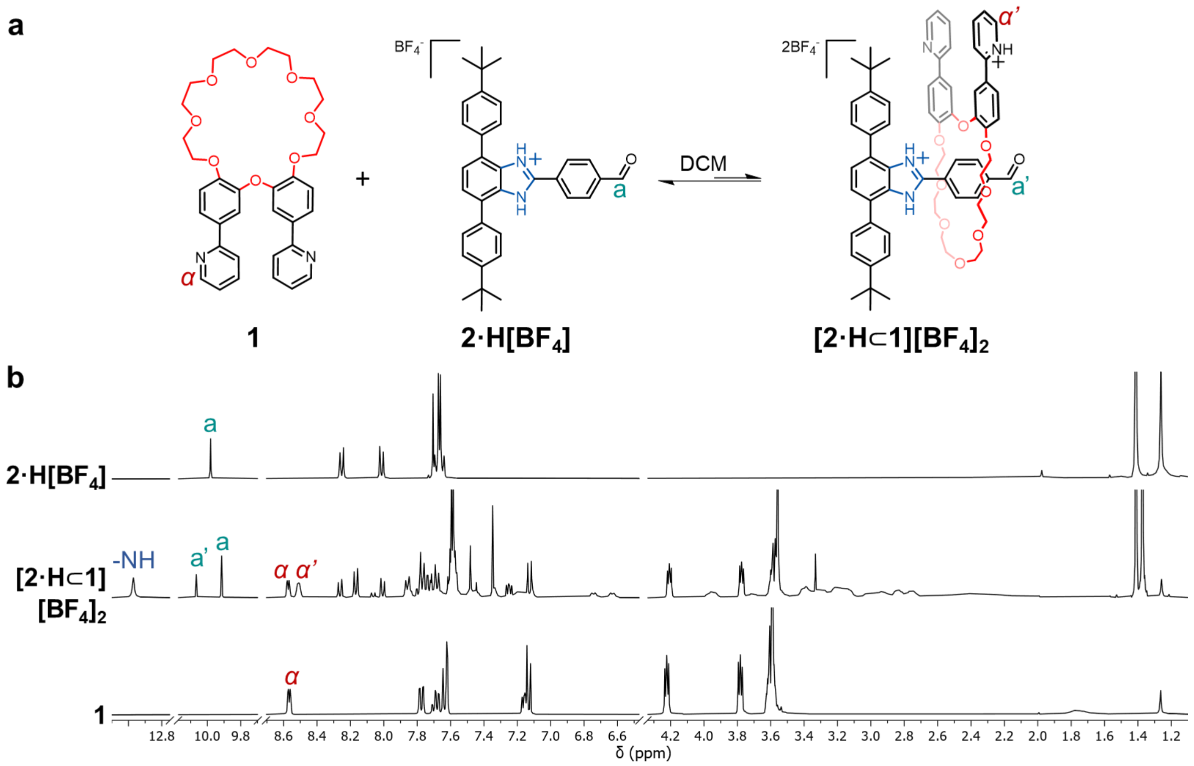

Figure S26.a. Reaction scheme showing the formation of pseudorotaxane [**2·H ⊂ 1][BF$_4$]$_2$** upon mixing the neutral macrocycle **1** and the T-shaped axle precursor **2·H[BF$_4$]** in DCM. **b.** (From top to bottom) ^{1}H NMR spectra (400 MHz, CD_2Cl_2) of the T-shaped axle precursor **2·H[BF$_4$]**, pseudorotaxane [**2·H ⊂ 1**][**BF$_4$]$_2$** formed by mixing **1** and **2·H[BF$_4$]** (4.7 mM) in a 1:1 molar ratio, and the macrocycle **1**.

Compound 1·$H_2[BF_4]_2$

Compound **1** (148 mg, 0.25 mmol) was suspended in CH_3CN (24 mL) in a polypropylene beaker. Next, HBF_4 (50-55 wt.% in Et_2O, 260 µL, 1.0 mmol) was added dropwise using a plastic syringe to produce a clear, light brown solution. The solvents were left to evaporate overnight inside a fume hood to afford a brown oil, which was dissolved in nitromethane (16 mL) and washed with water (3 × 10 mL). The organic fraction was dried over anhydrous $MgSO_4$ and concentrated in *vacuo* to obtain **1·$H_2[BF_4]_2$** as a brown oil (147 mg, 0.19 mmol, 77%). **^{1}H NMR (400 MHz, $CDCl_3$)**: δ 8.65 – 8.64 (*m*, 2H), 8.09 (*td*, *J* = 8.1, 1.5 Hz, 2H), 7.88 (*d*, *J* = 8.2 Hz, 2H), 7.67 (*dd*, *J* = 8.6, 2.2 Hz, 2H), 7.56 – 7.53 (*m*, 2H), 7.51 (*d*, *J* = 2.2 Hz, 2H), 7.15 (*d*, *J* = 8.6 Hz, 2H), 4.20 – 4.18 (*m*, 4H), 3.79 – 3.77 (*m*, 4H), 3.59 – 3.54 (*m*, 16H). **^{13}C{^{1}H} NMR (101 MHz, $CDCl_3$)**: δ 154.1, 152.8, 147.1, 145.9, 142.6, 127.9, 124.6, 124.0, 123.3, 118.7, 115.5, 71.4, 71.14, 71.09, 71.05, 70.1, 69.8.

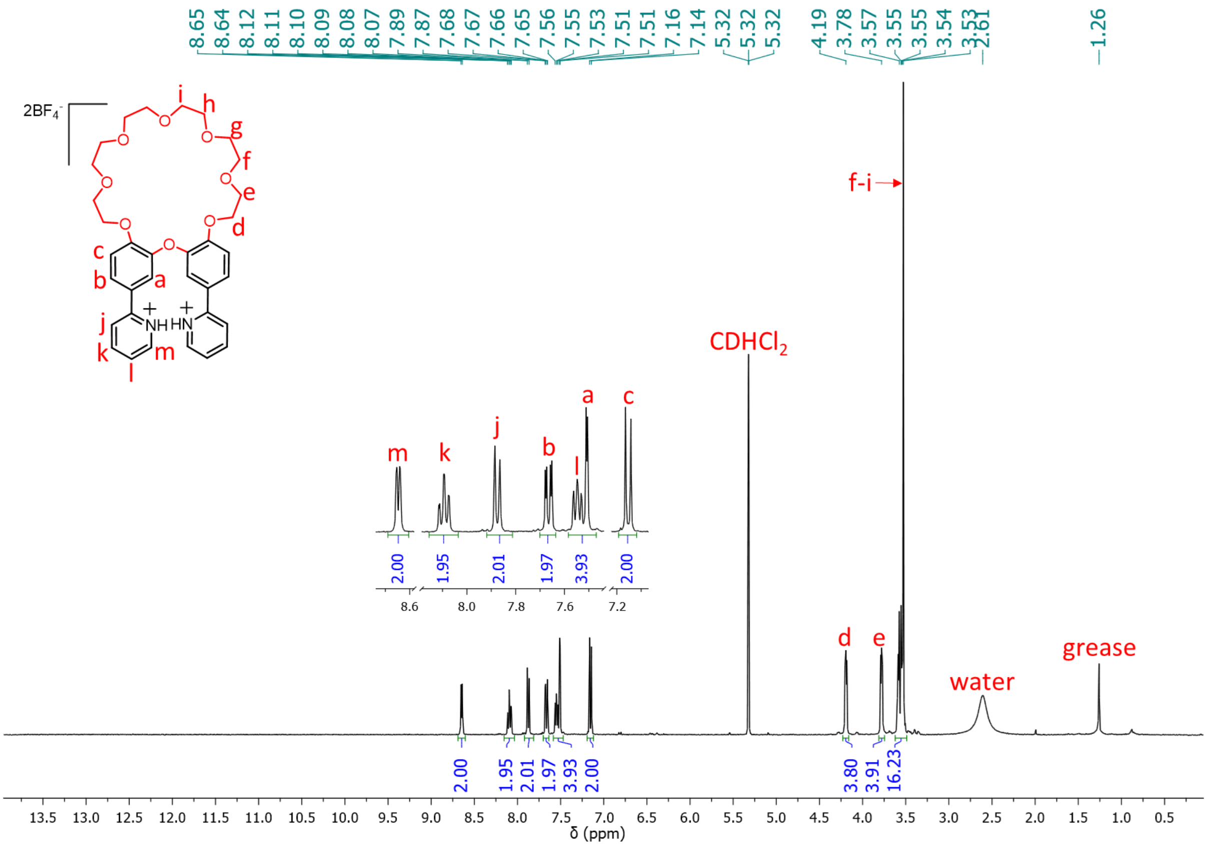

Figure S27. ^{1}H NMR spectrum (400 MHz, CD_2Cl_2) of compound **1·$H_2[BF_4]_2$**.

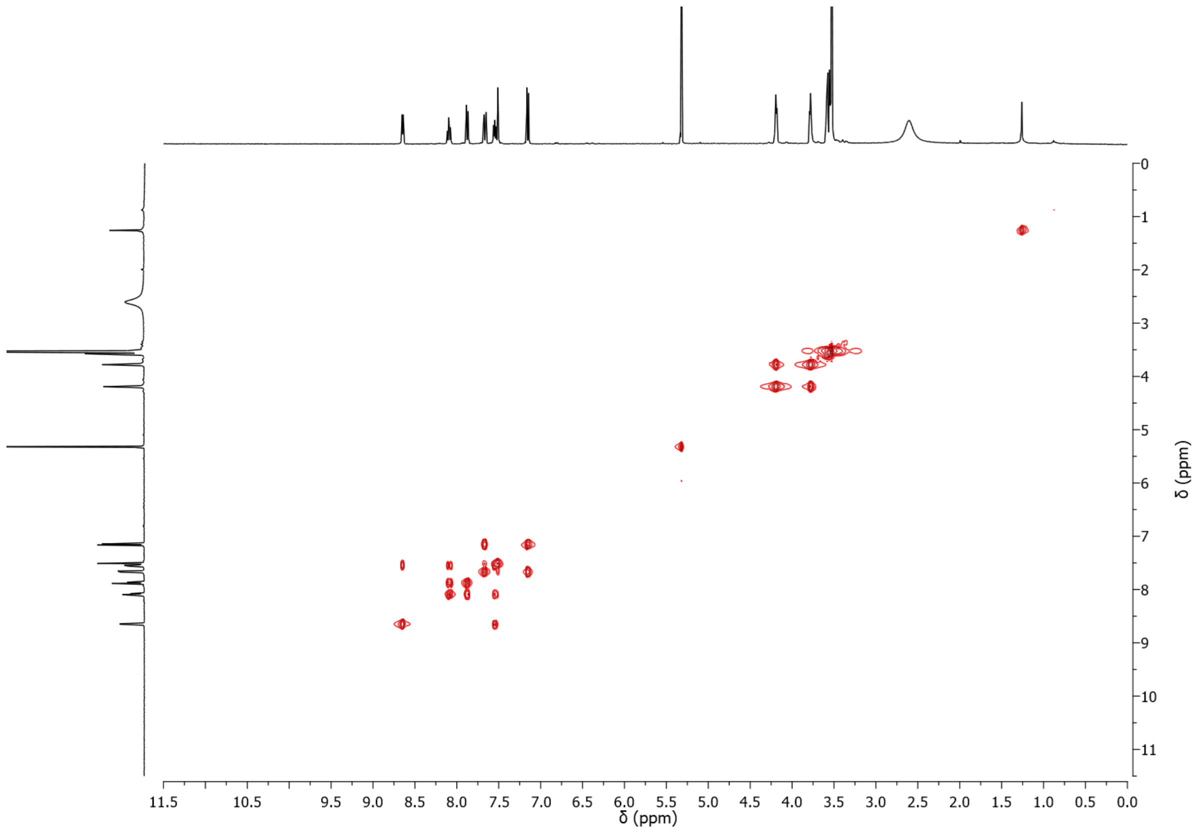

Figure S28. ^{1}H-^{1}H COSY NMR spectrum (400 MHz, CD_2Cl_2) of compound **1·H$_2$[BF$_4$]$_2$**.

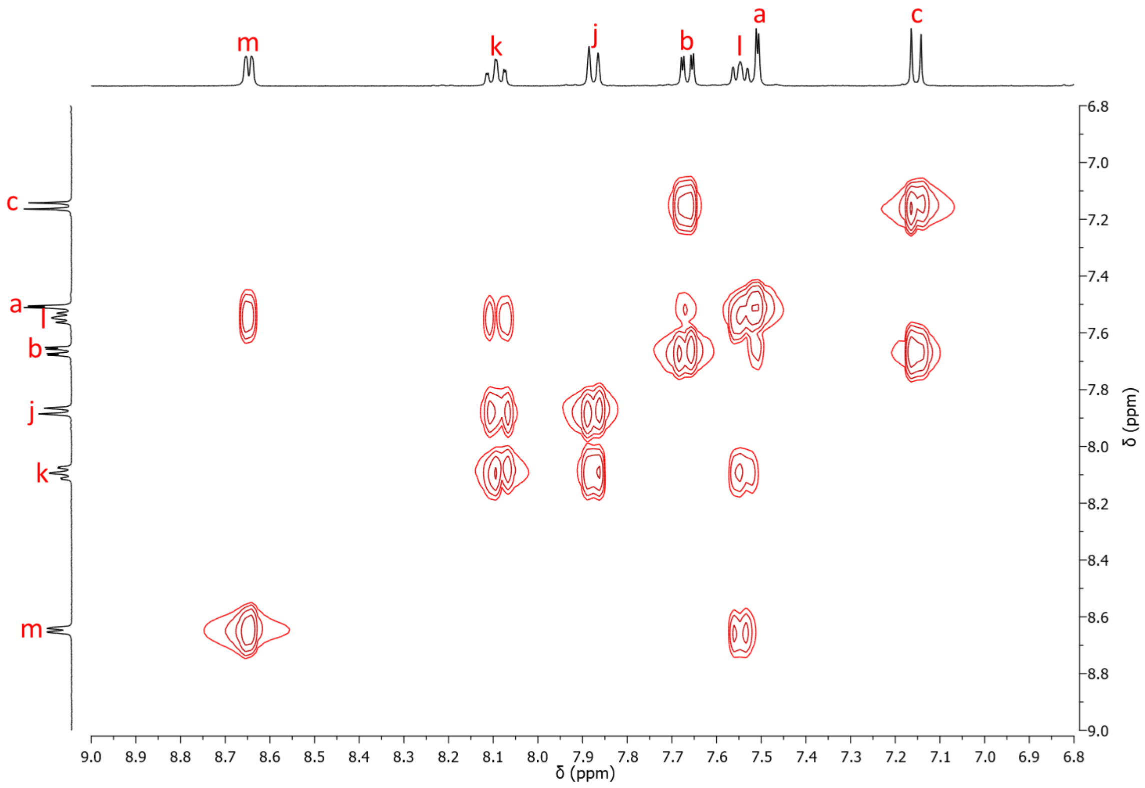

Figure S29. Partial ^{1}H-^{1}H COSY NMR spectrum of compound **1·H$_2$[BF$_4$]$_2$**.

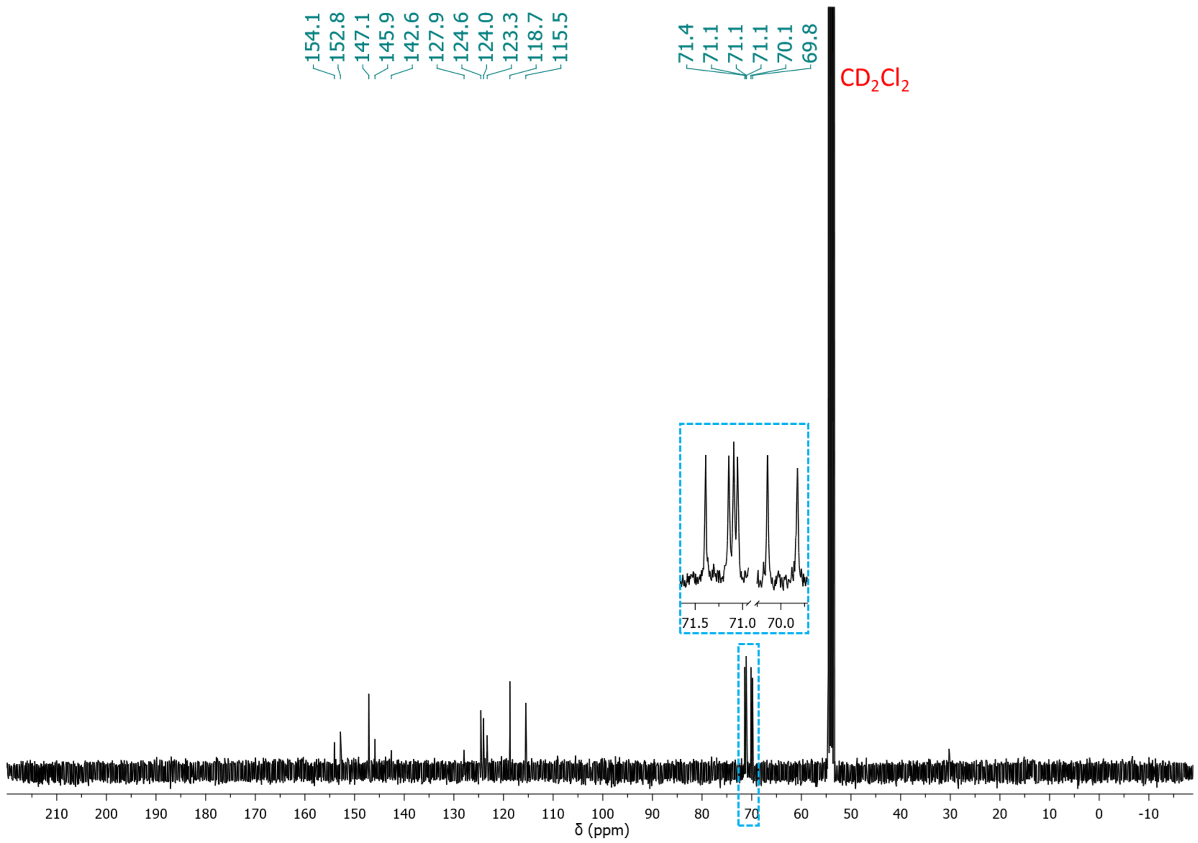

Figure S30. $^{13}C\{^{1}H\}$ NMR spectrum (101 MHz, CD_2Cl_2) of compound **$1{\cdot}H_2[BF_4]_2$**.

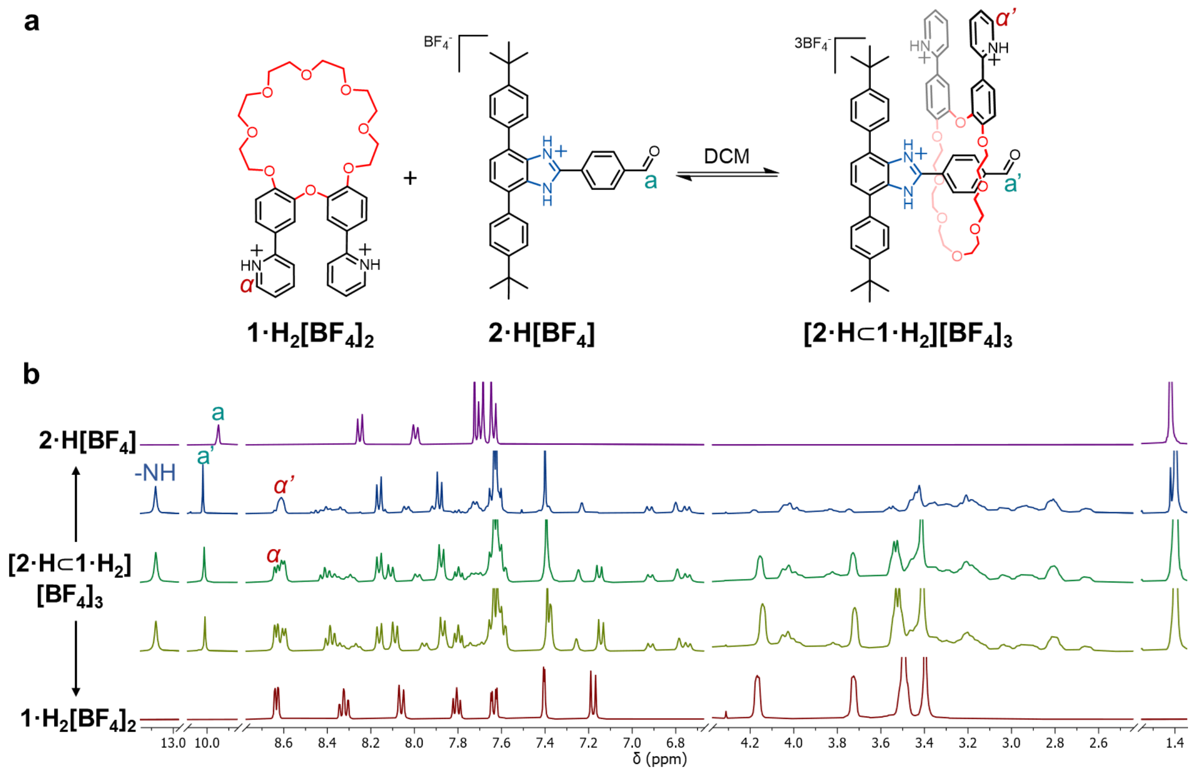

Figure S31.a. Reaction scheme for the formation of pseudorotaxane **[2·H⊂1·H$_2$][BF$_4$]$_3$** upon mixing the macrocycle **1·H$_2$[BF$_4$]$_2$** and the T-shaped axle precursor **2·H[BF$_4$]** in DCM. **b.** (From top to bottom) ^{1}H NMR spectra (400 MHz, CD_2Cl_2) of the T-shaped axle precursor **2·H[BF$_4$]**, pseudorotaxane [**2·H⊂1·H$_2$][BF$_4$]$_3$** formed by mixing **1·H$_2$[BF$_4$]$_2$** and **2·H[BF$_4$]** (4.7 mM) in a 1:1, 1:1.5, and 1:2 molar ratios, and the macrocycle **1·H$_2$[BF$_4$]$_2$**.

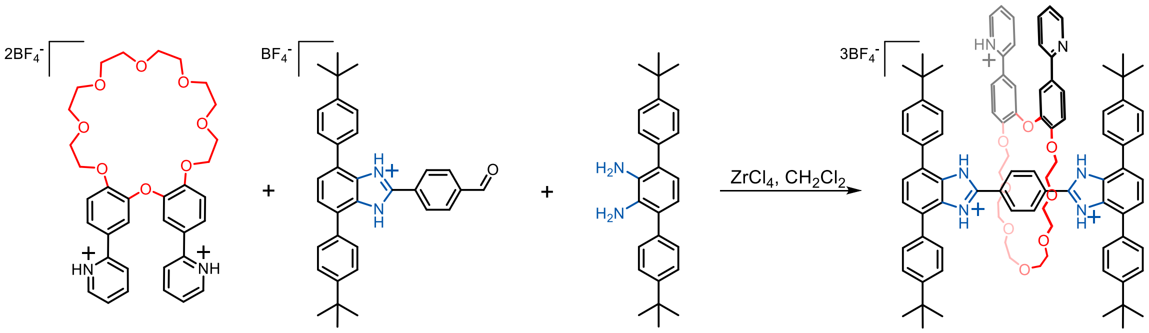

1·H$_2$[BF$_4$]$_2$ **2·H[BF$_4$]** **H** **3·H$_3$[BF$_4$]$_3$**

Scheme S3: Synthesis of [2]rotaxane **3·H$_3$[BF$_4$]$_3$**.

Compound 3·H$_3$[BF$_4$]$_3$

Compounds **1·H$_2$[BF$_4$]$_2$** (194 mg, 0.25 mmol) and **2·H[BF$_4$]** (82 mg, 0.14 mmol) were dissolved in DCM (31 mL) and stirred for 10 minutes. Next, $ZrCl_4$ (4 mg, 0.014 mmol) was added followed by compound **H** (58 mg, 0.16 mmol), and the mixture was stirred at room temperature for 24 h. After removing the solvent under vacuum, the product was purified by column chromatography on silica gel using ethyl acetate:hexanes (1:1) as the eluent first, followed by DCM:acetone (8:2, v/v) to elute the product **3·H$_3$[BF$_4$]$_3$** (R_f = 0.56) , which was isolated as a yellow solid (193 mg, 0.11 mmol, 79%). **^{1}H NMR (400 MHz, CD_2Cl_2)**: δ 12.91 (*s*, 2H), 10.56 (*s*, 2H), 8.52 (*m*, 1H), 8.12 – 8.09 (*m*, 3H), 8.04 – 8.02 (*dd*, 2H), 7.70 – 7.54 (*m*, 18H), 7.45 – 7.37 (*m*, 8H), 7.29 – 7.27 (*m*, 1H), 7.13 – 7.09 (*m*, 2H), 6.87 (*m*, 1H), 6.78 – 6.76 (*d*, *J* = 8.1 Hz, 1H), 6.69 – 6.67 (*d*, *J* = 8.1 Hz, 1H), 3.96 – 3.81 (*m*, 3H), 3.59 – 3.52 (*m*, 5H), 3.32 – 3.14 (*m*, 10H), 2.96 – 2.76 (*m*, 6H), 1.39 (*s*, 18H), 1.36 (*s*, 18H). **^{13}C{^{1}H} NMR (101 MHz, CD_2Cl_2)**: δ 156.20, 156.0, 152.5, 151.7, 151.0, 150.9, 150.0, 149.5, 146.2, 144.9, 137.2, 137.0, 136.0, 133.9, 133.8, 133.6, 133.1, 129.7, 129.3, 127.7, 126.6, 126.6, 126.3, 125.7, 123.7, 122.4, 122.1, 120.0, 119.2, 116.5, 113.7, 112.8, 71.3, 70.9, 70.8, 70.2, 70.1, 69.8, 68.4, 67.8, 35.2, 35.1, 31.7, 31.6. **ESI-HRMS:** [**3** + H$^+$] *m/z* = 1442.7709 (experimental), *m/z* = 1442.7709 (calculated), relative error = 0 ppm. The synthesis of **3·H$_3$[BF$_4$][PF$_6$]$_2$** was also tested with **1·H$_2$[PF$_6$]$_2$** instead of **1·H$_2$[BF$_4$]$_2$**; all the reaction parameters remained the same and comparable yields were obtained.

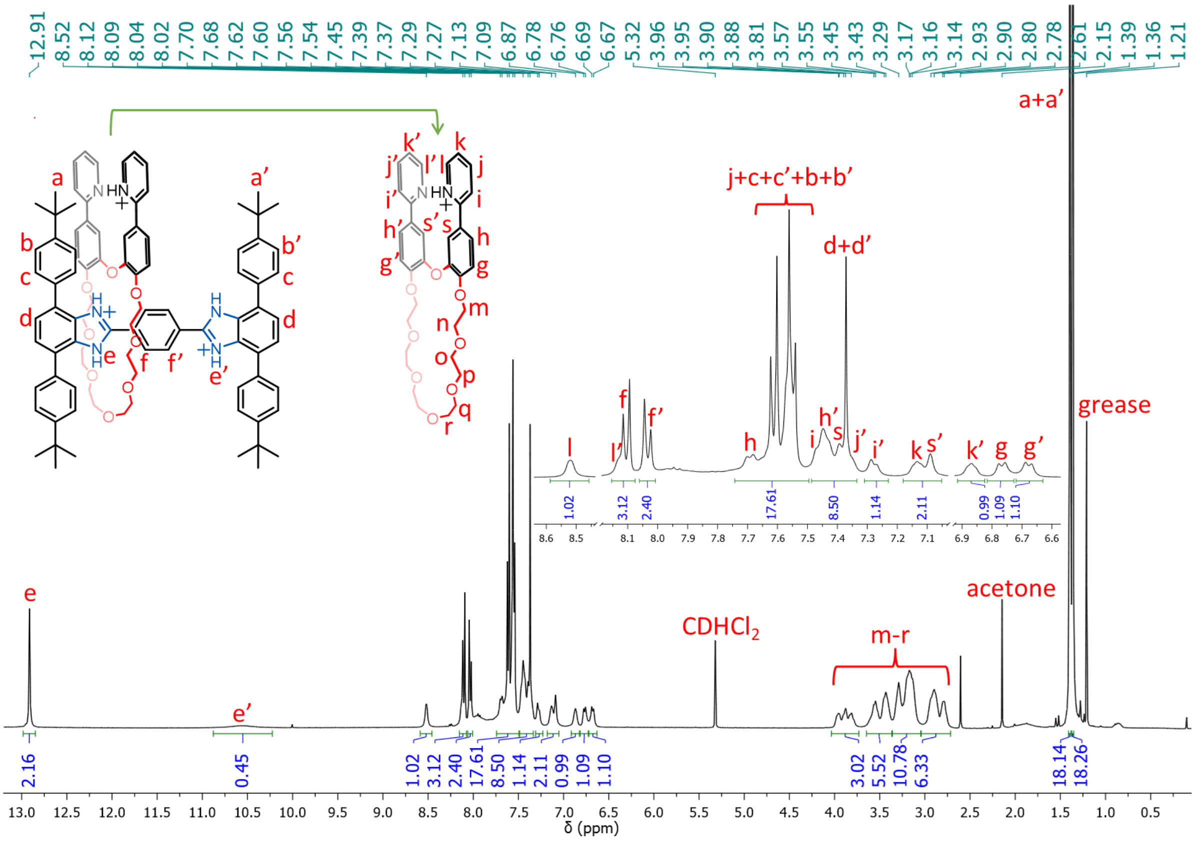

Figure S32. ^{1}H NMR spectrum (400 MHz, CD_2Cl_2) of compound **3·H$_3$[BF$_4$]$_3$**.

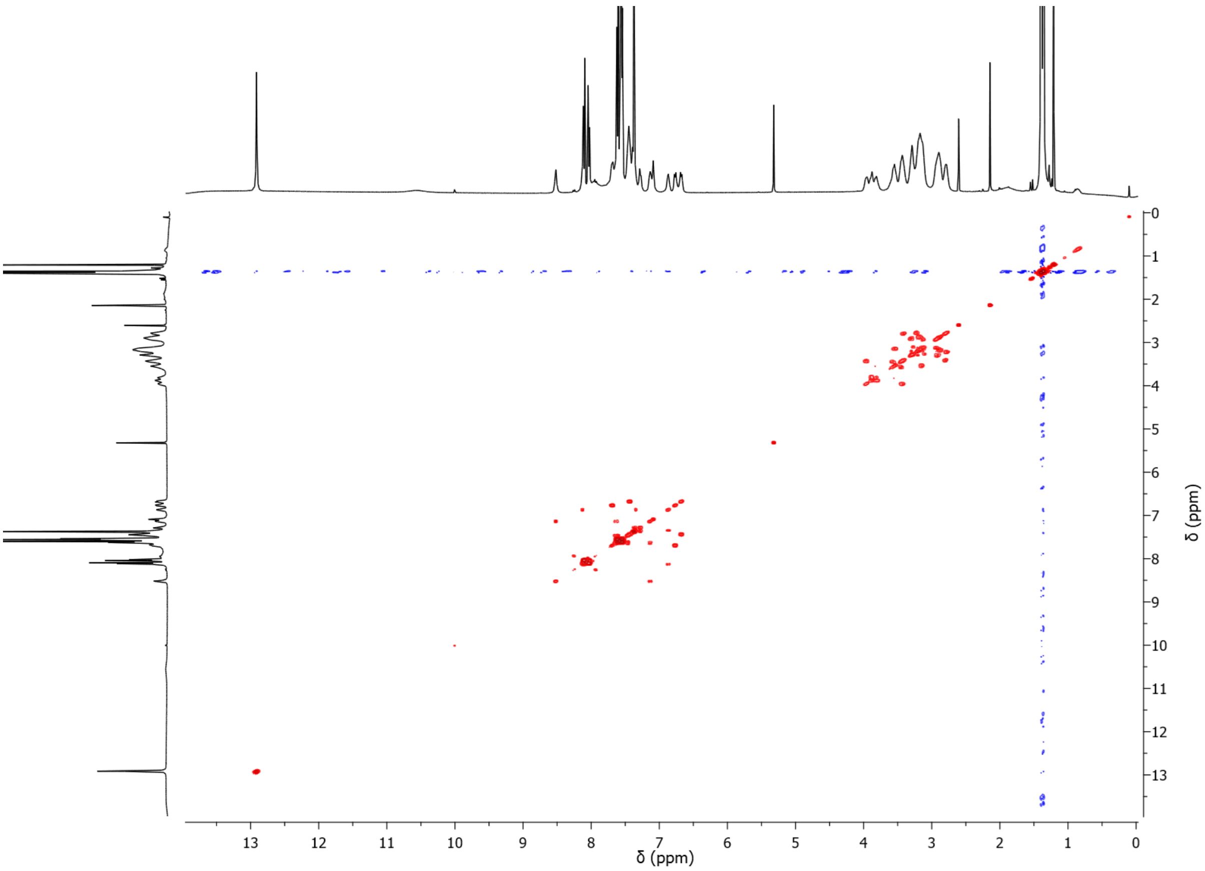

Figure S33. ^{1}H-^{1}H COSY NMR spectrum (400 MHz, CD_2Cl_2) of compound **3·H$_3$[BF$_4$]$_3$**.

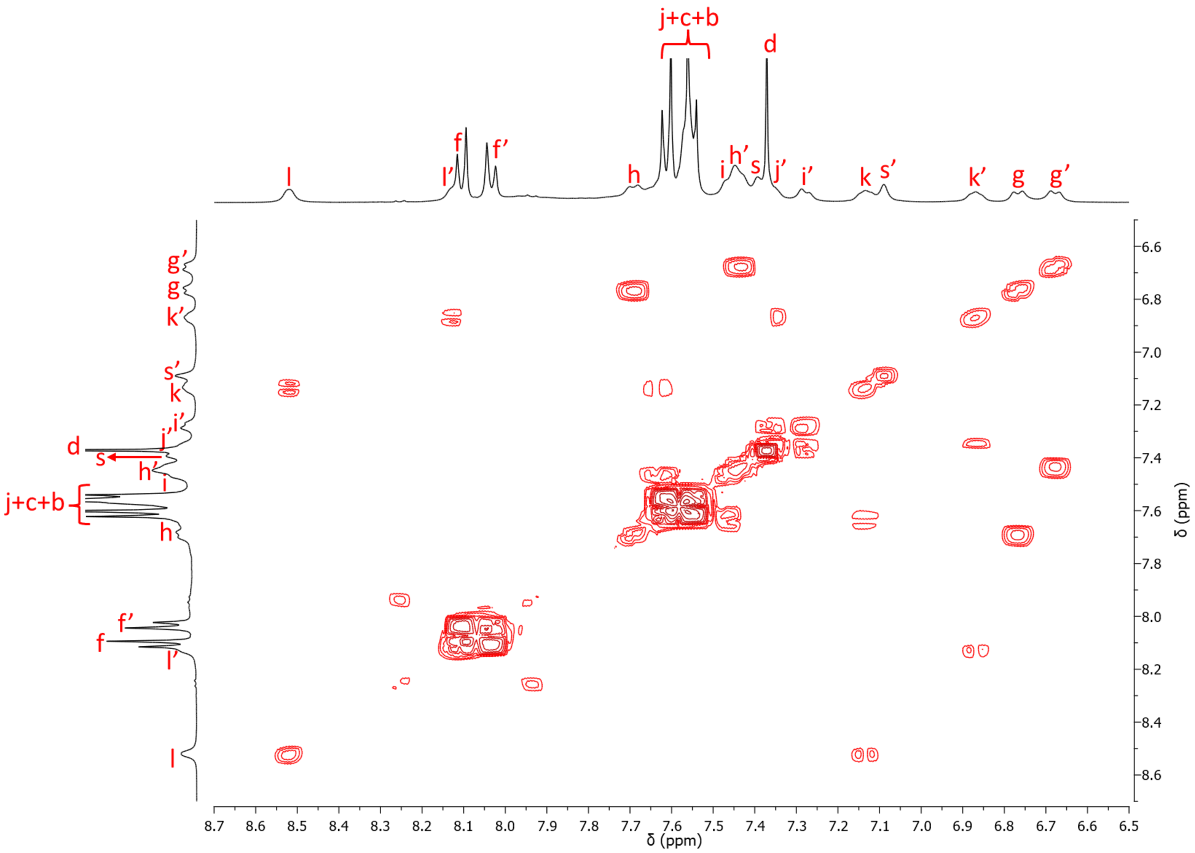

Figure S34. Partial ^{1}H-^{1}H COSY NMR spectrum (400 MHz, CD_2Cl_2) of compound **3·H$_3$[BF$_4$]$_3$**.

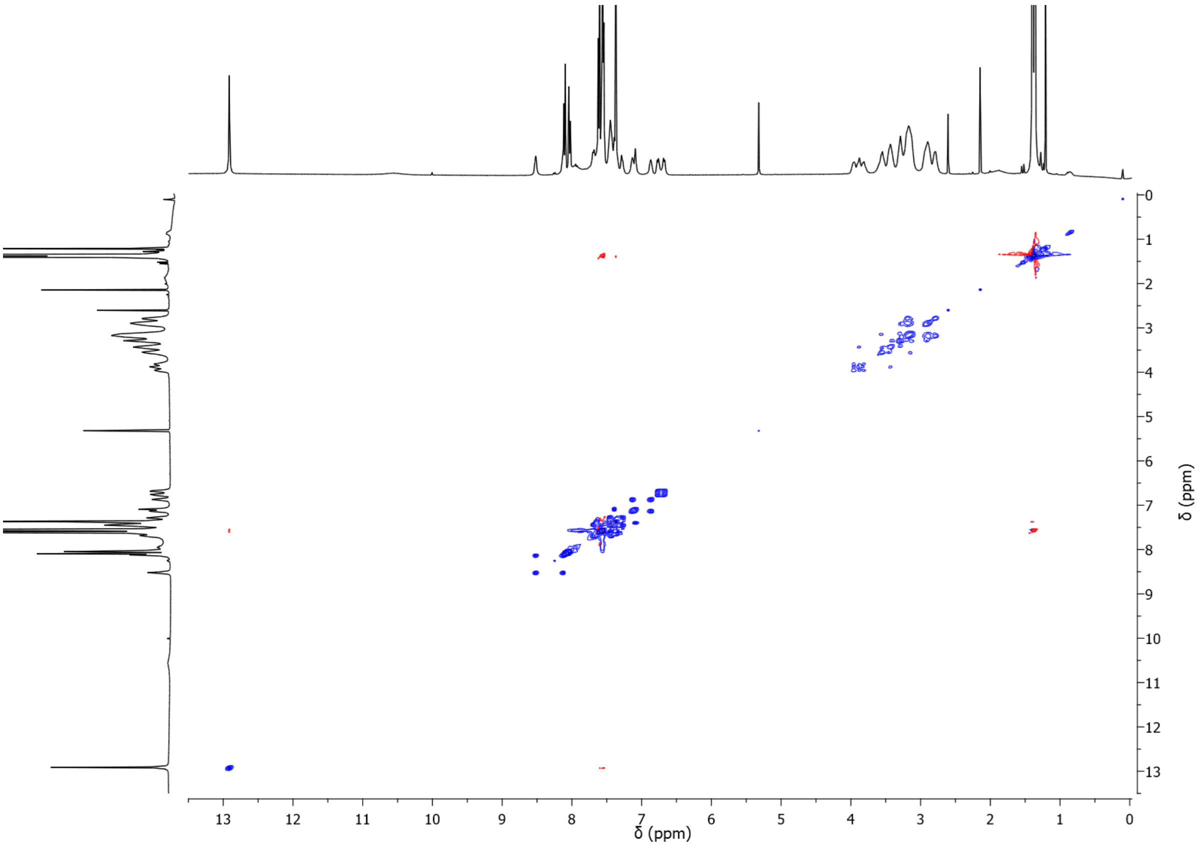

Figure S35. ^{1}H-^{1}H NOESY NMR spectrum (400 MHz, CD_2Cl_2) of compound **3·H$_3$[BF$_4$]$_3$**.

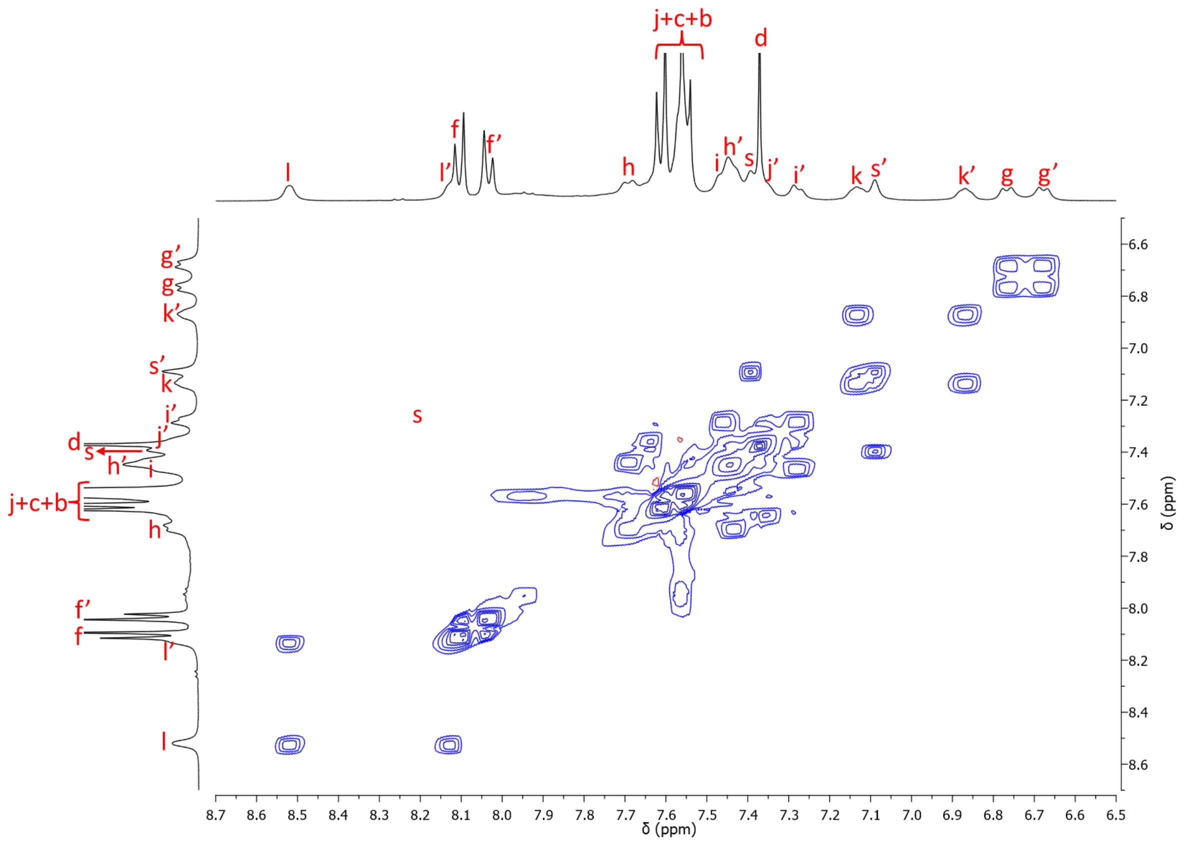

Figure S36. Partial ^{1}H-^{1}H NOESY NMR spectrum (400 MHz, CD_2Cl_2) of compound **3·H_3[BF_4]$_3$**.

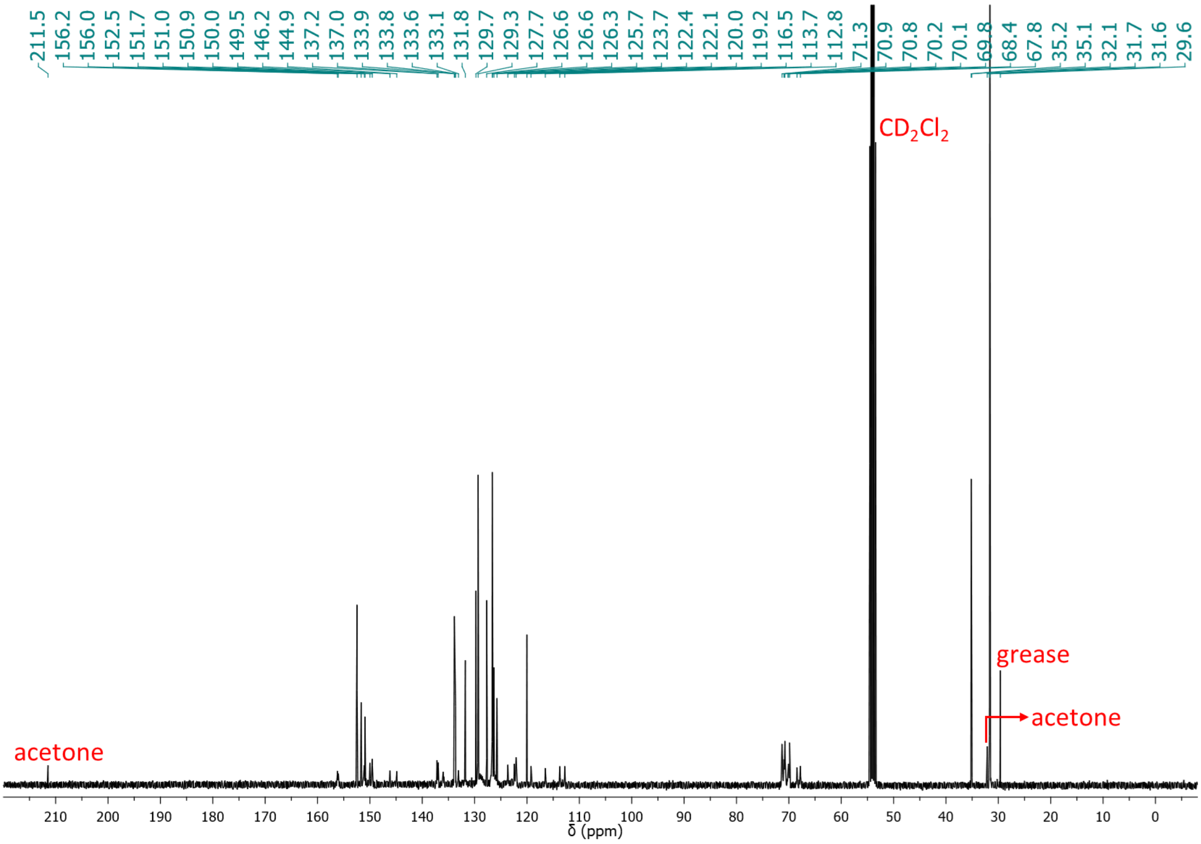

Figure S37. ^{13}C{^{1}H} NMR spectrum (101 MHz, CD_2Cl_2) of compound **3·H_3[BF_4]$_3$**.

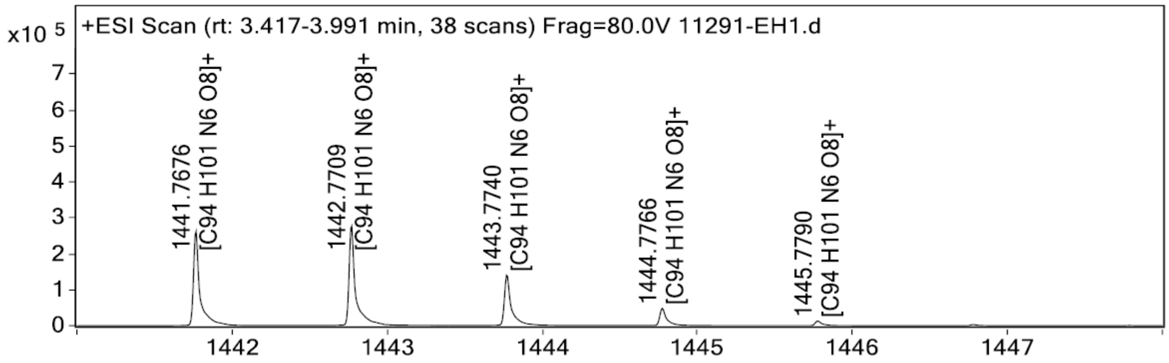

Figure S38. ESI-HRMS of compound **3·H$_3$[BF$_4$]$_3$**.

Compound 3

Triethylamine (140 μL, 0.1 mmol) was added to a solution of compound **3·H$_3$[BF$_4$]$_3$** (51 mg, 0.03 mmol) in CH_3CN (4 mL) and the mixture was heated to reflux at 82 °C for 1 h. After the reaction mixture cooled down to the room temperature, the precipitates were filtered, washed with cold acetonitrile (3 × 3 mL, *T* ~ 0 °C), and dried under vacuum to obtain **3** as a pale white solid (0.027 g, 0.019 mmol, 64%). **^{1}H NMR (400 MHz, CD_2Cl_2)**: δ 10.74 (*s*, 2H), 8.51 (*ddd*, *J* = 4.9, 1.9, 0.9 Hz, 2H), 7.82 – 7.79 (*m*, 4H), 7.68 (*dd*, 8.6, 2.2 Hz, 2H), 7.61 – 7.55 (*m*, 6H), 7.51 – 7.48 (*m*, 6H), 7.39 (*d*, *J* = 7.6 Hz, 2H), 7.34 – 7.31 (*m*, 4H), 7.24 (*d*, *J* = 7.7 Hz, 2H), 7.08 (*ddd*, *J* = 7.4, 4.8, 1.2 Hz, 2H), 6.87 (*d*, *J* = 8.6 Hz, 2H), 3.95 – 2.90 (*m*, 24H), 1.37 (*s*, 18H), 1.25 (*s*, 18H). **^{13}C{^{1}H} NMR (101 MHz, CD_2Cl_2)**: δ 156.7, 153.2, 151.7, 150.9, 150.2, 150.0, 146.7, 143.0, 137.0, 136.6, 136.4, 133.7, 132.8, 131.7, 131.6, 129.7, 129.0, 128.7, 126.4, 125.5, 125.3, 123.3, 122.8, 122.0, 121.6, 120.1, 118.0, 113.5, 70.6, 70.3, 70.0, 69.8, 69.4, 68.5, 35.1, 34.9, 31.70, 31.65. **ESI-HRMS:** [**3** + H$^+$] *m/z* = 1442.7706 (experimental), *m/z* = 1442.7709 (calculated), relative error = -0.2 ppm.

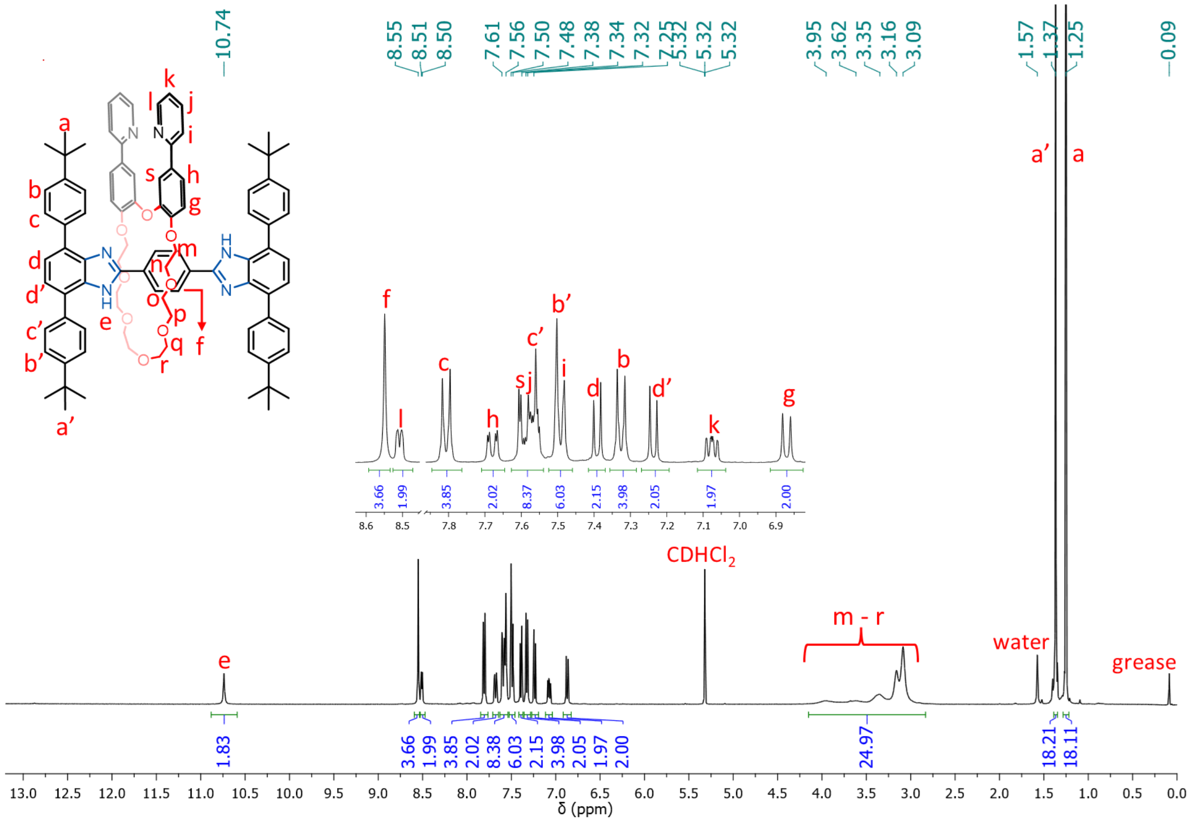

Figure S39. ^{1}H NMR spectrum (400 MHz, CD_2Cl_2) of compound **3**.

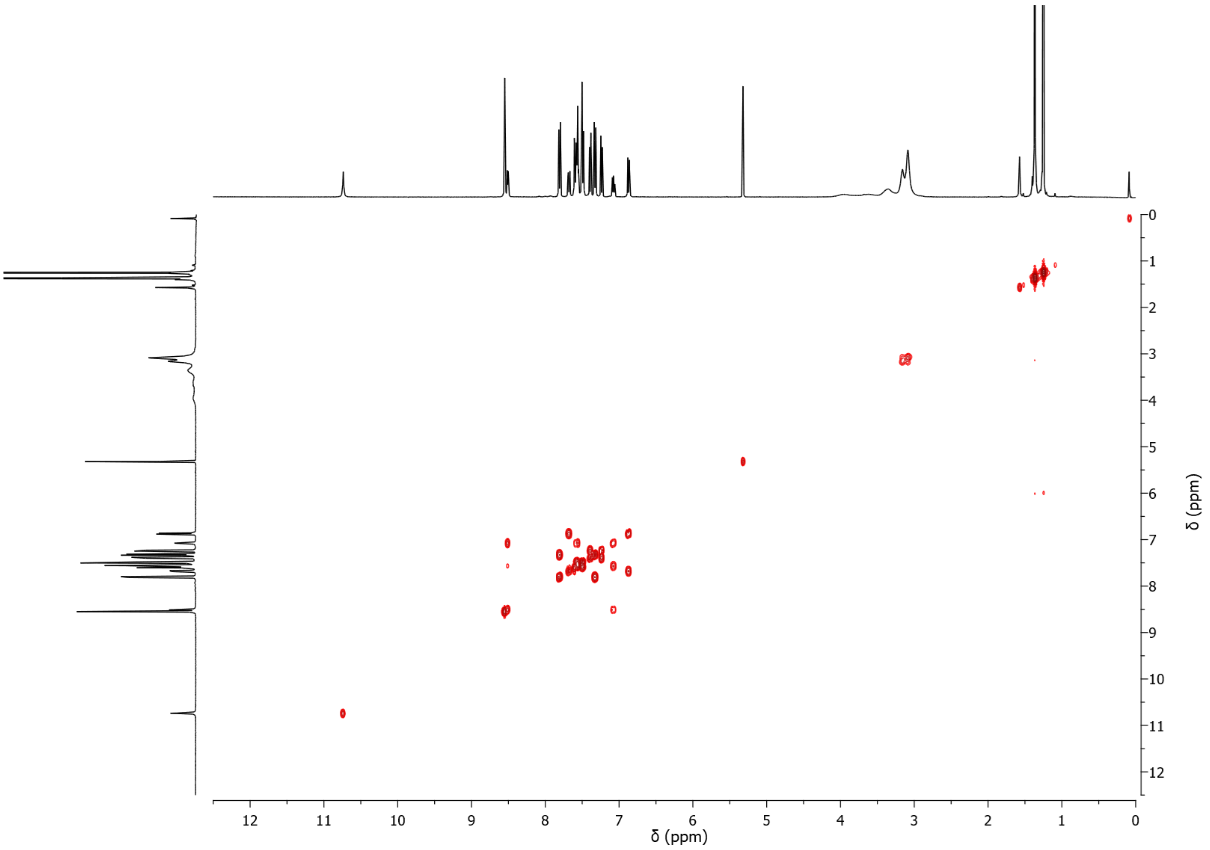

Figure S40. ^{1}H-^{1}H COSY NMR spectrum (400 MHz, CD_2Cl_2) of compound **3**.

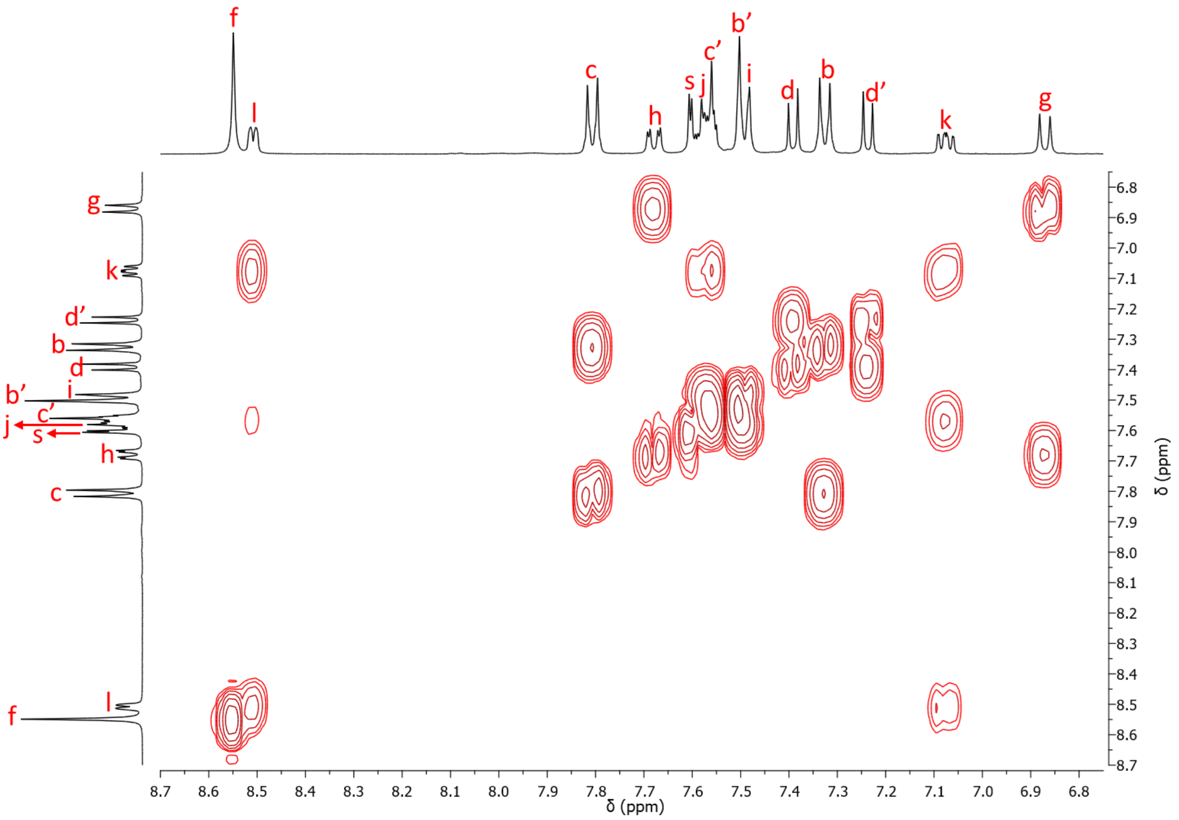

Figure S41. Partial ^1H-^1H COSY NMR spectrum (400 MHz, CD_2Cl_2) of compound **3**.

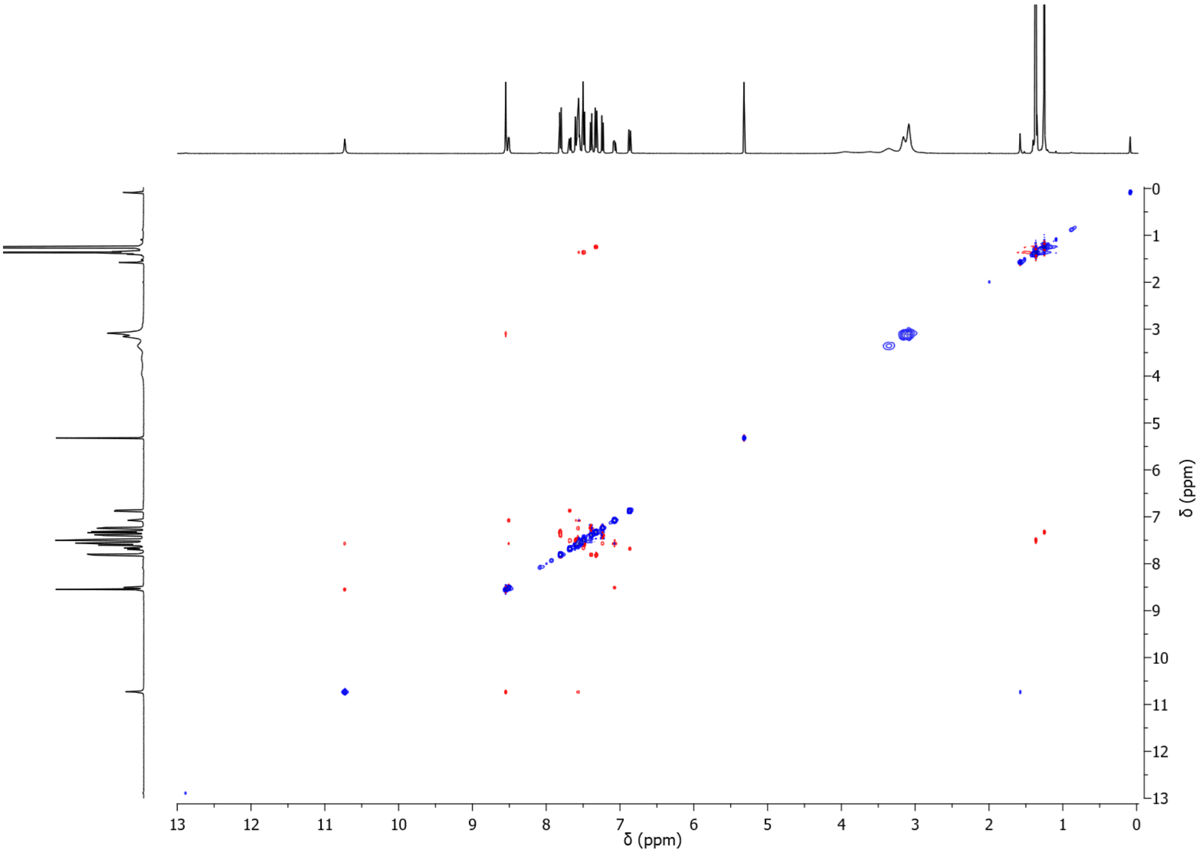

Figure S42. ^1H-^1H NOESY NMR spectrum (400 MHz, CD_2Cl_2) of compound **3**.

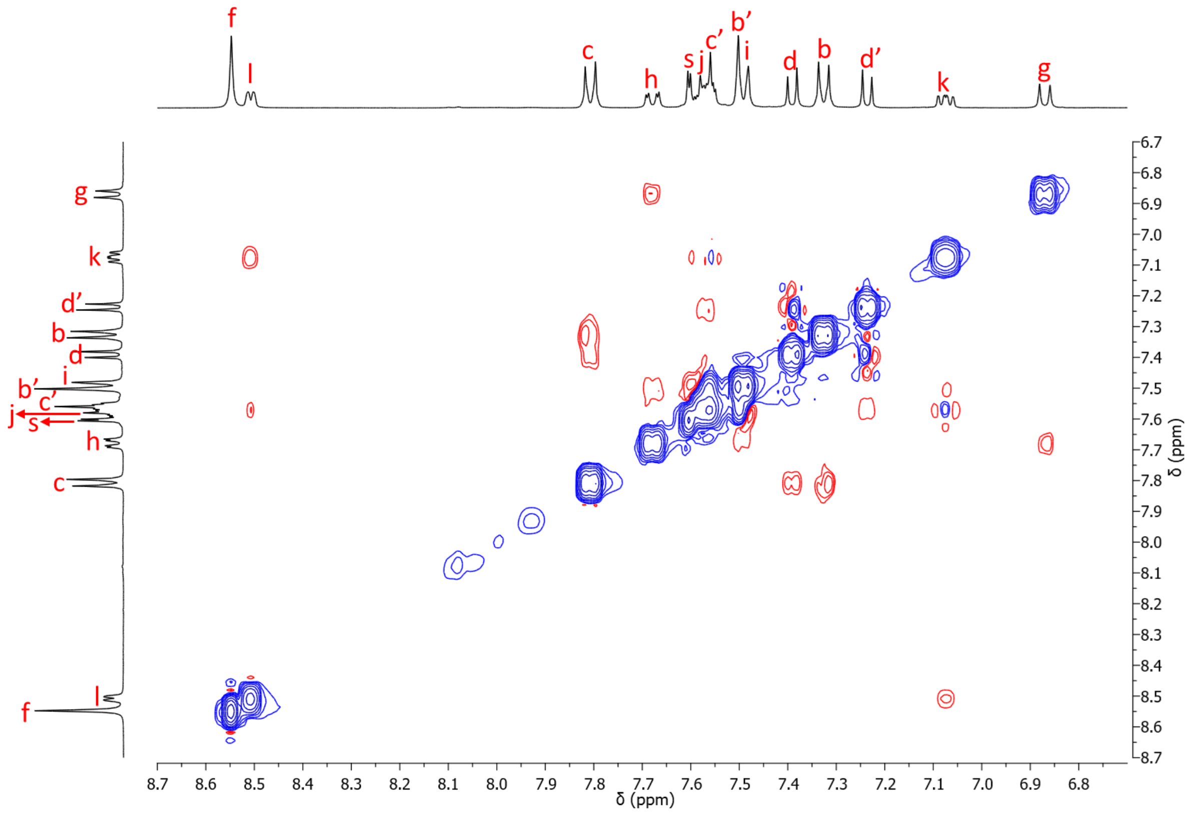

Figure S43. Partial ^{1}H-^{1}H NOESY spectrum (400 MHz, CD_2Cl_2) of compound **3.**

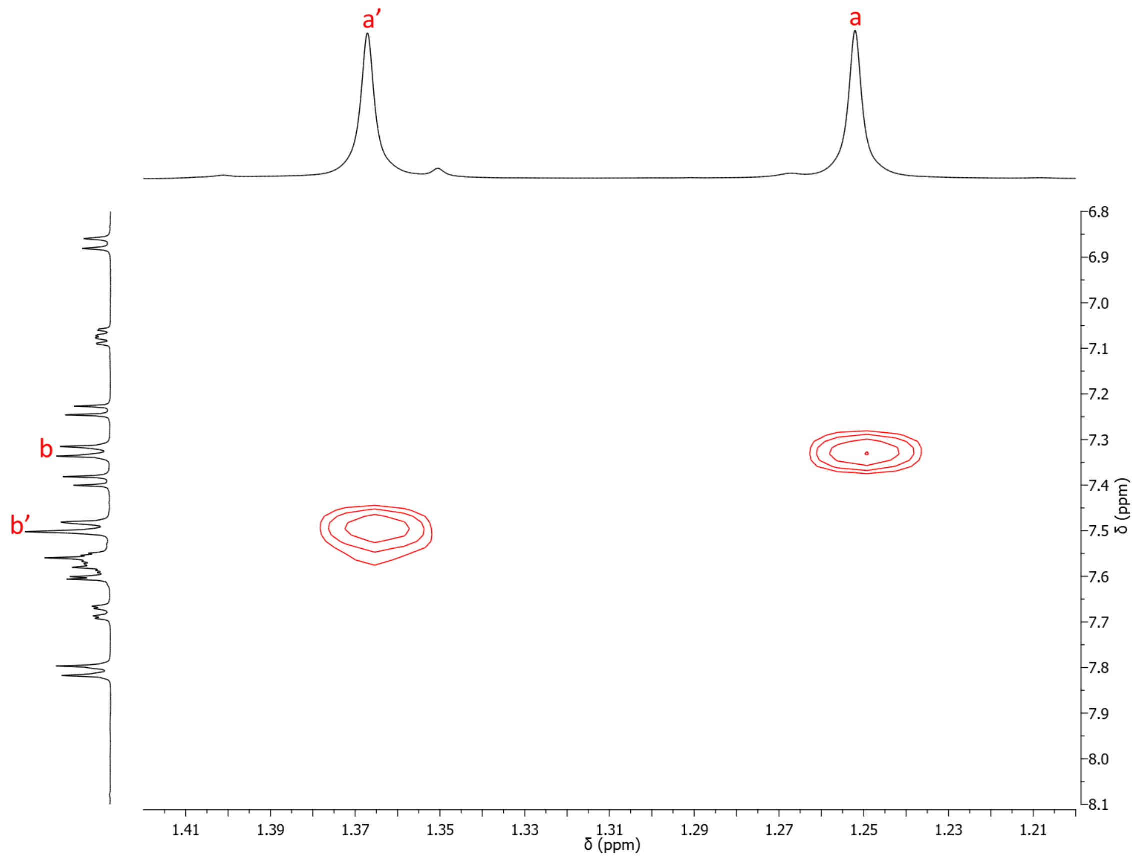

Figure S44. Partial ^{1}H-^{1}H NOESY spectrum (400 MHz, CD_2Cl_2) of compound **3.**

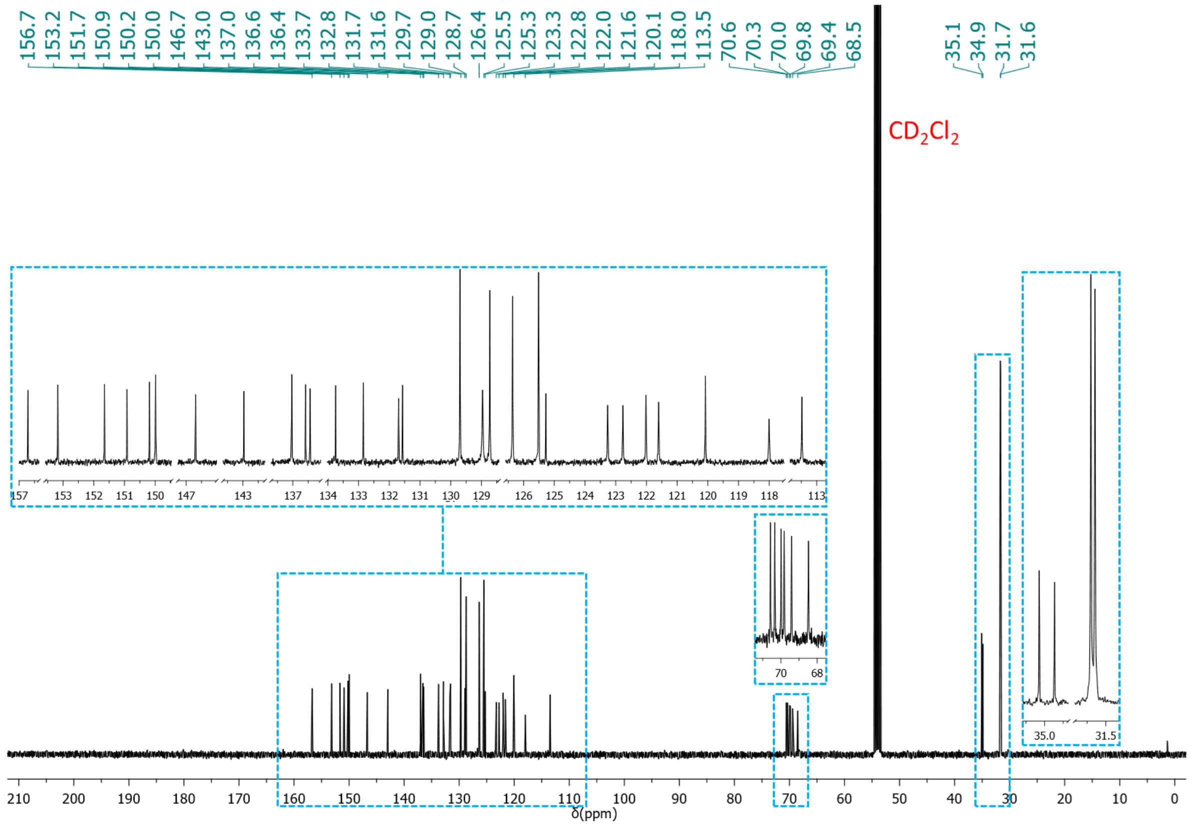

Figure S45. $^{13}C\{^{1}H\}$ NMR spectrum (101 MHz, CD_2Cl_2) of compound **3**.

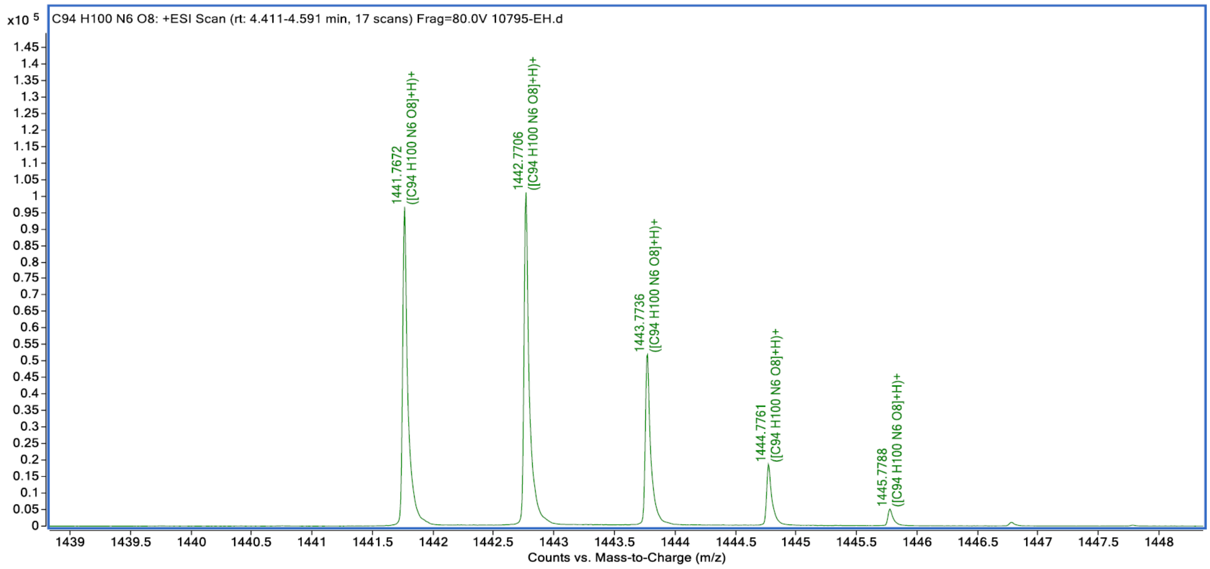

Figure S46. ESI-HRMS of compound **3**.

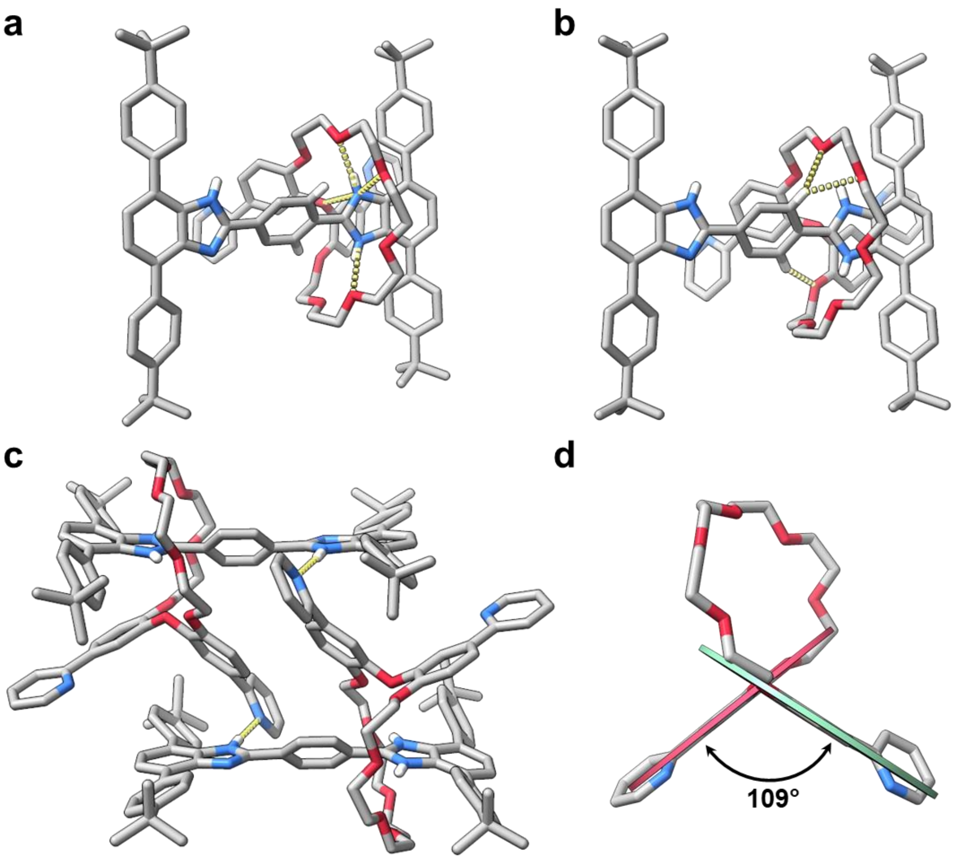

Figure S47. Different views of the solid-state molecular structure of the organic [2]rotaxane **3·H[BF$_4$]** as determined by SCXRD. Dashed yellow lines highlight **a.** N$^+$···O interactions, **b.** CH···O interactions, **c.** NH···N interactions between two adjacent [2]rotaxanes. Panel **d** shows the dihedral angle formed between the phenyl groups in the macrocycle. Counterions and solvent molecules have been omitted; only relevant hydrogen atoms are shown for clarity.

Compound 3-Pt^{II}

Compound **3-Pt^{II}** was synthesized from a previously reported procedure with some modifications.[15] A flask containing compound **3** (80 mg, 0.047 mmol) and $PtCl_2$ (15 mg, 0.056 mmol) was evacuated and backfilled with N_2 three times. Degassed benzonitrile (6.4 mL) was added and the reaction mixture was heated at 180 °C for 72 h.

Once the flask cooled to room temperature, the solvents were removed under vacuum to obtain a brown solid. This was identified as the protonated version of the product **3-Pt^{II}** because of the ^{1}H NMR signal at 13.12 ppm, which is indicative of the benzimidazolium proton involved in charge-assisted hydrogen bonding interactions with the macrocycle; see Figure S45. Therefore, to neutralize this, the isolated solid was transferred to a flask, evacuated, and backfilled with N_2 three times, and dissolved in degassed acetonitrile (15 mL) and triethylamine (200 µL, 1.4 mmol). The reaction was stirred at 82 °C for 1 h under N_2, followed by the evaporation of the solvent under vacuum. The residue was purified by column chromatography on silica gel using acetone:hexanes (1:2, v/v) as the mobile phase with 2.5% (v/v) triethylamine (R_f = 0.25) to obtain a yellow solid (55.8 mg, 0.034 mmol, 73%). **^{1}H NMR (400 MHz, CD_2Cl_2)**: δ 10.92 (*s*, 2H), 8.95 – 8.93 (*m*, 2H), 8.76 (*s*, 4H), 8.18 (*d*, *J* = 8.3 Hz, 4H), 7.90 – 7.89 (*m*, 4H), 7.58 (*d*, *J* = 8.3 Hz, 4H), 7.49 – 7.34 (*m*, 16H), 7.26 (*d*, *J* = 7.7 Hz, 2H), 6.62 (*d*, *J* = 8.3 Hz, 2H), 3.79 (*t*, *J* = 5.6 Hz, 4H), 3.44 – 3.42 (*m*, 4H), 3.35-3.33 (*m*, 4H), 3.28 (*t*, *J* = 5.6 Hz, 4H), 3.10 (*t*, *J* = 5.6 Hz, 4H), 3.00 (*t*, *J* = 5.6 Hz), 1.28 (*s*, 18H), 1.23 (*s*, 18H). **^{13}C{^{1}H} NMR (101 MHz, CD_2Cl_2)**: δ 166.1, 153.1, 150.6, 150.5, 150.2, 148.0, 142.8, 141.5, 138.9, 138.4, 136.2, 133.8, 131.4, 131.3, 129.5, 129.1, 128.7, 128.5, 126.2, 125.5, 125.3, 123.1, 121.5, 120.0, 119.4, 107.5, 70.61, 70.57, 69.9, 69.6, 67.2, 34.78, 34.75, 31.5, 31.4. **ESI-HRMS:** [**3-Pt^{II}** + H^+] *m/z* = 1635.7180 (experimental), *m/z* = 1635.7201 (calculated), relative error = -1.3 ppm. **UV-vis (DCM)**: λ_{max} = 268 nm (ε_{max} = 6.4 × 10^4 M^{-1} cm^{-1}); λ_{max} = 286 nm (ε_{max} = 6.3 × 10^4 M^{-1} cm^{-1}); λ_{max} = 323 nm (ε_{max} = 7.1 × 10^4 M^{-1} cm^{-1}).

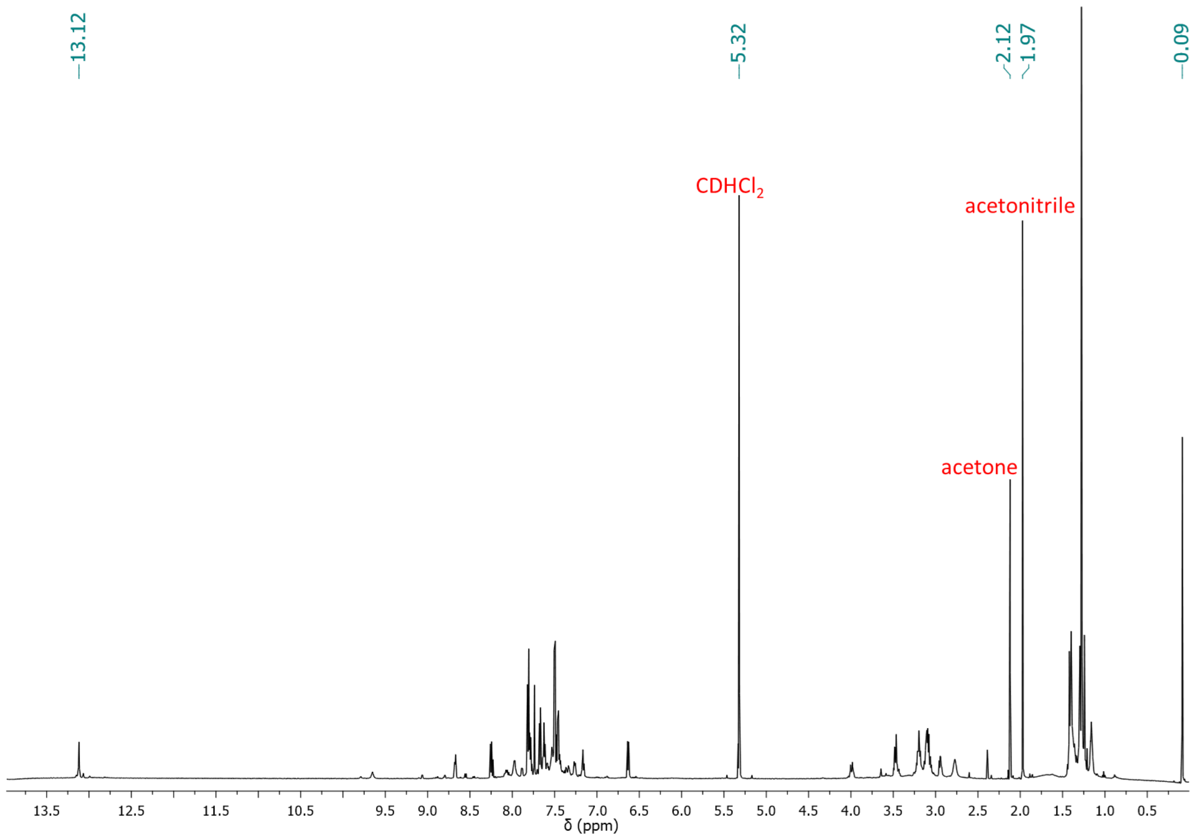

Figure S48. ^{1}H NMR spectrum (400 MHz, CD_2Cl_2) of the crude brown solid identified as the protonated product of **3-Pt^{II}**. This was based on the signal at 13.12 ppm, which is indicative of the benzimidazolium proton in the axle involved in charge-assisted hydrogen bonding interaction with the oxygen atoms in the macrocycle.

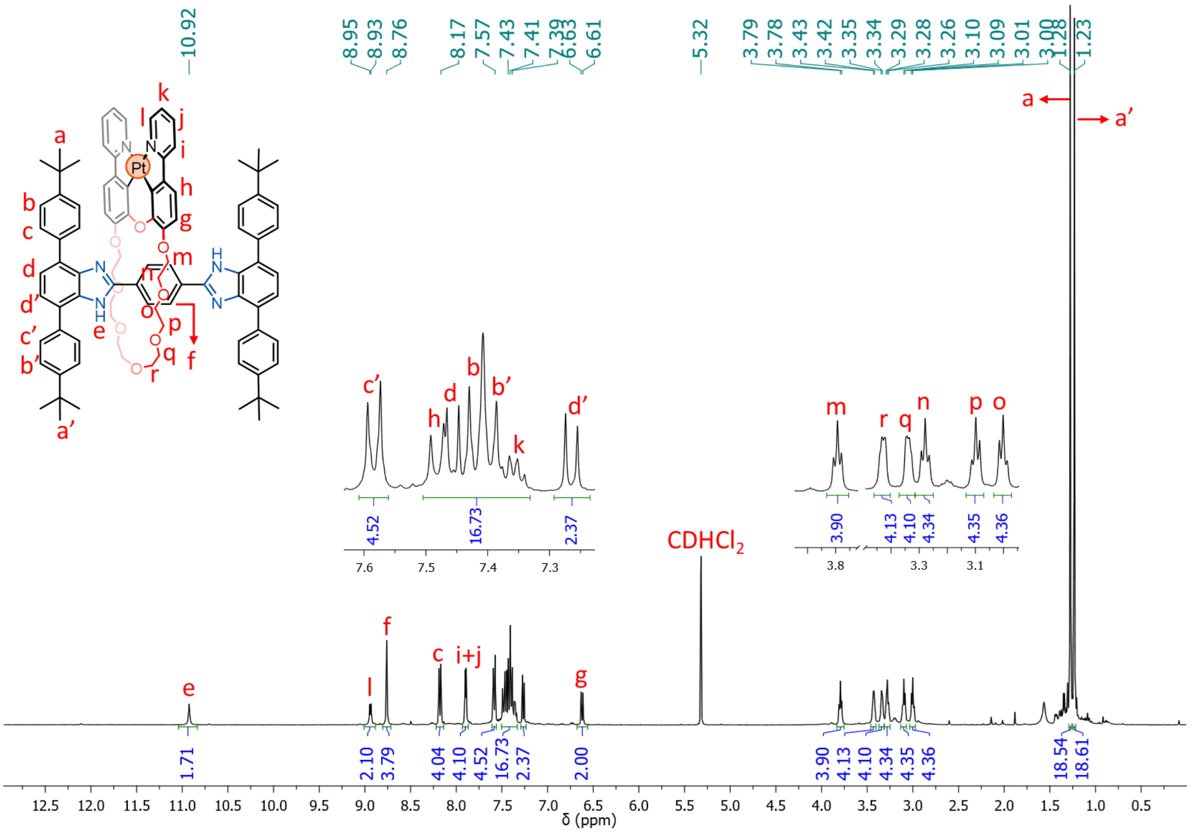

Figure S49. ^{1}H NMR spectrum (400 MHz, CD_2Cl_2) of compound **3-Pt^{II}**.

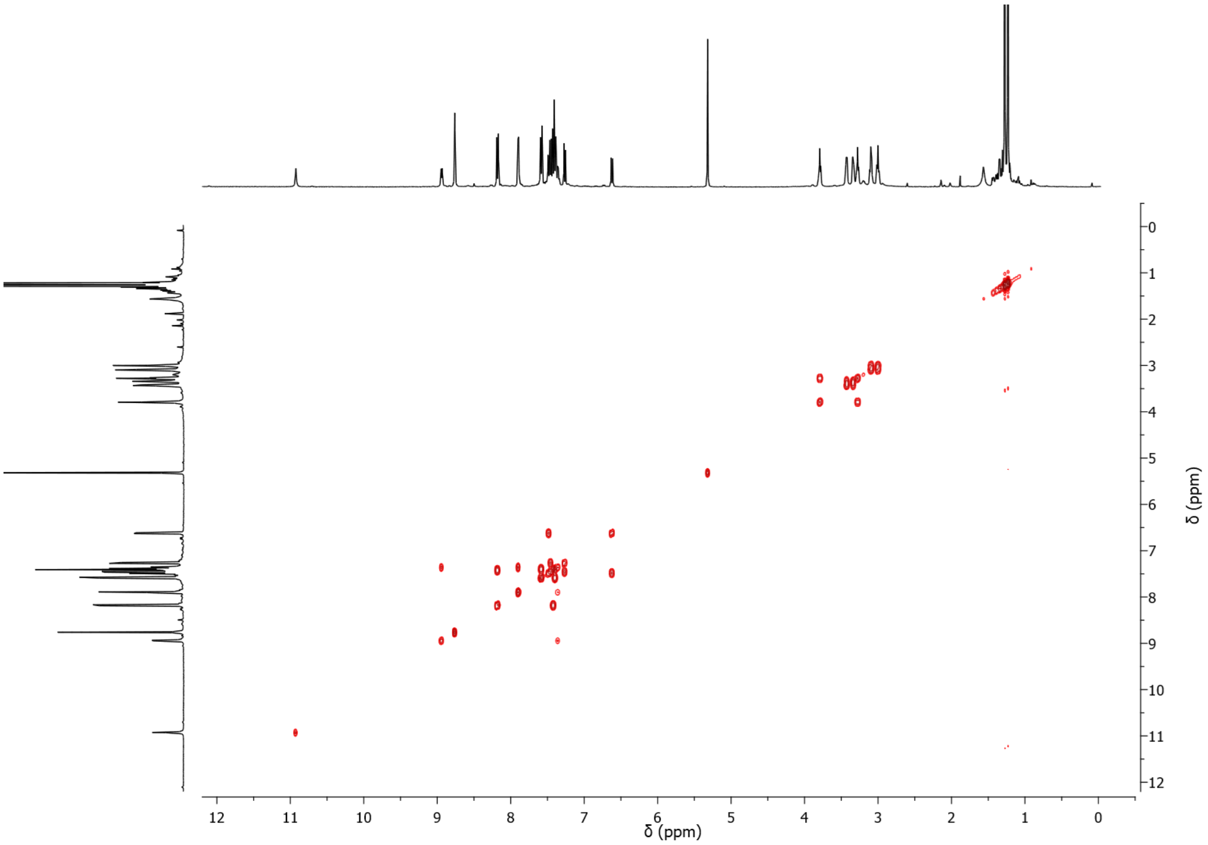

Figure S50. ^{1}H-^{1}H COSY NMR spectrum (400 MHz, CD_2Cl_2) of compound **3-Pt^{II}**.^{H}H

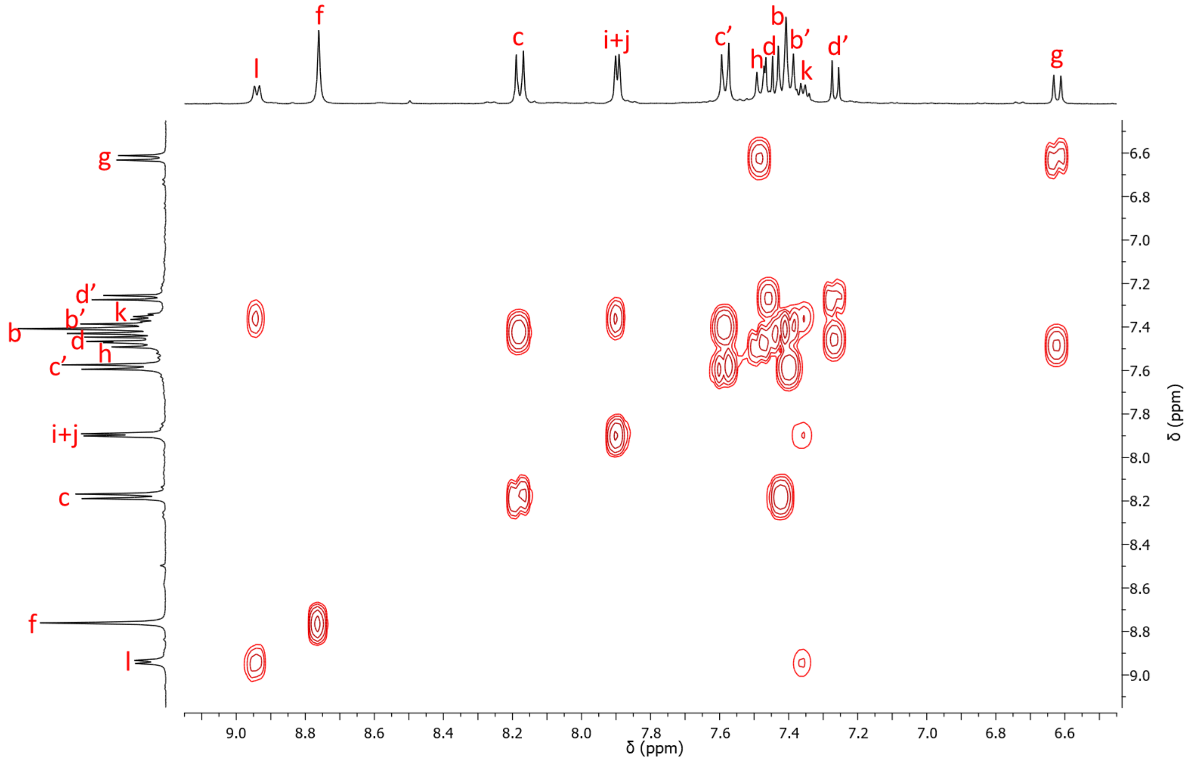

Figure S51. Partial ^{1}H-^{1}H COSY NMR spectrum (400 MHz, CD_2Cl_2) of compound **3-Pt^{II}**.^{H}H

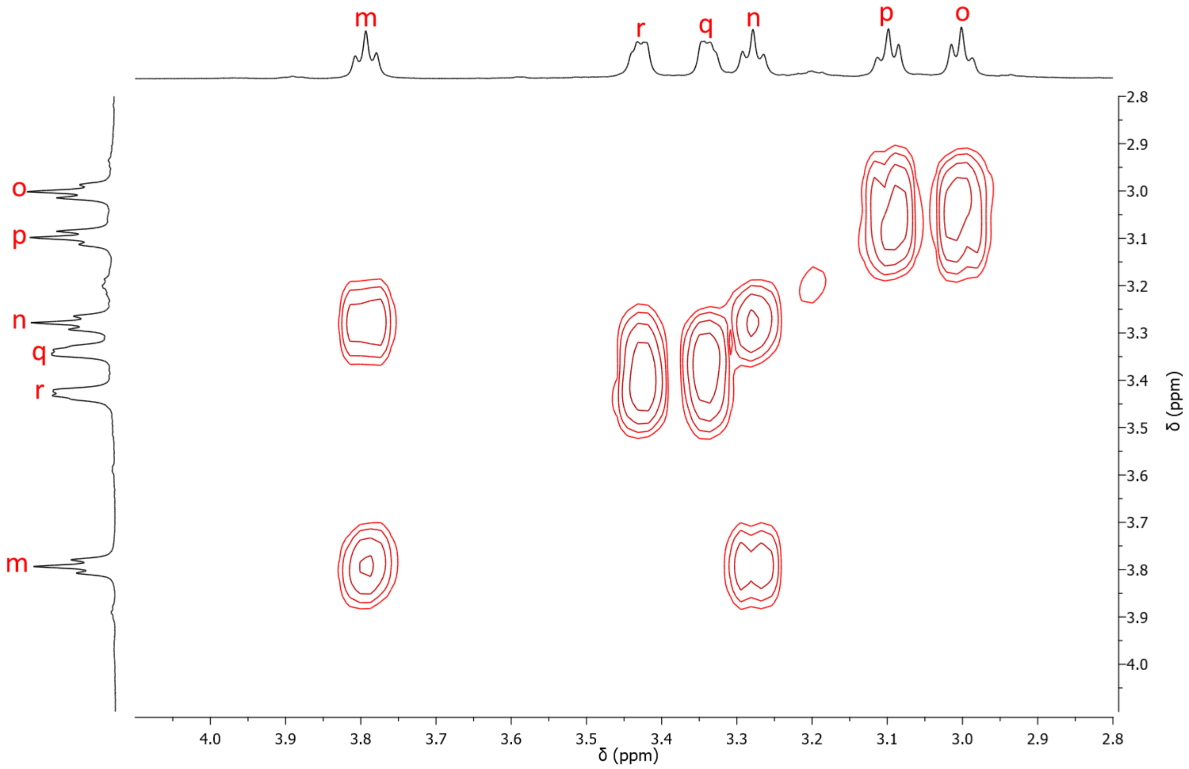

Figure S52. Partial ^{1}H-^{1}H COSY NMR spectrum (400 MHz, CD_2Cl_2) of compound **3-Pt^{II}**.^{H}H

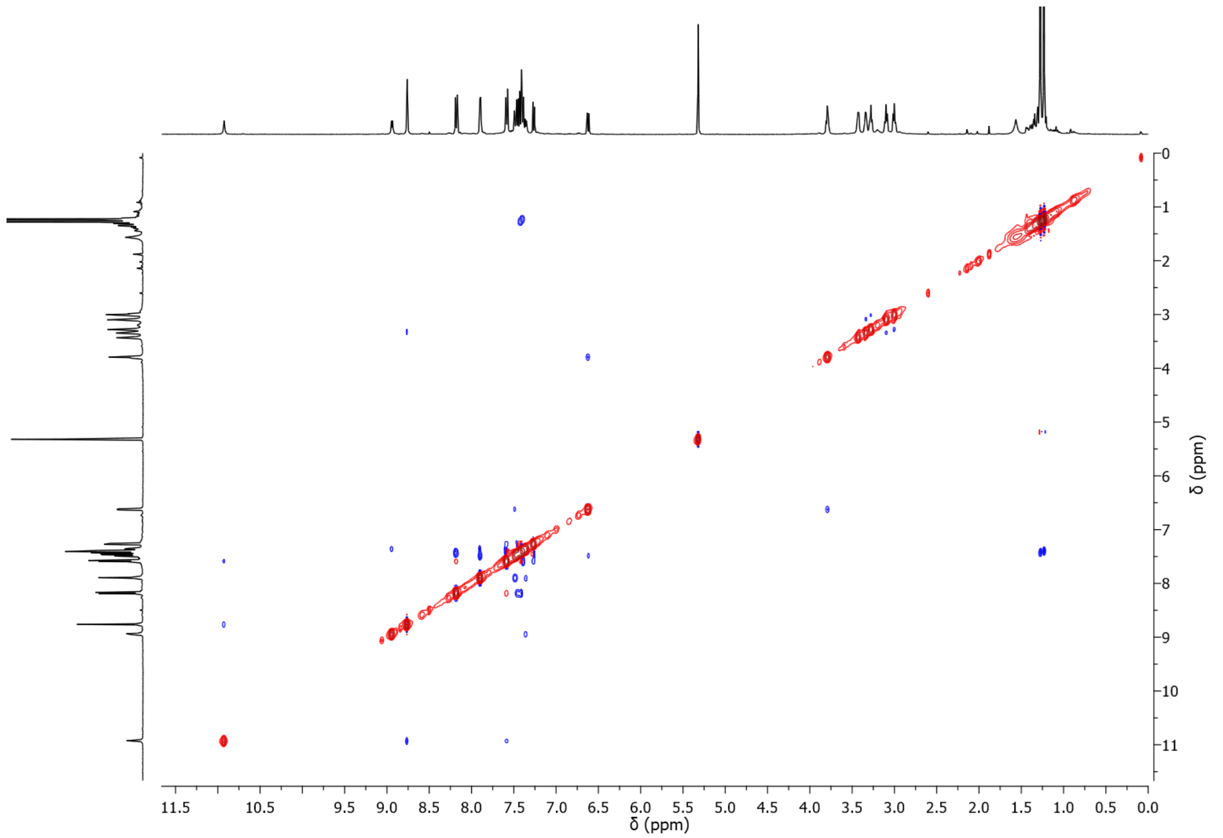

Figure S53. ^{1}H-^{1}H NOESY NMR spectrum (400 MHz, CD_2Cl_2) of compound **3-Pt^{II}**.

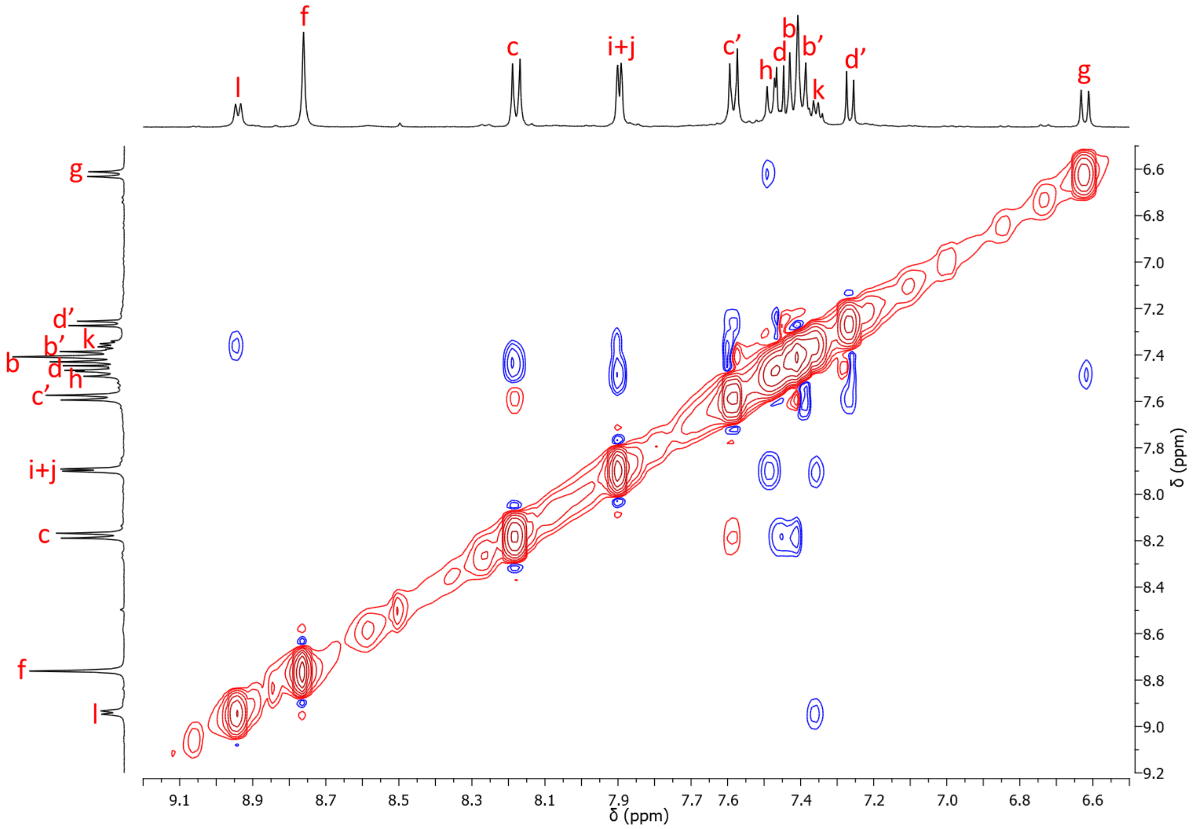

Figure S54. Partial ^{1}H-^{1}H NOESY NMR spectrum (400 MHz, CD_2Cl_2) of compound **3-Pt^{II}**.^{H}H

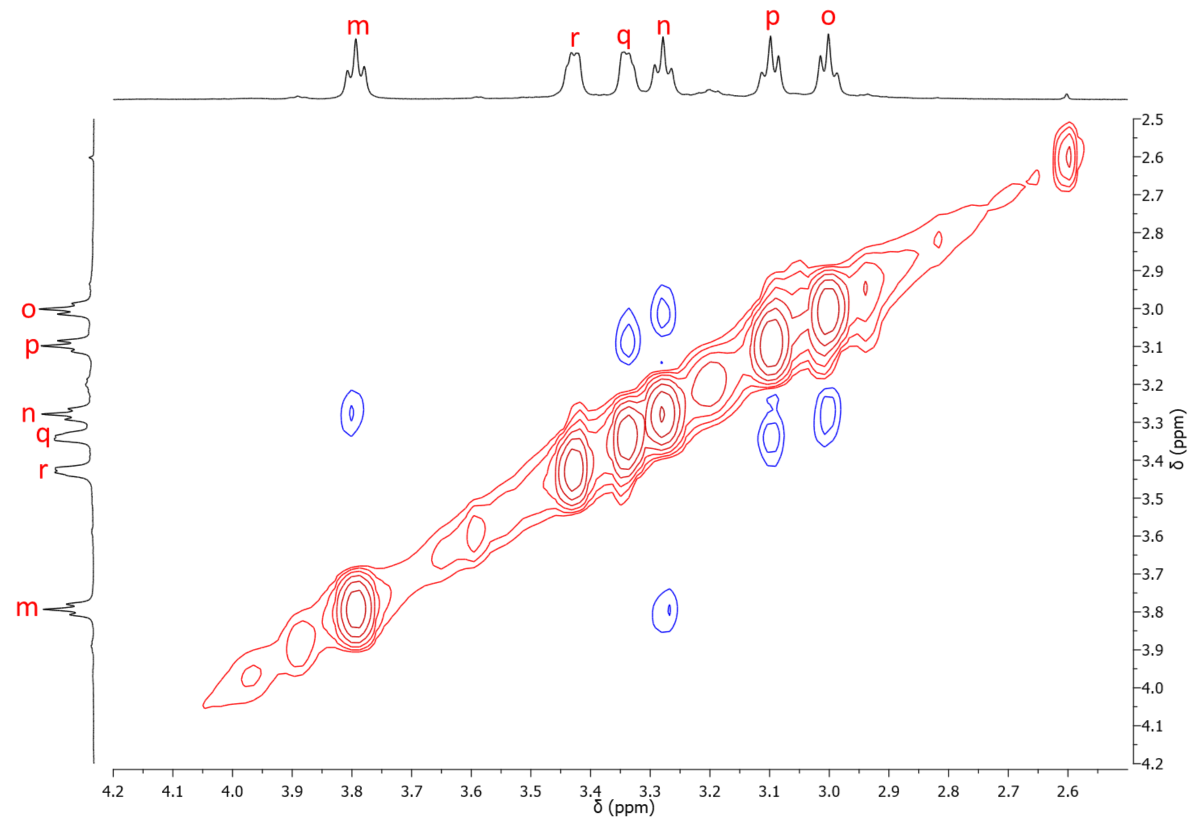

Figure S55. Partial ^{1}H-^{1}H NOESY NMR spectrum (400 MHz, CD_2Cl_2) of compound **3-Pt^{II}**.^{H}H

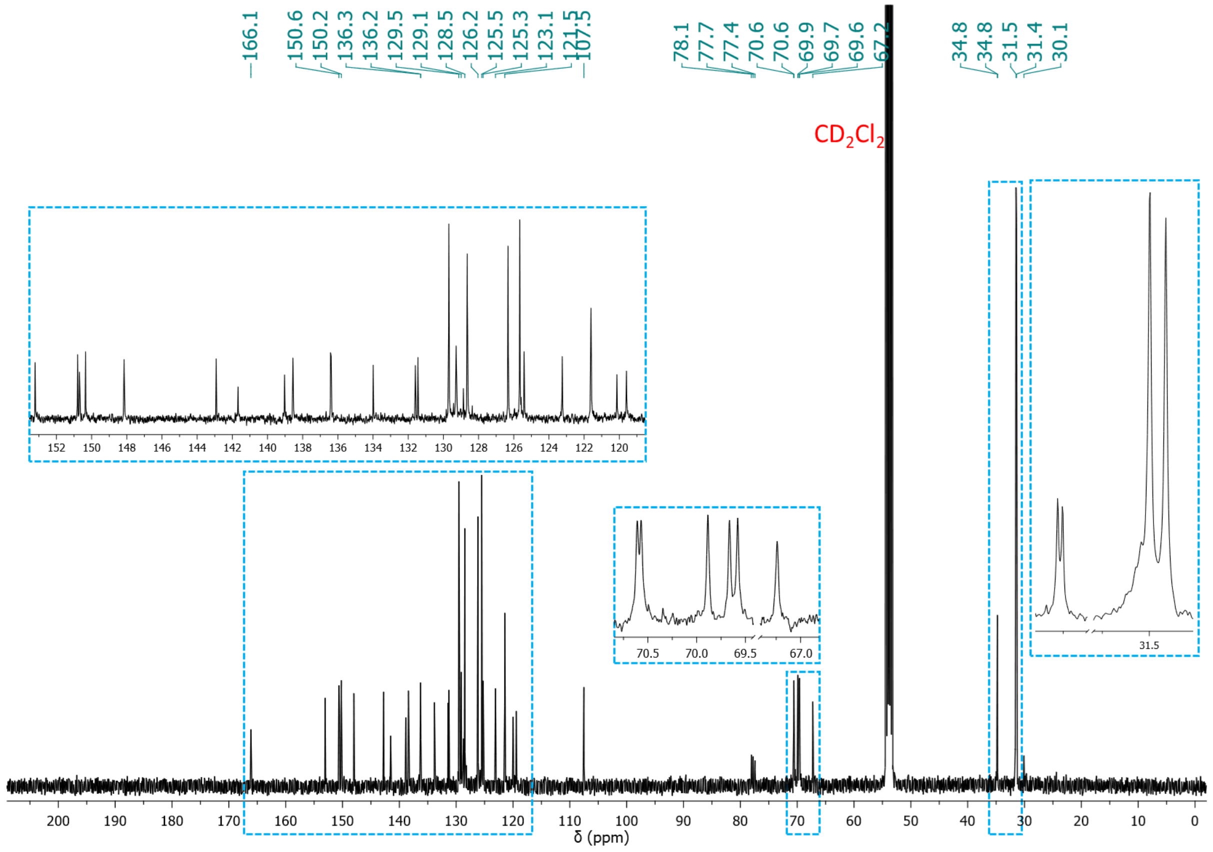

Figure S56. ^{13}C{^{1}H} NMR (101 MHz, CD_2Cl_2) spectrum of compound **3-Pt^{II}**.

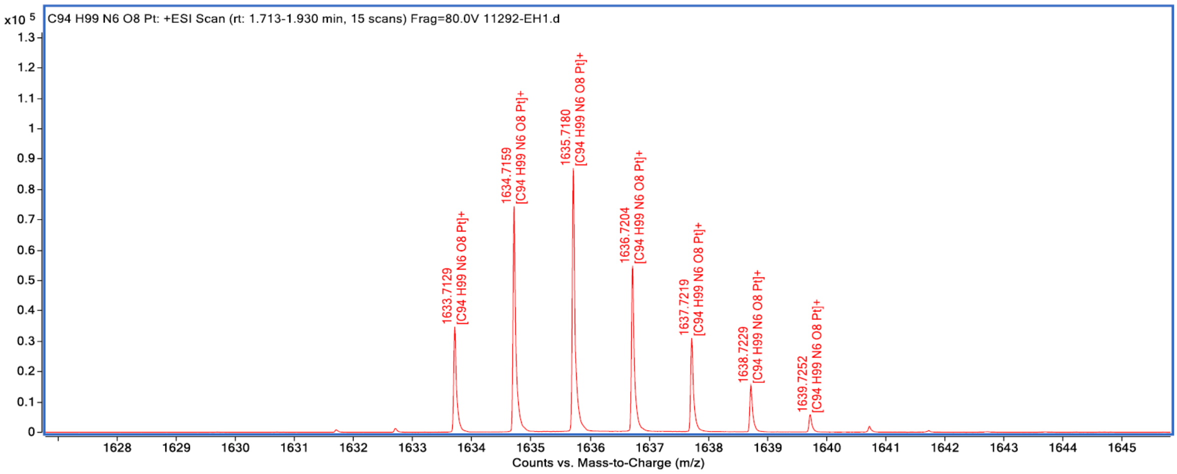

Figure S57. ESI-HRMS of compound **3-Pt^{II}**.

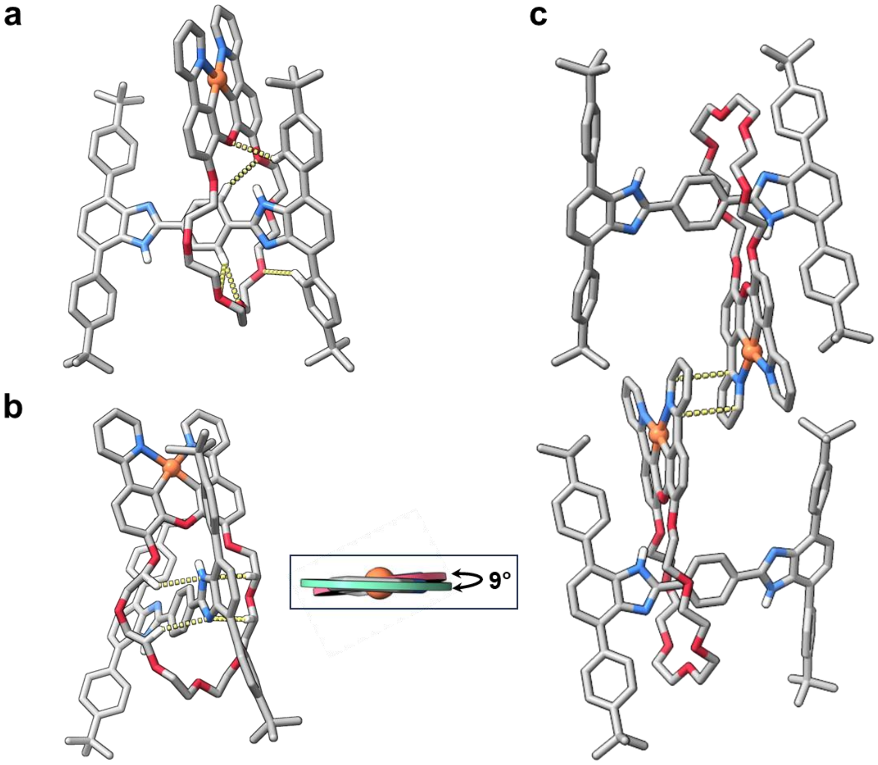

Figure S58. Selected views of the solid-state structures of the metalated [2]rotaxane **3-Pt^{II}** as determined by SCXRD. Dashed yellow lines highlight **a.** CH···O interactions, **b.** NH···N interactions (inset: dihedral angle between two 5-membered metallacycles showing deviation from planarity.) and **c.** π···π interactions between the phenyl rings in the macrocycles of two adjacent rotaxanes. Solvent molecules are omitted, and only relevant hydrogen atoms are shown for clarity.

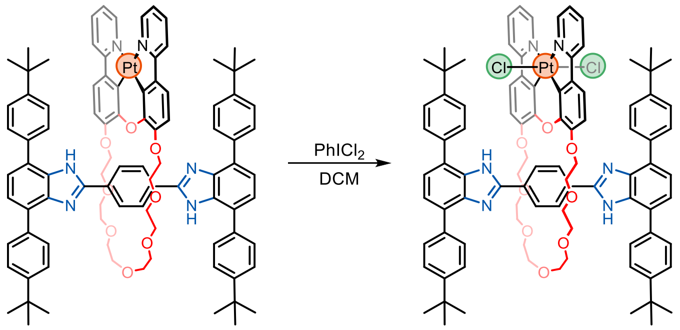

Scheme S4. Synthesis of Pt^{IV}-containing [2]rotaxane **3-Pt^{IV}**.

Compound 3-Pt^{IV}

The oxidation of **3-Pt^{II}** to **3-Pt^{IV}** was first tested on an NMR scale using a previously reported procedure.[16] The oxidant, $PhICl_2$ (37 µL of 4 × 10^{-2} M solution in CD_2Cl_2, *1*.48 × 10^{-3} mmol), was added to a solution of **3-Pt^{II}** (2.2. mg, 1.35 × 10^{-3} mmol) in DCM-*d*$_2$. Upon mixing for 30 s, the color changed to pale yellow. A recorded ^{1}H NMR spectrum of the reaction mixture showed the signals for **3-Pt^{II}** disappeared to give rise to new signals, which were ascribed to **3-Pt^{IV}**(Figure S55). After removing the solvent under vacuum, the crude product was washed with hexanes (3 × 1.5 mL). Then, it was dissolved in a minimum amount of DCM (approx. 0.5 mL) and triturated with methanol to give **3-Pt^{IV}** as a pale-yellow solid (1.8 mg, 1.06 × 10^{-3} mmol, 78%). For larger-scale reactions with 10-30 mg of the reactant, the mixtures were stirred between 10-15 min after the addition of $PhICl_2$; subsequent purification using the same procedure gave the product in comparable yields. **^{1}H NMR (400 MHz, CD_2Cl_2)**: δ 10.81 (*s*, 2H), 9.10 (*m*, 2H), 8.72 (*s*, 4H), 8.26-8.23 (*m*, 4H), 8.12-8.08 (*m*, 2H), 8.05-8.00 (*m*, 2H), 7.61-7.56 (*m*, 8H), 7.48-7.41 (*m*, 10H), 7.25 (*d*, *J* = 7.7 Hz, 2H), 6.69 (*d*, *J* = 8.5 Hz, 2H), 3.84 (*t*, *J* = 5.5 Hz, 4H), 3.36-3.25 (*m*, 12H), 3.07 (*t*, *J* = 5.0 Hz, 4H), 2.92 (*t*, *J* = 5.5 Hz, 4H), 1.26-1.24 (*m*, 36H). **ESI-HRMS:** [**3-Pt^{IV}** + H^+] *m/z* = 1705.6578 (experimental), *m/z* = 1705.6547 (calculated), relative error = 1.8 ppm. **UV-vis (DCM)**: λ_{max} = 282 nm (ε_{max} = 4.4 × 10^4 M^{-1} cm^{-1}); λ_{max} = 342 nm (ε_{max} = 4.6 × 10^4 M^{-1} cm^{-1}).

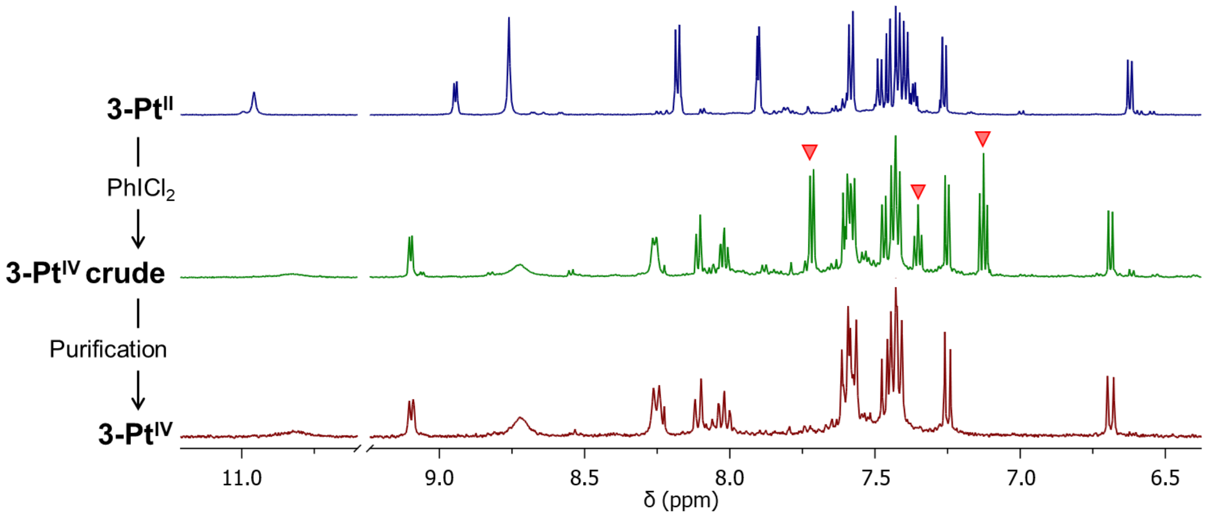

Figure S59. ^{1}H NMR spectra (400 MHz, CD_2Cl_2) of **a. 3-Pt^{II}**, **b. 3-Pt^{IV}** crude after the addition (ca. 1 min) of $PhICl_2$, and **c. 3-Pt^{IV}** after hexane washes and trituration with methanol. The signals highlighted by the red triangles belong to iodobenzene, which is a by-product of the reaction.

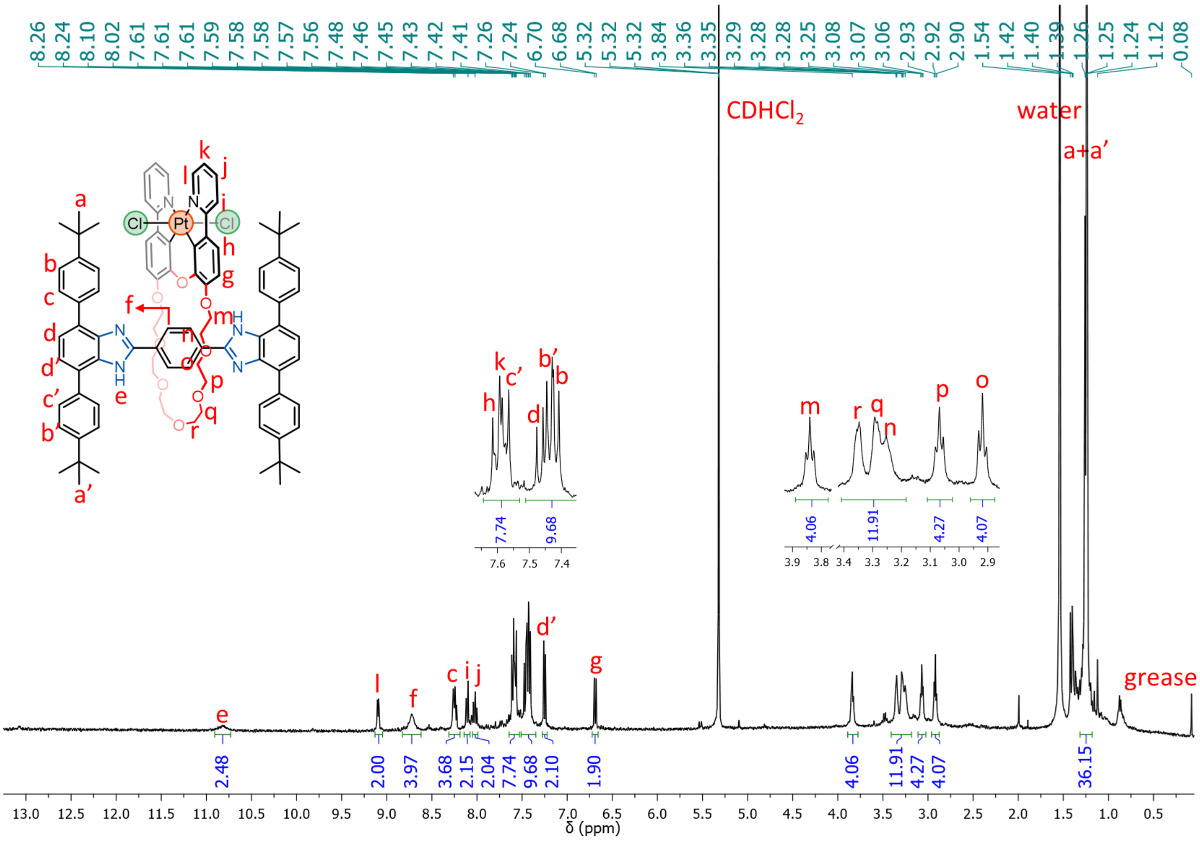

Figure S60. ^{1}H NMR spectrum (400 MHz, CD_2Cl_2) of compound **3-Pt^{IV}**.

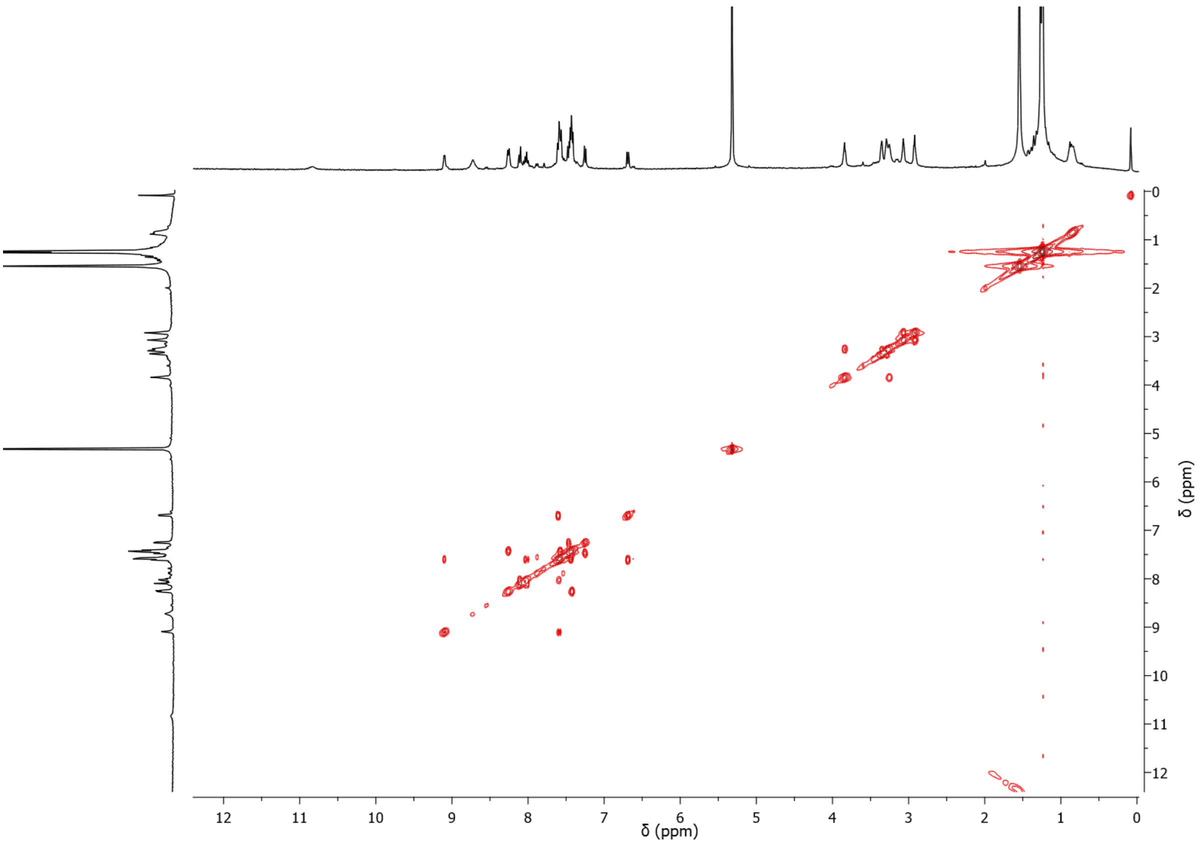

Figure S61. ^{1}H-^{1}H COSY NMR spectrum (400 MHz, CD_2Cl_2) of compound **3-Pt^{IV}**.

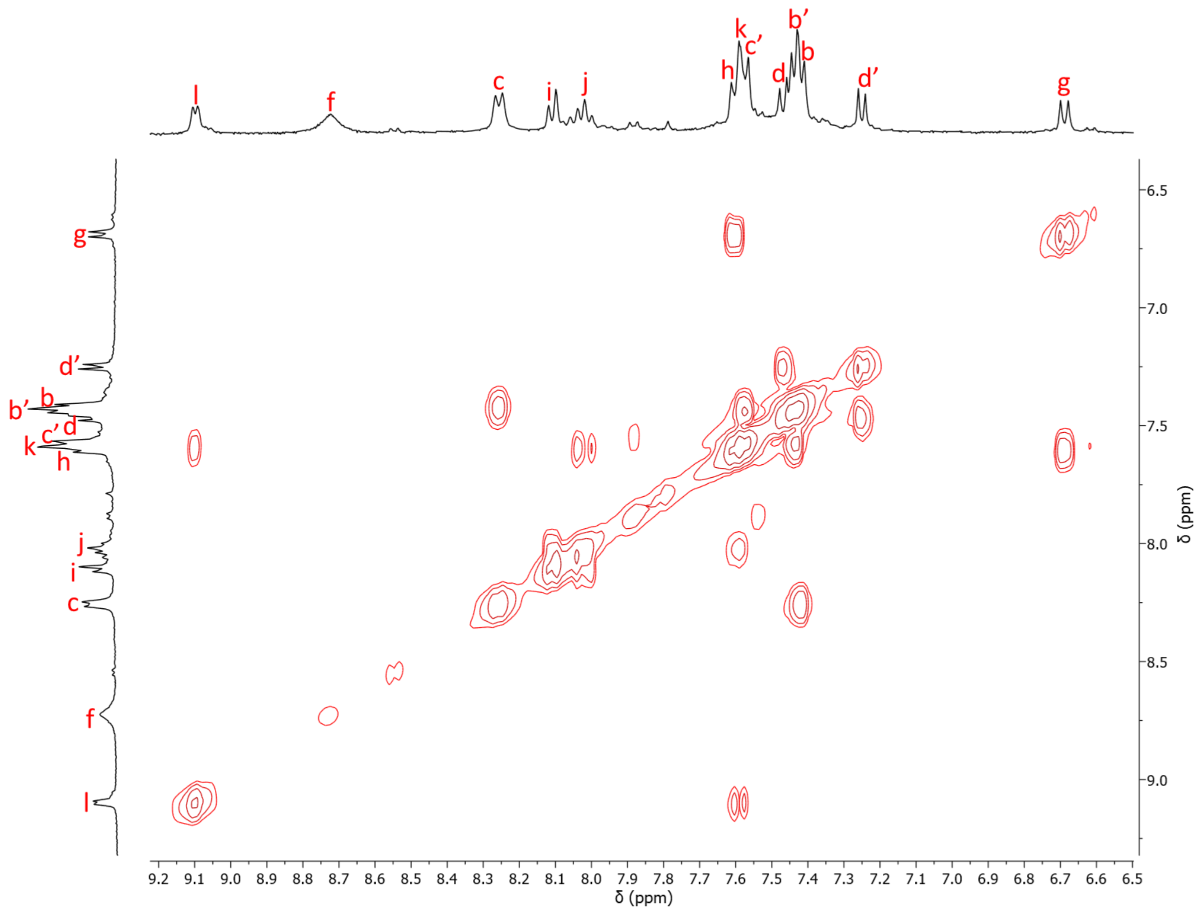

Figure S62. Partial ^{1}H-^{1}H COSY NMR spectrum (400 MHz, CD_2Cl_2) of compound **3-Pt^{IV}**.

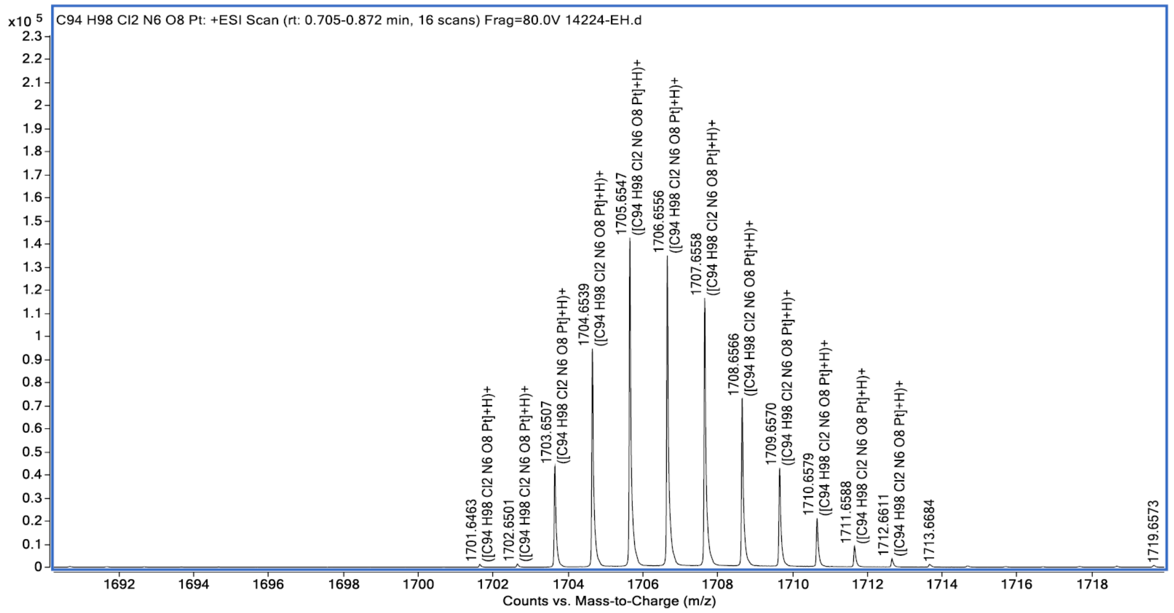

Figure S63. ESI-HRMS of compound **3-Pt^{IV}**.

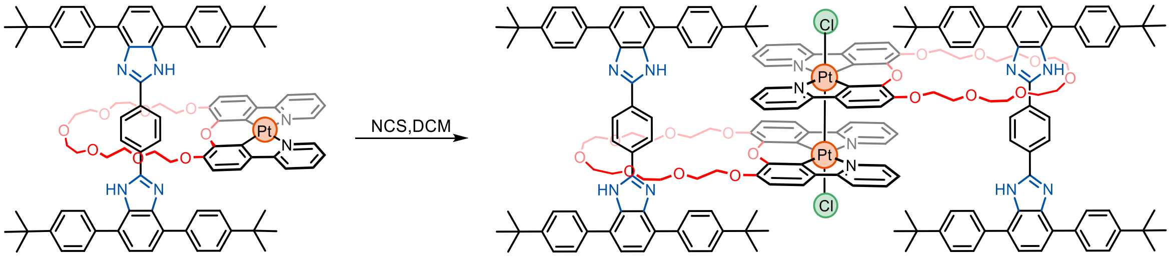

Scheme S5: Synthesis of Pt^{III}-containing [3]rotaxane **3-Pt^{III}**.

Compound 3-Pt^{III}

A solution of compound **3-Pt^{II}** (12.5 mg, 7.6 × 10^{-3} mmol) in DCM (1.5 mL) was treated with *N*-chlorosuccinimide (NCS) (180 μL of a 4.8 × 10^{-2} M solution in DCM, 8.41 × 10^{-3} mmol), and the mixture was stirred at room temperature for 20 min. The resulting orange solution was washed with saturated brine solution (3 × 3 mL) and dried over anhydrous $MgSO_4$. Removal of solvents under vacuum, followed by purification using size exclusion chromatography in DCM (Bio-beads S-X1, CH_2Cl_2, first fraction), yielded **3-Pt^{III}** as an orange solid after solvent removal (8.1 mg, 2.4 × 10^{-3} mmol, 63%). **^{1}H NMR (600 MHz, CD_2Cl_2)**: δ 12.23 (*s*, 2H), 10.71 (*s*, 2H), 9.32 (*s*, 4H), 8.26 (*d*, *J* = 8.3 Hz, 4H), 8.06 (*s*, 4H), 7.99 (*d*, *J* = 8.4 Hz, 4H), 7.75 – 7.74 (*m*, 4H), 7.71 (*d*, *J* = 8.2 Hz, 4H), 7.66 (*d*, *J* = 8.4 Hz, 4H), 7.62 (*d*, *J* = 8.3 Hz, 4H), 7.55 (*d*, *J* = 7.5 Hz, 2H), 7.52 (*d*, *J* = 8.4 Hz, 4H), 7.35 (*d*, *J* = 7.5 Hz, 2H), 7.30 (*d*, *J* = 7.6 Hz, 2H), 7.24 – 7.20 (*m*, 8H), 7.16-7.14 (*m*, 8H), 7.08 (*d*, *J* = 7.6 Hz, 2H), 6.95 (*d*, *J* = 8.5 Hz, 4H), 6.71 – 6.69 (*m*, 4H), 6.45 (*d*, *J* = 8.4 Hz, 4H), 4.20 – 4.13 (*m*, 4H), 3.54 – 3.45 (*m*, 6H), 3.33 – 3.31 (*m*, 2H), 3.17 – 3.13 (*m*, 2H), 3.06 – 3.02 (*m*, 2H), 2.95 – 2.88 (*m*, 4H), 2.81 – 2.77 (*m*, 2H), 1.45 (*s*, 18H), 1.44 (*s*, 18H), 1.38 (*s*, 18H), 0.89 (*s*, 18H). **^{13}C{^{1}H} NMR (151 MHz, CD_2Cl_2)**: δ 161.7, 155.8, 152.7, 150.7, 150.57, 150.55, 150.3, 149.6, 146.8, 143.3, 142.6, 139.8, 138.53, 138.51, 137.7, 137.3, 136.6, 135.9, 135.7, 134.3, 133.8, 132.0, 131.7, 131.0, 130.7, 130.4, 129.5, 129.4, 128.0, 126.5, 126.1, 125.9, 125.8, 125.6, 125.5, 123.42, 123.35, 122.0, 121.7, 121.5, 121.4, 120.0, 118.8, 118.7, 108.2, 71.0, 70.6, 69.70, 69.69, 69.5, 66.9, 35.1, 35.0, 34.9, 34.7, 31.9, 31.7, 31.6, 31.4. **MALDI-TOF (matrix: dctb):** [**3-Pt^{III}**-Cl^{-}]$^{+}$ *m/z* = 3303.38(experimental), 3303.39(calculated), relative error = -3.0 ppm; [**3-Pt^{III}**-2Cl^{-}]$^{2+}$ *m/z* = 3268.41 (experimental), 3268.42 (calculated), relative error = -3.0 ppm. **UV-vis (DCM)**: λ_{max} = 285 nm (ε_{max} = 12.5 × 10^{4} M^{-1} cm^{-1}); λ_{max} = 334 nm (ε_{max} = 12.7 × 10^{4} M^{-1} cm^{-1}); λ_{max} = 457 nm (ε_{max} = 0.3 × 10^{4} M^{-1} cm^{-1}).

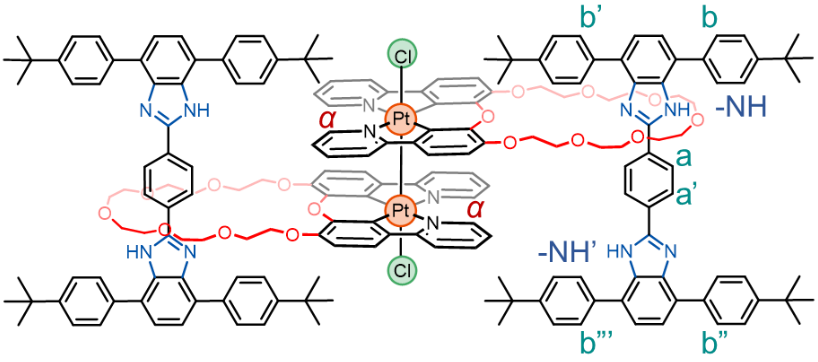

3-Pt^{III}

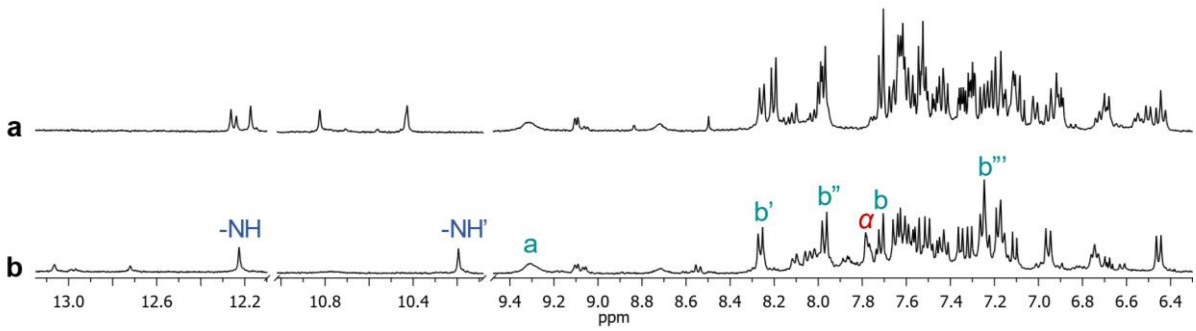

Figure S64. ^{1}H NMR spectra (400 MHz, CD_2Cl_2) of **3-Pt^{II}** **a.** after adding NCS and **b.** following brine-wash. Only selected protons are labelled for clarity.

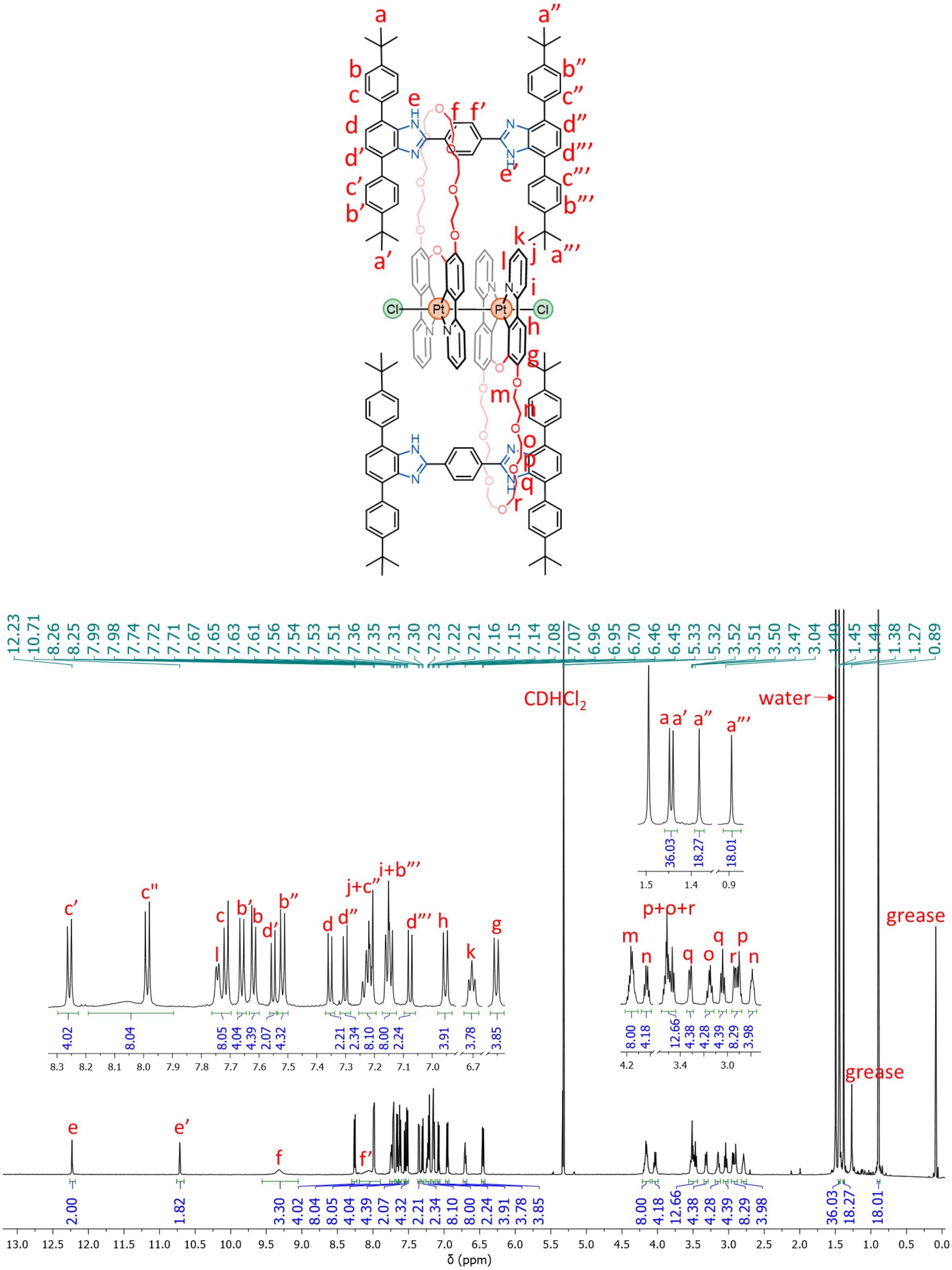

Figure S65. ^{1}H NMR spectrum (600 MHz, CD_2Cl_2) of compound **3-Pt^{III}**.

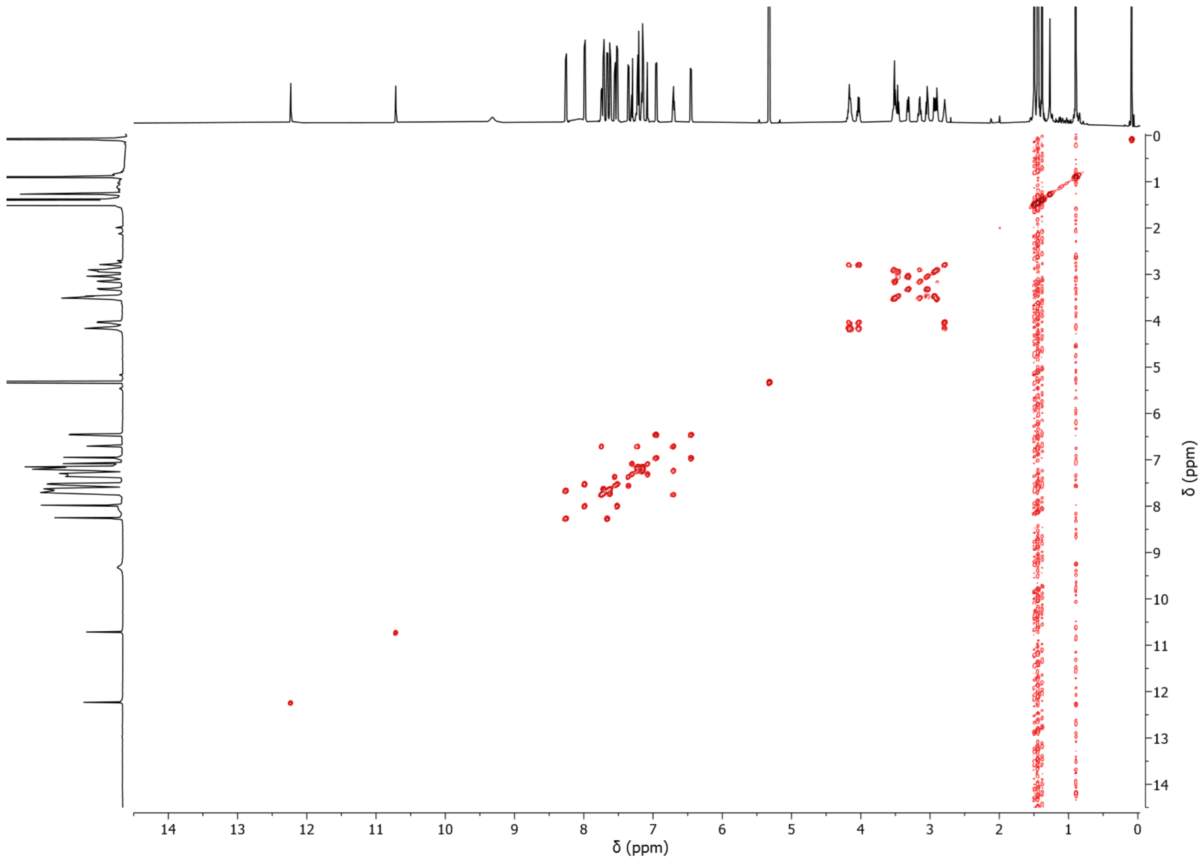

Figure S66. ^1H-^1H COSY NMR spectrum (600 MHz, CD_2Cl_2) of compound **3-Pt^{III}**.

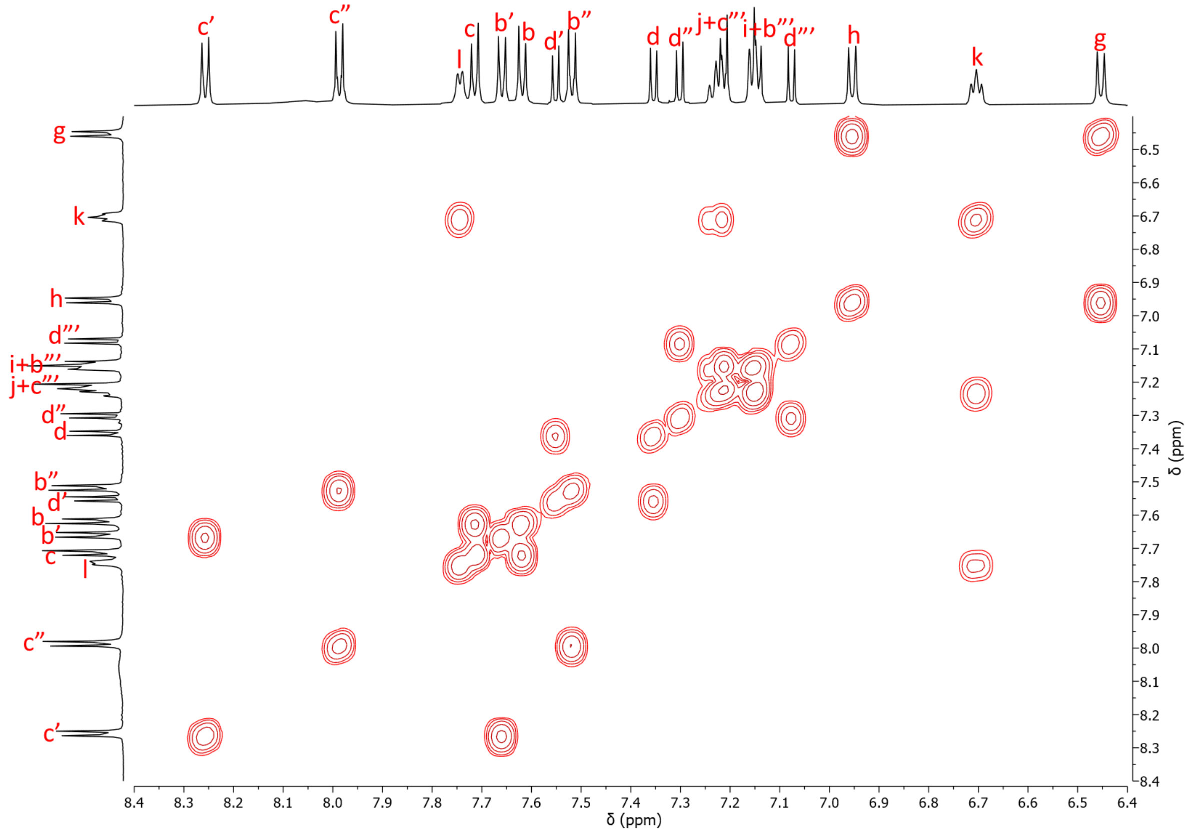

Figure S67. Partial ^1H-^1H COSY NMR spectrum (600 MHz, CD_2Cl_2) of compound **3-Pt^{III}**.

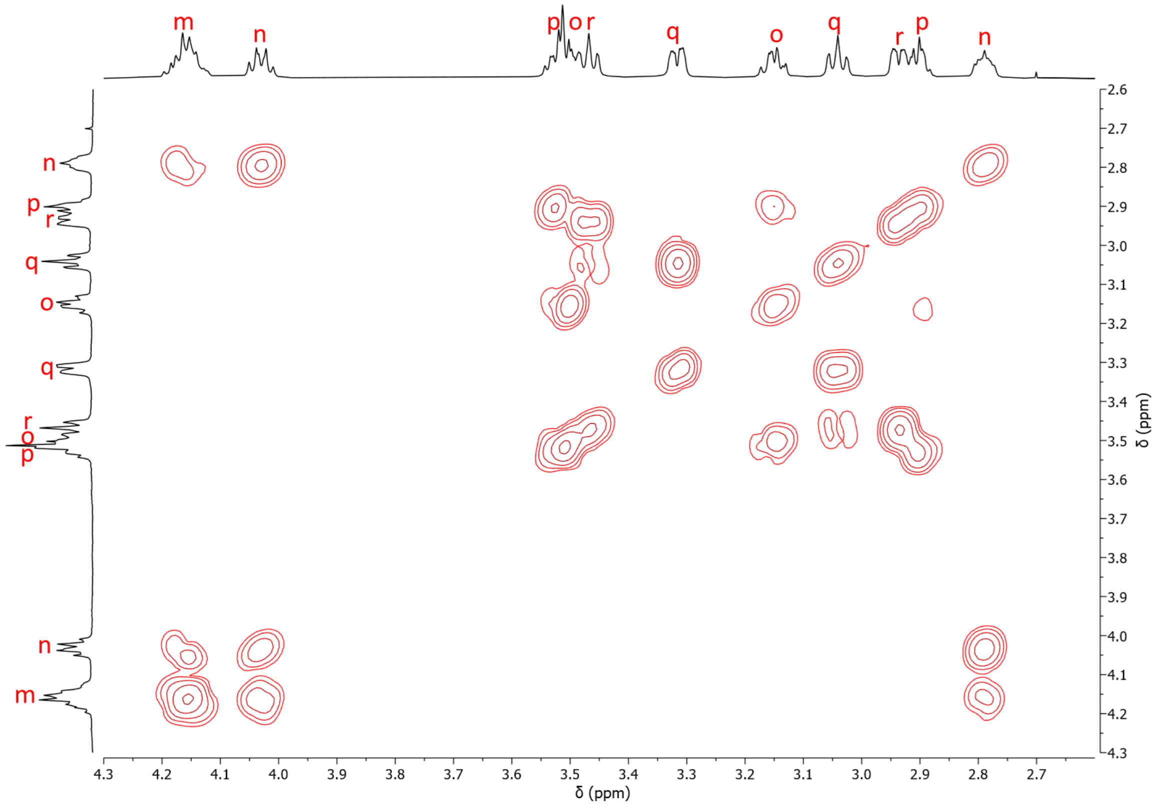

Figure S68. Partial ^{1}H-^{1}H COSY NMR spectrum (600 MHz, CD_2Cl_2) of compound **3-Pt^{III}**.

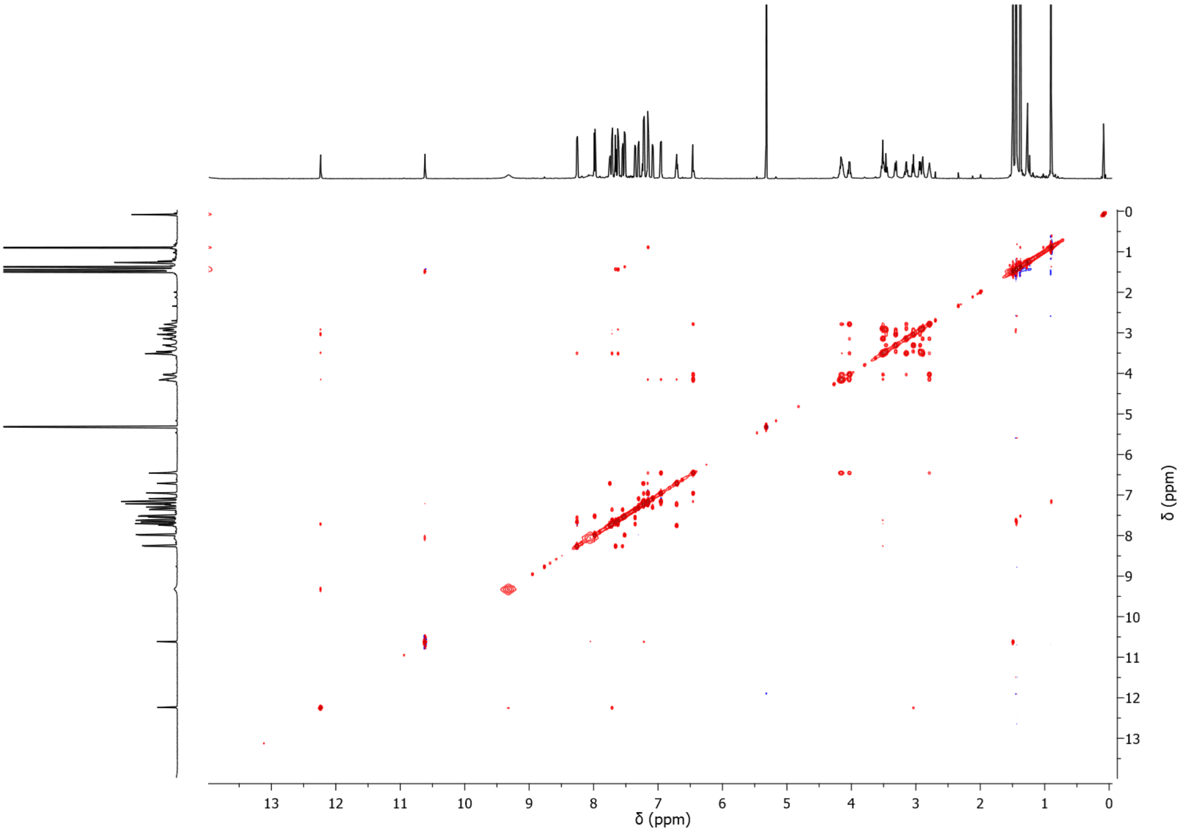

Figure S69. ^{1}H-^{1}H NOESY NMR spectrum (600 MHz, CD_2Cl_2) of compound **3-Pt^{III}**.

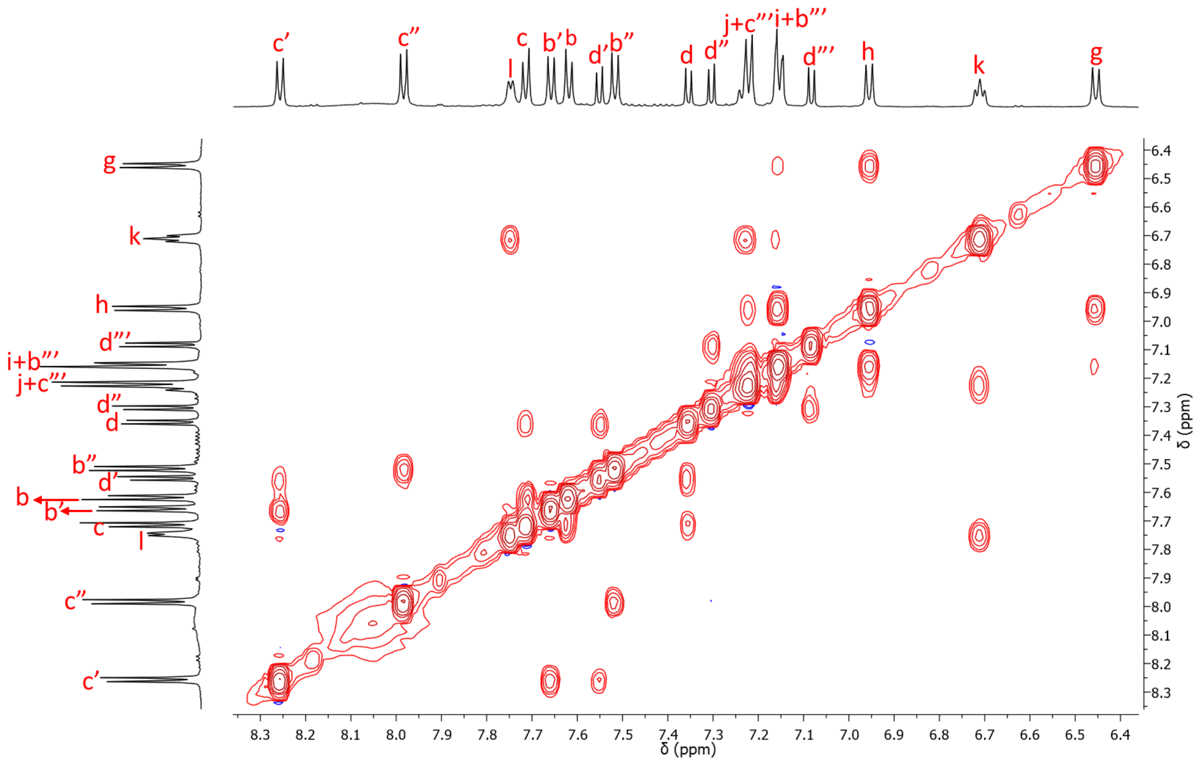

Figure S70. Partial ^{1}H-^{1}H NOESY NMR spectrum (600 MHz, CD_2Cl_2) of compound **3-Pt^{III}**.

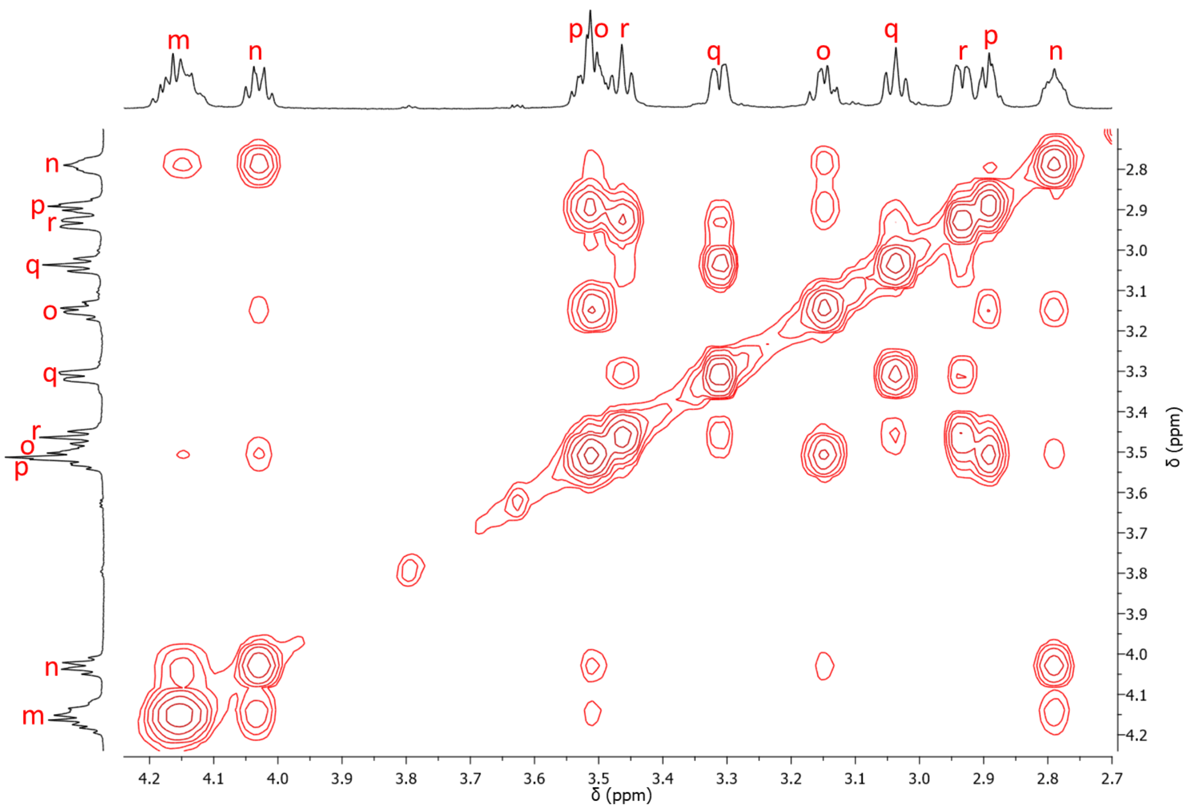

Figure S71. Partial ^{1}H-^{1}H NOESY NMR spectrum (600 MHz, CD_2Cl_2) of compound **3-Pt^{III}**.

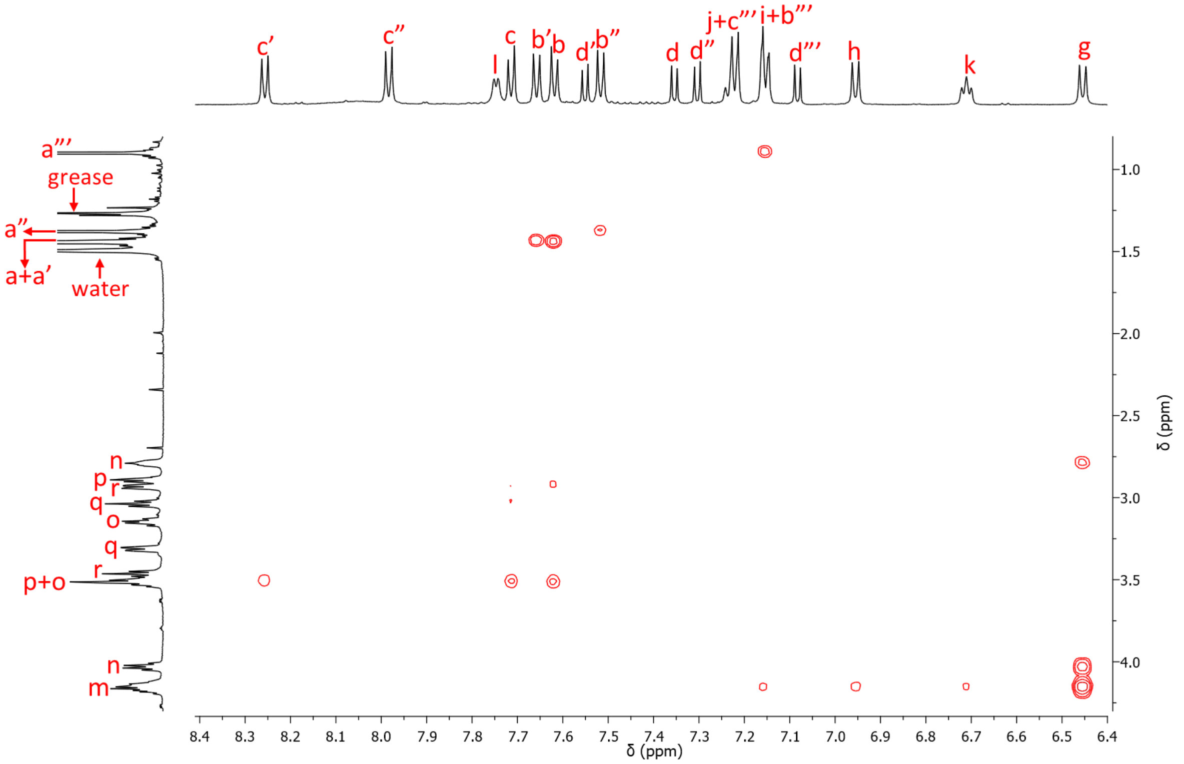

Figure S72. Partial ^{1}H-^{1}H NOESY NMR spectrum (600 MHz, CD_2Cl_2) of compound **3-Pt^{III}**.

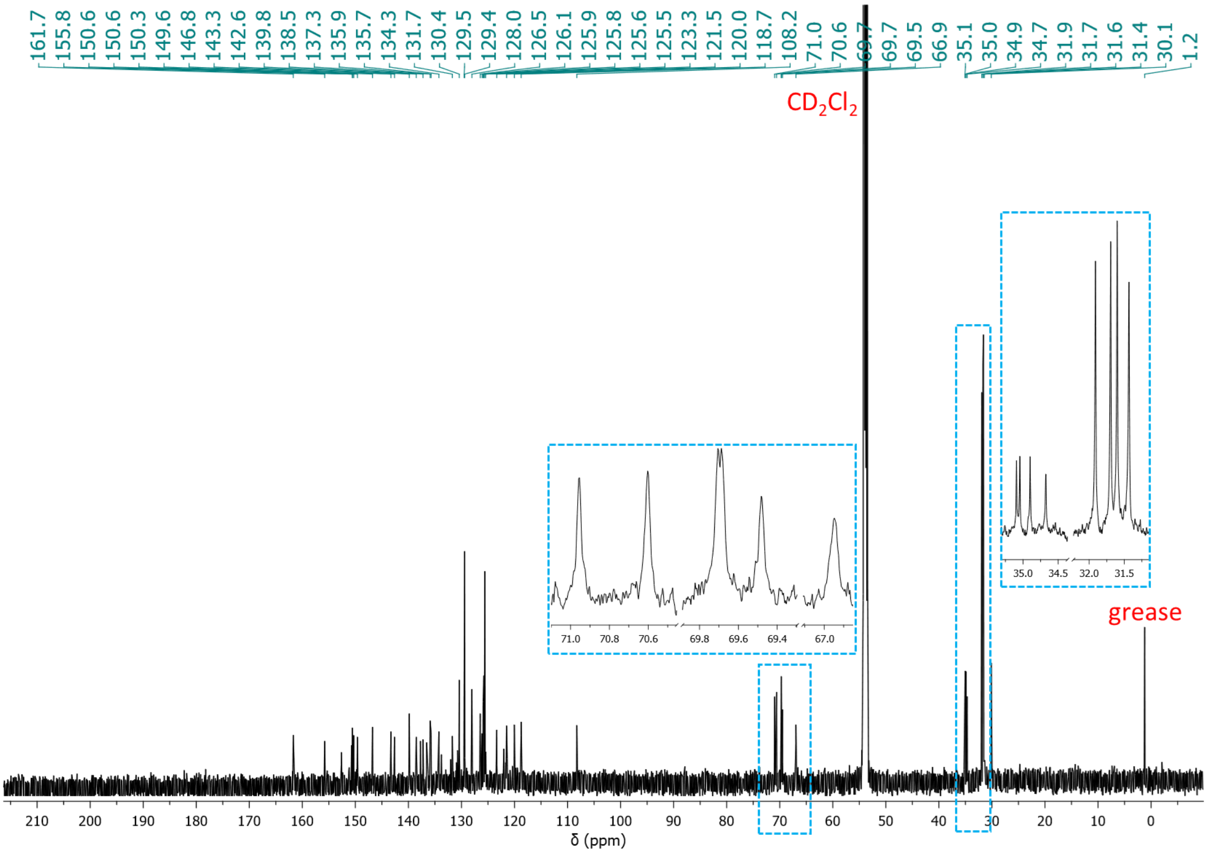

Figure S73. $^{13}C\{^{1}H\}$ NMR spectrum (151 MHz, CD_2Cl_2) of compound **3-Pt^{III}**.

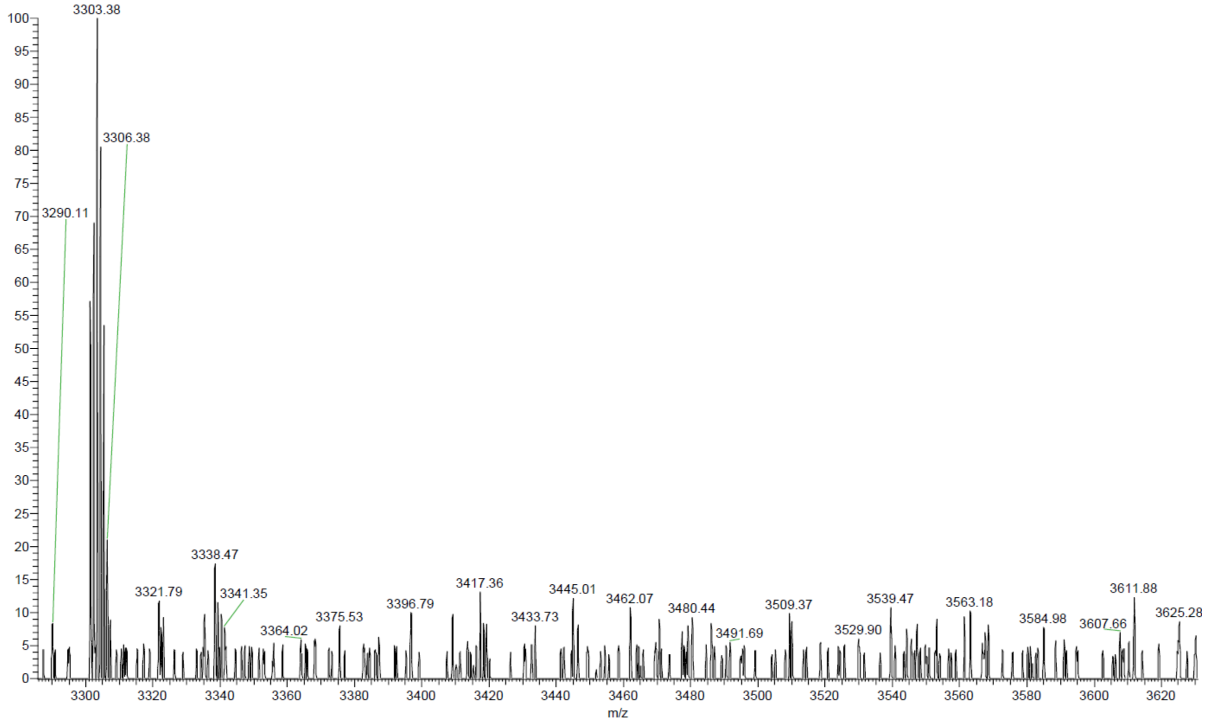

Figure S74. MALDI-TOF mass spectrum of compound **3-Pt^{III}** showing [**3-Pt^{III}**-Cl^{-}]$^{+}$ peak at *m/z* = 3303.38 (calc. *m/z* = 3303.39).

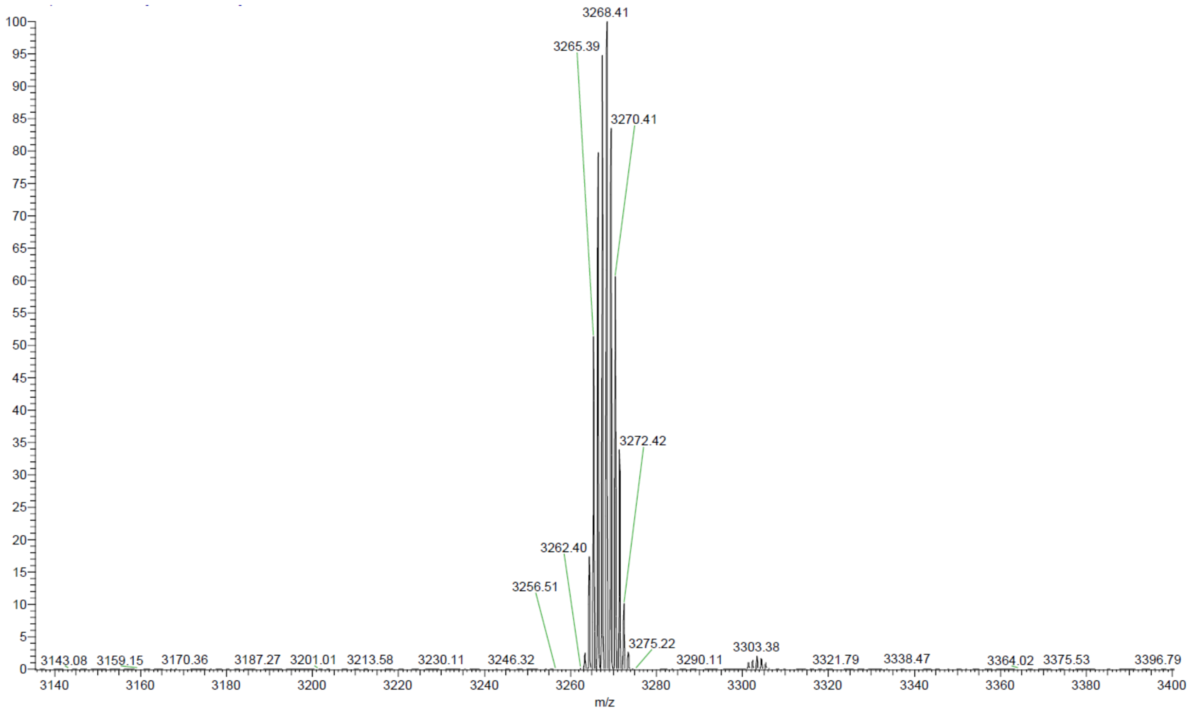

Figure S75. MALDI-TOF mass spectrum of compound **3-Pt^{III}** showing [**3-Pt^{III}**-2Cl^{-}]$^{2+}$ peak at *m/z* = 3268.41 (calc. *m/z* = 3268.42).

Computational data

Comparison between computed structures of the rotaxanes

3-Pt^{II}

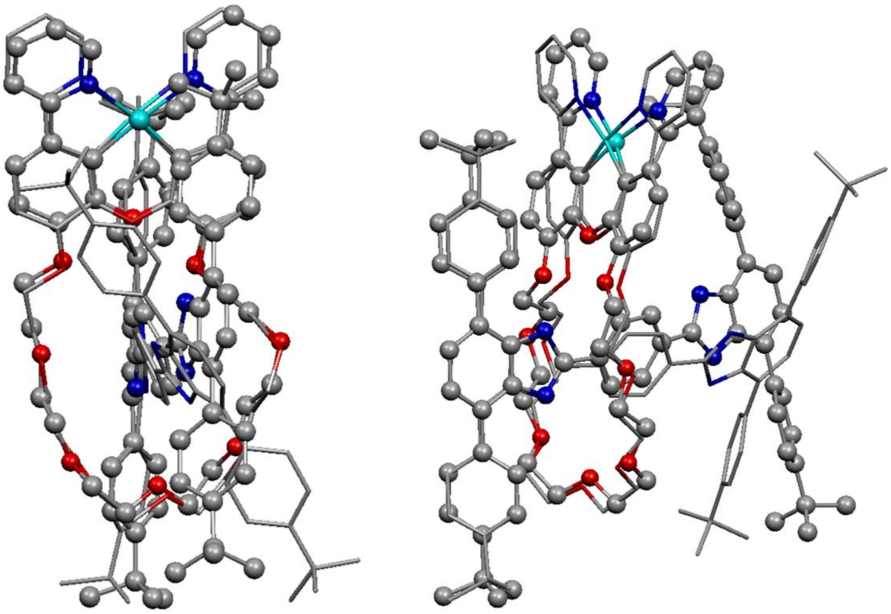

Figure S76. Solid-state molecular structure (as determined by SCXRD) and the computed structure for **3-Pt^{II}** are overlayed in capped stick, and ball and stick styles, respectively. All hydrogen atoms have been omitted for clarity.

The comparison shows that whereas in the **3-Pt^{II}** computed structure at the 6-31g(d);cc-pVDZ,ECP60MDF/ωB97XD/Acetone, the Pt complex, the first stopper and the central phenyl ring are almost parallel to the farther stopper with geometries very close to the SCXRD structure, the second stopper is tilted in a different way (Figure S73). The torsion along the axle between the phenyl and the two benzimidazoles are 21° and -7° in the former and 18.4° and 11° in the latter, respectively, indicating that the stopper rotates in the other direction in the computed structure.

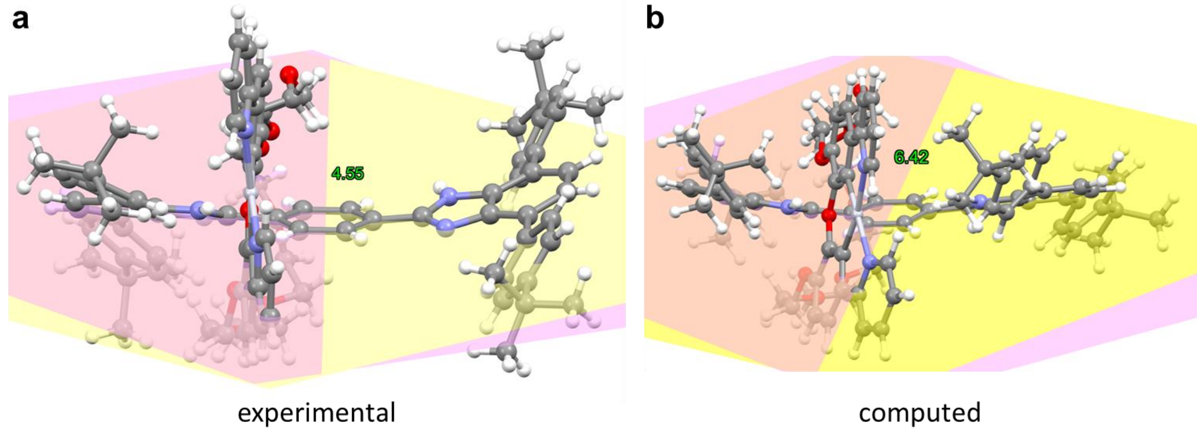

Figure S77. Angles between the three carbon atoms on each side of the axle are highlighted in **a.** the

solid-state molecular structure (as determined by SCXRD) and **b.** the computed structure (right) for **3-Pt^{II}**.

The central benzene unit has a boat conformation, and the angles between the three carbon atoms on each side of the axle are 4.6° and 6.4°, respectively, for the experimental and the computed structures (Figure S77).

It is worth noting that the SCXRD structure of **3-Pt^{II}** shows an H-bond with the NH of the axle at 2.838 Å compared to 2.644 Å for the computed one. Furthermore, calculations indicate that the structure with bridging oxygen H-bond interaction is less stable than with glycolic oxygen by 1.5 and 2.5 kcal/mol at the 6-31g; cc-pVDZPP, ECP60MDF/PBE1PBE/Acetone and 6-31g(d); cc-pVDZ-PP, ECP60MDF/acetone level of theory respectively. This finding suggests that the molecule can easily switch between the two H-bond modes, changing the environment or the coordination of the Pt atom as in the case of **3-Pt^{IV}**.

Table S1. Cartesian coordinates of **3-Pt^{II}** at the 6-31g(d); cc-pVDZ-PP, ECP60MDF/ωB97XD/Acetone level of theory.

	H 11.5760642539 13.8341820543 4.5951868299
Pt 14.0732320433 16.2168507362 10.4193627194	H 12.4494921573 15.270473314 4.0468310991
O 10.9280199212 15.1299467523 10.9637968775	H 10.6934334016 15.1846563611 3.8482456307
O 10.0389970072 12.7028260993 10.6424970748	C 12.5813960524 15.189920365 6.7600910264
O 9.4535309398 9.2878108757 11.8501821386	H 12.5696334387 15.6976297318 7.7286186629
O 6.2374491306 9.2479651085 13.5351965552	H 13.5170405464 15.4580586414 6.2536861508
O 4.7575478564 11.0598413598 15.1445907583	H 12.5928281215 14.1114843291 6.9554567681
O 4.4670761836 13.8789436739 15.4376814593	C 11.368284415 17.1154066496 5.699522064
O 6.5392773048 16.0744680132 13.5612230844	H 10.5149208716 17.4300508577 5.0880762918
O 8.8389786079 16.607571928 11.6700438846	H 12.2877174356 17.4328834979 5.1936440718
N 15.6674194715 15.1338724333 9.4582949812	H 11.3148355979 17.6452823243 6.6564066624
N 14.8720859611 18.1528712454 10.9300949518	C 7.1084015636 13.2721517256 12.2228481466
C 16.860197174 15.5929584677 9.0537111866	C 7.987889878 13.1428428389 13.3875313198
H 17.0634170429 16.6372917255 9.248855047	C 7.7090887628 12.2077221455 14.3903127484
C 17.7926458369 14.8083813772 8.398512904	H 6.7854089692 11.6377857512 14.361464752
H 18.7418157012 15.2340849281 8.0962114081	C 8.6302269076 11.9885202253 15.4084427605
C 17.4665674731 13.4790550388 8.1429529856	H 8.4357610755 11.2369252971 16.1671086759
H 18.167318935 12.8244683717 7.6354286581	C 9.8360300828 12.6978166069 15.4370770262
C 16.2250895615 13.0029892052 8.5314526185	C 10.0698051317 13.6846202653 14.4731142842
H 15.9504260011 11.9772466008 8.3191349161	C 9.1545317158 13.9063059493 13.4601484364
C 15.3246947867 13.8493510681 9.1842081489	C 10.8745095099 12.3920472403 16.4263768845
C 13.9628138737 13.4701552853 9.5820178969	C 7.4733308522 9.693425962 23.5183751674
C 13.4533051292 12.1934557806 9.3930365229	H 6.8977626724 9.7165890014 22.5862350574
H 14.054560488 11.391675477 8.9776715434	H 7.6574620908 10.725607961 23.8369102886
C 12.1406298535 11.9181821486 9.7486008969	H 6.8493872357 9.2163646322 24.2820503546
H 11.7610889907 10.9147962246 9.6081501864	C 9.5004836005 8.8868200541 24.7228321575
C 11.3295262156 12.9170190765 10.2816738561	H 8.8492846386 8.4484642458 25.4882408813
C 11.839567914 14.2250323696 10.4601547159	H 9.7624906045 9.9031845724 25.0377450962
C 13.1638633168 14.5038111709 10.1343302201	H 10.4210151934 8.2948857306 24.688365313
C 9.9997727186 10.5917386921 11.8659667708	C 8.4271319455 7.4627620258 22.948607655
H 11.0918828202 10.5029803419 11.851551523	H 9.3189634648 6.8324736962 22.869236767
H 9.7226713478 11.134569698 12.7764247149	H 7.915382752 7.4496699061 21.9797465542
C 8.4883390163 9.0118962722 12.8472955505	H 7.7621292452 7.0096384281 23.6933004973
H 8.7510123449 9.5017753775 13.7944468625	C 8.7809917689 8.9050871158 23.3602364667
H 8.5061859613 7.9300075647 13.0093546889	C 9.7076167632 9.5208861435 22.3049184053
C 4.8984404821 9.6231261445 13.2718970219	C 9.3759591593 10.6641084114 21.5728277615
H 4.3740928496 8.8208337978 12.7291011718	H 8.4288089373 11.1655134706 21.7398201337
H 4.8719862033 10.5364167376 12.6645159855	C 10.2392657451 11.1961295621 20.6180883408
C 4.1887124619 9.9045714594 14.577336926	H 9.9447264126 12.0830135578 20.067535937

H	4.2744466157	9.0476075331	15.2643433583
H	3.1230898956	10.06471238	14.360612908
C	4.0056531716	11.6569816463	16.1697243451
H	3.9786775913	11.0193621581	17.0677723346
H	2.966866916	11.8231808357	15.8430215279
C	4.652300251	12.9841063934	16.5123089781
H	4.2121443196	13.3866266119	17.4371638492
H	5.7240731109	12.8154262252	16.6914986693
C	5.4886586544	14.84244653	15.3069716565
H	6.4746293638	14.3598219383	15.3090900688
H	5.4616823647	15.5648614831	16.1371072738
C	5.2980999492	15.5309437772	13.9628767943
H	4.9639626206	14.7750062089	13.2427437972
H	4.5349440027	16.3227650911	14.018994634
C	6.471829376	16.6550409208	12.2808746445
H	5.6375524538	17.373478279	12.2358171742
H	6.2971023469	15.8971963267	11.506313976
C	7.729391451	17.4428145926	12.005669599
H	7.5415325309	18.1333867089	11.1748008476
H	7.9612242495	18.0233210039	12.9051086715
C	12.42732035	17.0753722576	11.0641602115
C	11.1754244331	16.4697786446	11.1701350422
C	10.0380436179	17.2410128426	11.5291906471
C	10.1786114426	18.6155511972	11.7248093611
H	9.3237386847	19.2319818562	11.9620479709
C	11.4214193634	19.2260978767	11.6296709572
H	11.4844567075	20.2957215473	11.8007851013
C	12.5388295798	18.466234839	11.322224435
C	13.8962625985	19.0295158884	11.2792148204
C	14.2195561652	20.3456023987	11.6193807692
H	13.4378701486	21.0448698209	11.8892027885
C	15.5442955551	20.7522277167	11.6316968479
H	15.7988185961	21.7729296409	11.897312315
C	16.5393013222	19.8294146056	11.3214253701
H	17.5903832931	20.090595052	11.3438858106
C	16.1529375574	18.5455263651	10.9802810262
H	16.8942236208	17.7903175641	10.7560785232
N	5.8678521051	12.8470926764	12.1379786632
H	8.5367329383	14.0081016129	10.8465864686
N	7.5648564211	13.7961973913	11.0464258797
N	10.7078455103	11.8310104195	17.6029869153
N	12.1862692627	12.678523498	16.1599454839
H	12.5631042497	12.9854830256	15.2755881176
C	-1.2581866614	11.4820004004	13.3696395205
H	-0.7957712968	12.2390582025	14.0115404392
H	-2.1161108354	11.0637546065	13.9091474918
H	-1.6310948423	11.9828579685	12.4692052947
C	0.2368158237	9.6812427386	14.2880805895
H	0.9472019176	8.8801812362	14.0532454332
H	-0.6044870404	9.241112752	14.8361277177
H	0.7345038508	10.3910790541	14.9576381548
C	-1.0108738295	9.3140675065	12.1637633518
H	-1.423208954	9.7462464806	11.2451516127
H	-1.8469206804	8.9096990873	12.7447143881
H	-0.3601276801	8.4759989622	11.8899380155
C	-0.2652004237	10.3627414522	13.000657093
C	0.9223022264	10.9714971311	12.2456009765
C	1.7258696446	11.9384984134	12.8665132306
H	1.5051081328	12.2549120581	13.8827142024
C	2.8098492122	12.5179862646	12.2231000562
H	3.4173312017	13.2373251142	12.7602455837
C	3.1351604498	12.1642922934	10.9054525683
C	2.3421663497	11.1974110329	10.282134228
H	2.5818740058	10.8762260107	9.2720319257
C	1.2608994403	10.6130582471	10.9388108132
H	0.6857254882	9.8619807711	10.4084551107
C	11.4781125777	10.6045509861	20.3566082156
C	11.8149963633	9.4553669071	21.0839513849
H	12.7587989541	8.9530000985	20.8898400119
C	10.9497462805	8.9302562642	22.0340217145
H	11.2523295406	8.0335370587	22.5680763607
C	12.4167584928	11.1746452709	19.361914909
C	13.7953463887	11.1742840523	19.5787873839
H	14.1773794786	10.7756039422	20.5141014486
C	14.716094015	11.694611016	18.6537952609
H	15.7751907664	11.672465991	18.8949817179
C	14.3087222558	12.2644857166	17.4520206076
C	12.9253276492	12.2597823724	17.2394299973
C	11.9823281759	11.7335862316	18.1414769741
C	15.2276813401	12.8068079354	16.4231862827
C	14.9742833901	14.0385542475	15.8114593153
H	14.1379965964	14.6467383752	16.1474926226
C	15.7834622716	14.5109770679	14.780740334
H	15.5385408306	15.4690264716	14.3348565808
C	16.8796093129	13.775875164	14.3220991351
C	17.1433936603	12.5553147576	14.9603787989
H	17.9809600149	11.9443262248	14.6358528139
C	16.339743217	12.0778526936	15.9876341538
H	16.5567090981	11.1126885758	16.4373425339
C	17.7410654911	14.2245286088	13.1365466997
C	19.2323322747	14.201282315	13.5192830109
H	19.5699595454	13.2004278067	13.8061477101
H	19.4308300568	14.8799811361	14.3563806566
H	19.8415173096	14.5229402265	12.6666195398
C	17.4901183176	13.25460453	11.9658584739
H	18.0629217369	13.5637852346	11.0838382603
H	16.4282384195	13.2445731261	11.6962994973
H	17.7851296218	12.2310106976	12.2214083101
C	17.3920594355	15.6428993007	12.667039043
H	17.5269492698	16.3839656579	13.4633617124
H	16.3612970152	15.7071889283	12.3016686264
H	18.0522841682	15.9181775679	11.8363155933
C	7.0883119657	9.4480435679	12.4314659794
H	6.7570179887	8.8657217054	11.5570933717
H	7.0869801004	10.509220508	12.1452442018
C	9.5168185739	11.3772263696	10.6458821421
H	8.4337643978	11.4991065812	10.6825465223
H	9.7530035848	10.8547038972	9.7139557093
H	10.9639576478	14.2995881838	14.5124656264
H	9.3494137317	14.6791531546	12.7289949446

C	4.2740433614	12.7849033878	10.1882711697
C	4.2238921006	13.0770228004	8.8258824333
H	3.3031051881	12.8907319225	8.2804899672
C	5.3158051486	13.6146062971	8.1241921957
H	5.2050342889	13.8098365479	7.061248752
C	6.5280142162	13.918226491	8.7412116424
C	6.5600040674	13.6611883401	10.1198010984
C	5.4887097014	13.0878389218	10.8331925435
C	7.7246075723	14.3848773729	8.0009539537
C	8.6162951631	15.3182074598	8.5470785951
H	8.4152280367	15.7698495481	9.5139315462
C	9.7698173279	15.6863308653	7.8670167859
H	10.43829763	16.4008419978	8.3407295851
C	10.0827376864	15.1604977599	6.606218072
C	9.1799785985	14.2437944725	6.0598846847
H	9.3707659707	13.798469989	5.0895980572
C	8.0297873194	13.8575690843	6.7433954998
H	7.3740859881	13.1131726937	6.2998216035
C	11.3721112132	15.586927501	5.8942769863
C	11.5221112722	14.9259163314	4.5172450627

3-Pt^{IV}

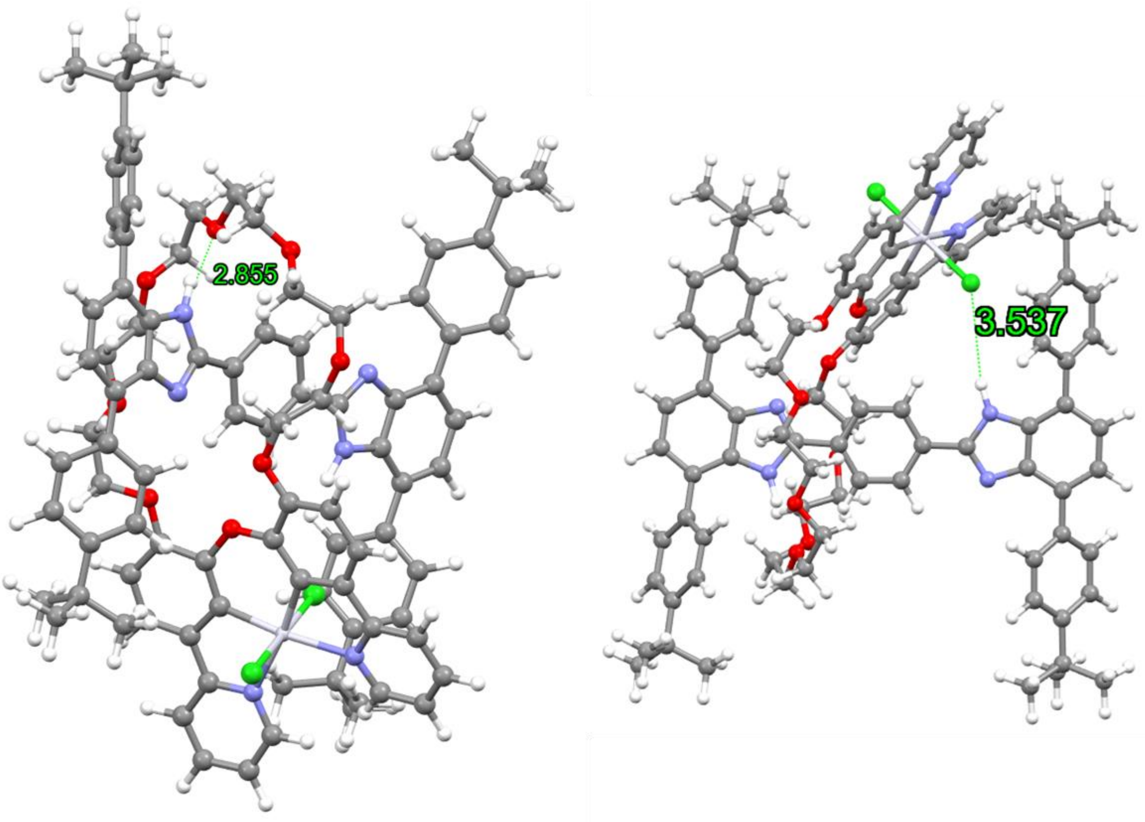

Figure S78. Computed structures for **3-Pt^{IV}** are shown from different perspectives.

3-Pt^{IV} shows an H-Bond interaction with the oxygen of the glycolic unit as in the experimental structure (Figure S78). The distance of the closest oxygen atoms to the protonated nitrogen atom is 2.855 Å which compares with 2.939 Å in the SCXRD structure. Furthermore, the angle NHO in the computed structure is 168.6° whereas the experimental has a smaller value of 138.1°.

Because of the reduced constraints compared to the SCXRD structure, one of the axial chlorine atoms can establish another H-bond with the hydrogen atom of the nitrogen atom on the second stopper at 3.573 Å. In the SCXRD structure, this distance amounts to 4.139 Å.

Table S2. Cartesian coordinates of **3-Pt^{IV}** at the 6-31g(d); cc-pVDZ-PP, ECP60MDF/ωB97XD/Acetone level of theory.

Pt	4.9762176143	-1.0160320996	-0.222287057	H	4.9491637881	0.3167082588	-7.8215638103
O	1.9175008055	-0.5329284777	-1.4950760393	H	3.3191783881	0.4937124476	-8.4912656776
O	0.029073868	-2.3005542382	-1.7605889474	C	4.3187490706	0.0562132676	-5.1988111183
O	-2.7344697462	-4.2683268167	-0.3757524402	H	4.046863332	0.4410029909	-4.2099915757
O	-5.8423598905	-2.4249797118	-0.3734345223	H	5.3607920582	0.3369045201	-5.3889180038
O	-6.4390326745	0.3091356731	-0.7135964868	H	4.2715966959	-1.0377724997	-5.1648230229
O	-5.0326068069	2.6300388326	-1.3823498002	C	-2.9364489888	-0.0772132939	-1.996433105
O	-1.6620534536	3.0269821999	-2.3869049808	C	-2.5448833196	0.0500071508	-0.5897019177
O	0.98316983	1.6706896874	-2.5875506303	C	-3.423460221	-0.1847102016	0.4723779153
N	5.6707941023	-2.7858849369	0.8495320652	H	-4.4576855215	-0.4560454517	0.2819600534
N	6.6332268158	0.409867676	-0.1945611716	C	-2.9625789352	-0.1084158246	1.7810411344
C	6.7611570456	-2.9289810375	1.6132119166	H	-3.6358811028	-0.2984681999	2.6099726467
H	7.3519304423	-2.0397199346	1.7834465205	C	-1.623445026	0.1979217617	2.0511938057
C	7.1193847573	-4.1328973919	2.1903145924	C	-0.7525090564	0.4345697314	0.9803678655
H	8.0123044332	-4.1962681367	2.7999332859	C	-1.207094713	0.3653872407	-0.3258158374
C	6.3017822227	-5.2373441046	1.9634825366	C	-1.1755021113	0.2696550271	3.448249089
H	6.5500696476	-6.204590011	2.387274781	C	-7.9659474985	-0.2869326192	8.1611152293
C	5.1514014133	-5.0851886911	1.2073988209	H	-7.9714942253	-0.6033878703	7.1120386748
H	4.4927922306	-5.9297426871	1.0484659196	H	-7.8650196357	0.8037514626	8.192879692
C	4.8366163259	-3.8361869738	0.6656655119	H	-8.940797289	-0.5429363837	8.590334462
C	3.5985953853	-3.5334257122	-0.0620057858	C	-6.9490563734	-0.5156646544	10.424784053
C	2.5986505413	-4.4708153427	-0.2861907018	H	-7.9436992873	-0.7363386344	10.8298385158
H	2.7172796581	-5.5080843039	0.0065820142	H	-6.7815293627	0.5647709844	10.4985397249
C	1.407293823	-4.0811156373	-0.8782752794	H	-6.2109796802	-1.0169740053	11.059485144
H	0.6374082507	-4.8251371498	-1.0345383294	C	-7.0973047936	-2.5051260491	8.8925251793
C	1.1877133158	-2.7520662639	-1.2394609779	H	-6.3645178173	-3.0594786842	9.4880364806
C	2.2255967917	-1.8006052676	-1.070302811	H	-7.0346037174	-2.863156634	7.8587680142
C	3.4126376834	-2.201224065	-0.4817601134	H	-8.0944500409	-2.7469842557	9.2789131362
C	-1.6622716496	-3.3442619969	-0.3431007328	C	-6.8560210291	-0.9845423194	8.959806952
H	-0.8871957354	-3.7313057789	0.3283700604	C	-5.4588264107	-0.6679311593	8.4128083599
H	-1.9789510418	-2.3732139063	0.0464536916	C	-5.2421127071	0.1590126993	7.3072834027
C	-3.9652500539	-3.7856420077	0.124329834	H	-6.0803234041	0.6182870302	6.7945448488
H	-3.8078111191	-3.0453114575	0.9194676138	C	-3.9597254744	0.4222870779	6.8320117501
H	-4.4941024895	-4.6388932988	0.5595001773	H	-3.8323062429	1.0652777291	5.9679065625
C	-6.8286325356	-1.9664371966	-1.2742237107	C	-2.8343270944	-0.1320619961	7.447957965
H	-7.6167698986	-2.7224694865	-1.4036863658	C	-3.0446406732	-0.9643803523	8.5552971267
H	-6.3951833983	-1.766907047	-2.2630429888	H	-2.1954698243	-1.4334703921	9.0448766341
C	-7.4395714421	-0.6924266565	-0.7335056071	C	-4.3256471349	-1.2226599285	9.023800448
H	-7.8246016865	-0.8520495558	0.2836086117	H	-4.4384965971	-1.8807762421	9.8811896587
H	-8.2707920567	-0.3887492934	-1.3827448005	C	-1.4632795007	0.156634869	6.9664418218
C	-6.8954520611	1.5873571902	-0.3193318942	C	-0.3996122102	0.3244297819	7.8515140861
H	-7.4311745503	1.5202118767	0.637882194	H	-0.5909227482	0.2740303044	8.9194920349
H	-7.5860752878	1.9891685372	-1.0725831599	C	0.9089033482	0.5957244454	7.421791023
C	-5.7009832859	2.500374942	-0.1478438872	H	1.6856663328	0.7429600941	8.1669140342
H	-6.045249431	3.4791240567	0.2198161456	C	1.2396915509	0.7193970871	6.0752089028
H	-5.0230889507	2.0736750447	0.6057609189	C	0.1708105389	0.5363672025	5.18652922
C	-3.7443497861	3.1980007823	-1.2530595356	C	-1.1492662005	0.2672968506	5.5956017017
H	-3.1461725557	2.6225523538	-0.5338142675	C	2.6134921699	1.0136147763	5.6059197461
H	-3.8151348133	4.2323925184	-0.8856787914	C	2.8482635766	1.9617637316	4.6060761885
C	-3.05113467	3.1296273702	-2.6095220609	H	2.0180733419	2.5290245935	4.1922315757
H	-3.4260220713	2.2509540952	-3.1494779631	C	4.1359818603	2.2079541109	4.1377570523
H	-3.2767266846	4.0152250022	-3.2239884627	H	4.2613009256	2.9385157614	3.3468648929
C	-0.9558957334	2.7393669752	-3.5734513029	C	5.2409200648	1.5219630658	4.6464036868
H	-1.1845061675	3.4927118156	-4.3449453556	C	5.0017573403	0.6031500785	5.6774014765
H	-1.2268131136	1.7531529901	-3.9584258886	H	5.8299777381	0.0540470287	6.116702877
C	0.5269510434	2.8070299668	-3.3185955034	C	3.7195268508	0.3469679236	6.1453633948
H	1.0513057005	2.8418357659	-4.2820310956	H	3.5694793966	-0.3981360006	6.9220119774

H 0.7388572392 3.7249113945 -2.7583272732	C 6.664625711 1.7304761996 4.1165670809
C 4.1289257474 0.4934073161 -1.1935395661	C 7.5314409891 2.3553296421 5.2257386968
C 2.8112452913 0.4983922256 -1.6253966797	H 7.5702837446 1.7147225986 6.1135754006
C 2.2875996082 1.6662710507 -2.2438303383	H 7.1333977131 3.330561337 5.5283147642
C 3.1323253068 2.7572486641 -2.4588247634	H 8.558202954 2.5001606673 4.8690285376
H 2.7704547893 3.6444069222 -2.9584716294	C 7.2609774904 0.3712392349 3.7023076206
C 4.4481368522 2.7422823511 -2.0224454121	H 8.265171769 0.5132880352 3.2853767423
H 5.058534921 3.6216986904 -2.1943987599	H 6.6279694677 -0.0965335502 2.9410474268
C 4.9490634862 1.6264436278 -1.3673485079	H 7.3460878425 -0.3182217806 4.5484462004
C 6.3123572622 1.56170838 -0.8282713174	C 6.6961682189 2.6503237713 2.8879546515
C 7.2571643167 2.5846551311 -0.9474330758	H 6.3353507699 3.6584261298 3.1208564498
H 6.9975924246 3.5108772697 -1.4440534568	H 6.0913553073 2.242397566 2.0700715896
C 8.5332481268 2.4067829263 -0.4400118052	H 7.7262956411 2.7437848955 2.5281089023
H 9.2703462031 3.197379657 -0.5313560346	C -4.8018817889 -3.1565324497 -0.9844493393
C 8.8593204323 1.1997177217 0.1724225935	H -5.2005977197 -3.9373537457 -1.6481623461
H 9.8487673118 1.0063461177 0.5682293391	H -4.1735168012 -2.4897528868 -1.5926823808
C 7.8769208415 0.2320424914 0.2677784607	C -1.110117018 -3.15530799 -1.7566508466
H 8.0947736281 -0.7269068584 0.7150805753	H -1.8384198328 -2.6383301751 -2.3812323828
N -4.2213351805 -0.0918230969 -2.4655958482	H -0.8843467985 -4.1206484652 -2.2237615697
H -5.0521374406 0.148124653 -1.9191064317	H 0.2921454955 0.6810009321 1.1449748233
N -2.0462080659 -0.2172263666 -2.9497282675	H -0.5319866936 0.5467948686 -1.1531577981
N -1.9624245275 0.1096797697 4.4895191895	Cl 5.9212348823 -1.8655686765 -2.2022656927
N 0.12024552 0.5306226176 3.8099955122	Cl 4.041652604 -0.1931684626 1.7510494156
H 0.9181966946 0.6171927693 3.1983174478	
C -11.2971745215 1.7287915866 -4.3612322799	
H -10.750948495 2.5727898627 -3.9275485453	
H -12.3633208661 1.8814868041 -4.1571501623	
H -11.1499421986 1.7510898871 -5.446777009	
C -11.0444047806 0.3871251783 -2.2476008504	
H -10.7370815692 -0.5696909721 -1.8096673915	
H -12.1034173192 0.5444482792 -2.0119862569	
H -10.4711661397 1.1830903369 -1.7600791291	
C -11.705036816 -0.7297959115 -4.3751369245	
H -11.6153303415 -0.7704247533 -5.466465699	
H -12.756571753 -0.5393922482 -4.1346758383	
H -11.4452299743 -1.7139836773 -3.9695023941	
C -10.8334739332 0.3816814108 -3.7743957365	
C -9.3437762336 0.1790157525 -4.0754715881	
C -8.4063769511 1.1141954189 -3.6137912707	
H -8.7450794421 1.9897709284 -3.0665360983	
C -7.0444505391 0.9588487587 -3.8333940028	
H -6.349903265 1.698386966 -3.4414428683	
C -6.5636475009 -0.1371616382 -4.5609052936	
C -7.4883510059 -1.0678783917 -5.0363320665	
H -7.1373964436 -1.9313375692 -5.5951230917	
C -8.8516281866 -0.9157836742 -4.7900998782	
H -9.5284383725 -1.6730318204 -5.1700245869	
C -5.1161343873 -0.2828784188 -4.8422538105	
C -4.6251035747 -0.4444634016 -6.136407009	
H -5.3361870144 -0.4944122882 -6.9565183267	
C -3.2503706853 -0.4897323997 -6.4287359635	
H -2.9447206381 -0.5785810755 -7.4675579393	
C -2.2783227481 -0.4002147527 -5.4359420335	
C -2.7643613321 -0.313367283 -4.1153573984	
C -4.1417787271 -0.2304862797 -3.8360390148	
C -0.8265700392 -0.261327155 -5.7116097585	
C 0.1399422042 -0.7593629238 -4.8325616011	
H -0.1609112349 -1.3146921692 -3.9530646353	
C 1.4917408752 -0.4884216045 -5.0212402163	
H 2.1904316237 -0.8732069007 -4.2864088212	
C 1.9436899154 0.2831189539 -6.0933680446	
C 0.9731627928 0.7507612332 -6.9924417191	
H 1.2701871319 1.3541672983 -7.8463493291	
C -0.378799611 0.4906329219 -6.8072920365	
H -1.1001323414 0.9153238864 -7.5006631159	
C 3.4216934249 0.6350101667 -6.3018407872	

C 3.5897045708 2.1665053872 -6.2944242305<br>H 2.9922619444 2.648399174 -7.0754067956<br>H 4.6401943766 2.4322744261 -6.4630108374<br>H 3.287975957 2.580933662 -5.325911552<br>C 3.8924102731 0.0744345518 -7.6574744004<br>H 3.7834310094 -1.0156722764 -7.6854283806	

Dimeric $Pt^{III}Cl_2$ and 3-Pt^{III}

In the case of the dimeric $\mathbf{Pt^{III}Cl_2}$ crown, the trend of the distances shows that it is very sensitive to the Pt-Pt rotation and the level of theory used. The largest distance is observed when the two bridging oxygen atoms are fully trans while shorter distance (2.719 Å) was observed in the minimum corresponding to the *syn* oxygen orientation, with a O-Pt-Pt-O dihedral angle (θ) of 47°. The closest to a trans was an *anti*-conformation at θ = 125.8°.

Figure S79 shows the trend of the free $\mathbf{Pt^{III}Cl_2}$ crown dimer while rotating around the Pt-Pt bond at the 6-31g(d);cc-pVDZ-PP,ECP60MDF/ωB97XD/DCM level of theory.

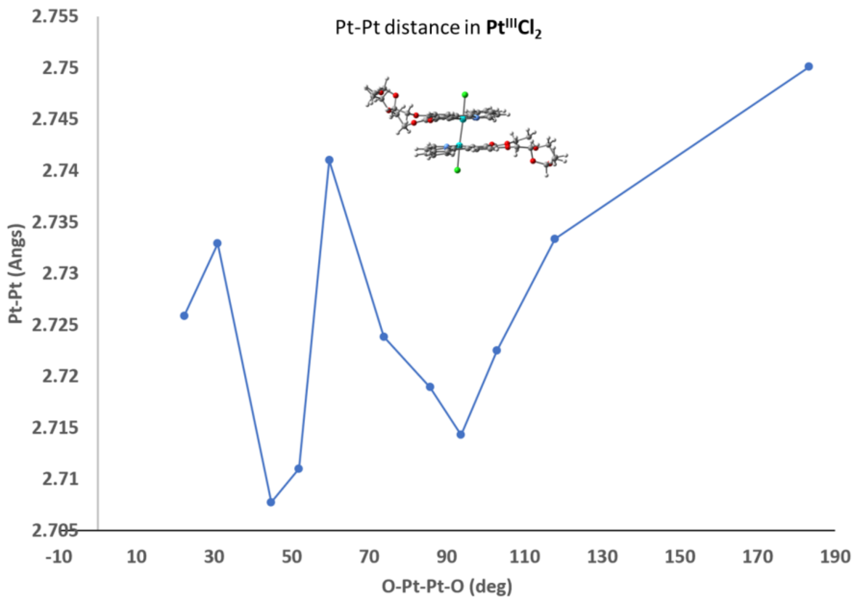

Figure S79. Graph showing Pt-Pt distance (Å) in $\mathbf{Pt^{III}Cl_2}$ crown as a function of O-Pt-O-Pt dihedral angle (°).

Furthermore, the distance is also very sensitive to the basis set and the xc-functional. Below are the results obtained with different levels of theory for the minimum energy structure closer to the trans conformation (termed here as the *anti*-conformation) and the fully trans conformation. *Anti*-conformations are in the range of θ = 120 – 135° for the O-Pt-Pt-O torsion; so, they are closer to trans conformation but are not fully trans.

Table S3. A comparison of Pt-Pt distances and Pt-Cl bond lengths obtained for minimum energy structure using different levels of theory.

(H C N O)basis set /xc-functional/solvent	d(Pt-Pt) (ECP60MDF/cc-pVDZPP)	Cl (6-31g(d))
trans conformation		
6-31g(d)/ωB97XD/DCM	2.750 Å	2.500 Å
6-31g(d)/ωB97XD/DCM (different crown conformation)	2.746 Å	2.497 Å
6-31g/PBE1PBE/DCM	2.753 Å	2.493 Å
anti conformation		
6-31g(d)/ωB97XD/vac	2.732 Å	2.449 Å
6-31g(d)/ωB97XD/DCM	2.715 Å	2.503 Å
6-31g(d)/PBE1PBE/DCM	2.739 Å	2.476 Å
6-31g(d)/PW6B95D3/DCM	2.701 Å	2.475 Å
6-31g/PBE1PBE/DCM	2.739 Å	2.475 Å
(H C N O)basis set /xc-functional/solvent	d(Pt-Pt) (ECP60MDF/cc-pVTZPP)	Cl (6-31g(d))
6-31g(d)/wB97XD/DCM	2.747 Å	2.480 Å

This is relevant because the calculation of the larger **3-Pt^{III}** has been performed using the smaller basis set on hydrogen and the second-row atoms though retaining the 6-31g(d) basis set on the chlorine atoms and the cc-pVDZ-PP with ECP60MDF pseudopotential on the Pt atoms. Furthermore, we used the hybrid PBE1PBE functional, which performs better than the hybrid b3lyp xc-functional, (6-31g;6-31g(d); cc-pVDZ-PP, ECP60MDF/BBE1PBE/DCM level of theory) that shows less geometry convergence problem than the more complex meta hybrid xc-functional including dispersion corrections we used in smaller molecules.

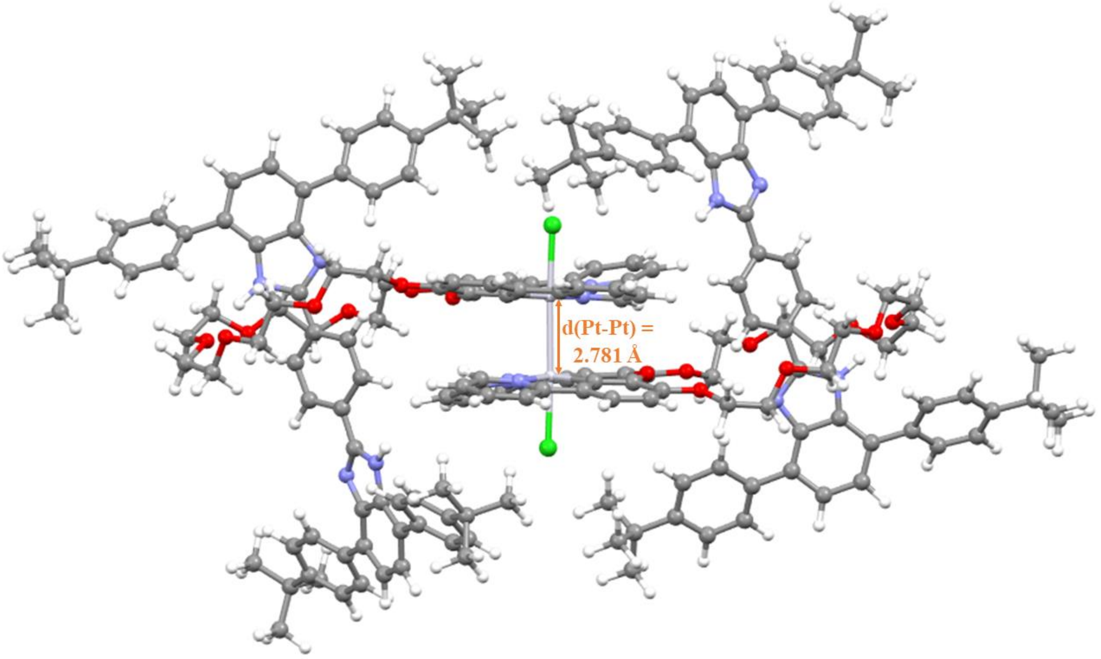

Figure S80. Computed structure of **3-Pt^{III}** showing the Pt-Pt distance of 2.781 Å, as obtained from 6-31g; cc-pVDZ-PP, ECP60MDF/PBE1PBE/DCM level of theory.

The calculation has been performed assuming that the H-bond between the protonated hydrogen and the oxygen of the glycolic units are preserved. This is computed at 2.772 Å with a θ of 163°, which is larger than the calculated one for **3-Pt^{IV}**, but closer to the experimental value. The Pt-Cl distance does not change significantly compared to the free dimeric **Pt^{III}**-crown complex (2.493 Å) being 2.472 Å at the same level of theory. The Pt-Pt distance is slightly longer, 2.781 Å than the distance (2.753 Å) found in the free **Pt^{III}Cl$_2$** crown at the same level of theory 6-31g; cc-pVDZ-PP, ECP60MDF/PBE1PBE/DCM as the **3-Pt^{III}** with the **Pt^{III}** dimer locked in the full trans conformation.

Table S4. Cartesian coordinates for **3-Pt^{III}** at the 631g; Cl 6-31g(d), Pt cc-pVDZ-PP, ECP60MDF level of theory.

Pt	5.7417873154	9.9456974613	15.7601244142
O	7.5294090947	12.3678995453	14.2384400753
O	9.2680508713	13.9855489472	15.3242968138
O	12.3223303162	15.8383256996	16.2980624549
O	14.0330221987	17.6080114463	13.5889319197
O	13.5828354207	17.783500016	10.7328539251
O	11.742193398	16.7326980194	8.8661479546
O	8.7251367308	14.9191613341	9.6037101429
O	7.0629615632	13.4514791702	11.8277416549
N	5.6977689393	9.5166257338	17.8812102441
N	4.2296089055	8.7065160644	14.8513510764
C	4.854587398	8.6931791706	18.5349222204
H	4.224502304	8.0649416122	17.9295388143
C	4.767981342	8.6471277772	19.9181286418
H	4.071154404	7.9686790328	20.3927314865
C	5.591818294	9.4919600554	20.6652618607
H	5.5512126614	9.4872877286	21.7485156678
C	6.4531072025	10.352583085	20.0004647361
H	7.0797998571	11.0324877633	20.5622371497
C	6.4927584129	10.367462059	18.5987776557
C	7.2837099727	11.2989172382	17.8101430263
C	8.1890589046	12.2027842735	18.3728149374
H	8.384167399	12.2107790721	19.4392841043
C	8.8710775353	13.1007164501	17.561937634
H	9.5928916705	13.7757382392	18.0036056177
C	8.6316249982	13.1291313995	16.1860123232
C	7.6974229735	12.2299926466	15.6189347891
C	7.0557083494	11.2857618628	16.4118444731
C	11.5336595844	14.6698830342	15.9496944527
H	11.6726247472	13.9132016951	16.7306629186
H	11.8646109996	14.2452264965	14.9953112324
C	13.4121985514	16.1898248636	15.4068377989
H	13.5752079845	15.4016646038	14.663703696
H	14.3196245074	16.2878387221	16.0098714049
C	13.951547367	18.858901075	12.8632898432
H	14.5521330121	19.6260037869	13.3706318954
H	12.9138475369	19.2105170756	12.8247862289
C	14.4813185268	18.658808146	11.4644627663
H	15.4809932379	18.2070497938	11.4925516739
H	14.5438267397	19.6313551271	10.958576291
C	13.9477092082	17.561839342	9.3471094917
H	15.0165511757	17.3193071473	9.2817849795
H	13.7586889774	18.4744557061	8.7707142531
C	13.1487051522	16.4011245135	8.8090250097
H	13.456645604	16.1981916156	7.7736815926
H	13.3413727649	15.5042457656	9.4113274153
C	10.8587581747	15.5902578951	8.7202286038
H	11.159408179	14.7931846882	9.4110765987
H	10.9052406021	15.2020756969	7.6946978457
C	9.4650257375	16.0592013813	9.0983618539
H	9.5627933518	16.8232468734	9.8773487869
H	8.9312063726	16.5028820768	8.2469539206
C	7.5241349371	15.319878607	10.3094776551
H	6.9463271922	16.0101580216	9.677459955
H	7.7875143357	15.8349099236	11.2406932351
C	6.6637634414	14.117275684	10.5857379212
H	5.6199277472	14.4339663283	10.6892211625
H	6.7440472955	13.4172825035	9.7469693007
C	5.7003715395	10.7441784605	13.9381109458

C	6.4766094761	11.8188647824	13.5090861143
C	6.2560359022	12.4005511533	12.2346016942
C	5.2371239915	11.896908126	11.4203970814
H	5.0485826499	12.3234977654	10.445974753
C	4.4495766617	10.8353073774	11.8453818862
H	3.6650117724	10.4757389198	11.1895630926
C	4.672006862	10.2571549448	13.0945110154
C	3.8838034628	9.1443422556	13.6048069812
C	2.8414863487	8.5196129858	12.9056503877
H	2.5729686582	8.8656641285	11.9161961911
C	2.1573885632	7.4570187604	13.4798709252
H	1.3515138605	6.9714579583	12.9415518991
C	2.5315566574	7.0140605649	14.7501093306
H	2.0396681156	6.1767446545	15.2279035104
C	3.576549318	7.65995231	15.3929668497
H	3.9293272927	7.3120251431	16.3498418883
N	11.0310747695	17.5241207477	11.7843693462
H	11.8876378689	17.6404457326	11.2255649461
N	9.4441458281	16.4861032565	12.9849370243
N	14.7936750084	11.3618578893	11.8646711382
N	12.9572831784	10.1450658783	11.4538668456
H	11.988836708	9.9067758551	11.3181817513
C	13.0540835676	23.1677893131	7.0873857956
H	12.7830160699	22.2296464344	6.5917839401
H	13.7259882026	23.7206857379	6.4202397742
H	12.1413364877	23.7587755475	7.2232048455
C	15.0347122517	22.0938055942	8.2108445946
H	15.5529432435	21.9090727689	9.1589197967
H	15.7152971997	22.6414570292	7.5481174843
H	14.8166062069	21.1262412046	7.7462963886
C	14.1453029378	24.2736455273	9.0416670709
H	13.2712052543	24.9144845418	9.2038855629
H	14.817304713	24.7929552881	8.3496119239
H	14.6723928982	24.1566025216	9.9953898922
C	13.748553147	22.9156717896	8.4429372028
C	12.8036156234	22.131914553	9.3615585944
C	12.3357328373	20.8645993375	8.9703683605
H	12.6333563394	20.4572401269	8.0084040863
C	11.4810686119	20.1134371428	9.7740017497
H	11.1481151911	19.1392142531	9.4276591395
C	11.0389441821	20.6147046886	11.0108003271
C	11.5022012283	21.8787009185	11.4075819913
H	11.1900270534	22.2827030602	12.3660453119
C	12.3686489726	22.6188298407	10.6019962672
H	12.7040101914	23.5865312731	10.956127372
C	10.0657162523	19.8840920231	11.8506462456
C	8.9838444865	20.5580274057	12.4289302344
H	8.8682792712	21.6179097095	12.2262383733
C	8.0291940097	19.9193477717	13.2311554278
H	7.2145639531	20.5186299926	13.6215802094
C	8.0692399865	18.5485161614	13.5186266456
C	9.1285869357	17.8394059064	12.9059009188
C	10.1130853227	18.5039716519	12.1227810803
C	7.0872565702	17.9360330918	14.4400260966
C	6.7831242321	16.5604325162	14.4283042799
H	7.3038681777	15.8996108404	13.7467765202
C	5.8511707982	16.0264088637	15.3151109458
H	5.6520308844	14.9594458993	15.2694204486
C	5.1787759983	16.8216204276	16.2591321891
C	5.4923387335	18.1875968354	16.2759546627
H	5.0193179941	18.8471300968	16.994309913
C	6.4248378242	18.7296747029	15.3931044562
H	6.6586521721	19.7859761948	15.4745912637

C	4.1695059051	16.1879007224	17.2225829373
C	3.5569503949	17.2191334651	18.182843417
H	4.321513327	17.6924384362	18.8095898759
H	2.8437402815	16.7174384959	18.8462446086
H	3.0162576244	18.0043832305	17.6419548384
C	4.8777329163	15.1037958895	18.0634067408
H	5.2982030289	14.3140602356	17.4326080936
H	4.1625776215	14.6380024451	18.7520815268
H	5.6901782705	15.5420829462	18.6544638577
C	3.025188096	15.5413478912	16.4131615569
H	2.4999572204	16.295063519	15.8151429686
H	2.3020558013	15.0761121206	17.0937789056
H	3.3986474413	14.7646657024	15.7382367741
C	10.5856737792	16.3317410902	12.3095189931
C	11.3001997908	15.0652764689	12.1522145381
C	12.7037877987	15.0439733471	12.0770804343
H	13.2635337569	15.9693585054	12.1550757186
C	13.3845975065	13.8424713283	11.9336492649
H	14.4675725799	13.8240750873	11.8970641521
C	12.6883784594	12.6259642447	11.8435600313
C	11.284770948	12.6507669255	11.91274518
C	10.596911281	13.8513726038	12.0782917022
C	13.469941879	11.4010210303	11.716821067
C	21.3923628679	13.826780038	12.3013868406
H	20.5867036234	14.4456833915	12.7123899452
H	21.2902213661	13.8030142198	11.2104394827
H	22.343968722	14.3154320969	12.5382331669
C	22.5703749879	11.625774441	12.3071988396
H	23.5107933928	12.146346117	12.5241865675
H	22.4702014552	11.5354353314	11.2196353125
H	22.6371802243	10.6173695273	12.7289147028
C	21.5673774723	12.5338316186	14.4298957477
H	21.6079610549	11.5503649385	14.9098591721
H	20.7410720938	13.0982116209	14.8766798873
H	22.5037310402	13.0588560019	14.6536988239
C	21.3828628961	12.4127846513	12.9018157506
C	20.0769932346	11.6659852221	12.6051773388
C	19.0203697102	12.2340504286	11.879817739
H	19.1071201064	13.2433903519	11.4944497137
C	17.8416465767	11.5293491543	11.6307776681
H	17.040435487	12.0027524154	11.0764949397
C	17.6690534106	10.2167000985	12.0968214754
C	18.7252017165	9.6450002031	12.8304047305
H	18.6179016999	8.643325307	13.2350201825
C	19.8970610147	10.3530939326	13.0756634514
H	20.6819020501	9.8745049149	13.6538077467
C	16.4432601674	9.4431724641	11.8161967369
C	16.4820181159	8.0534791691	11.6528411147
H	17.4388829111	7.5446825988	11.6993440258
C	15.3364743619	7.290794386	11.3839671287
H	15.4457460013	6.2219006448	11.2324031404
C	14.0607267007	7.854522389	11.2569372181
C	14.0136759218	9.2473712874	11.4342728203
C	15.1654633309	10.0325407174	11.7000941294
C	12.8889745302	7.0142158226	10.9354776761
C	11.9396974416	7.3997914055	9.9775516615
H	12.0458356909	8.3507895664	9.4625142918
C	10.8894751938	6.5494940444	9.6223065605
H	10.1916489201	6.882021872	8.8627950689
C	10.7427378622	5.2840311777	10.2069838494
C	11.68292337	4.9148910899	11.1855902009
H	11.5977408421	3.9521784762	11.6800479796
C	12.7306034775	5.756253811	11.5432795657

H	13.4336151579	5.4423896999	12.3087503704
C	9.6278641291	4.3064049527	9.8179397253
C	10.2567710366	2.989966909	9.3123753783
H	10.8803507851	2.5190979533	10.0794394344
H	10.8795407197	3.1709471496	8.4289032862
H	9.4668640465	2.2809195583	9.0373748494
C	8.7527034976	4.0107299941	11.0541094307
H	7.9582259722	3.3022754981	10.7897919463
H	8.2873884764	4.9286290367	11.4287727671
H	9.3396113218	3.5727847731	11.8684013472
C	8.7278758982	4.8649611967	8.7057121961
H	9.2966372737	5.0715537557	7.7917952569
H	8.2249737794	5.7864173297	9.0187873024
H	7.9550086675	4.1282158598	8.4599833314
C	13.0953819942	17.4929741903	14.6894448824
H	13.1893955494	18.350203507	15.3689054115
H	12.0670383402	17.4673857643	14.3067069021
C	10.0763466081	15.0899934292	15.8257839838
H	9.9646149135	15.853652396	15.0570792738
H	9.6791274783	15.4653730054	16.774199892
H	10.7129112589	11.7310713134	11.838791869
H	9.5153731595	13.8528425828	12.1304939692
Pt	7.7543003644	8.1353407886	15.1226116272
O	6.3010829937	5.8640583201	17.145852963
O	6.8629169644	5.3639029872	19.6481674104
O	6.6739964566	5.183344468	23.3443547806
O	4.1178763641	2.9456549974	24.7000612844
O	2.0757872168	1.3269650342	23.450497875
O	1.1662705647	0.8497916124	20.8082076159
O	2.6569756375	1.8736388105	17.6896005022
O	4.8636408165	3.7197168869	16.4097705942
N	9.4113110245	9.4383880163	15.6249387019
N	7.4058617697	8.3741865368	13.005878452
C	10.1272610707	10.2188464674	14.7918695708
H	9.746055736	10.3408096581	13.7928933611
C	11.3061654114	10.8424698335	15.1708269377
H	11.8405233095	11.4600372637	14.4611569446
C	11.7695787343	10.6556546487	16.4747625097
H	12.6887980529	11.1241539076	16.8074848485
C	11.0423872517	9.8490612436	17.3375403062
H	11.3973743058	9.6747656329	18.3445943659
C	9.8615646274	9.2322217344	16.8995798746
C	9.0837013371	8.2948281688	17.6937587582
C	9.3712631499	8.0030112244	19.0295996504
H	10.1635192054	8.5204376233	19.5586710703
C	8.6328343071	7.041859173	19.7072063045
H	8.8530938909	6.8382918765	20.747109173
C	7.6192890042	6.3409416413	19.0495863717
C	7.3315452301	6.6312045271	17.6947511698
C	8.028967662	7.6284314095	17.022663622
C	6.4421015026	5.7569462307	22.0310790174
H	6.8104608404	6.7891839992	22.0354146952
H	5.3725306728	5.7741452063	21.7938189337
C	5.5059190261	4.7975053409	24.1137948735
H	4.5883235106	5.1429686894	23.6253171545
H	5.5764119235	5.2778396333	25.0940915226
C	3.9217741309	1.5404094106	24.9907107166
H	4.2441290011	1.3210898403	26.0175899023
H	4.5179915984	0.9227134991	24.3086804627
C	2.4579392363	1.2040632966	24.8464770619
H	1.845050179	1.8864718607	25.4487855157
H	2.2869252893	0.1747917375	25.187931345
C	0.6971484424	0.975915595	23.1671409698

H	0.0388939324	1.4450451619	23.9102188044
H	0.5786234276	-0.1112746532	23.2308623946
C	0.3255137787	1.4866560233	21.7976508122
H	-0.7334250924	1.2632828369	21.6053256962
H	0.4640122627	2.5742821982	21.7543021153
C	1.1374205824	1.5005718058	19.5113279412
H	1.3267042775	2.5747279012	19.6273669495
H	0.1556171732	1.3658011726	19.0392061389
C	2.2600075887	0.898212584	18.6861896422
H	3.0977117282	0.677118606	19.3567416328
H	1.9575377465	-0.0370465616	18.1957943747
C	3.9377435866	1.5536447346	17.0919292001
H	3.9061466711	0.5284803411	16.6944715077
H	4.7309974399	1.6098508949	17.8467914669
C	4.2206517837	2.4875375287	15.9458689249
H	4.8972110602	1.996325164	15.2377305176
H	3.2816211478	2.7280614815	15.4356376107
C	6.4932547091	6.6261838144	14.8132780654
C	6.0624350802	5.7217056832	15.7818388022
C	5.2856165026	4.5919873928	15.4180273006
C	4.9628601769	4.3899860696	14.0728709058
H	4.3694097836	3.5395696417	13.7698614358
C	5.3968140141	5.2797892013	13.0991285271
H	5.132378773	5.087097041	12.0657012312
C	6.160572325	6.3906272659	13.4569183191
C	6.6600935927	7.3544790936	12.4866431073
C	6.4200225262	7.2916252177	11.1065513734
H	5.8250812399	6.4850052445	10.6989840413
C	6.942770717	8.2627795974	10.2633857794
H	6.7589985406	8.2163707623	9.1960101739
C	7.6951865856	9.3034631326	10.81134136
H	8.1060797544	10.0934892232	10.1962551623
C	7.8922775075	9.3242237064	12.1839075407
H	8.4223681033	10.1396128088	12.6469146364
N	4.1575916539	1.2422788431	21.6166121792
H	3.3102912651	1.1634923725	22.1965791846
N	5.6123897473	2.1274381757	20.1525054946
N	0.7051497811	7.6370205248	21.5700950839
N	0.5706728671	7.4780991517	19.3381378878
H	0.7234802836	7.1357744001	18.4040245344
C	0.8355024967	-4.9601323627	24.5700453998
H	0.2921819359	-4.510021229	23.7325622576
H	0.1132591777	-5.509845603	25.1851665529
H	1.5572029395	-5.6773973823	24.1632547468
C	0.5024013167	-2.9089139599	25.9913701331
H	0.9836864908	-2.1450193101	26.6129806669
H	-0.2229137279	-3.4494501001	26.61109429
H	-0.0477621436	-2.4018949698	25.1915402933
C	2.2354594314	-4.5980122784	26.6050538698
H	2.9742080923	-5.3315177816	26.2626383012
H	1.4827272473	-5.1316699605	27.1956228883
H	2.7363699195	-3.8837021626	27.2683418308
C	1.5498398798	-3.8918837418	25.4255751372
C	2.5479486091	-3.1230205926	24.5516644659
C	2.0940243962	-2.4190449527	23.4217539171
H	1.0385760989	-2.4413963984	23.1657211938
C	2.9627418382	-1.7023765733	22.6018415875
H	2.5668214238	-1.1744035533	21.7388635189
C	4.3420827437	-1.6770748265	22.8719485902
C	4.8020535739	-2.3771982721	23.997515396
H	5.8612522305	-2.3618696289	24.236741546
C	3.9227680883	-3.0808522249	24.8218754375
H	4.3254590954	-3.6001224759	25.683566074

C	5.3025909004	-1.0072339611	21.9689611644
C	6.4722359966	-1.6633859657	21.5703882986
H	6.6468758041	-2.6719467772	21.9311122445
C	7.4037710289	-1.0910056357	20.6944070225
H	8.2651321099	-1.6860606929	20.4132841292
C	7.245119899	0.1860951871	20.1398906163
C	6.0584722289	0.8589702098	20.5125981717
C	5.1316747546	0.2780236245	21.4226543989
C	8.2762080922	0.747934454	19.2406886527
C	7.9947649534	1.7282287668	18.273778455
H	7.0007140825	2.1545188541	18.2174405227
C	8.9819644386	2.1780382974	17.3930890739
H	8.7093881218	2.9268298026	16.6578615496
C	10.2930340135	1.6843150778	17.4396345877
C	10.5815417444	0.7379344753	18.4383020137
H	11.5907722496	0.350017856	18.5396551257
C	9.6044746885	0.2832432176	19.3151440869
H	9.8852509904	-0.4302302374	20.082769164
C	11.3982824993	2.1376825799	16.479108622
C	12.0036964744	0.9061452615	15.7716741744
H	12.4385439407	0.1989059546	16.4856285926
H	12.7981950273	1.2241693975	15.0859962514
H	11.2390782857	0.3783777528	15.1902572026
C	12.5044122082	2.8573782454	17.2806344785
H	12.1031812996	3.7458264848	17.7810279216
H	13.3094636817	3.175735853	16.6073934885
H	12.9383259676	2.201389019	18.0431964271
C	10.8737116033	3.0977871982	15.402522934
H	10.0856402338	2.6328345262	14.7983606417
H	11.6940334286	3.3732946773	14.7296752565
H	10.4762139266	4.0231935611	15.8314872379
C	4.4793835731	2.3268741619	20.8329063178
C	3.660381848	3.5350902227	20.7585228431
C	2.8723411374	3.9440911037	21.8481919272
H	2.8996227443	3.3870566804	22.7780969315
C	2.0641690778	5.0701065655	21.7486953093
H	1.4624979679	5.3907823941	22.5916485823
C	2.0008757509	5.8052512902	20.5540914996
C	2.8058945676	5.4060981453	19.4740442743
C	3.6394387663	4.2958801137	19.5790127671
C	1.1130376717	6.9607835793	20.4979302603
C	-0.4575639393	9.0092358313	28.4012712625
H	0.4761554738	8.4974446608	28.1418738177
H	-1.2935639296	8.3293154203	28.2013158006
H	-0.4405561541	9.2116328507	29.477920223
C	-1.9218722318	11.0071271362	28.089918218
H	-1.9040806738	11.1708510952	29.1740550015
H	-2.7847836715	10.3750024985	27.8515495077
H	-2.0648561191	11.9778020778	27.6035835629
C	0.582793918	11.2433847101	28.0028068368
H	0.507451517	12.221309532	27.5158473373
H	1.5298366381	10.7828335641	27.6996748977
H	0.6104710038	11.4062364059	29.0868704575
C	-0.6091101808	10.3330145586	27.636645684
C	-0.642877672	10.1244113834	26.1180910562
C	-0.5372201657	8.8618805187	25.5182283408
H	-0.4284770885	7.9759618993	26.1331789899
C	-0.5715872075	8.7049242098	24.1317958873
H	-0.4740000875	7.7158838912	23.7012670444
C	-0.7173422979	9.8109864518	23.279770698
C	-0.8135538	11.0812920963	23.8781533225
H	-0.8927806116	11.9654384848	23.2533088036
C	-0.7783477903	11.2306221594	25.2605237796

H	-0.8492633294	12.2310931013	25.6768634019
C	-0.7972471164	9.6630894177	21.8127708643
C	-1.552901521	10.5488581897	21.0347318174
H	-2.1121395363	11.3365122955	21.527962555
C	-1.6645034015	10.4298485053	19.6423059616
H	-2.3071354691	11.1241174438	19.1109823094
C	-1.0236172045	9.420671311	18.9127432491
C	-0.2431163001	8.5448307576	19.6857353448
C	-0.1377191313	8.6396643582	21.0976993363
C	-1.2192043108	9.2924400407	17.4543221869
C	-1.4096572778	8.0449790358	16.8409244133
H	-1.4002381044	7.139112875	17.4408140144
C	-1.6831503042	7.9470149783	15.4746697505
H	-1.8401436818	6.9622395801	15.0503185718
C	-1.7846649232	9.0869503102	14.665357767
C	-1.5743660433	10.333063329	15.2825782
H	-1.6318287324	11.2432457035	14.6937621827
C	-1.2944415166	10.4367874583	16.6407906107
H	-1.1256634141	11.415263725	17.0796303361
C	-2.1453423922	9.024097682	13.1762271166
C	-3.507541816	9.720267644	12.9623069565
H	-3.4748262982	10.7678325325	13.2802732385
H	-4.2957501847	9.2144380669	13.5312754762
H	-3.778854394	9.6953319039	11.9001072079
C	-1.0705462319	9.7537173005	12.3438673819
H	-1.3253410233	9.7015082135	11.2787832882
H	-0.0873218129	9.293179867	12.4875010752
H	-0.9924890656	10.8105671414	12.618683409
C	-2.2561418088	7.5792881287	12.6663760042
H	-3.0495820414	7.0270394284	13.1824418298
H	-1.3149525918	7.0320061795	12.7935122681
H	-2.497702826	7.5885741279	11.5979011879
C	5.4554058087	3.2838262365	24.253275749
H	6.2050777844	2.9276686943	24.9718563178
H	5.6577201895	2.8136433828	23.2823342474
C	7.1678372621	4.9068299903	20.9978602148
H	6.7917775731	3.8843746384	21.0108484433
H	8.2487115914	4.8946799483	21.1714964404
H	2.7956592224	5.9664901579	18.54433012
H	4.2673197321	4.0093496677	18.7461488601
Cl	3.8795722288	11.4794145924	16.3253150097
Cl	9.5650499227	6.5850742275	14.4688476267

Variable-temperature (VT) ^{1}H NMR characterization

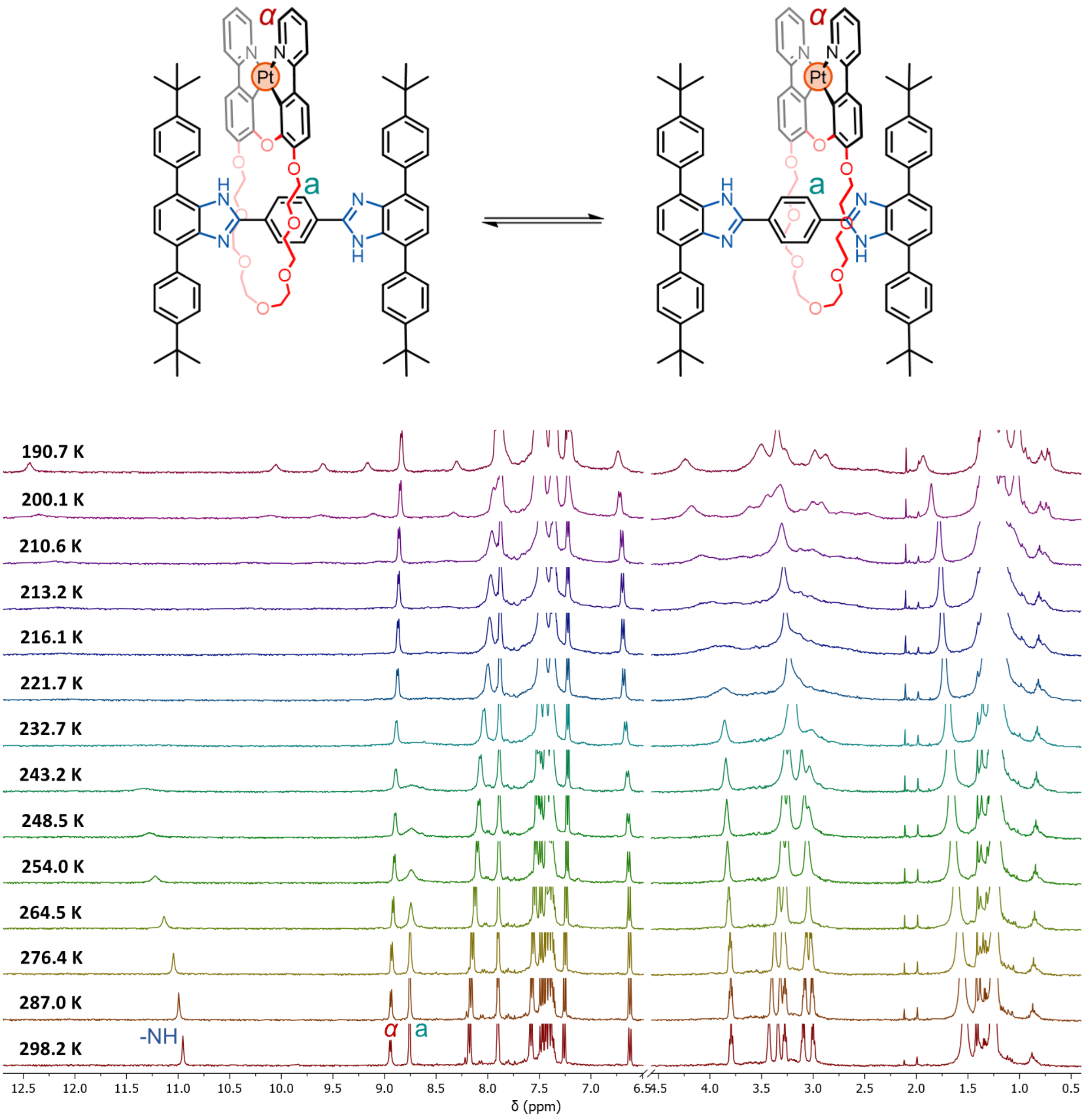

Figure S81. Variable-temperature (VT) ^{1}H NMR spectra (400 MHz, CD_2Cl_2) of compound **3-Pt^{II}** on cooling from 298 to 191 K.

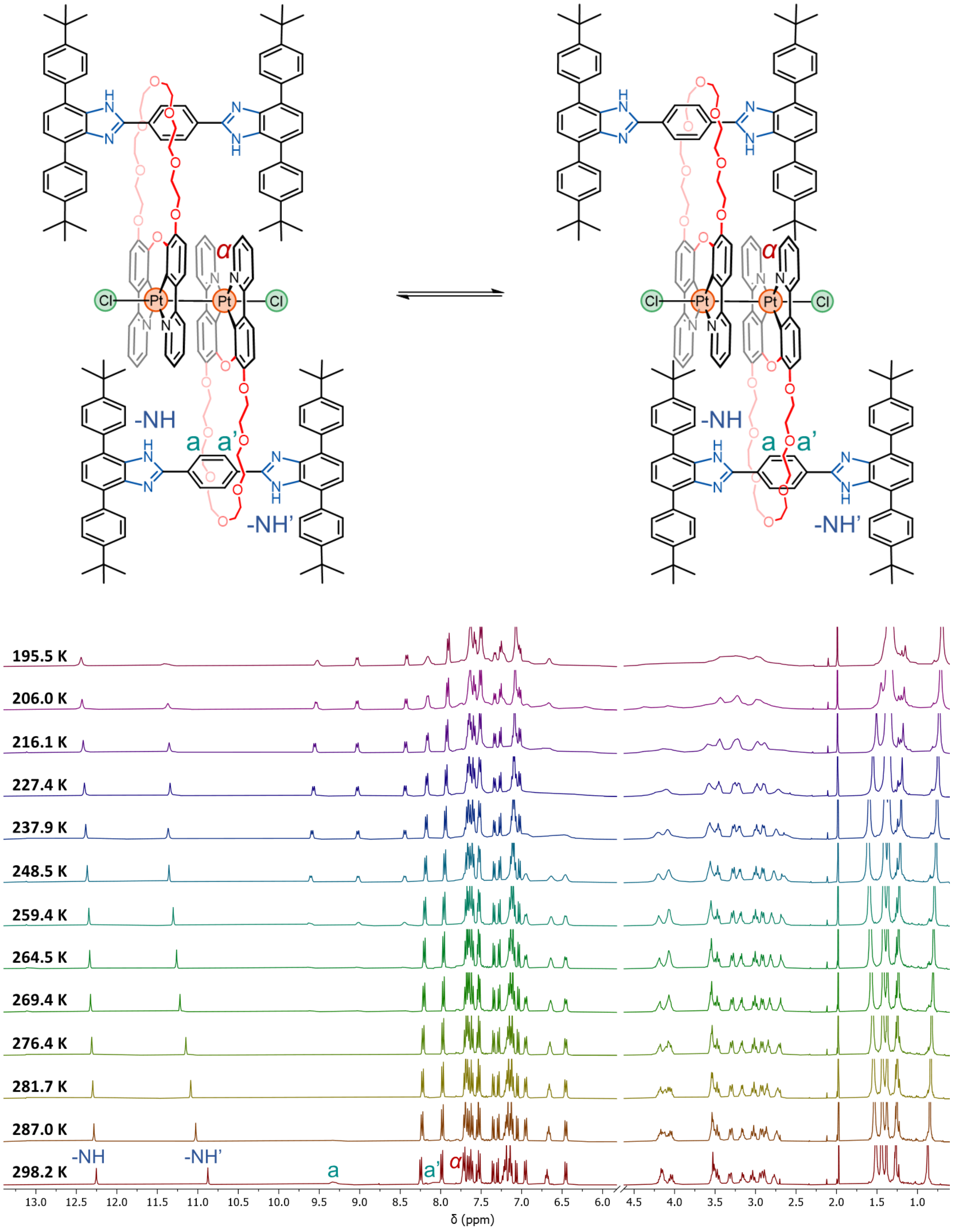

Figure S82. Variable-temperature (VT) ^{1}H NMR spectra (400 MHz, CD_2Cl_2) of **3-Pt^{III}** on cooling from 298 K to 196 K.

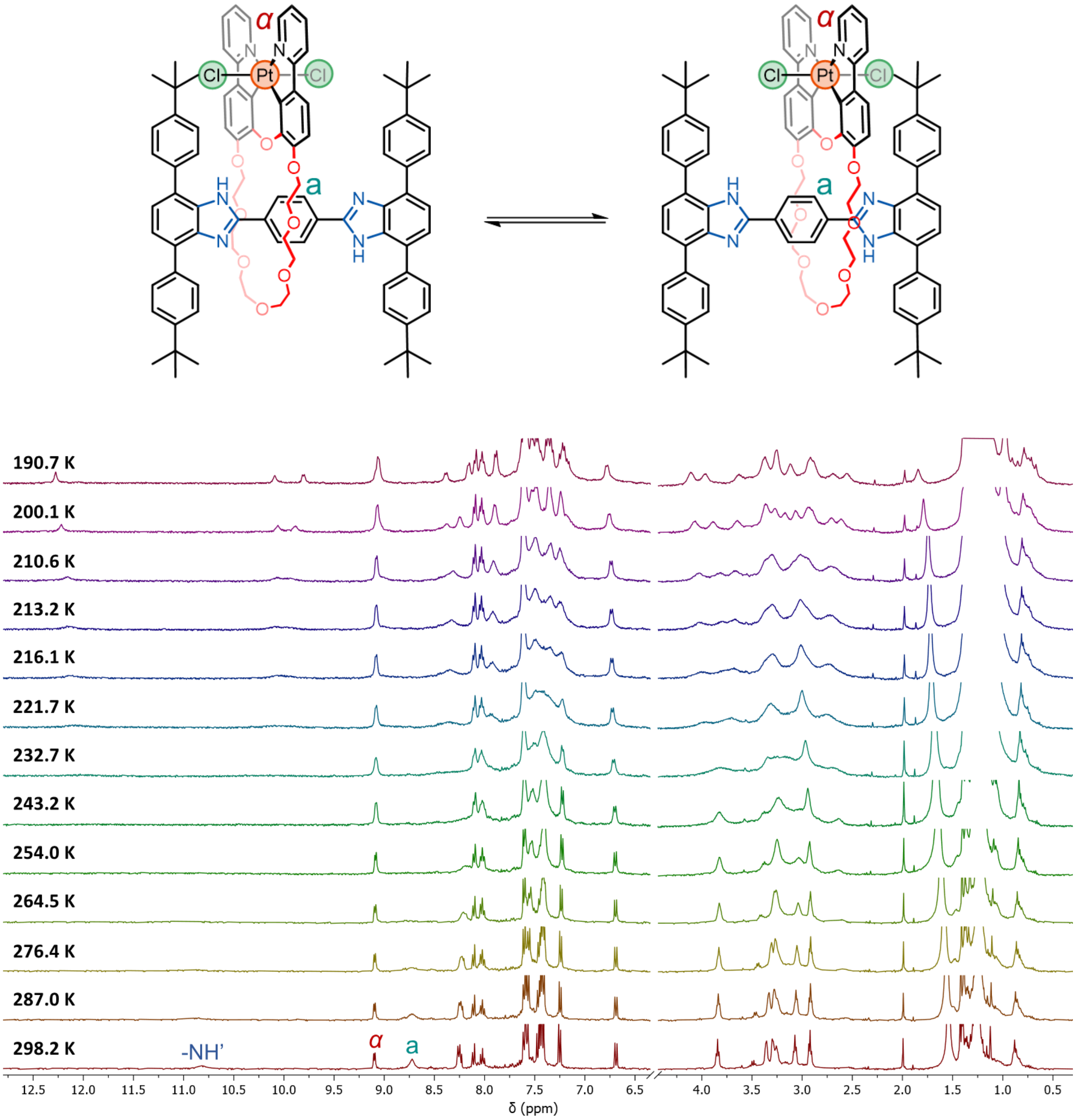

Figure S83. Variable-temperature ^{1}H NMR spectra (400 MHz, CD_2Cl_2) of **3-Pt^{IV}** on cooling from 298 to 190 K.

Determination of the shuttling rates

The rates of exchange were calculated by a coalescence temperature method:

$$k_c = (\pi \Delta v) 2^{1/2}$$

where k_c = the rate of shuttling at coalescence temperature and Δv = maximum peak separation in the low temperature (i.e., slow exchange) limit (in Hz). The Eyring equation,

$$\Delta G_c^{\neq} = -RT_c \ln(\frac{k_c h}{k_B T_c})$$

was used to estimate the Gibbs energy of activation ($\Delta G_c^{\neq}$) at the coalescence temperature (T_c), and this value was used to extrapolate the shuttling rate to room temperature (298 K).

Table S5. Rates of exchange ($\boldsymbol{k_c}$, $\boldsymbol{k}_{\mathbf{(298\,K)}}$) and Gibbs energy of activation ($\boldsymbol{\Delta G_c^{\neq}}$) for rotaxanes **3-Pt^{II}**, **3-Pt^{III}**, and **3-Pt^{IV}** obtained by the coalescence temperature method, using data from low-temperature VT NMR spectra (CD_2Cl_2, 298 K). The errors in the chemical shift and the temperature values were determined to be 0.1069 ppm and 0.2 K, respectively, and the error in $\boldsymbol{\Delta G_c^{\neq}}$ was estimated through error propagation of these values.

**Shuttle**	**Δυ (Hz)**	**T_c (K)**	**k_c (s^{-1})**	**$k_{(298\,K)}$ (s^{-1})**	**$\Delta G_c^{\neq}$ (kcal/mol)**
**3-Pt^{II}**	956	233	2123	3.1×10^5	9.96 ± 0.04
**3-Pt^{III}**	874	282	519	2.0×10^3	12.95 ± 0.04
**3-Pt^{IV}**	234	254	1942	5.7×10^4	10.96 ± 0.04

Solvent-excluded surface calculations

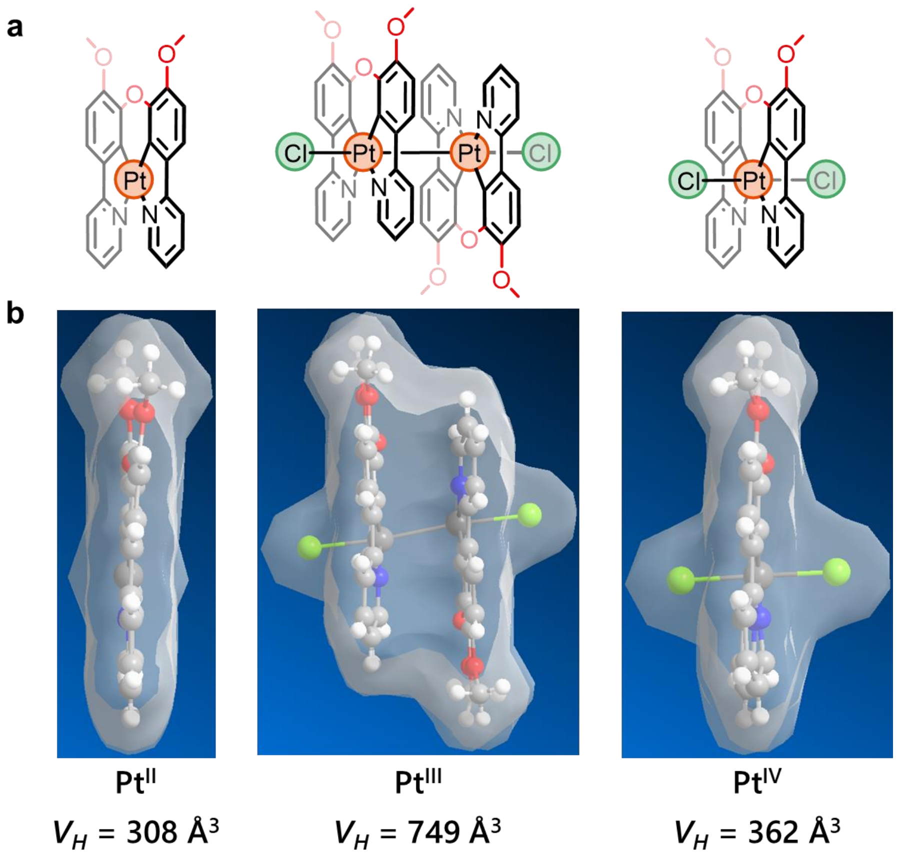

Figure S84. a. Chemical structures of the cyclometalated **Pt^II**, **Pt^III**, and **Pt^IV** complexes from our previous work.[1] b. Connolly solvent excluded volumes were computed for each complex in Chem3D using mol files from single-crystal x-ray structures; the volumes obtained are shown on the bottom of each corresponding image.

UV-Vis and emission spectra

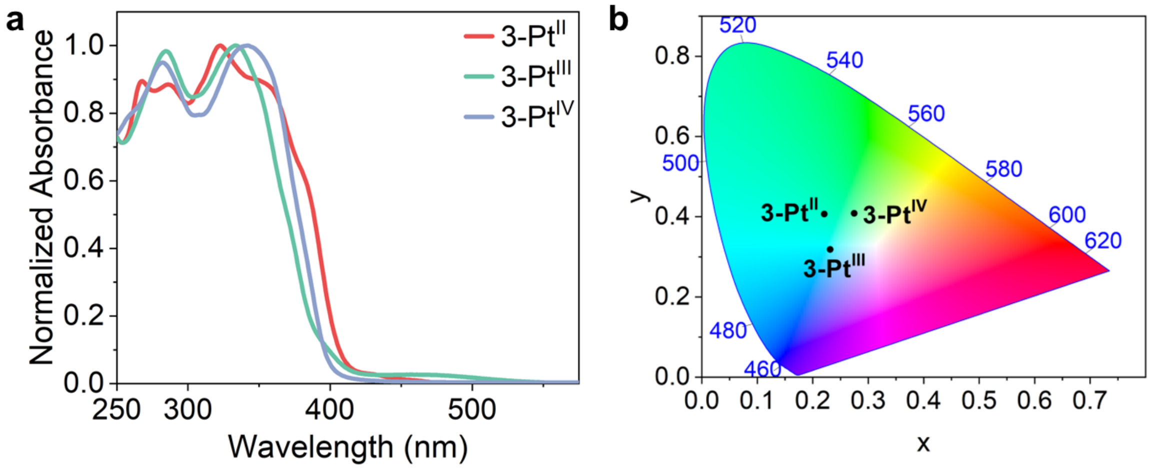

Figure S85. a. Spectra showing normalized absorbance for rotaxanes **3-Pt^{II}**, **3-Pt^{III}**, and **3-Pt^{IV}** in DCM. b. CIE plot of the three rotaxanes also in DCM.

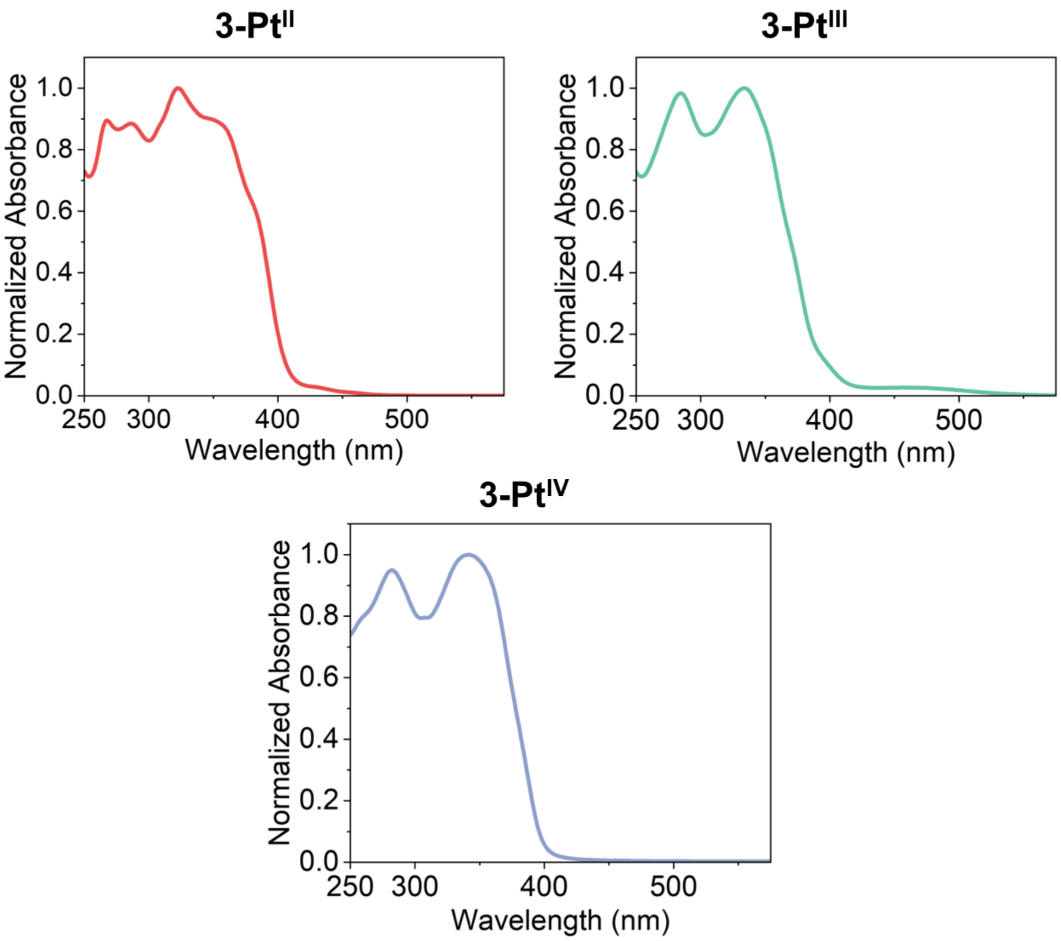

Figure S86. UV-vis spectra of rotaxanes **3-Pt^{II}**, **3-Pt^{III}**, and **3-Pt^{IV}** in DCM.

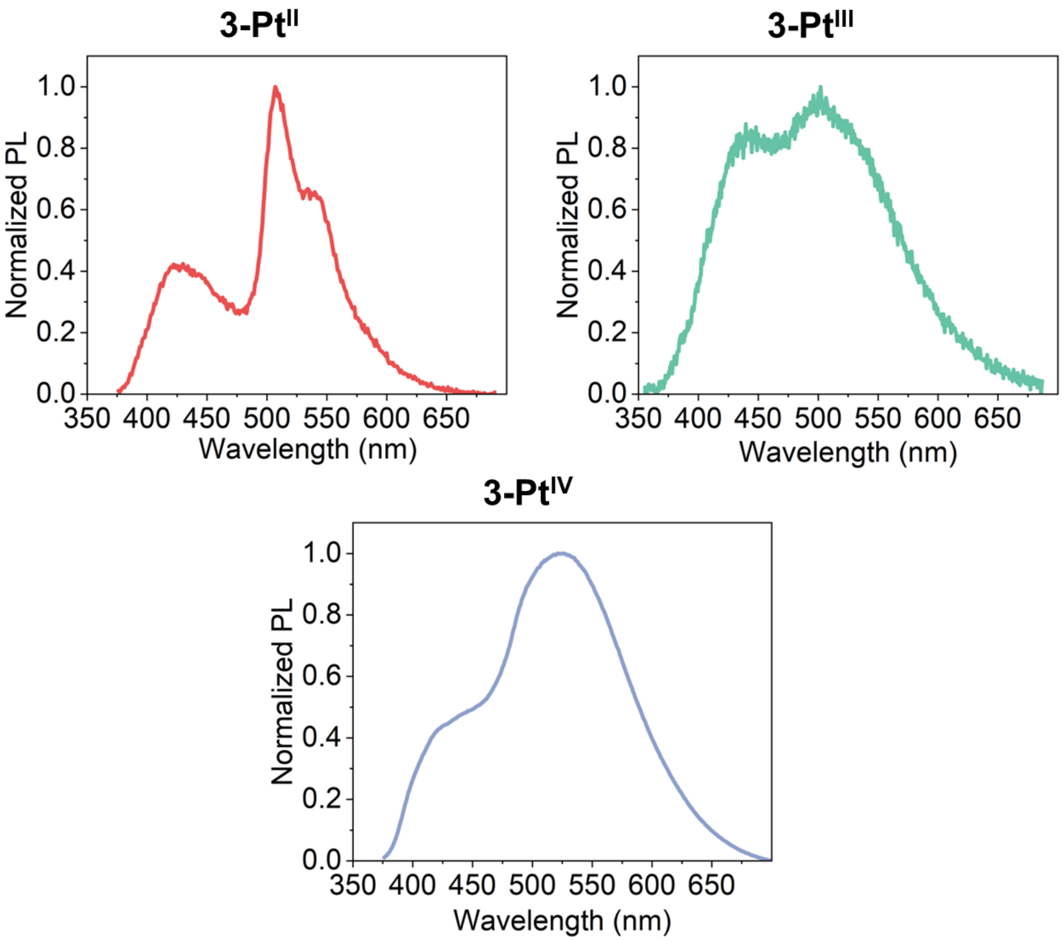

Figure S87. Photoluminescence spectra (PL) of rotaxanes **3-Pt^{II}**, **3-Pt^{III}**, and **3-Pt^{IV}** in DCM.

Crystallographic data

General

The structure was solved with the ShelXT[17] solution program using Olex2 1.5[18] as the graphical interface. The model was refined with ShelXL[19] using full matrix least squares minimization on F^2.

3[PF₆]

CCDC deposition number: 2372418

Experimental

Single green block-shaped crystals of **3[PF₆]** were obtained *via* a slow diffusion of pentane into an acetone solution of the compound. A suitable crystal with dimensions 0.36 × 0.20 × 0.08 mm³ was selected and mounted on a Bruker APEX II area detector diffractometer. The crystal was kept at a steady T = 100(2) K during data collection. Data were measured using ω and ϕ scans of 0.3° per frame per 18 s with MoK_α radiation (TRIUMPH monochromator, sealed X-ray tube, 45kV, 30mA). The maximum resolution that was achieved was Θ = 25.385° (0.83 Å).

Crystal Data. $C_{96.7}H_{106.4}F_6N_6O_{8.9}P$, M_r = 1640.09, triclinic, P-1 (No. 2), a = 17.0151(16) Å, b = 17.2012(17) Å, c = 32.991(3) Å, α = 99.7200(13)°, β = 100.4426(12)°, γ = 91.8945(12)°, V = 9339.5(16) Å³, T = 100(2) K, Z = 4, Z' = 2, $\mu(MoK_\alpha)$ = 0.099, 136740 reflections measured, 34276 unique (R_{int} = 0.0485) which were used in all calculations. The final wR_2 was 0.2458 (all data) and R_1 was 0.0811 ($I \geq 2\,\sigma(I)$).

Structure solution and refinement

The unit cell was refined using SAINT V8.40B (Bruker, V8.40B, 2016) on 9933 reflections, 7% of the observed reflections. The final completeness is 100% out to Θ = 25.385°.

Data reduction was performed using SAINT V8.40B (Bruker, V8.40B, 2016). SADABS-2016/2 (Bruker, 2016/2) was used for absorption correction. wR_2(int) was 0.0500 before and 0.0437 after correction. The ratio of minimum to maximum transmission is 0.8892. The λ/2 correction factor is not present. The absorption coefficient μ of this material is 0.099 mm^{-1} at this wavelength (λ = 0.71073Å) and the minimum and maximum transmissions are 0.882 and 0.992.

The structure was solved, and the space group P-1 (# 2) determined by the ShelXT[17] structure solution program using dual methods and refined by full matrix least squares minimization on $\boldsymbol{F^2}$ using version 2019/1 of ShelXL.[19] All non-hydrogen atoms were refined anisotropically. Most hydrogen atom positions were calculated geometrically and refined using the riding model, but some hydrogen atoms were refined freely. The value of Z' is 2. This means that there are two independent molecules in the asymmetric unit.

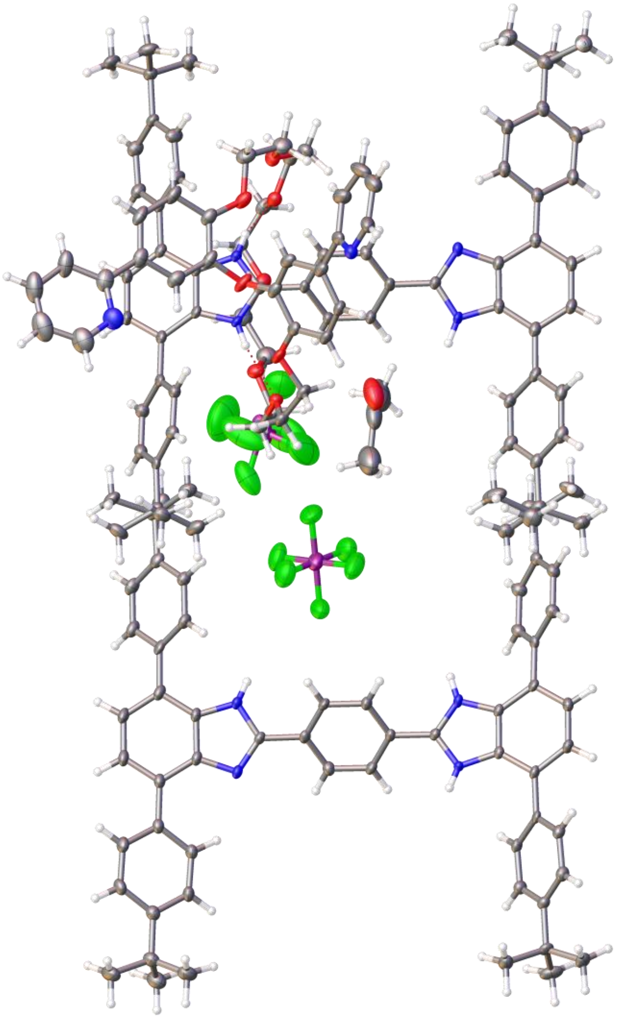

Figure S88. Molecular structure of **3[PF$_6$]** as determined by SCXRD. Ellipsoids are plotted at 50% probability. (The macrocycle used for the rotaxane formation was protonated using either HBF_4 (50-55 wt% in Et_2O) or HPF_6 (60% in H_2O). Both methods produced comparable yields.)

3-Pt^{II}

CCDC deposition number: 2372419

Experimental

Single yellow irregular-shaped crystals of **3-Pt^{II}** were obtained *via* a slow diffusion of pentane into an acetone solution of the compound. A suitable crystal with dimensions 0.32 × 0.14 × 0.12 mm^3 was selected and mounted on a Bruker APEX II area detector diffractometer. The crystal was kept at a steady T = 100(2) K during data collection. Data were measured using ω and ϕ scans of 1° per frame between 20 and 90 s with CuK$_a$ radiation (microfocus sealed X-ray tube, 45 kV, 0.60 mA). The maximum resolution that was achieved was Θ = 59.043° (0.90 Å).

Crystal Data. $C_{104.4}H_{120.2}N_6O_{10.3}Pt$, M_r = 1818.95, triclinic, *P*-1 (No. 2), a = 15.2274(3) Å, b = 15.7704(3) Å, c = 20.3260(4) Å, α = 78.8330(10)°, β = 86.8380(10)°, γ = 88.2440(10)°, *V* = 4780.43(16) Å^3, *T* = 100(2) K, *Z* = 2, *Z'* = 1, $\mu(CuK_\alpha)$ = 3.223, 13749 reflections measured, 13749 unique (R_{int} = 0.115) which were used in all calculations. The final wR_2 was 0.1484 (all data) and R_1 was 0.0582 (I≥2 σ(I)).

Structure solution and refinement

Since the crystal was twinned, data from the major twin domain was used for the refinements. The unit cell was refined using SAINT V8.40B (Bruker, V8.40B, 2016) on 9845 reflections, 72% of the observed reflections. The final completeness is 99.7% out to 59.043° in Θ.

Data reduction was performed using SAINT V8.40B (Bruker, V8.40B, 2016). A multi-scan absorption correction was performed using TWINABS-2012/1 (Bruker, 2012) was used for absorption correction. For component 1: wR_2(int) was 0.0865 before and 0.0665 after correction. For component 2: wR_2(int) was 0.1243 before and 0.0757 after correction. The ratio of minimum to maximum transmission is 0.80. Final HKLF 4 output contains 95191 reflections, R_{int} = 0.1151 (32729 with I > 3 σ(I), R_{int} = 0.0727). The absorption coefficient *m* of this material is 3.223 mm^{-1} at this wavelength (λ = 1.54178Å) and the minimum and maximum transmissions are 0.543 and 0.679.

The structure was solved, and the space group *P*-1 (# 2) determined by the ShelXT[17] structure solution program using dual methods and refined by full matrix least squares minimization on $\boldsymbol{F}^2$ using version 2019/1 of ShelXL[19]. All non-hydrogen atoms were refined anisotropically. Hydrogen atom positions were calculated geometrically and refined using the riding model.

There is a single formula unit in the asymmetric unit, which is represented by the reported sum formula. In other words: Z is 2 and Z' is 1.

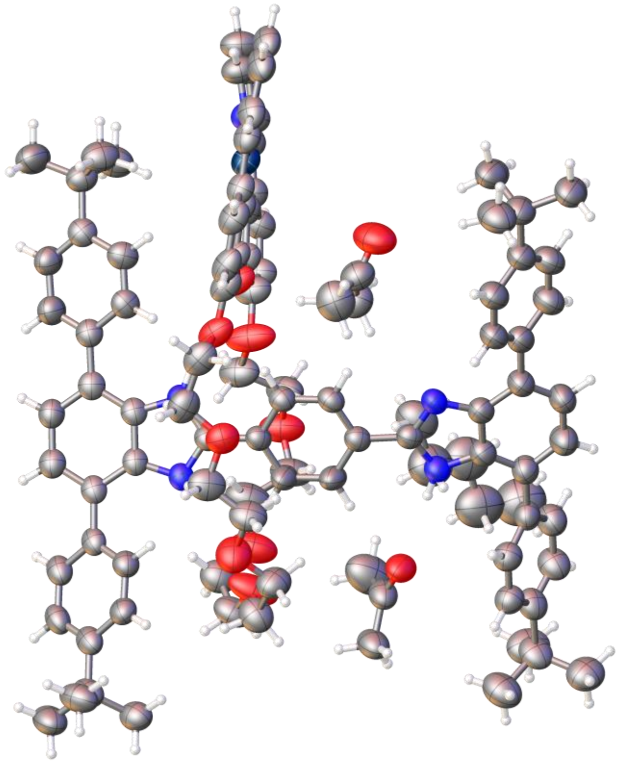

Figure S89. Molecular structure of **3-Pt^{II}** as determined by SCXRD. Ellipsoids are plotted at 50% probability.

3-Pt^{IV}

CCDC deposition number: 23724120

Experimental

Single yellow block-shaped crystals were recrystallized from dichloromethane by slow evaporation**.** A suitable crystal with dimensions 0.12 × 0.06 × 0.06 mm^3 was selected and mounted on a Bruker APEX-II CCD diffractometer. The crystal was kept at a steady T = 100(2) K during data collection. Data were measured using *w* and *f* scans of 0.5° per frame per 30 s with MoK$_{\alpha}$ radiation (TRIUMPH monochromator, sealed X-ray tube, 45kV, 30mA). The maximum resolution that was achieved was Θ = 25.472° (0.83 Å).

Crystal Data. $C_{94}H_{98}Cl_2N_6O_8Pt$, M_r = 1705.77, triclinic, *P*-1 (No. 2), a = 15.3075(12) Å, b = 16.2101(14) Å, c = 20.9954(16) Å, α = 73.166(3)°, β = 75.687(2)°, γ = 68.569(2)°, V = 4582.9(6) Å^3, T = 100(2) K, Z = 2, Z' = 1, μ(MoK$_{\alpha}$) = 1.644, 107696 reflections measured, 16966 unique (R_{int} = 0.0729) which were used in all calculations. The final wR_2 was 0.1133 (all data) and R_1 was 0.0488 (I≥2 σ(I)).

Structure solution and refinement

The unit cell was refined using SAINT V8.40B (Bruker, V8.40B, 2016) on 9658 reflections, 9% of the

observed reflections. The final completeness is 100% out to $\Theta = 25.472^{\circ}$.

Data reduction was performed using SAINT V8.40B (Bruker, V8.40B, 2016). SADABS-2016/2 (Bruker, 2016/2) was used for absorption correction. wR_2(int) was 0.0605 before and 0.0542 after correction. The ratio of minimum to maximum transmission is 0.9520. The λ/2 correction factor is not present. The absorption coefficient μ of this material is 1.644 mm^{-1} at this wavelength (λ = 0.71073Å) and the minimum and maximum transmissions are 0.709 and 0.745.

The structure was solved and the space group *P*-1 (# 2) determined by the ShelXT[17] structure solution program and refined by full matrix least squares minimization on $\boldsymbol{F}^2$ using version 2019/1 of ShelXL.[19] All non-hydrogen atoms were refined anisotropically. Hydrogen atom positions were calculated geometrically and refined using the riding model.

There is a single formula unit in the asymmetric unit, which is represented by the reported sum formula. In other words: Z is 2 and Z' is 1.

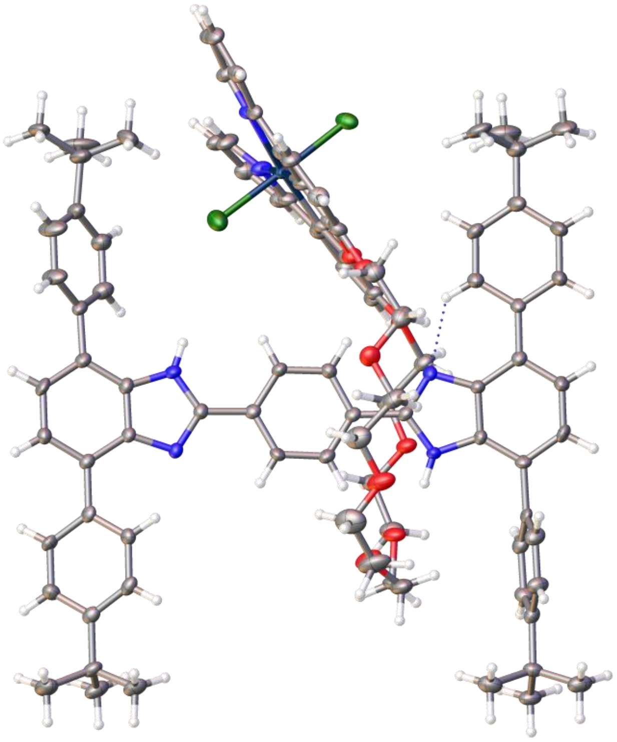

Figure S90. Molecular structure of **3-Pt^{IV}** as determined by SCXRD. Ellipsoids are plotted at 50% probability.

Table S6. Summary of crystallographic data and structure refinement for **3·H**, **3-Pt^{II}**, and **3-Pt^{IV}**.

	**3·H**	**3**	**3-Pt^{IV}**
Chemical Formula	$C_{96.7}H_{106.4}F_6N_6O_{8.9}P$	$C_{104.4}H_{120.2}N_6O_{10.3}Pt$	$C_{94}H_{98}Cl_2N_6O_8Pt$
Formula Weight	1640.09	1818.95	1705.77
Crystal size (mm)	0.36×0.20×0.08	0.32×0.14×0.12	0.12×0.06×0.06
Temperature (K)	100(2)	100(2)	100(2)
Crystal system	triclinic	triclinic	triclinic
Space group	*P*-1	*P*-1	*P*-1
Unit cell dimensions	a = 17.0151(16) Å<br>b = 17.2012(17) Å<br>c = 32.991(3) Å<br>α = 99.7200(13)°<br>β = 100.4426(12)°<br>γ = 91.8945(12)°	a = 15.2274(3) Å<br>b = 15.7704(3) Å<br>c = 20.3260(4) Å<br>α = 78.8330(10)°<br>β = 86.8380(10)°<br>γ = 88.2440(10)°	a = 15.3075(12) Å<br>b = 16.2101(14) Å<br>c = 20.9954(16) Å<br>α = 73.166(3)°<br>β = 75.687(2)°<br>γ = 68.569(2)°
Unit Cell Volume (Å^3)	9339.5(16) Å^3	4780.43(16)	4582.9(6)
Z	4	2	2
Absorption coefficient (mm^{-1})	0.099 mm^{-1}	3.223 mm^{-1}	1.644 mm^{-1}
Measured reflections	136740	49085	107696
Independent reflections	34276	13749	16966
Completeness to theta	25.385° (0.83 Å)<br>100.0 %	59.043° (0.90 Å)<br>99.7 %	25.472° (0.83 Å)<br>100.0 %
Goodness-of-fit on F^2	1.022	0.988	1.033
Final R_1 value [$I>2\sigma(I)$]	0.0868	0.0582	0.0488
Final wR_2 value (all data)	0.2004	0.1484	0.01133
Largest diff. peak and hole (e.Å^{-3})	0.796 and -0.823	1.075 and -0.432	2.578 and -1.199

References

[1] M. A. Soto, V. Carta, R. J. Andrews, M. T. Chaudhry, Mark. J. MacLachlan, *Angew. Chem. Int. Ed.* **2020**, *59*, 10348–10352.

[2] L. Ji, Z. Yang, Y. Zhao, M. Sun, L. Cao, X.-J. Yang, Y.-Y. Wang, B. Wu, *Chem. Commun.* **2016**, *52*, 7310–7313.

[3] N. Huang, P. Wang, M. A. Addicoat, T. Heine, D. Jiang, *Angew. Chem. Int. Ed.* **2017**, *56*, 4982–4986.

[4] M. J. Frisch, G. W. Trucks, H. B. Schlegel, G. E. Scuseria, M. A. Robb, J. R. Cheeseman, G. Scalmani, V. Barone, G. A. Petersson, H. Nakatsuji, X. Li, M. Caricato, A. V. Marenich, J. Bloino, B. G. Janesko, R. Gomperts, B. Mennucci, H. P. Hratchian, J. V. Ortiz, A. F. Izmaylov, J. L. Sonnenberg, D. Williams-Young, F. Ding, F. Lipparini, F. Egidi, J. Goings, B. Peng, A. Petrone, T. Henderson, D. Ranasinghe, V. G. Zakrzewski, J. Gao, N. Rega, G. Zheng, W. Liang, M. Hada, M. Ehara, K. Toyota, R. Fukuda, J. Hasegawa, M. Ishida, T. Nakajima, Y. Honda, O. Kitao, H. Nakai, T. Vreven, K. Throssell, J. A. Montgomery Jr., J. E. Peralta, F. Ogliaro, M. J. Bearpark, J. J. Heyd, E. N. Brothers, K. N. Kudin, V. N. Staroverov, T. A. Keith, R. Kobayashi, J. Normand, K. Raghavachari, A. P. Rendell, J. C. Burant, S. S. Iyengar, J. Tomasi, M. Cossi, J. M. Millam, M. Klene, C. Adamo, R. Cammi, J. W. Ochterski, R. L. Martin, K. Morokuma, O. Farkas, J. B. Foresman, D. J. Fox, **2016**.

[5] C. Adamo, V. Barone, *J. Chem. Phys.* **1999**, *110*, 6158–6170.

[6] Y. Zhao, D. G. Truhlar, *J. Phys. Chem. A* **2005**, *109*, 5656–5667.

[7] J.-D. Chai, M. Head-Gordon, *Phys. Chem. Chem. Phys.* **2008**, *10*, 6615–6620.

[8] W. J. Hehre, R. Ditchfield, J. A. Pople, *J. Chem. Phys.* **1972**, *56*, 2257–2261.

[9] M. M. Francl, W. J. Pietro, W. J. Hehre, J. S. Binkley, M. S. Gordon, D. J. DeFrees, J. A. Pople, *J. Chem. Phys.* **1982**, *77*, 3654–3665.

[10] D. Figgen, K. A. Peterson, M. Dolg, H. Stoll, *J. Chem. Phys.* **2009**, *130*, 164108.

[11] G. Scalmani, M. J. Frisch, *J. Chem. Phys.* **2010**, *132*, 114110.

[12] M. A. Soto, V. Carta, M. T. Cano, R. J. Andrews, B. O. Patrick, M. J. MacLachlan, *Inorg. Chem.* **2022**, *61*, 2999–3006.

[13] N. Noujeim, K. Zhu, V. N. Vukotic, S. J. Loeb, *Org. Lett.* **2012**, *14*, 2484–2487.

[14] K. Zhu, V. N. Vukotic, N. Noujeim, S. J. Loeb, *Chem. Sci.* **2012**, *3*, 3265–3271.

[15] I. Allison, H. Lim, A. Shukla, V. Ahmad, M. Hasan, K. Deshmukh, R. Wawrzinek, S. K. M. McGregor, J. K. Clegg, V. V. Divya, C. Govind, C. H. Suresh, V. Karunakaran, N. U. K. N., A. Ajayaghosh, E. B. Namdas, S.-C. Lo, *ACS Appl. Electron. Mater.* **2019**, *1*, 1304–1313.

[16] M. A. Soto, V. Carta, I. Suzana, B. O. Patrick, F. Lelj, M. J. MacLachlan, *Angew. Chem. Int. Ed.* **2023**, *62*, e202216029.

[17] G. M. Sheldrick, *Acta Crystallogr. Sect. A Found. Adv.* **2015**, *71*, 3–8.

[18] O. V. Dolomanov, L. J. Bourhis, R. J. Gildea, J. A. K. Howard, H. Puschmann, *J. Appl. Crystallogr.* **2009**, *42*, 339–341.

[19] G. M. Sheldrick, *Acta Crystallogr. Sect. C Struct. Chem.* **2015**, *71*, 3–8.
